# Effects of different foods and cooking methods on the gut microbiota: an *in vitro* approach

**DOI:** 10.3389/fmicb.2023.1334623

**Published:** 2024-01-08

**Authors:** Alberto M. Lerma-Aguilera, Sergio Pérez-Burillo, Beatriz Navajas-Porras, E. Daniel León, Sonia Ruíz-Pérez, Silvia Pastoriza, Nuria Jiménez-Hernández, Bettina-Maria Cämmerer, José Ángel Rufián-Henares, María José Gosalbes, M. Pilar Francino

**Affiliations:** ^1^Área de Genómica y Salud, Fundación para el Fomento de la Investigación Sanitaria y Biomédica de la Comunidad Valenciana-Salud Pública, Valencia, Spain; ^2^Departamento de Nutrición y Bromatología, Centro de Investigación Biomédica, Instituto de Nutrición y Tecnología de los Alimentos, Universidad de Granada, Granada, Spain; ^3^Instituto de Investigación Biosanitaria (ibs.GRANADA), Universidad de Granada, Granada, Spain; ^4^CIBER Epidemiología y Salud Pública, Instituto de Salud Carlos III, Madrid, Spain; ^5^Department of Food Chemistry and Analytics, Technische Universität Berlin, Berlin, Germany

**Keywords:** gut microbiota, 16S rRNA, *in vitro* fermentation, food, cooking method, personalized nutrition

## Abstract

To support personalized diets targeting the gut microbiota, we employed an *in vitro* digestion-fermentation model and 16S rRNA gene sequencing to analyze the microbiota growing on representative foods of the Mediterranean and Western diets, as well as the influence of cooking methods. Plant- and animal-derived foods had significantly different impacts on the abundances of bacterial taxa. Animal and vegetable fats, fish and dairy products led to increases in many taxa, mainly within the Lachnospiraceae. In particular, fats favored increases in the beneficial bacteria *Faecalibacterium*, *Blautia*, and *Roseburia*. However, butter, as well as gouda cheese and fish, also resulted in the increase of *Lachnoclostridium*, associated to several diseases. Frying and boiling produced the most distinct effects on the microbiota, with members of the Lachnospiraceae and Ruminococcaceae responding the most to the cooking method employed. Nevertheless, cooking effects were highly individualized and food-dependent, challenging the investigation of their role in personalized diets.

## 1 Introduction

Long and short-term dietary intake influence the structure and activity of the gut microbiota, as confirmed by the shifts in microbial community structure caused by short-term animal or plant-based diets (David et al., 2014), and by the strong association between enterotypes and long-term diets (Wu et al., 2011). Dietary patterns such as the “Western” diet alter the composition and metabolic activity of the microbiota, inducing changes suspected of contributing to growing epidemics of chronic illnesses such as obesity and type 2 diabetes (Gurung et al., 2020). Although there are only a few studies to date, growing evidence suggests that the chemical alterations undergone by foods during cooking may also play an important role in modulating the gut microbiota, for example by modifying phenolic compounds (Pérez-Burillo et al., 2018a; Shen et al., 2010; Carmody et al., 2019; Chen et al., 2020; Nissen et al., 2022; Cattivelli et al., 2023). These modulatory effects will depend on both the cooking method and the food, so that they should be studied separately for different foods.

Dietary interventions are likely tools for modulation of the gut microbiota. However, the large inter-individual variability in gut microbiota composition, due to differences in diet and other factors, leads to different host responsiveness to interventions. Furthermore, the responsive bacterial taxa may differ among individuals. Therefore, host and microbiota response to an intervention are difficult to predict; and this variability may influence the results of intervention studies and interfere with reproducibility (Healey et al., 2017). Ongoing research focuses on finding dietary manipulations aimed at promoting beneficial microorganisms that take into account host-microbiota relationships, with the aim of using personalized nutrition in therapeutic interventions and as a solution to tackle the variability challenge.

Nevertheless, intervention studies focused on gut microbiota modifications are difficult to perform, and the impact of a particular food cannot be assessed. For this reason, a variety of *in vitro* digestion and fermentation models that mimic human processes have been developed, allowing the assessment of the direct effects of foods on the microbiota (Nissen et al., 2020). Each approach has advantages and limitations, as, although *in vivo* studies provide more relevant physiological information, *in vitro* models are key for testing specific foods and for initial screenings. Furthermore, batch fermentation models enable short-time parallel cultures to carry out many fermentations simultaneously. Therefore, this is the best approach to characterize the behavior of the gut microbiota when fermenting many different foods, which can be tested one by one without the confounding factors of other foods.

The aim of this work is to study how the composition of gut microbiota communities responds to a wide variety of foods representative of “Western” and “Mediterranean” diets, as well as the influence of cooking methods. We have performed over 900 *in vitro* digestion and fermentation assays to test the effect of 55 different foods, raw or cooked with up to five different methods, on the microbiota of three healthy adult individuals. The characterization of the resulting fermentative microbiota has revealed which bacteria tended to increase or decrease when exposed to specific foods. This knowledge will be useful to refine dietary interventions aimed at modulating the gut microbiota, moving toward the goal of personalized nutrition.

## 2 Materials and methods

### 2.1 Study design

**FIGURE 1:**
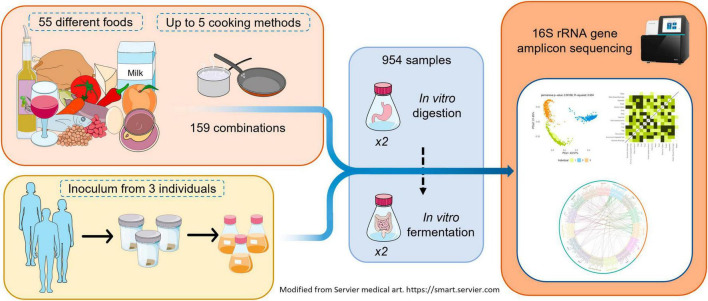
Graphic summary of study design.

Fifty-five different foods, raw or cooked with up to five cooking methods, resulting in 159 total combinations, were *in vitro* digested, and fermented to study their effect on the gut microbiota. Stools from three healthy adults were used as separate inocula and *in vitro* fermentations were performed in duplicate. A graphical summary of the study design is shown in Figure 1.

Due to the large number of foods used, they have been grouped at three different levels for the analysis of results: (i) Animal-based and plant-based foods, (ii) Food categories and (iii) Individual foods. The foods included at each level are described in Table 1.

**TABLE 1:** Foods employed in this study during *in vitro* digestions and fermentations.

Plant based/animal-based	Food category	Food	Raw	Fried	Boiled	Roasted	Grilled	Toasted
Animal-based	Meat	Chicken		•	•	•	•	
		Beef		•	•	•	•	
		Lamb		•	•	•	•	
		Pork		•	•	•	•	
	Dairy product	Milk	•					
		Yogurt	•					
		Gouda	•	•		•	•	
	Egg	Egg		•	•	•	•	
	Fish	Salmon	•	•	•	•	•	
		Cod fish		•	•	•	•	
	Animal and vegetable Fats	Butter	•	•				
Plant-based	Animal and vegetable fats	Olive oil	•	•				
		Sunflower oil	•	•				
	Fruit	Apple	•	•		•	•	
		Banana	•	•		•	•	
		Orange	•	•		•	•	
		Grapes	•	•		•	•	
		Plum	•	•		•	•	
		Peach	•	•		•	•	
		Olives	•					
	Grain-based product	Bread	•	•				•
		Bread whole grain	•	•				•
		Penne			•			
		Penne whole grain			•			
		Rice longo			•			
		Rice longo whole grain			•			
		Biscuits	•					
		Biscuits whole grain	•					
		Breakfast cereal whole grain	•					
		Breakfast cereal	•					
	Legumes	Beans kidney			•	•	•	
		Lentils			•	•	•	
	Nuts	Nut mix	•	•		•		
		Peanuts			•	•	•	
	Starchy tubers	Potato		•	•	•	•	
		Sweet potato		•	•	•	•	
	Sugar	Dark chocolate	•					
		Nutella	•					
	Vegetable	Zucchini	•	•	•	•	•	
		Capsicum	•	•	•	•	•	
		Carrot	•	•	•	•	•	
		Eggplant	•	•	•	•	•	
		Onion	•	•	•	•	•	
		Cauliflower	•	•	•	•	•	
		Spinach	•	•	•	•	•	
		Garlic	•	•	•	•	•	
		Tomato	•	•	•	•	•	
		Cabbage	•	•	•	•	•	
		Lettuce	•					
	Alcoholic beverage	Red wine						
		Beer						
	Water based beverage	Coffee						
		Coffee instant						
	Water based beverage	Cola						
		Cola light						

Different cooking methods performed with each food are indicated with a dot. Information is provided on which foods belong to which categories of the two food classifications employed. These two classifications as well as individual foods will be used in subsequent analyses.

### 2.2 Food and cooking methods

The foods and cooking methods employed are representative of Western and Mediterranean diets (Caradonna et al., 2020). We investigated 42 plant foods belonging to 10 different categories (alcoholic drinks, fruit, grain-based products, legumes, nuts, starchy tubers, sugar, vegetables, vegetable fats and water-based beverages), and 11 animal foods belonging to 4 categories (dairy products, egg, fish, and meat) (Table 1). In addition, the beverages cola and light cola were also investigated. Foods were bought in Spanish supermarkets and stored at room temperature or under refrigeration for a maximum of 2 days before cooking.

The food samples were submitted to different culinary treatments: boiling, grilling, roasting, frying, and toasting. In addition, some foods were also investigated in raw form. Milk was commercially processed by ultra-high temperature.

Boiling was performed at 100°C for 20 min at a water/food ratio of 5:1. Roasting was performed at 180°C for 10 min. Extra virgin olive oil was used as a cooking medium for grilling and frying. Grilling was performed at 220–250°C for 3 min on each side at an oil/food ratio of 0.5:1 and frying at 180°C for 8 min at an oil/food ratio of 5:1. Toasting was performed in a Grunkel TS140H toaster at the fourth level at 200°C for 3 min at 900 W, following the manufacturer’s instructions.

Cooking times and food/medium ratios were based on Ramírez-Anaya et al., 2015 and adapted to our equipment and laboratory conditions. Once cooked, all samples were homogenized and stored under nitrogen atmosphere at −80°C to avoid oxidation.

### 2.3 Fecal material collection

Fecal samples were obtained from three healthy donors, who had not taken antibiotics, prebiotics, or probiotics for 3 months prior to the assay, with a mean body mass index of 21.3. At least three fecal samples of each donor were obtained and pooled together to reduce intra-individual daily variability. Fecal donors followed a regular Mediterranean diet before sample collection.

Stools were deposited in a sterile recipient, stored in a home refrigerator, and transported to the laboratory in a cooler bag within 4 h. Upon arriving at the laboratory, the feces were mixed with a water:glycerol solution (20% vol/vol) and stored at −80°C.

### 2.4 *In vitro* gastrointestinal digestion and fermentation

All foods were submitted to *in vitro* batch digestion-fermentation mimicking physiological processes in the human gut, according to previously described protocols (Pérez-Burillo et al., 2018b,^2021^). For each food (in duplicate), 5 g were added to Falcon tubes and the three digestion phases were performed sequentially under agitation at 37°C: oral (adding α-amylase for 2 min), gastric (adding pepsin for 2 h at pH 2–3) and intestinal (adding bile salts and pancreatin for 2 h at pH 7). The *in vitro* fermentations were performed at 37°C for 20 h using the digested foods, the fecal microbiota and fermentation medium as described (Pérez-Burillo et al., 2021). The fermentation medium was composed of peptone solution (14 g L^–1^) at pH 7, 0.312 g L^–1^ of cysteine, 0.312 g L^–1^ of sodium sulfide and 1.25 mL L^–1^ of resazurin solution at 0.1% (wt/vol). This oligotrophic medium was suitable for testing the different foods as the main source of energy and nutrients for the microbiota, in order to highlight the bacterial community involved in the metabolism of each food. A fermentation control was performed using water in place of the digested substrate. Fermentations were centrifuged, and bacterial cells were recovered for taxonomical analysis by high throughput sequencing.

### 2.5 DNA extraction

Bacterial pellets derived from *in vitro* fermentations were lysed with 0.1 mg/ml lysozyme during 30 min at 37°C. DNA extraction was performed with the MagNaPure LC JE379 platform and DNA Isolation Kit III (Roche). DNA was quantified with a Qubit 3.0 Fluorometer (Invitrogen), while agarose gel electrophoresis (0.8% w/v agarose in Tris-borate-EDTA buffer) was used to determine DNA integrity. DNA was stored at −20°C until further processing.

### 2.6 16S rRNA gene amplicon sequencing

The V3-V4 hypervariable region of the 16S rRNA gene was amplified using 12 ng of DNA, following the Illumina protocol for 16S Metagenomic Sequencing Library Preparation. PCR was performed with forward primer (5′-TCGT CGGC AGCG TCAG ATGT GTAT AAGA GACA GCCT ACGG GNGG CWGCA-G3′) and reverse primer (5′-GTCT CGTG GGCT CGGA GATG TGTA TAAG AGAC AGGA CTAC HVGG GTAT CTAA TCC3′), fitted with adapter sequences for compatibility with the Illumina Nextera XT Index kit. Amplicon libraries were pooled and sequenced in an Illumina Miseq sequencer in 2 × 300 cycles paired-end runs (MiSeq Reagent kit v3).

### 2.7 Bioinformatic analyses

The DADA2 (v1.8.0) package as implemented in R (v3.6.0) was employed for sequence read processing and forward and reverse merging as well as clustering into amplicon sequence variants (ASVs) (Callahan et al., 2016). Filtering and trimming parameters were as follows: maxN = 0, maxEE = c(2, 5), truncQ = 0, trimLeft = c(17, 21), truncLen = c(270, 220), and rm.phix = TRUE. A minimum overlap of 15 nucleotides and a maximum mismatch of 1 were required for read merging.

Taxonomic identification was assigned to ASVs using DADA2 and the SILVA v.138 reference database. The MegaBLAST tool from BLAST v (2.10.0) was further used for those ASVs identified only at genus level with DADA2, requiring at least 97% identity for species-level assignation and a minimum difference of 2% between the first- and second-best matches. ASVs with a total number of counts lower than 10 were removed.

### 2.8 Statistical analysis

Alpha and beta diversity indices (Shannon, Chao1 and Bray–Curtis) were computed using the vegan package (v2.5-2) in the R platform. Differences in alpha-diversity were tested using the Wilcoxon rank-sum test. The Bray–Curtis dissimilarity index was employed to quantify the overall dissimilarity between two microbial communities and was used in permutational multivariate analysis of variance (PERMANOVA) and principal coordinates analysis (PCoA). PERMANOVA was performed using the adonis function from Vegan with 600 permutations. The Benjamini–Hochberg procedure was applied for false discovery rate control.

Analysis of the composition of microbiomes (ANCOM) was used to identify differentially abundant taxa among samples and test significance was determined using the Benjamini–Hochberg procedure for false discovery rate control, as described in Kaul et al., 2017. ANCOM was performed at the level of individual foods and at the two different levels of food classification for each individual, and significance was assigned at *q* < 0.05.

## 3 Results

16S rRNA gene amplicon sequencing of the microbiota in the 159 food combinations, after fermentation in duplicate with 3 different inocula, generated a total of 89,826,329 reads, averaging 93,277 reads per sample, 76,205,997 of which were assigned at genus-level. Taxonomic assignation identified reads belonging to 10 phyla, 65 families, 175 genera and 147 species.

### 3.1 Microbiota composition

**FIGURE 2:**
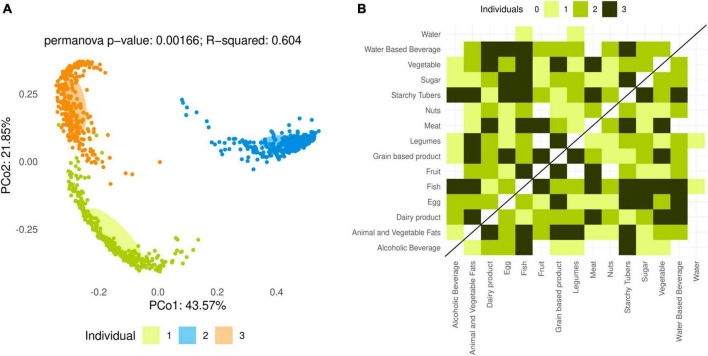
Analysis of overall microbiota composition after *in vitro* fermentations. **(A)** Principal coordinates analysis (PCoA) based on the Bray–Curtis dissimilarity index for all fermented food samples. Colors represent individuals 1, 2 and 3. PERMANOVA reveals significant differences in overall microbiota composition among individuals (*p* = 0.00166). **(B)** Number of individuals with significant (*q* < 0.05) PERMANOVA between food categories based on the Bray–Curtis dissimilarity index.

Significant differences in bacterial composition (Figure 2A) and diversity (Shannon index and Chao1 estimator, *q* < 0.001) were detected among the microbiota of the three analyzed individuals. Moreover, most of the variance in microbiota composition when all samples were considered was caused by differences among individuals. Therefore, the effect of foods and cooking methods was studied separately for each individual in all following analyses.

Permutational multivariate analysis of variance (PERMANOVA) revealed that, in each individual, the overall composition of the microbiota was significantly different (*q* < 0.01) between fermentations of plant and animal-based foods. At the level of lower food categories, few significant differences in overall microbiota composition were detected between individual categories and the water control (for fish and legumes, in one individual only), but numerous differences existed between different food categories (Figure 2B). This may be due to different foods modifying the microbiota composition in different directions, resulting in larger differences between two foods than between each and the water control. In all three individuals, starchy tubers and fish resulted in significantly different microbiota compositions when compared to the largest number of other foods (Figure 2B).

### 3.2 Differential abundance of taxa

**FIGURE 3:**
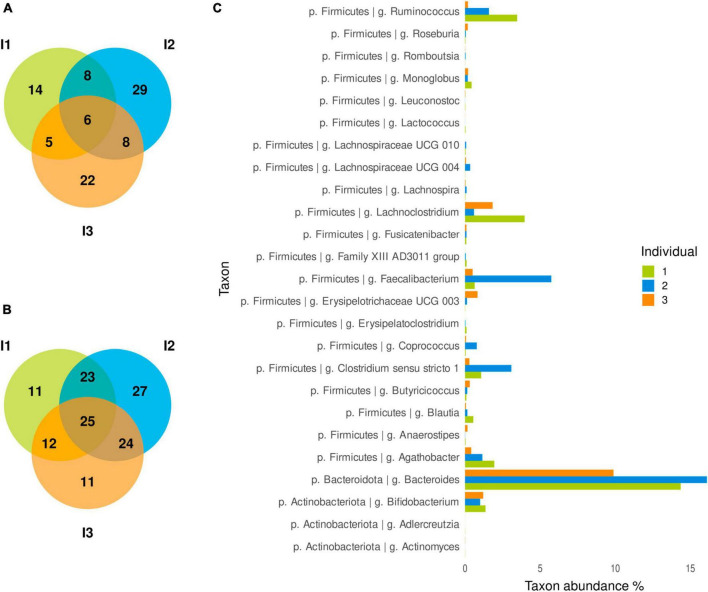
Differentially abundant genera shared among individuals. **(A)** Venn diagrams between plant-based vs. animal-based foods and **(B)** different food categories. Differential abundances were assessed with the ANCOM method and the Benjamini–Hochberg procedure for false discovery rate control (*q* < 0.05). Differences are considered common among individuals when changes take place in the same direction. **(C)** Relative abundance of differentially abundant genera in food category comparisons. The Y-axis presents the 25 genera detected as differentially abundant for a given food comparison in all individuals. Abundances were normalized by Total Sum Scaling. The mean relative abundance for all samples from an individual is presented.

Of 175 taxa identified at genus level, 78 were found in all individuals. Of these, 6 taxa were significantly more abundant in the fermentations of animal-based foods than in those of plant-based foods in each individual (Figure 3A): *Faecalibacterium*, *Lachnoclostridium*, Lachnospiraceae UCG 004, *Fusicatenibacter*, *Romboutsia*, and *Actinomyces*. In addition, 21 other taxa were significantly different between these fermentations for two individuals, and many more differences were specific to a single individual (Figure 3A). Taxa present across individuals could have differential abundances between animal- and plant-based foods in one individual but not in others, as most of the taxa that were differential in only one individual were also present in the other two. At food category level, 49, 59 and 25 genera were differentially abundant for a given comparison in one, two or three individuals, respectively (Figure 3B). Again, taxa present across all three individuals could behave differently in each. Of the 25 genera in which differences were detected for a given comparison in all individuals, the following stand out for their higher abundance: *Bacteroides*, *Faecalibacterium*, *Lachnoclostridium*, *Ruminococcus*, *Bifidobacterium*, and *Agathobacter* (Figure 3C).

**FIGURE 4:**
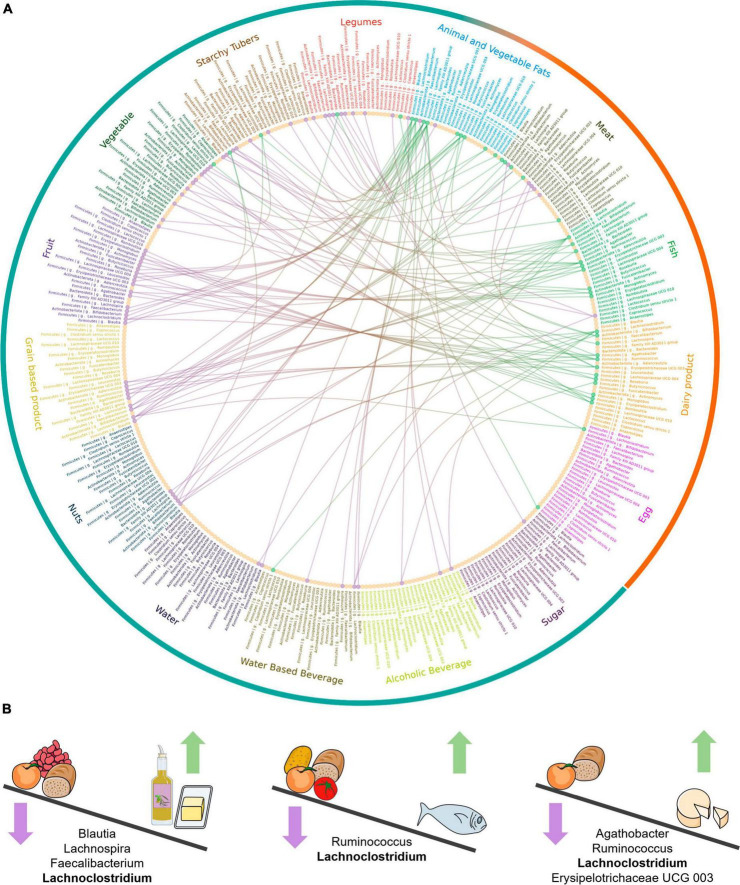
Genus-level abundance differences between food categories. **(A)** Circos plot showing differentially abundant genera between food categories. The same 25 genera, with the same order, in which common differences have been detected across all individuals, are represented in each food category. The fifteen food categories are shown with different colors and the wider level of classification, animal and plant-based foods, is shown in orange and blue in the outermost circle. The lines represent comparisons significant across all individuals between two food categories for a given taxon. Green and purple indicate higher and lower abundance in the food category. The dots for each taxon are colored to indicate whether most of their differences were increased abundances (green), decreased abundances (purple) or whether there was no difference (yellow). **(B)** Graphical summary of main differential outcomes in relative taxon abundance between food categories. The Figure was partly generated using Servier Medical Art, provided by Servier, licensed under a Creative Commons Attribution 3.0 unported license.

A genus-level summary of the food category comparisons significant in all the individuals is shown in Figure 4; also, all significant comparisons for food categories and individual foods can be found in further detail in Supplementary Figures 1–4, at both genus and species level. In the following sections we review the food categories having the strongest impacts on microbiota composition, indicating those taxa whose abundance is differentially affected across all three individuals, and highlighting relevant differences among specific foods within each category.

#### 3.2.1 Effect of animal and vegetable fats

Animal and vegetable fats was the category that resulted in the most significant effects on taxon abundance. The abundance of the Lachnospiraceae genera *Blautia*, *Lachnoclostridium*, *Lachnospira* and *Roseburia*, and of the Oscillospiraceae genus *Faecalibacterium* (*F. prausnitzii*), increased with animal and vegetable fats compared with fruits, legumes, grain-based products (excluding *Roseburia*) and water-based beverages (excluding *Faecalibacterium*). In particular, butter showed the highest number of significant comparisons of all foods tested in any food category, resulting in higher abundances of *Faecalibacterium* and the aforementioned Lachnospiraceae genera, as well as *Agathobacter* and *Fusicatenibacter*, in comparison with many other foods. In general, olive oil also induced significantly higher abundances of most of these genera, but only in comparison to a smaller array of foods. However, *Blautia* was not found at higher abundances in olive oil in comparison to any other food. Regarding sunflower oil, the number of significant comparisons was lower than for the other fats, with only *Faecalibacterium* and *Agathobacter* showing higher abundances in comparison to various other foods. *Faecalibacterium* was increased in all three types of fats for a similar number of comparisons to foods belonging to a variety of plant-based categories.

#### 3.2.2 Effect of fish

Fish fermentations led to higher abundances of numerous genera, most notably *Lachnoclostridium* and *Ruminococcus*, which were elevated with respect to 6 and 5 plant-based categories, respectively. Several genera showed higher abundances in fish fermentations when compared to those of fruits, including the two aforementioned plus Erysipelotrichaceae UCG 003, *Fusicatenibacter*, *Actinomyces*, and *Monoglobus*. In contrast, *Clostridium sensu stricto* 1 had lower abundances in fish fermentations in comparison to vegetables, legumes, and water-based beverages.

At food level, both salmon and cod induced higher abundances of *Lachnoclostridium* and *Ruminococcus* in comparison to a variety of plant-based foods. In addition, cod was involved in a very high number of significant comparisons, nearing those identified with butter, often resulting in higher abundances of *Faecalibacterium*, *Monoglobus*, and *Bifidobacterium*.

#### 3.2.3 Effect of dairy products

Dairy products resulted in a higher abundance of *Bifidobacterium* compared with animal and vegetable fats, meat, and nuts. Firmicutes genera, such as *Agathobacter*, *Lachnoclostridium*, *Ruminococcus* (*R. bromii*), *Romboutsia* and Erysipelotrichaceae UCG 003, were also often increased in dairy products in comparison to plant-based food categories, such as fruit and grain-based products, and, to a lesser extent, vegetables, and legumes. In particular, all of these genera were more abundant in dairy product fermentations than in those involving fruits. At food level, gouda was involved in a high number of significant comparisons, showing higher abundances of *Lachnoclostridium*, *R. bromii*, *Romboutsia*, and Erysipelotrichaceae UCG 003 when compared to a variety of plant-based foods. In contrast, yogurt resulted in a single significant comparison, with *Roseburia* in lower abundance in comparison to butter, while no significant comparisons involving milk fermentations were common among the three individuals.

#### 3.2.4 Effect of fruit

Fruit reduced the abundance of a large number of genera (17) in comparison to at least one other food. However, most of these genera were reduced only with respect to one or two food categories, mostly animal and vegetable fats, fish or dairy.

Apple was the fruit resulting in the most differences, all representing reduced abundances. *Agathobacter* was reduced in the largest number of comparisons, with animal-based foods such as butter, salmon, beef and chicken, but also with plant-based foods such as biscuits, nuts, vegetable oils, cabbage, carrot, and plum. *Lachnoclostridium*, *Faecalibacterium*, *Fusicatenibacter*, *Dorea*, and *Butyricicoccus* were also reduced in apple fermentations compared to various other foods. The comparisons of apple against butter and gouda showed the highest number of reduced taxa (>12 in each). Similar results were obtained for bananas, which showed reduced abundances of 7 taxa in relation to butter and gouda. Also, oranges, grapes, plums and peaches showed reduced abundances in various taxa in comparison to butter. However, plums were unique among fruits in increasing the abundances of *Bacteroides* and *Ruminococcus* in relation to several other foods. On the other hand, peaches induced a significantly higher abundance of *Lachnoclostridium* than most other fruits, tomato, and whole-grain bread.

#### 3.2.5 Effect of vegetables

Similarly, to fruits, vegetables affected the abundance of a relatively large number of genera (11) but only in respect to one or two food categories in each case. In most instances, vegetables showed lower abundances of taxa in comparison with animal and vegetable fats, dairy products or fish.

At food level, results were also similar to those obtained with fruits; most of the significant comparisons were against butter, gouda, codfish and salmon. Onion resulted in lower abundances of *Lachnoclostridium*, *Ruminococcus*, and *Faecalibacterium* compared with these four foods, while tomato significantly reduced *Lachnoclostridium* and *Dorea* in comparison to these foods and several others. Garlic is a singular case as it showed differences against a large number of foods, including lower abundances of *Ruminococcus* and *Fusicatenibacter*, and higher abundances of *Bacteroides*. In particular, *Bacteroides* increased in garlic in relation to other plant foods such as capsicum, apple, nut mix and sweet potato, as well as animal foods such as gouda and salmon.

#### 3.2.6 Effect of grain-based products

As in the cases of fruits and vegetables, grain-based products reduced the abundances of several genera (9) only in respect to one or two food categories, mainly animal and vegetable fats, dairy products, or fish. *Lachnoclostridium* stands out as being reduced not only in relation to these three food categories, but also in comparison to egg and meat.

Most of the individual foods within this category, similarly to fruits and vegetables, differed in many taxa in relation to butter and gouda, and, to a lesser degree, in relation to salmon, cod and olive and sunflower oils. Interestingly, whole-grain bread, but not regular bread or whole-grain biscuits, reduced *Lachnoclostridium* in relation to a variety of foods, including butter, gouda, cod, salmon, peach and plum.

## 3.3 Effects of cooking methods on the fermentative microbiota

When taking together all food categories, only the comparison between fried and roasted foods yielded a significant difference in taxon abundance, specifically for the low-abundant Ruminococcaceae UBA1819 group, which was significantly reduced in fried foods in all individuals. Since the effects of cooking methods could be masked by the effects of the different food categories, we analyzed them separately within each.

**FIGURE 5:**
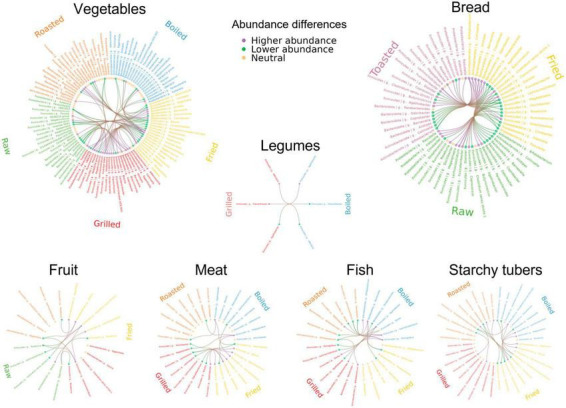
Circos plots of genus-level abundance differences between cooking methods by food category. Cooking methods and genera involved in significant comparisons for at least one individual are shown for each food category. Cooking methods are represented with different colors and differentially abundant genera are shown in the same order for all cooking methods. The lines represent significant comparisons between two cooking methods. Green and purple indicate higher and lower abundance of the corresponding genus. The dots for each genus are colored to indicate whether most of the differences were increased abundances (green), decreased abundances (purple) or whether there was no difference (yellow).

**FIGURE 6:**
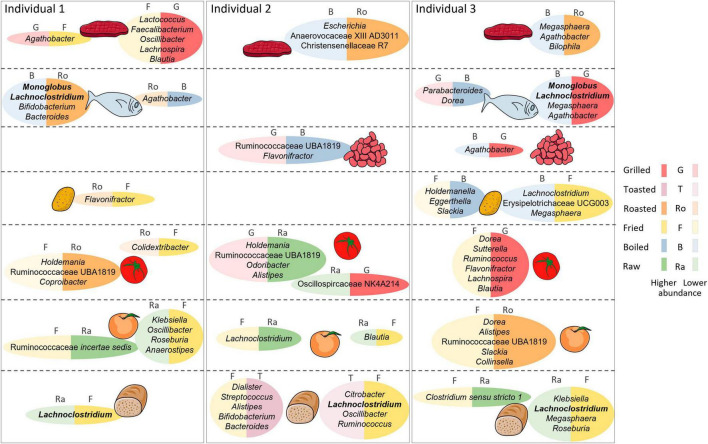
Graphical summary of main differential outcomes in relative taxon abundance between cooking methods. Differences are shown per individual and food category. For each individual, the two most differential cooking methods for each food category are shown, along with representative taxa whose abundance is reduced with the corresponding cooking method. The Figure was partly generated using Servier Medical Art, provided by Servier, licensed under a Creative Commons Attribution 3.0 unported license.

No significant differences in bacterial abundance were found in all individuals when comparing cooking effects within each food category, but a few common differences were found in two individuals and many in single individuals. All genus-level significant differences are shown in Figure 5 and Supplementary Figures 5, 6 presents the significant differences at species level. In the following sections, we review the food categories for which cooking methods had a stronger impact, indicating those taxa that were the most affected. A summary of the effects of the most differential cooking methods within each food category is presented in Figure 6.

### 3.3.1 Cooking methods in fish

Salmon and cod were boiled, fried, roasted, and grilled. Differences in taxon abundance depending on the cooking method were observed in individuals 1 and 3, but not in individual 2. Boiling resulted in the most marked differences, mostly in comparison to roasting in individual 1 and to grilling in individual 3. Overall, differences were observed mainly in the abundances of members of the Firmicutes, with boiling producing lower abundances of numerous genera, particularly in the Lachnospiraceae, Ruminococcaceae and Erysipelotrichaceae families. In particular, *Monoglobus* and *Lachnoclostridium* were reduced in both individuals 1 and 3 in comparison to roasting. The abundances of some Proteobacteria, as well as those of *Bacteroides* and *Bifidobacterium*, were also impacted by boiling, mostly in comparison to roasting in individual 1. Only 3 genera increased in boiled fish compared with other cooking methods: *Agathobacter* in individual 1 in relation to roasting, and *Dorea* and *Parabacteroides* in individual 3 in relation to grilling.

### 3.3.2 Cooking methods in meat

Like fish, meats were boiled, fried, roasted and grilled. Again, boiling resulted in the most marked differences, mainly consisting of reduced abundances of Firmicutes taxa in comparison to roasting in individuals 2 or 3. Of notice, in individual 3 boiling had a strong impact on the abundance of *Megasphaera*, which was reduced in relation to all other cooking methods. Only *Lachnoclostridium* increased in boiled meat compared to other methods, augmenting in individual 1 in relation to frying. Notable differences were also obtained when comparing frying and grilling, with frying reducing the abundances of the Firmicutes genera *Faecalibacterium*, *Oscillibacter*, *Lachnospira*, and *Blautia* in individual 1 and of *Lactococcus* in both individuals 1 and 2. In contrast, *Agathobacter* was increased in fried versus grilled meat in individual 1, as well as in fried versus boiled meat in individual 3.

### 3.3.3 Cooking methods in fruit

The effects of fruits (excluding olives, which were only analyzed raw) were compared when raw, fried, roasted or grilled. Frying and grilling resulted in a variety of differences in comparison to raw fruit, while there were no differences in roasted fruit versus raw. Fried fruits resulted in the most marked differences. In particular, frying decreased the abundances of many taxa in individual 3 in relation to roasting, including those of various Ruminococcaceae, *Dorea*, *Clostridium sensu stricto 1*, *Alistipes*, *Slackia*, and *Collinsella*. Differences between fried and raw fruits were observed in all individuals, although affecting different taxa in each: in individual 1, frying resulted in increases of *Klebsiella*, *Oscillibacter*, *Roseburia* and *Anaerostipes* and a reduction of Ruminococcaceae *incertae sedis*, in individual 2 it resulted in the increase of *Blautia* and the reduction of *Lachnoclostridium*, whereas in individual 3 it resulted in the reduction of Ruminococcaceae UBA1819, *Clostridium sensu stricto* 1 and three Eggerthellaceae genera.

In turn, grilled fruit also resulted in a variety of differences when compared to raw, some of which were also detected in comparison to roasted. Shared differences included reduced abundances of Ruminococcaceae UBA1819 and *Clostridium sensu stricto* 1 in individual 3. In addition, *Caproiciproducens*, *Peptoniphilus*, and Ruminococcaceae *incertae sedis* were reduced in grilled fruits in comparison only to roasted, in individuals 1, 2 and 3, respectively. Individual 1 also had increases of many taxa in grilled fruits in comparison to raw, including *Parasutterella*, *Lachnoclostridium*, *Oscillibacter*, and Oscillospiraceae UCG005.

### 3.3.4 Cooking methods in vegetables

The effects of vegetables (excluding lettuce, which was only analyzed raw) were compared when raw, boiled, fried, roasted or grilled. As for fruits, raw vegetables presented a variety of differences in comparison to those fried and grilled, while no differences were detected against those boiled or roasted. Again, frying resulted in the most marked differences, resulting mainly in taxon increases, including those of numerous genera of the Firmicutes, mostly in the Lachnospiraceae. The microbiota of individual 3 was particularly impacted by frying, showing a large number of differences in comparison to grilling, mostly not detected in the other individuals. These included mainly increases of genera in the families Ruminococcaceae, Oscillospiraceae and Lachnospiraceae. Among the latter, many were increased in individual 3 also when fried vegetables were compared to other cooking methods, with *Lachnoclostridium* and *Blautia* augmented in relation to all. Frying also resulted in decreases of some genera, including the Bacteroidota *Alistipes*, *Coprobacter*, and *Barnesiella*, the Actinobacteriota *Eggerthella*, and various Firmicutes. Among these, Ruminococcaceae *incertae sedis* is of note, as it was decreased in fried vegetables in relation to raw, boiled and roasted in individuals 1 and 3.

In contrast to frying, grilling resulted mainly in lower abundances of taxa in relation to raw vegetables and other methods of cooking. Of note, Ruminococcaceae UBA1819 was reduced in grilled vegetables in relation to raw, boiled and roasted in one or two individuals. In individual 3, numerous other taxa were also reduced with grilling in relation to one or two cooking methods, whereas in individual 2 other taxa were reduced with grilling but only in relation to raw. On the other hand, some Firmicutes taxa increased in grilled vegetables in relation to other methods, notably *Oscillospira* and Oscillospiraceae NK4A214 in individual 2.

### 3.3.5 Cooking methods in legumes

Kidney beans and lentils were assayed boiled, roasted, and grilled. Differences were only observed between boiling and grilling, with the former causing a reduction of *Agathobacter* in individual 3 and an increase of Ruminococcaceae UBA1819 and *Flavonifractor* in individual 2.

### 3.3.6 Cooking methods in starchy tubers

Potatoes and sweet potatoes were assayed fried, boiled, roasted, and grilled. Roasting displayed the most differences with other methods, although nearly all were detected only in individual 3. In particular, roasting resulted in reductions of *Flavonifractor*, *Roseburia* and *Lachnoclostridium* and in increases of Erysipelotrichaceae UCG003 and *Holdemanella* in relation to frying, as well as in increases of Erysipelotrichaceae UCG003 in relation to boiling, and of *Holdemanella* and *Clostridium sensu stricto* 1 in relation to grilling. Individual 3 also presented several differences between frying and boiling, with reductions of *Holdemanella*, *Slackia* and *Eggerthella* and increases of *Megasphaera*, *Lachnoclostridium* and Erysipelotrichaceae UCG003 with the former method. Therefore, for individual 3 the Erysipelotrichaceae (Erysipelotrichaceae UCG003 and *Holdemanella*) and Eggerthellaceae (*Eggerthella* and *Slackia*) appear to be particularly sensitive to the cooking methods employed for starchy tubers.

### 3.3.7 Cooking methods in bread

Among grain-based products, comparisons of cooking methods could only be performed for breads (white and whole grain) since other products were not exposed to different cooking procedures. Frying caused several differences in relation to both raw and toasted breads, mostly in individual 2, while the latter methods did not differ. Of note, *Lachnoclostridium* was increased in fried bread in relation to both raw and toasted in individuals 2 and 3. Numerous other taxa, mainly belonging to the Firmicutes, were increased or decreased in fried bread in relation to both cooking methods in individual 2. Of these, individual 3 shared an increase of *Fusicatenibacter* in relation to raw and a decrease of *Bifidobacterium* in relation to toasted. In addition, the Enterobacteriaceae *Raoultella* and *Citrobacter*, which can behave as opportunistic pathogens, were increased in fried bread in relation to raw and toasted in individual 2, and *Klebsiella*, another potential pathobiont of the same family, was increased in individual 3 in relation to raw. In addition, several taxa were increased in fried bread only in relation to toasted in individual 2, while others were increased only in relation to raw in individual 3.

## 4 Discussion

Here, we have examined in which specific ways representative foods of Mediterranean and Western diets can alter gut microbial communities. The initial composition of the microbiota of the three individuals analyzed defined most of the compositional variability in our data after the food fermentations. Moreover, many taxon abundance changes were detected in a single individual, suggesting that they may be composition dependent. Dietary intervention studies have also suggested that the response of the gut microbiota presents high inter-individual variability, and that certain microbial taxa can be responsive or resistant to dietary changes (Salonen et al., 2014; Healey et al., 2017). Further studies with expanded numbers of individuals will be required to understand composition-dependent changes and the taxa that drive them. Nevertheless, we have detected several shared trends across individuals, which likely represent the more robust effects. These include compositional differences between all plant-based and animal-based food fermentations, as well as differences in comparisons at food category and individual food level. In the following paragraphs, we discuss the significant composition differences that occurred in all three individuals.

Animal and vegetable fats was the food category that produced the most changes in the gut microbiota, generally resulting in higher abundances of bacteria of the phylum Firmicutes and lower abundances of the genera *Bacteroides* and *Bifidobacterium*, in accordance with the results of Hildebrandt et al. (2009) for mice fed a high-fat diet. Although there are not many studies on the effects of dietary fatty acids on the microbiota *in vitro*, the ability of the microbiota to grow on fatty acids has been reported, as well as a large effect of these substrates on microbiota composition (Agans et al., 2018). Our results showed that the use of fats (especially butter) as substrates induced an increase in the abundance of potentially beneficial taxa such as *Faecalibacterium* (mainly *F. prausnitzii*), *Roseburia* (*R. inulinivorans*) and *Blautia*, as previously described in *in vivo* studies with a medium to low percentage of fatty acids in the diet (Haro et al., 2017). Although *F. prausnitzii* is considered one of the main butyrate producers in the human gut and has been attributed significant anti-inflammatory properties, evidence on the effect of diet on the modulation of this species is still conflicting. Nevertheless, monounsaturated fatty acids have been associated with its increase (Haro et al., 2017; Wan et al., 2019). *Blautia* and *Roseburia* are members of the Lachnospiraceae, a phylogenetically and morphologically heterogeneous family that contains many members described as having the ability of hydrolyzing carbohydrates to produce butyrate and other short chain fatty acids (SCFAs) (Ozato et al., 2019). *Blautia* has been negatively associated with visceral fat accumulation (Ozato et al., 2019) and decreases in diseases such as diabetes, Crohn’s disease or colorectal cancer (Chen et al., 2012; Murri et al., 2013). It has also been reported to increase in high-fat diets (Wan et al., 2019; Jo et al., 2021), in accordance with our results; however, there is no consensus, as other research suggests the opposite (Wang et al., 2020). In addition, animal and vegetable fats also increased the abundance of *Lachnospira*, a butyrate producer mainly reported to increase with high-fiber foods, although it has also been found to increase with unsaturated fats and to correlate with olive oil consumption in vegans and vegetarians (Jo et al., 2021).

Many studies have assessed the effects of high fat diets and even of saturated and unsaturated fats on the gut microbiota (reviewed in Yang et al., 2020). Broadly, saturated, and unsaturated fats have been reported to have opposite effects on gut microbiota modulation, with the latter producing an increase in the abundance of beneficial taxa such as *Akkermansia* and *Bifidobacterium*, and a decrease in potentially detrimental taxa such as *Streptococcus* and *Escherichia*. Our analyses also indicate some differences between saturated and unsaturated fats. Butter, which contains mainly saturated fatty acids, produced some of the greatest effects of all foods, consistently resulting in higher abundances of the Lachnospiraceae *Blautia*, *Roseburia*, *Lachnospira*, *Agathobacter*, *Fusicatenibacter*, and *Lachnoclostridium* in comparisons to a large variety of foods. In contrast, Lachnospiraceae genera were detected to increase in vegetable oils, which contain mainly unsaturated fatty acids, only in a much smaller range of comparisons. Moreover, *Blautia* did not increase in fermentations with either olive or sunflower oil, while *Roseburia*, *Lachnospira*, and *Fusicatenibacter* increased in some comparisons involving olive oil, but not with sunflower oil.

Numerous differences in the fermentative microbiota were also detected in comparisons involving dairy products or fish. Some previous studies have assayed the effects of fish oil on the gut microbiota, although without considering the contribution of the whole fish, detecting increases in Bacteroidetes, Lachnospiraceae and *Bifidobacterium*. In our fish fermentations, we mainly detected high abundances of *Lachnoclostridium* and *Ruminococcus*. These genera are known SCFA producers, but they have also been correlated with trimethylamine N-oxide (TMAO), which is generated in the liver from trimethylamine (TMA) produced by the gut microbiota from choline and carnitine (present in sources such as eggs, beef, pork and fish). In agreement, *Lachnoclostridium* and *Ruminococcus* have been associated with risk of atherosclerosis and cardiovascular disorders (De Filippis et al., 2016; Cai et al., 2022). In addition, *Lachnoclostridium* has been reported to increase in high-fat diets, and to be positively correlated with visceral fat through decreasing circulating acetate levels (Jo et al., 2021). On the other hand, *Ruminococcus* has been associated with various contrasting dietary patterns, including long-term fruit and vegetable consumption, low dietary fiber intake and omnivore diets (David et al., 2014; De Filippis et al., 2016).

Our results also revealed increases in the abundances of the genera *Lachnoclostridium* and *Ruminococcus* in fermentations of dairy products, along with increases of *Bifidobacterium*, *Agathobacter*, Erysipelotrichaceae UCG 003 and *Romboutsia*. In a systematic review, Aslam et al., 2020 reported a generalized increase with dairy consumption of *Bifidobacterium* and *Lactobacillus*, which are widely considered to be beneficial to the host, although they highlighted a lack of consensus due to the wide variety of dairy products with very different nutritional composition. On the other hand, members of the Erysipelotrichaceae have also been positively associated with dairy intake (Smith-Brown et al., 2016). However, elevated abundances of these organisms are likely detrimental since they are enriched in the intestines of obese humans and mice, have been associated with symptoms of the metabolic syndrome and promote obesity in gnotobiotic mice (Woting et al., 2014). The dairy food that produced the greatest variation in the microbiota in our experiments was gouda cheese, which has a high fatty acid content. This further supports a fundamental role for fatty acids in modifying the composition of the gut microbiota. Furthermore, choline and choline phospholipids, like phosphatidylcholine, are particularly enriched in high-fat dairy products such as gouda and butter, which likely contributed to the significant effect of these two foods on *Lachnoclostridium* abundances.

It is remarkable that most elevated taxon abundances in the microbiota were detected in fermentations with animal and vegetable fats, dairy products, and fish. Overall, fermentations of these foods often produced higher abundances of taxa of the Lachnospiraceae family, with *Lachnoclostridium* standing out in the three food categories. In contrast, significant differences involving fruits, vegetables and grain-based products mostly represented reduced abundances of genera in comparison to these animal-based food categories. This may suggest that the short fermentation processes used in our experiments were not sufficient to favor increases of specific genera when carbohydrate-rich foods were employed. As most intestinal bacteria are mainly adapted to growth in the presence of the complex polysaccharides and fibers that reach the colon, many taxa present in the inoculum must have been able to take advantage of these substrates. Therefore, a short fermentation may not have provided enough time to select for taxa having relatively small growth advantages with respect to other carbohydrate fermenters. Longer fermentation times may thus be needed to amplify growth rate differences among taxa with specific carbohydrate-rich foods. In contrast, the ability to grow on proteins and, especially, fats is likely more unevenly distributed across gut bacteria, so that short fermentations with foods rich in these substrates may more readily select for subsets of specialized bacteria, resulting in significant increases in their abundances. In this respect, it is noteworthy that *Lachnoclostridium* is a known protein degrader and is the most abundant gut bacterium capable of metabolizing the choline obtained from high-fat foods (Cai et al., 2022). David et al., 2014 also reported that animal-based diets had a greater impact on gut microbiota composition, particularly an increase in the abundance of bile-tolerant microorganisms, presumably due to increased bile acid secretion as a result of the high fat intake linked to such diets. However, in our *in vitro* experiments there is no regulation of bile acid level depending on the food, suggesting that the effects of animal-based products on gut microbiota composition are also influenced by other factors. In accordance, the taxa favored by animal-based diets *in vivo* in the work by David et al. (2014) are the bile-resistant *Bilophila*, *Bacteroides*, and *Alistipes*, which were not increased by fatty foods in our experiments.

Regarding cooking methods, it is well established that they affect food digestibility and nutrient bioaccessibility (Chen et al., 2020; Navajas-Porras et al., 2020), and they may impact extensively on gut microbiota structure and functionality (Pérez-Burillo et al., 2018a). On one hand, cooking methods that apply high temperatures, such as frying or grilling, favor cellular break-down and release of chemicals into the environment (Miglio et al., 2007; Nissen et al., 2022; Cattivelli et al., 2023). This may favor bacterial growth by facilitating the uptake of bioactive compounds. However, released phytochemicals may be harmful for some bacteria (Poirier et al., 2016), while others may have the enzymatic equipment to metabolize such phytochemicals and grow on them (Rowland et al., 2018). On the other hand, cooking favors specific chemical reactions, such as chemical browning in protein and sugar-rich foods. Chemical browning involves a plethora of chain reactions that yield numerous compounds, such as furanic compounds, that have inhibitory effects on some bacteria (Monlau et al., 2014). Chemical browning can also yield melanoidins, known to have a fiber-like behavior and therefore promote the growth of some beneficial species (Rajakaruna et al., 2022). Accordingly, cooking may have a complex effect on gut microbial communities.

However, to date, few studies have been conducted on the effect of cooking methods on the composition of the gut microbiota (Shen et al., 2010; Pérez-Burillo et al., 2018a; Chen et al., 2020; Maldonado-Mateus et al., 2021). Although these studies have collectively evaluated the effect of different cooking processes on meats, legumes, vegetables, cereals, and fruits, none has addressed this issue at a large scale by analyzing a wide range of foods within the same study. Here, we have examined separately the effects of cooking methods on a variety of different food types.

In our experiments, frying resulted in a lower abundance of Ruminococcaceae UBA1819 in comparison with roasted foods for all food categories considered together and in all individuals. This taxon is a butyrate producer known to increase with consumption of soluble fibers and to correlate negatively with subcutaneous and epidermal fat, cholesterol and weight gain (Hu et al., 2022), suggesting that its decrease with fried foods is undesirable. This result is particularly remarkable in that one could expect the effects of cooking methods to be masked by those of food composition, suggesting that frying and roasting have strong effects across food categories. Previous work in our group identified frying as the cooking method that produced the highest amounts of furosine and furfural (used as indicators of the Maillard reaction) and found it to be correlated with the abundance of different taxa depending on the food (Pérez-Burillo et al., 2018a).

Our results also highlighted several trends when comparing cooking methods within the different food categories. However, these results were not detected across individuals, indicating that they are likely dependent on the composition of the microbiota and suggesting that cooking method effects may be highly personalized. Frying resulted in significant taxon abundance differences with other methods within various food categories, mainly fruit, vegetables and bread, resulting mostly in higher and lower abundances of genera within the Lachnospiraceae and Ruminococcaceae families, respectively, including lower abundances of the aforementioned Ruminococcaceae UBA1819. Similar results were also obtained for grilling, a method that resembles frying in reaching elevated temperatures and employing oil. These high temperature treatments have strong effects on the fibers and other complex carbohydrates present in plant-based foods, on which members of both the Lachnospiraceae and Ruminococcaceae are specialized to grow (Biddle et al., 2013), but such effects might facilitate the utilization of these substrates for some bacteria while hindering it for others. Olive oil retained in grilled and fried foods may also have contributed to Lachnospiraceae increases, as genera of this family were detected at high abundances in olive oil fermentations. However, grilling and frying did not result in Lachnospiraceae increases in meat. Another relevant observation regarding the effect of frying on meat is that *Lactococcus*, particularly *L. piscium*, known to be one of the predominant taxa within spoilage microbial communities in cold-stored meat products, was detected at lower abundance in fried meat, suggesting that frying may contribute to reduce the presence of these bacteria.

Boiling was another cooking method that yielded significant differences against other methods in various food categories, mainly fish and meat. Generally, we detected a lower abundance of many Firmicutes genera when comparing boiled foods with other methods. This result may be due to several complementary causes. On one hand, the loss of hydrosoluble nutrients such as vitamins and minerals may limit the growth of some bacteria. On the other, the lower temperature reached during boiling will result both in lower levels of cellular breakdown and in a paucity of Maillard reaction products, which would otherwise be abundant when protein-rich foods such as fish and meat are prepared at higher temperatures. The lack of Maillard reaction products such as melanoidins, which are fiber-like structures that can be metabolized by some bacteria, along with the lower levels of cellular breakdown, may have reduced the substrates available for the growth of Firmicutes genera in boiled fish and meat.

In conclusion, the *in vitro* approach followed in this work has revealed the short-term effects of specific foods on the gut microbiota of different individuals. The experimental design employed has certain limitations, such as the fact that we can only observe changes that occur rapidly due to the relatively short fermentation times. In particular, this limitation may have affected our ability to detect taxa with small growth advantages on specific plant-based foods. Longer fermentation times or dynamic *in vitro* digestion-fermentation systems may be required to amplify small growth rate differences among taxa, but such experiments would have to be limited to a restricted number of food/cooking method combinations due to their more involved and time-consuming nature. Also, our set up reflects mainly the punctual effects of drastically limiting the substrates available to the microbiota to those present in a single food, which may not be representative of what occurs when a food is consumed as part of a complex overall diet. Although effects on overall parameters such as alpha diversity may indeed mostly reflect this reduced availability of substrates, the fermentation experiments do reveal those bacterial taxa that are most responsive to a given food. Many of the detected effects of specific foods and, especially, cooking methods, have been particular to a single individual, but shared trends across all individuals have also been manifested. Further research that takes into account individual variation across a larger number of subjects will be required to unravel the dependency of food and cooking effects on the initial composition of the gut microbiota. This knowledge will pave the way to a personalized modulation of the microbiota for precision nutrition.

## Data availability statement

The datasets presented in this study can be found in online repositories. The names of the repository/repositories and accession number(s) can be found below: https://www.ebi.ac.uk/ena, PRJEB51719.

## Ethics statement

The studies involving humans were approved by the Ethics Committee of the University of Granada. The studies were conducted in accordance with the local legislation and institutional requirements. The participants provided their written informed consent to participate in this study.

## Author contributions

AL-A: Data curation, Formal analysis, Investigation, Methodology, Visualization, Writing – original draft, Writing – review & editing. SP-B: Investigation, Methodology, Writing – review & editing. BN-P: Investigation, Writing – review & editing. EL: Writing – review & editing. SR-P: Investigation, Methodology, Writing – review & editing. SP: Investigation, Writing – review & editing. NJ-H: Investigation, Methodology, Writing – review & editing. B-MC: Investigation, Writing – review & editing. JR-H: Conceptualization, Funding acquisition, Methodology, Project administration, Resources, Supervision, Writing – review & editing. MG: Conceptualization, Methodology, Resources, Supervision, Writing – original draft, Writing – review & editing. MF: Conceptualization, Methodology, Project administration, Resources, Supervision, Writing – original draft, Writing – review & editing.

## Supplementary material

Supplementary Figure 1 Heatmap of relative abundance differences at genus level between food categories. Y-axis food categories have higher (orange), or lower (green) abundance of a given genus against X-axis food categories. The ANCOM method was used for comparisons with the Benjamini–Hochberg procedure for false discovery rate control. Significant comparisons (*q* < 0.05) in all the individuals are represented (PDF).

Supplementary Figure 2 Heatmap of relative abundance differences at species level between food categories. Y-axis food categories have higher (orange), or lower (green) abundance of a given species against X-axis food categories. The ANCOM method was used for comparisons with the Benjamini–Hochberg procedure for false discovery rate control. Significant comparisons (*q* < 0.05) in all the individuals are represented (PDF).

Supplementary Figure 3 Heatmap of relative abundance differences at genus level between foods. Y-axis foods have higher (orange), or lower (green) abundance of a given genus against X-axis foods. The ANCOM method was used for comparisons with the Benjamini–Hochberg procedure for false discovery rate control. Significant comparisons (*q* < 0.05) in all the individuals are represented (PDF).

Supplementary Figure 4 Heatmap of relative abundance differences at species level between foods. Y-axis foods have higher (orange) or lower (green) abundance of a given species against X-axis foods. The ANCOM method was used for comparisons with the Benjamini–Hochberg procedure for false discovery rate control. Significant comparisons (*q* < 0.05) in all the individuals are represented (PDF).

Supplementary Figure 5 Fold-changes in relative abundance of bacterial genera between cooking methods per food category. Fold-changes are represented as log2(FC). Significant comparisons (*q* < 0.05) according to ANCOM for each individual are represented. Cooking methods are represented in different colors and the individuals in which a given comparison is significant are indicated on the bars (PDF).

Supplementary Figure 6 Fold-changes in relative abundance of bacterial species between cooking methods per food category. Fold-changes are represented as log2(FC). Significant comparisons (*q* < 0.05) according to ANCOM for each individual are represented. Cooking methods are shown in different colors and the individuals in which a given comparison is significant are indicated (PDF).
