# Supplementary material for: Effects of different foods and cooking methods on the gut microbiota: an in vitro approach
Source: Front Microbiol. 2024 Jan 8;14:1334623. doi: 10.3389/fmicb.2023.1334623 (PMC10800916; doi:10.3389/fmicb.2023.1334623)
Supplement: Supplementary Figure 1 — Heatmap of relative abundance differences at genus level between food categories. Y-axis food categories have higher (orange), or lower (green) abundance of a given genus against X-axis food categories. The ANCOM method was used for comparisons with the Benjamini–Hochberg procedure for false discovery rate control. Significant comparisons (q < 0.05) in all the individuals are represented (PDF). [file Data_Sheet_1.PDF]

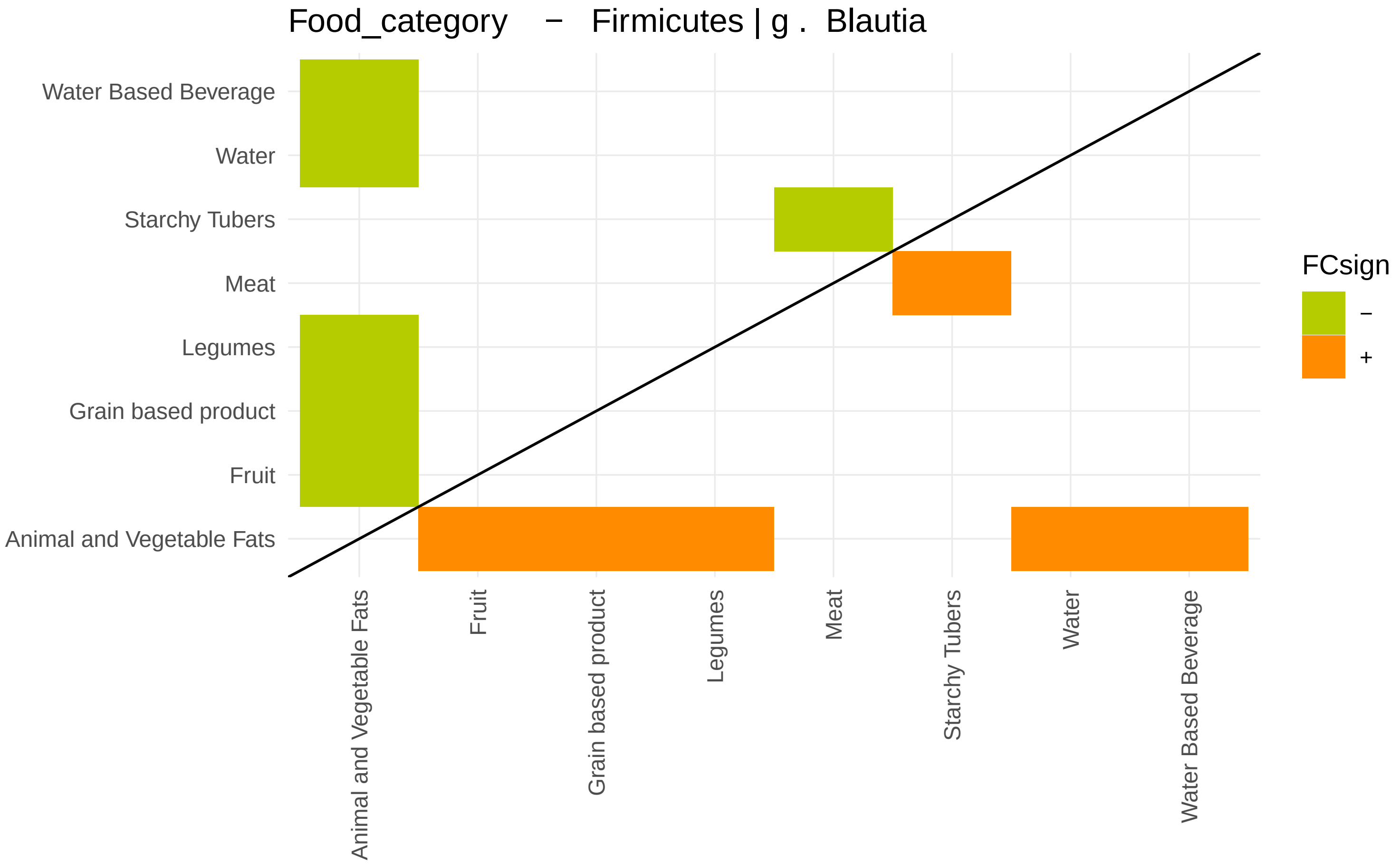
Food_category − Firmicutes | g . Blautia
Water Based Beverage
Water
Starchy Tubers
Meat
Legumes
Grain based product
Fruit
Animal and Vegetable Fats
FCsign
−
+

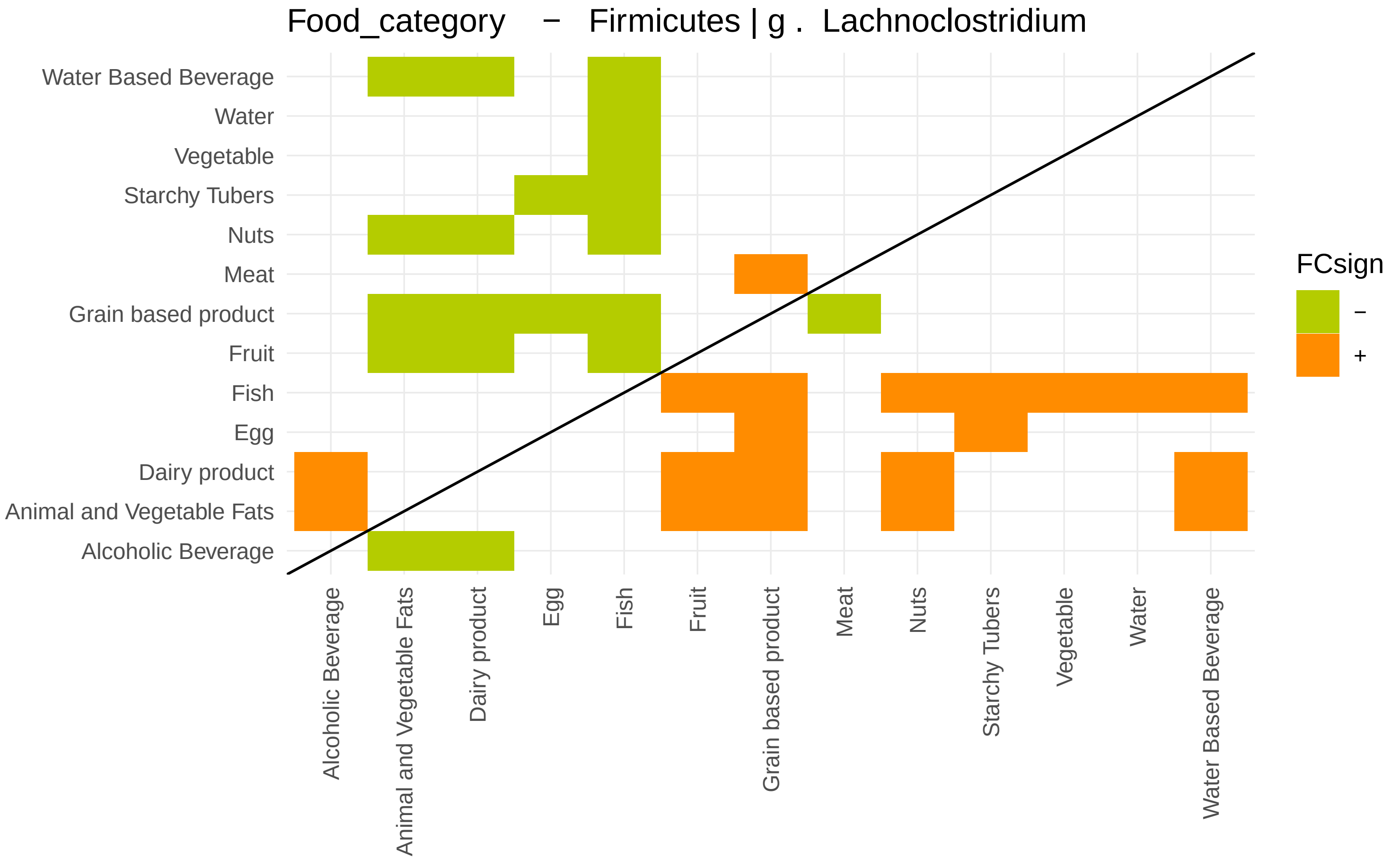

Food_category – Firmicutes | g . Lachnoclostridium
Water Based Beverage
Water
Vegetable
Starchy Tubers
Nuts
Meat
Grain based product
Fruit
Fish
Egg
Dairy product
Animal and Vegetable Fats
Alcoholic Beverage
FCsign
–
+

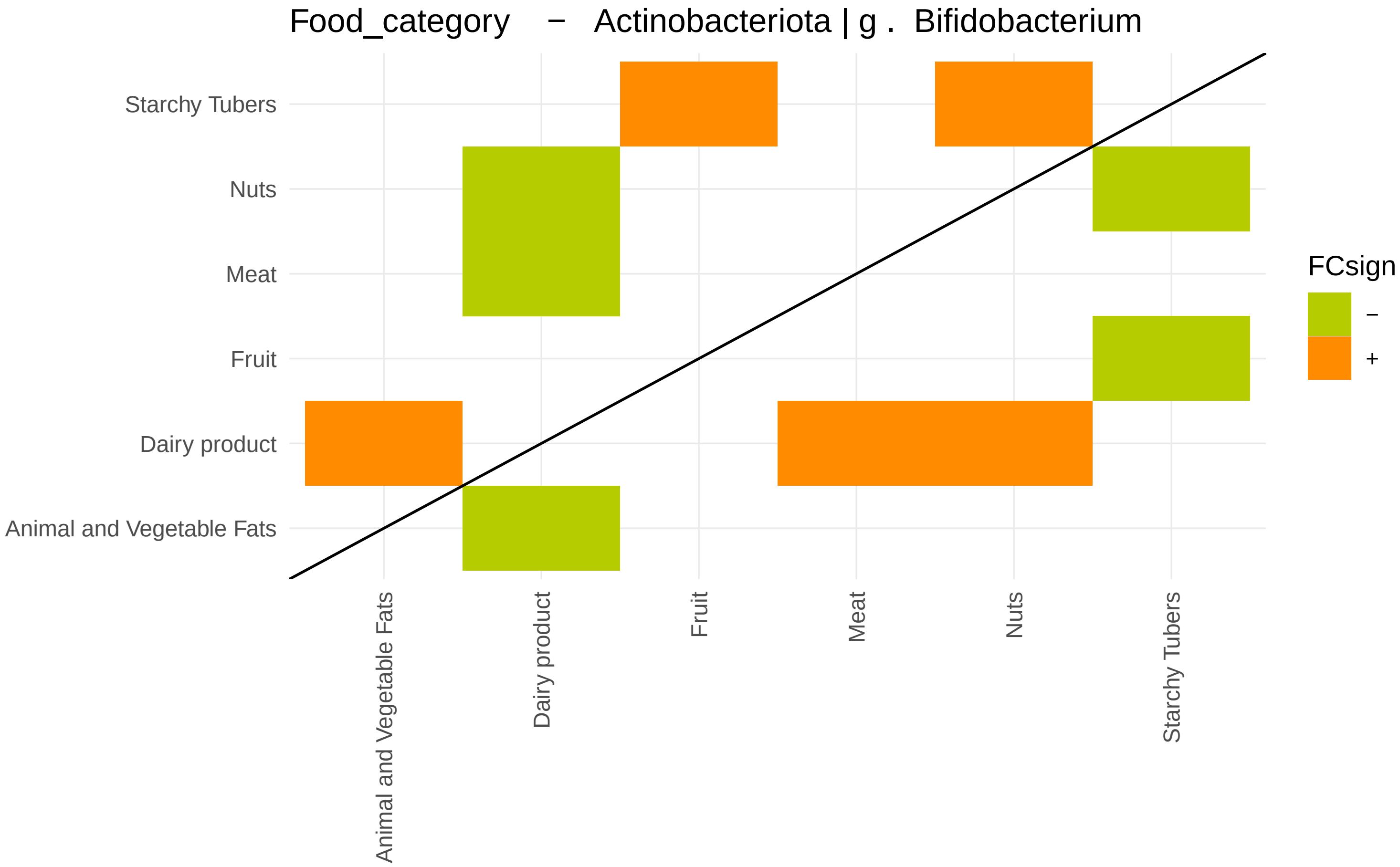
Food_category − Actinobacteriota | g . Bifidobacterium
Starchy Tubers
Nuts
Meat
Fruit
Dairy product
Animal and Vegetable Fats
Animal and Vegetable Fats
Dairy product
Fruit
Meat
Nuts
Starchy Tubers
FCsign
−
+

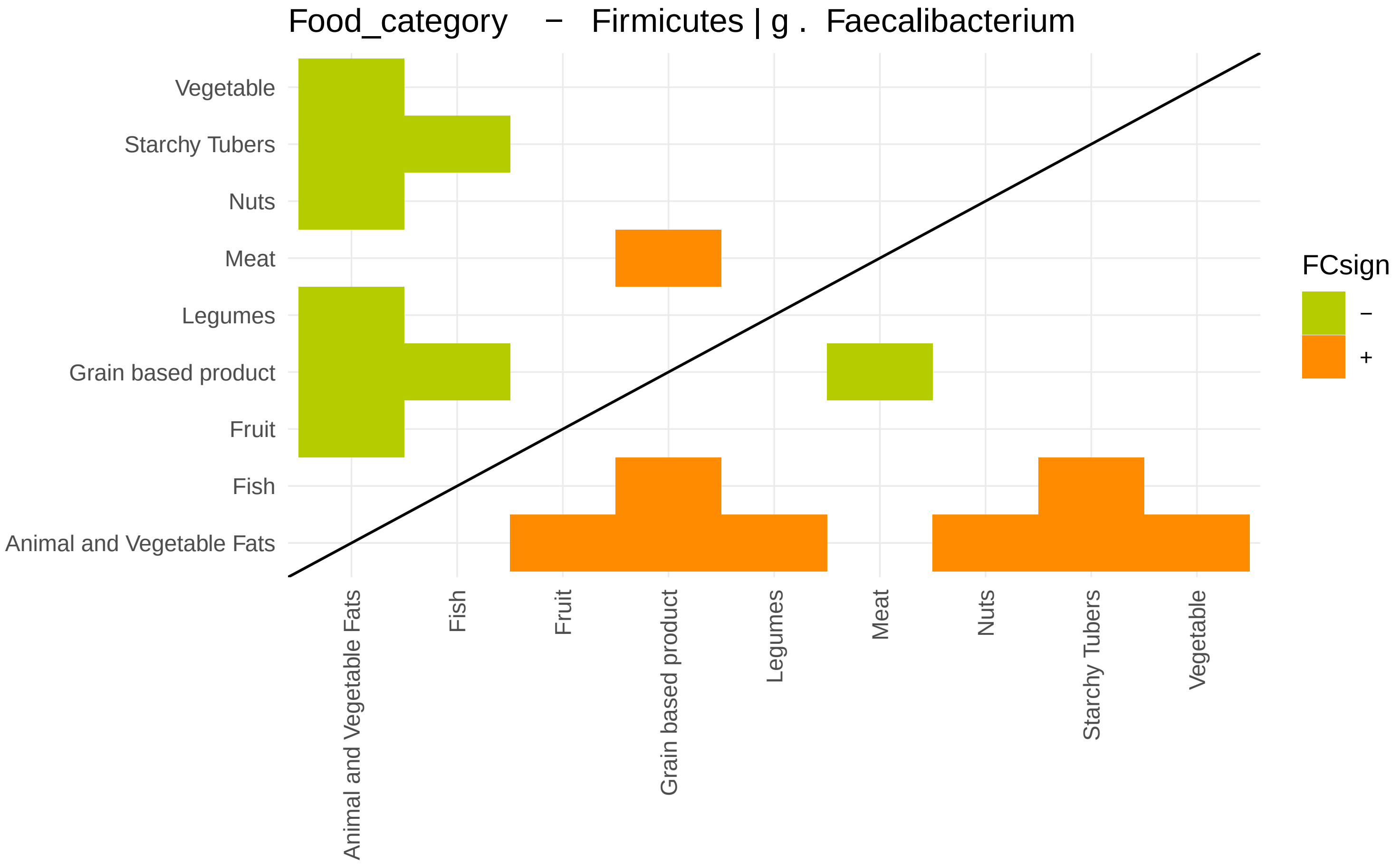

Food_category − Firmicutes | g . Faecalibacterium
Vegetable
Starchy Tubers
Nuts
Meat
Legumes
Grain based product
Fruit
Fish
Animal and Vegetable Fats
FCsign
−
+
Animal and Vegetable Fats
Fish
Fruit
Grain based product
Legumes
Meat
Nuts
Starchy Tubers
Vegetable

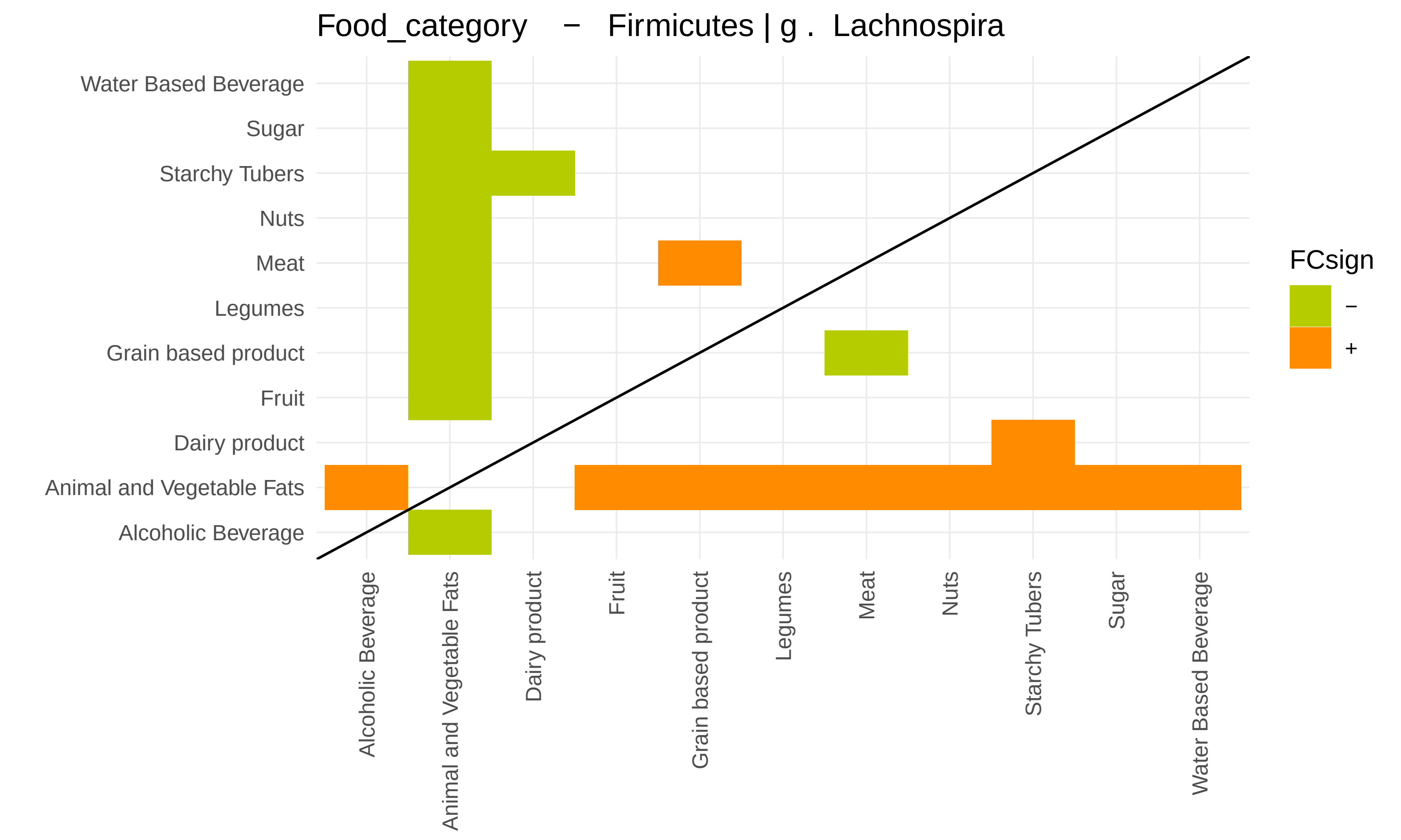

Food_category − Firmicutes | g . Lachnospira
Water Based Beverage
Sugar
Starchy Tubers
Nuts
Meat
Legumes
Grain based product
Fruit
Dairy product
Animal and Vegetable Fats
Alcoholic Beverage
FCsign
−
+

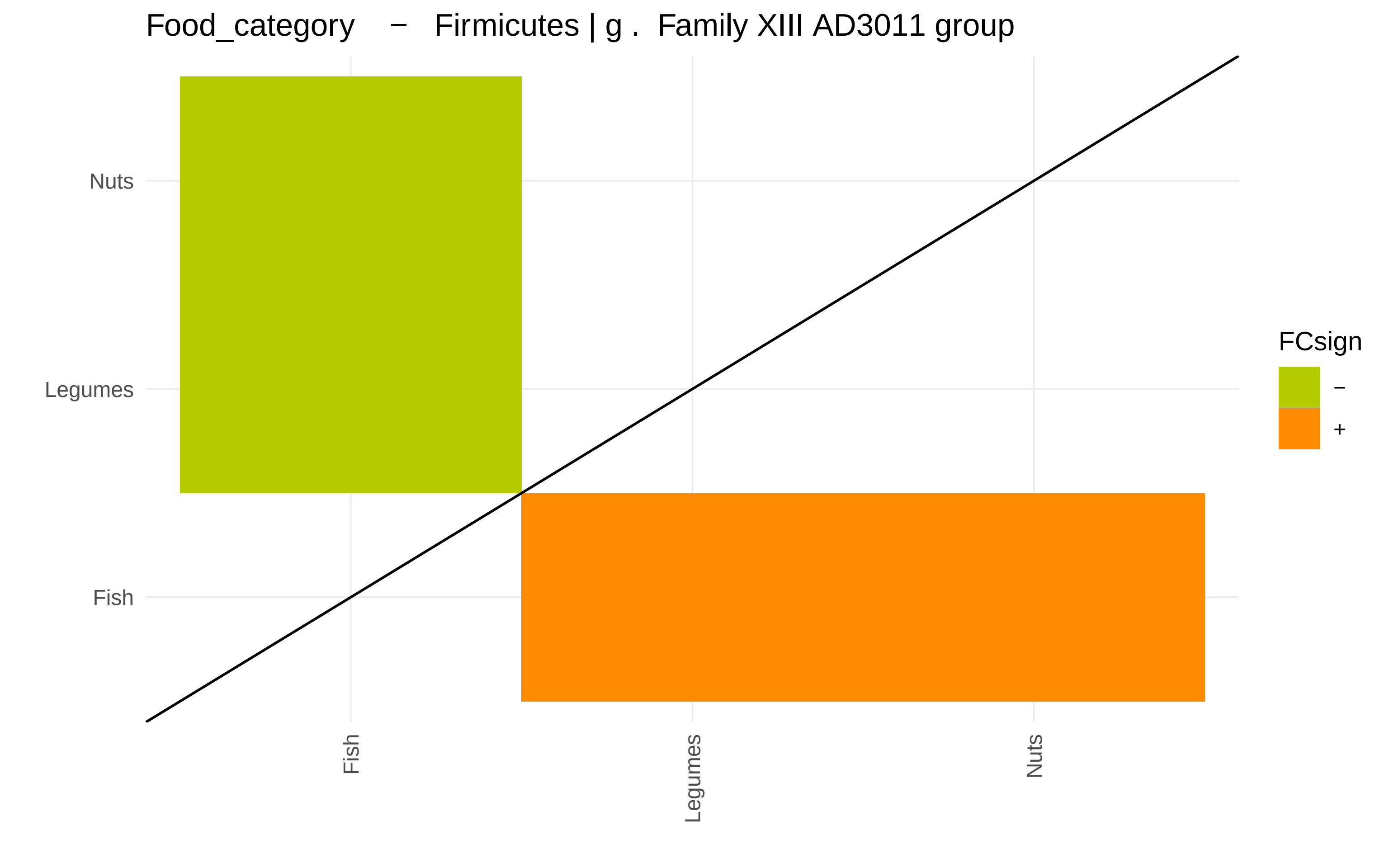
Food_category − Firmicutes | g . Family XIII AD3011 group
Nuts
Legumes
Fish
Fish
Legumes
Nuts
FCsign
−
+

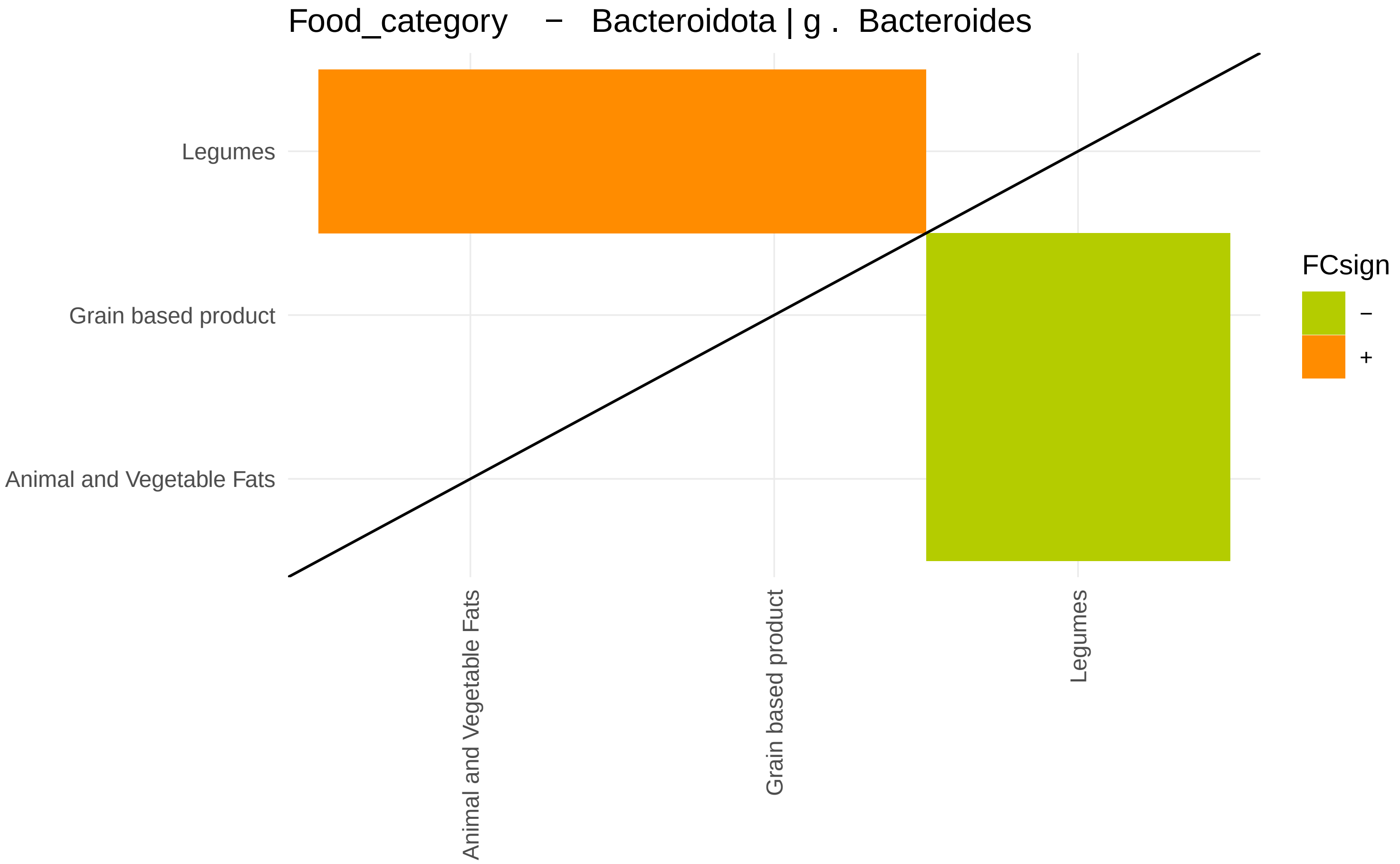

Food_category − Bacteroidota | g . Bacteroides
Legumes
Grain based product
Animal and Vegetable Fats
Animal and Vegetable Fats
Grain based product
Legumes
FCsign
−
+

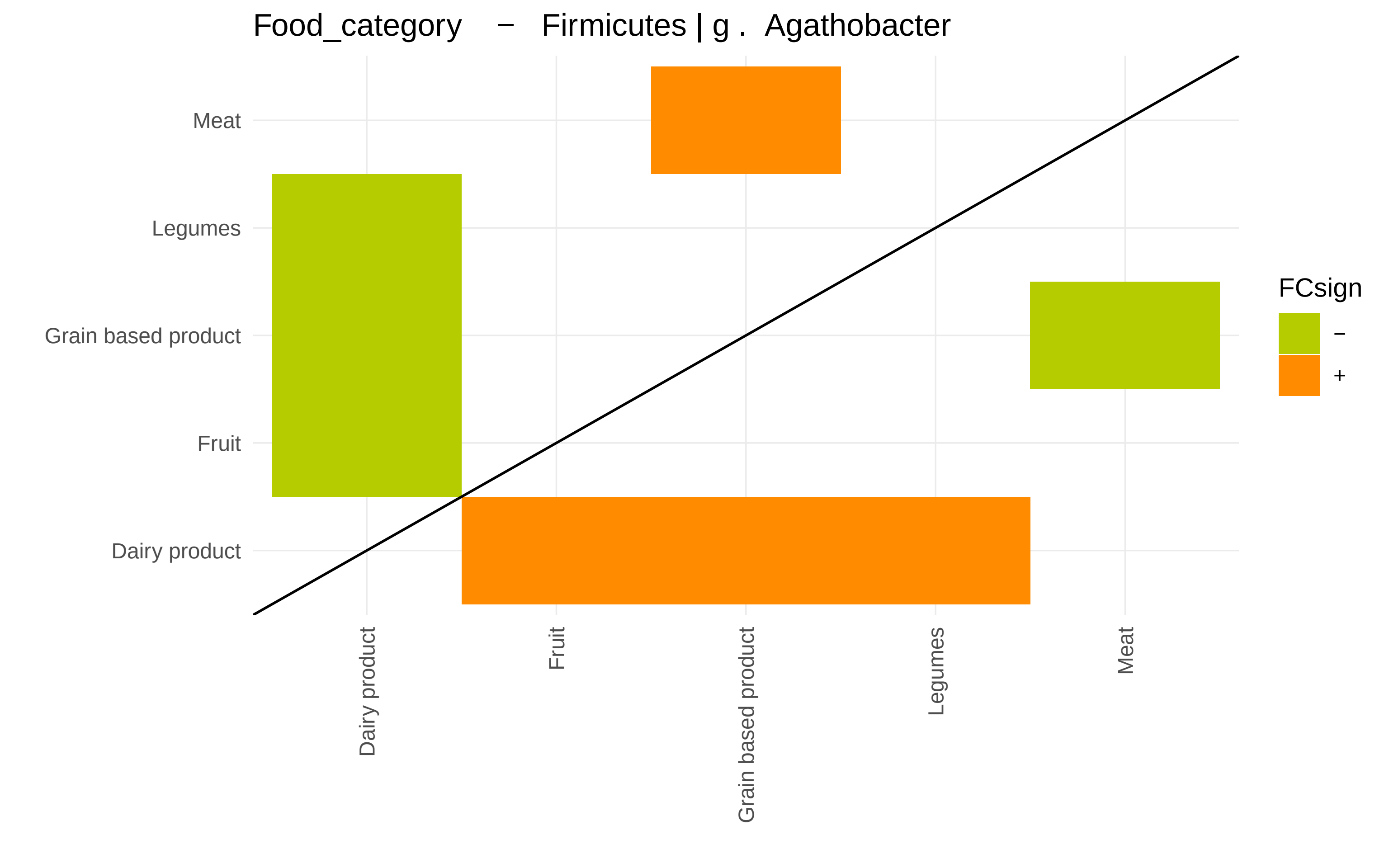
Food_category − Firmicutes | g . Agathobacter
Meat
Legumes
Grain based product
Fruit
Dairy product
Dairy product
Fruit
Grain based product
Legumes
Meat
FCsign
−
+

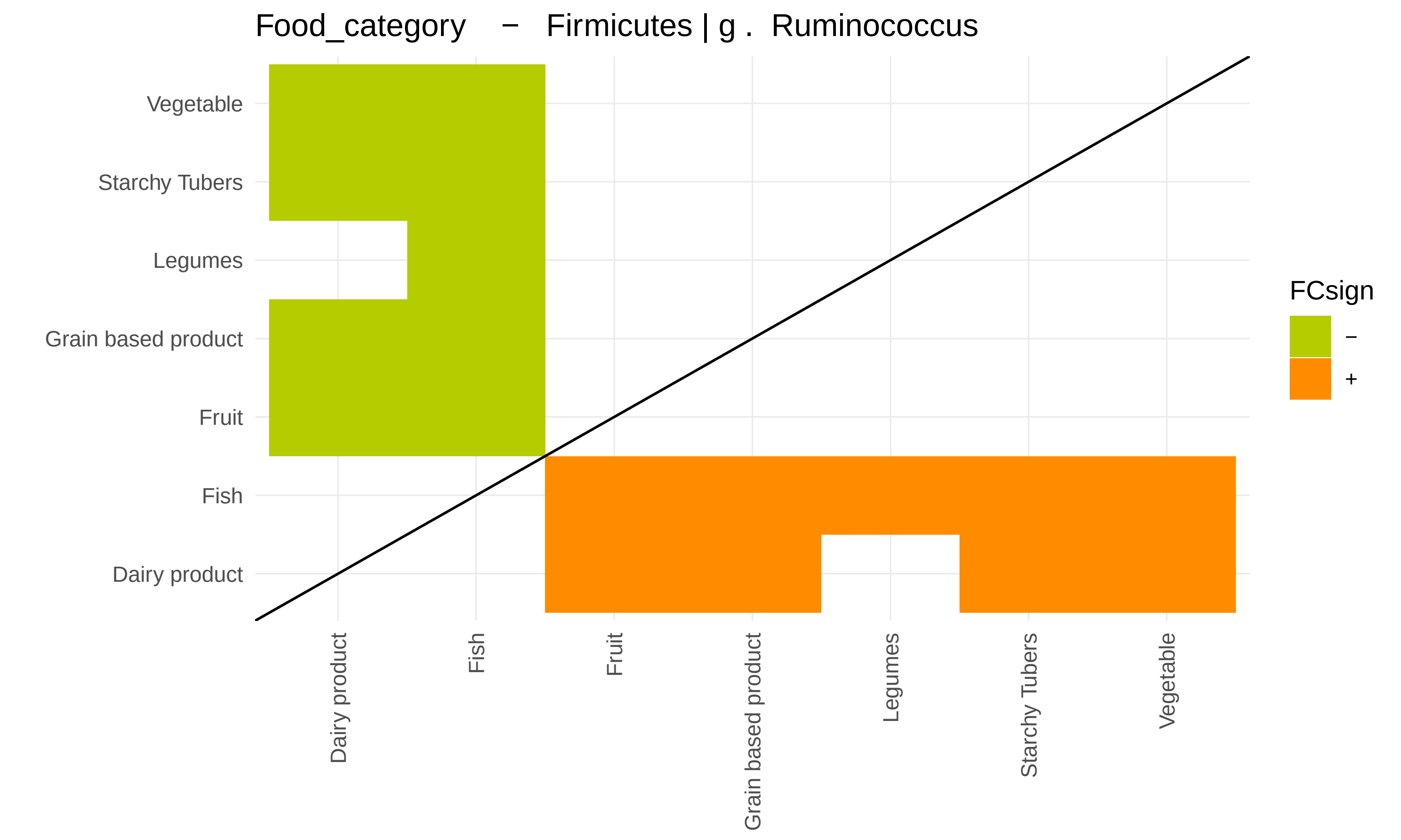

Food_category − Firmicutes | g . Ruminococcus
Vegetable
Starchy Tubers
Legumes
Grain based product
Fruit
Fish
Dairy product
Dairy product
Fish
Fruit
Grain based product
Legumes
Starchy Tubers
Vegetable
FCsign
−
+

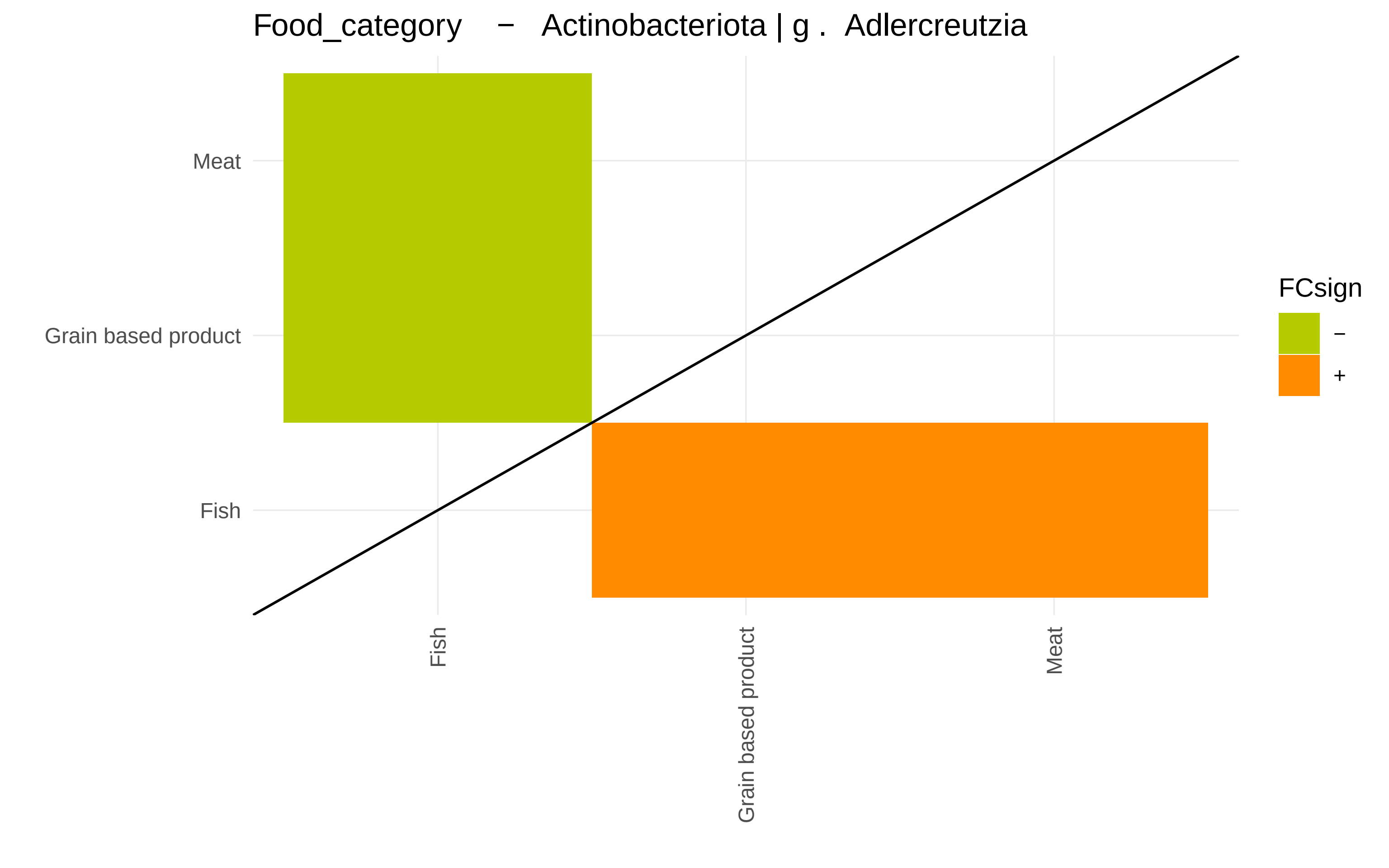

Food_category − Actinobacteriota | g . Adlercreutzia
Meat
Grain based product
Fish
Fish
Grain based product
Meat
FCsign
−
+

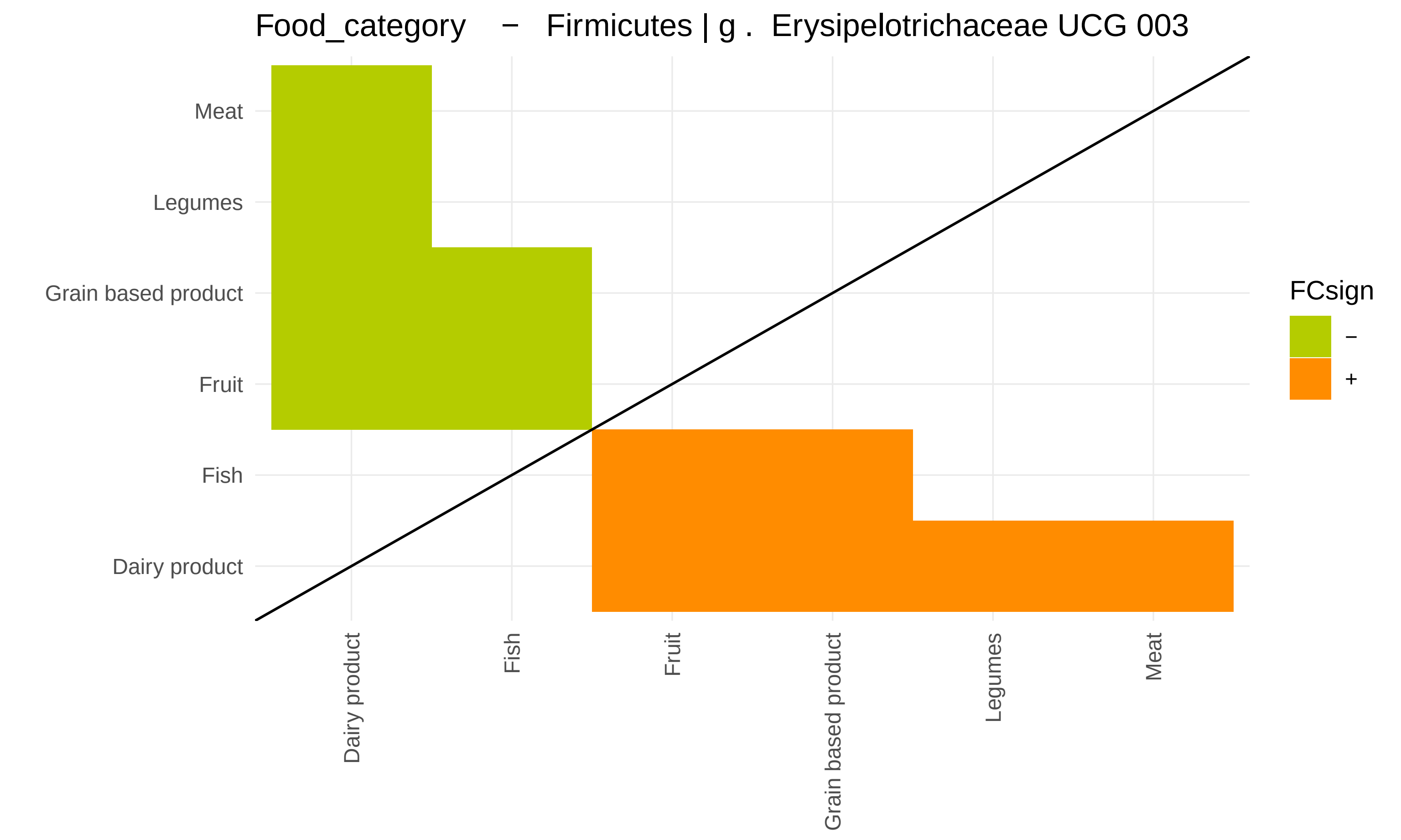

Food_category − Firmicutes | g . Erysipelotrichaceae UCG 003
Meat
Legumes
Grain based product
Fruit
Fish
Dairy product
Dairy product
Fish
Fruit
Grain based product
Legumes
Meat
FCsign
−
+

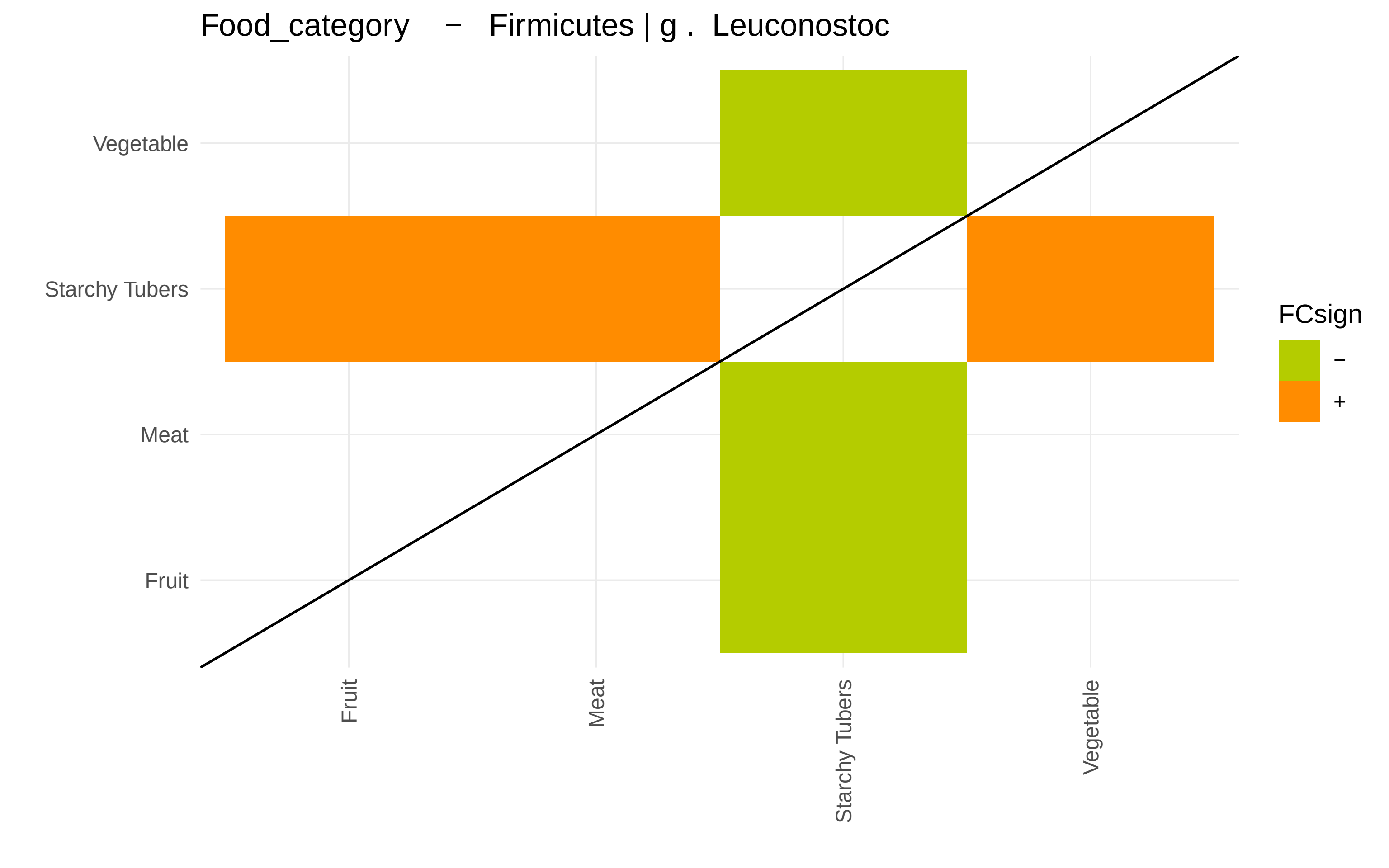

Food_category − Firmicutes | g . Leuconostoc
Vegetable
Starchy Tubers
Meat
Fruit
Fruit
Meat
Starchy Tubers
Vegetable
FCsign
−
+

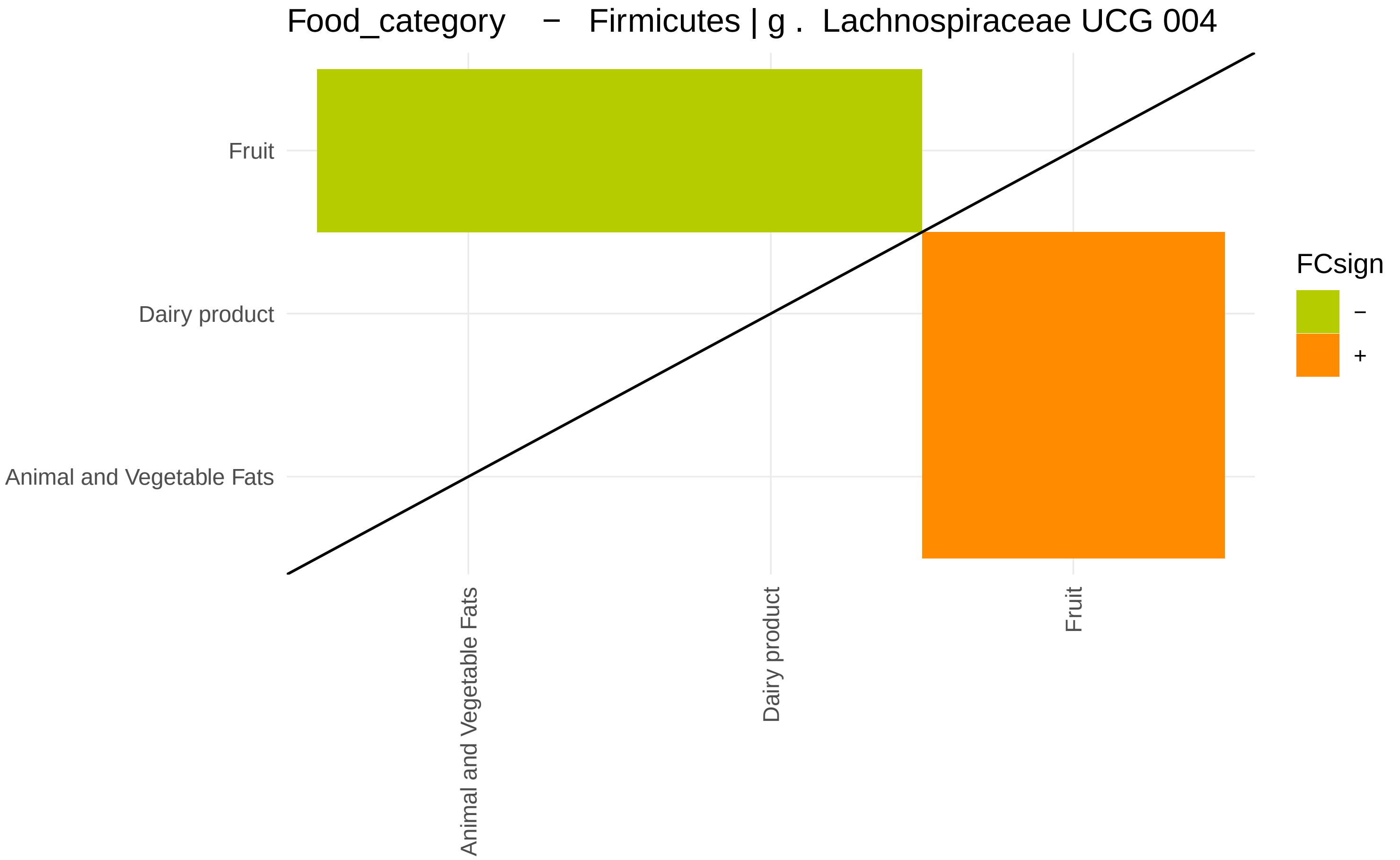

Food_category − Firmicutes | g . Lachnospiraceae UCG 004
Fruit
Dairy product
Animal and Vegetable Fats
Animal and Vegetable Fats
Dairy product
Fruit
FCsign
−
+

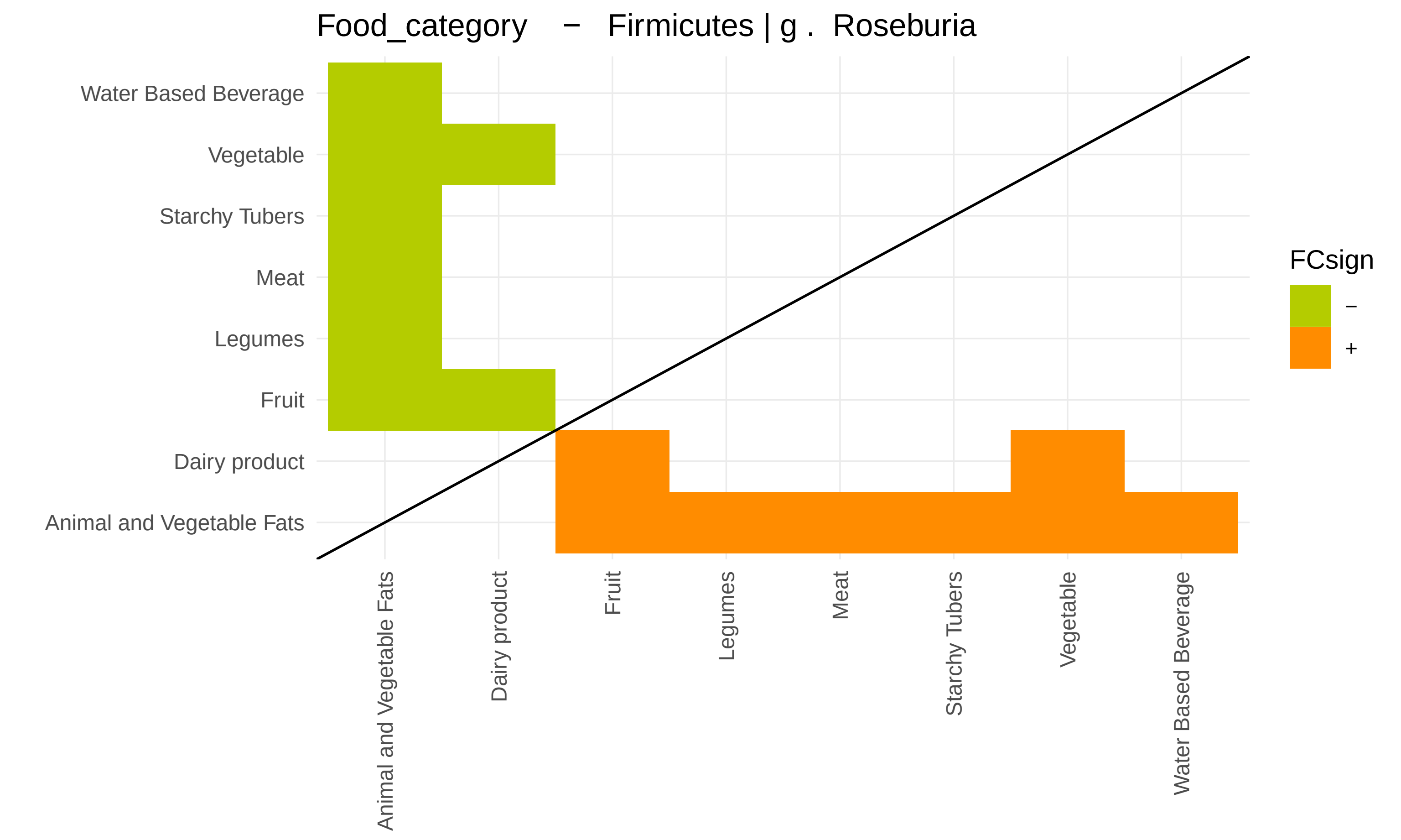

Food_category − Firmicutes | g . Roseburia
Water Based Beverage
Vegetable
Starchy Tubers
Meat
Legumes
Fruit
Dairy product
Animal and Vegetable Fats
Animal and Vegetable Fats
Dairy product
Fruit
Legumes
Meat
Starchy Tubers
Vegetable
Water Based Beverage
FCsign
−
+

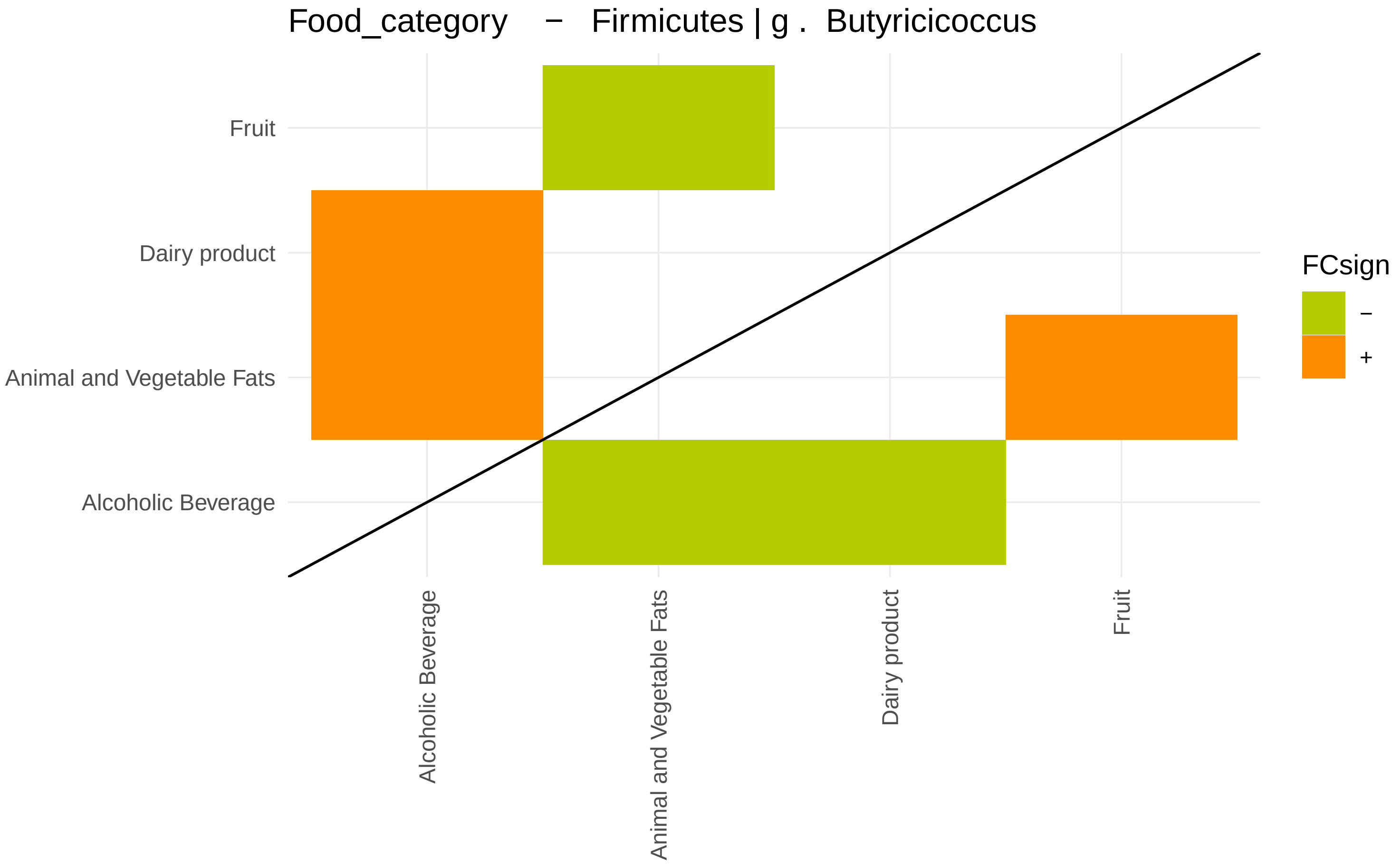
Food_category − Firmicutes | g . Butyricicoccus
Fruit
Dairy product
Animal and Vegetable Fats
Alcoholic Beverage
Alcoholic Beverage
Animal and Vegetable Fats
Dairy product
Fruit
FCsign
−
+

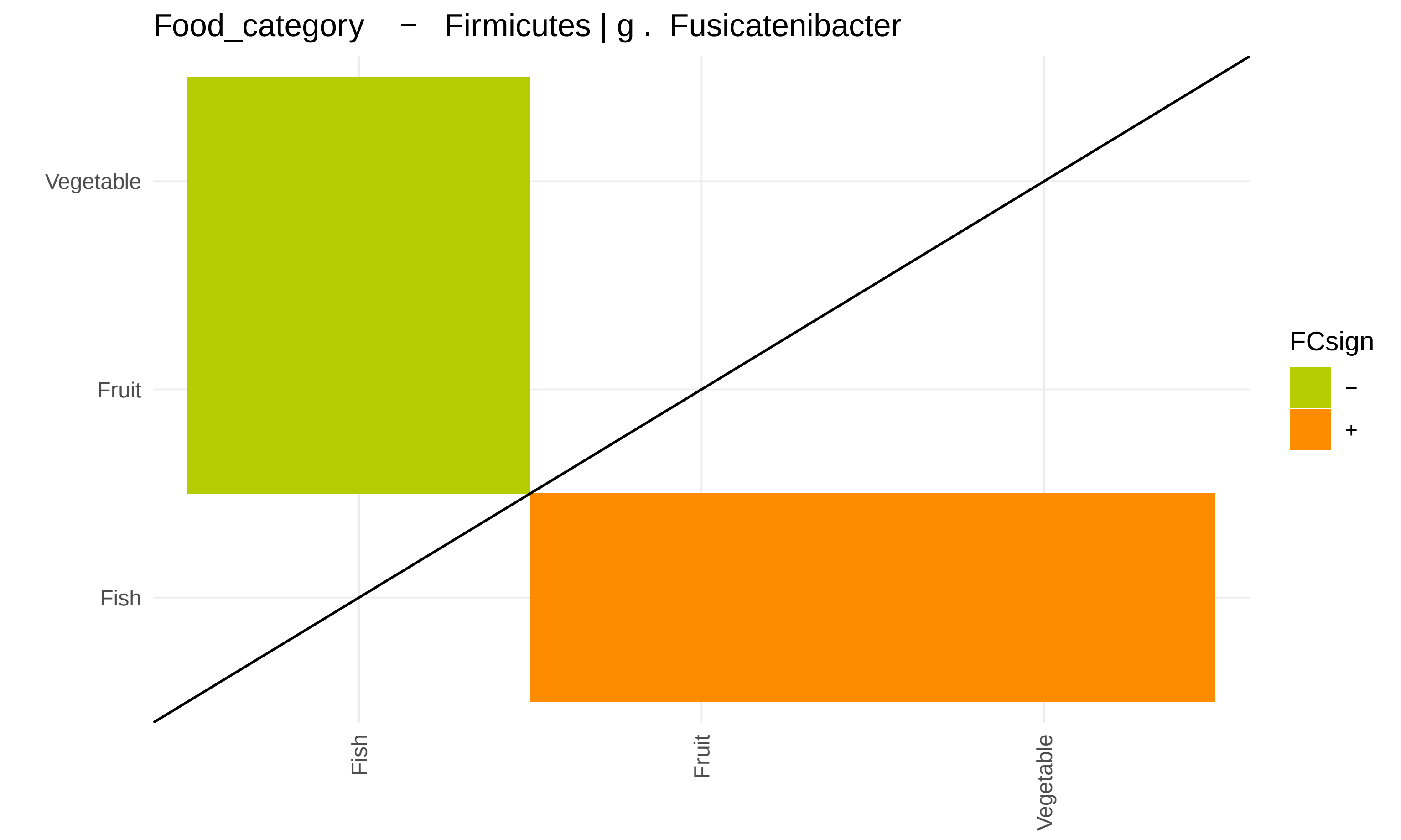

Food_category − Firmicutes | g . Fusicatenibacter
Vegetable
Fruit
Fish
Fish
Fruit
Vegetable
FCsign
−
+

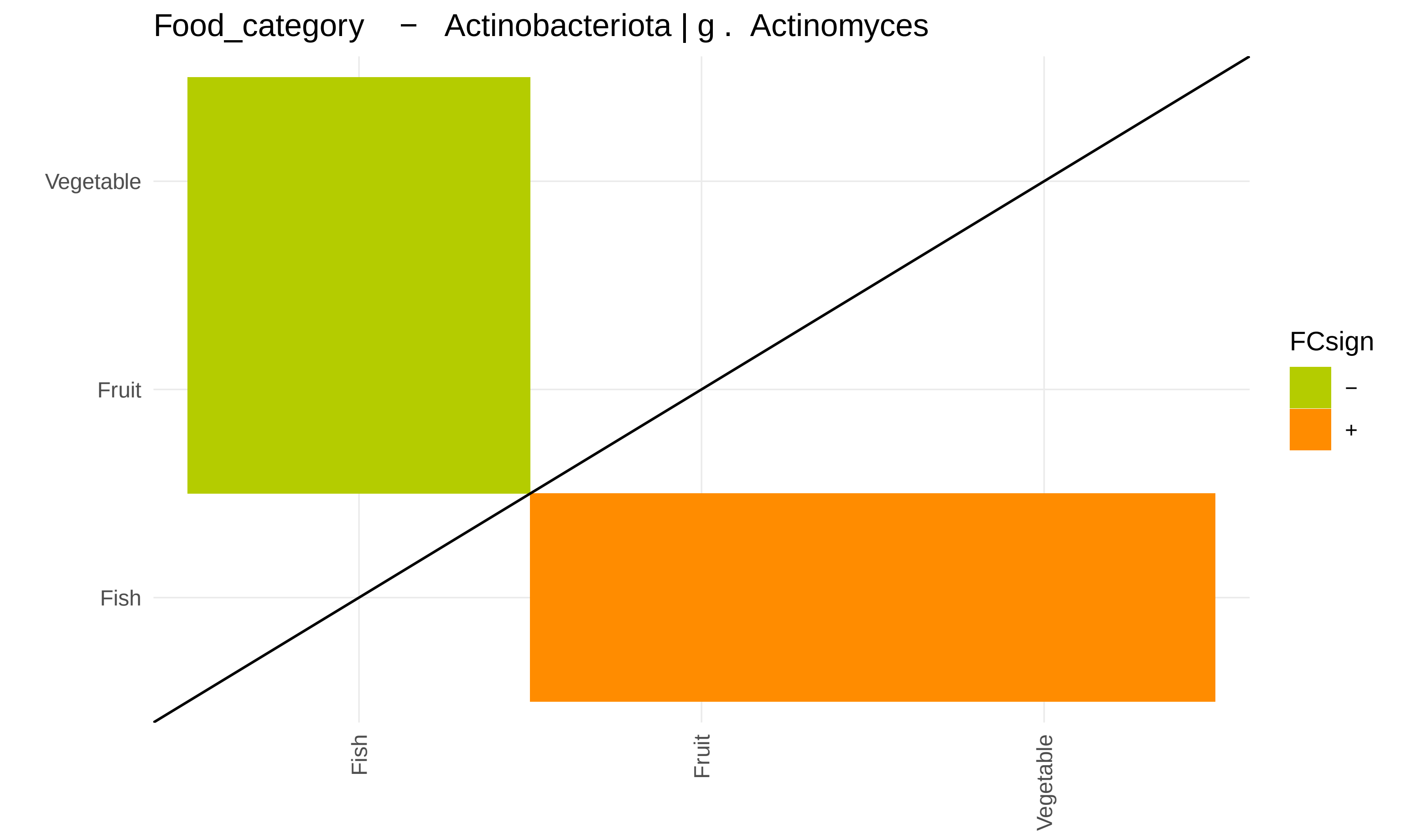

Food_category − Actinobacteriota | g . Actinomyces
Vegetable
Fruit
Fish
Fish
Fruit
Vegetable
FCsign
−
+

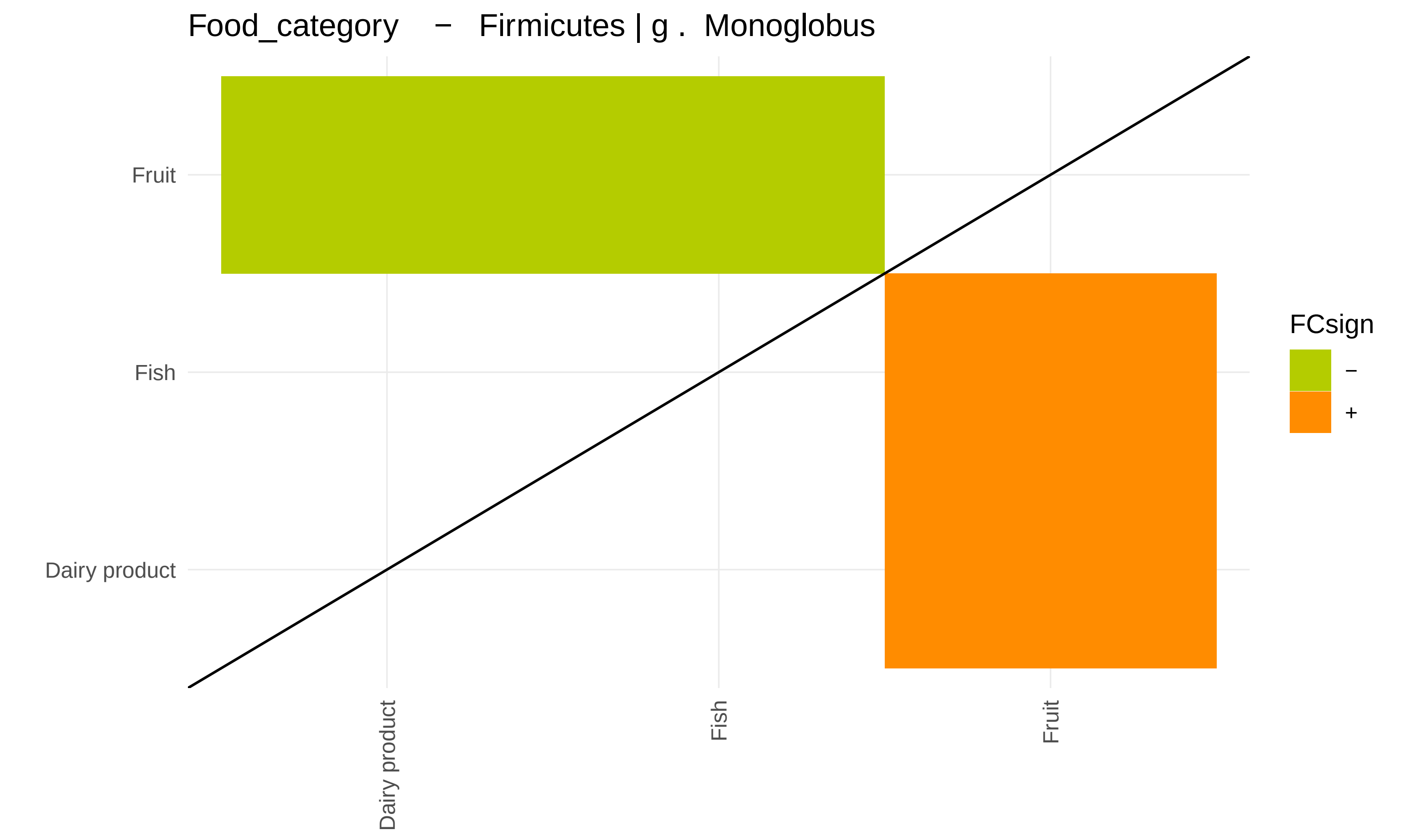
Food_category − Firmicutes | g . Monoglobus
Fruit
Fish
Dairy product
Dairy product
Fish
Fruit
FCsign
−
+

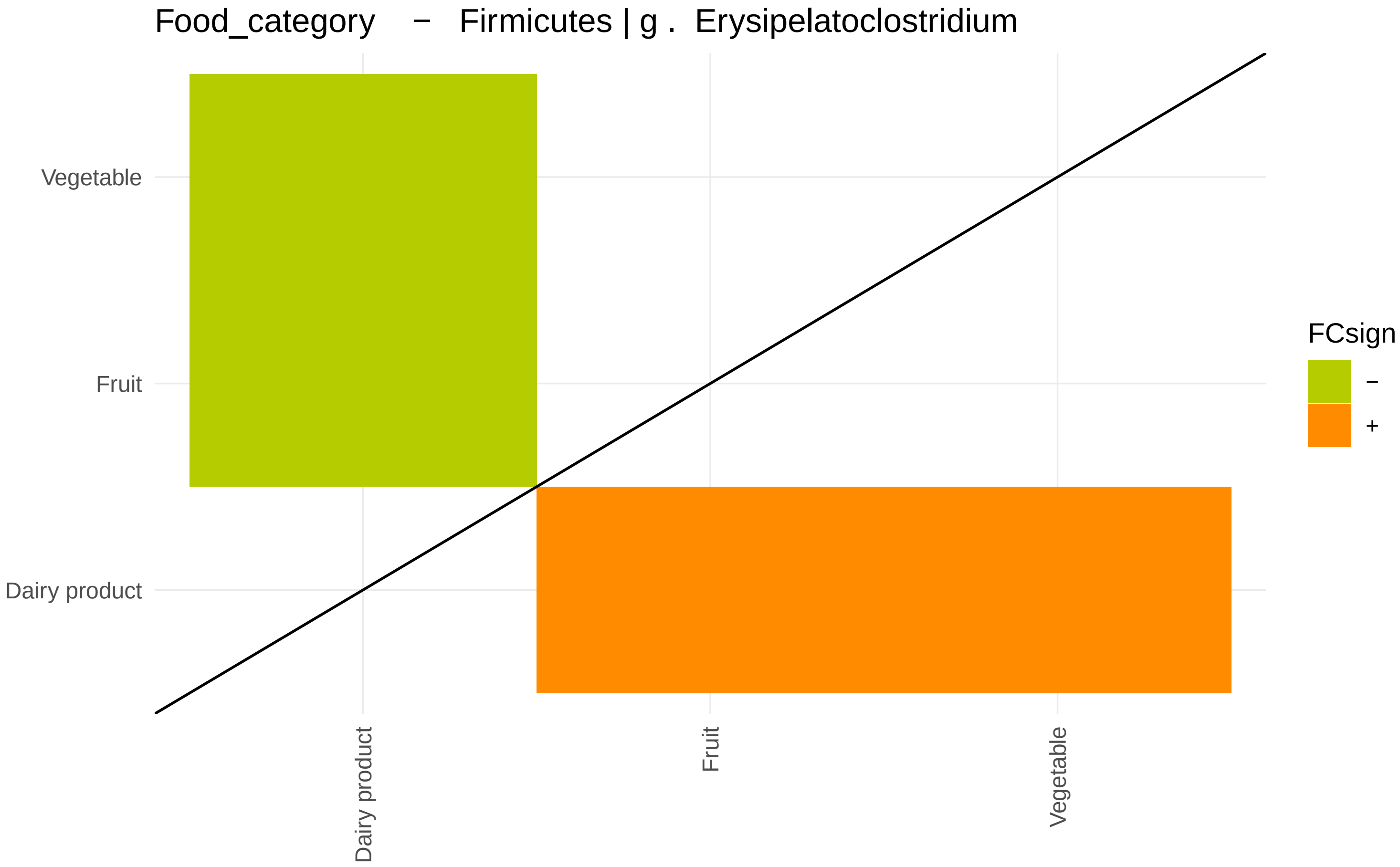

Food_category − Firmicutes | g . Erysipelatoclostridium
Vegetable
Fruit
Dairy product
Dairy product
Fruit
Vegetable
FCsign
−
+

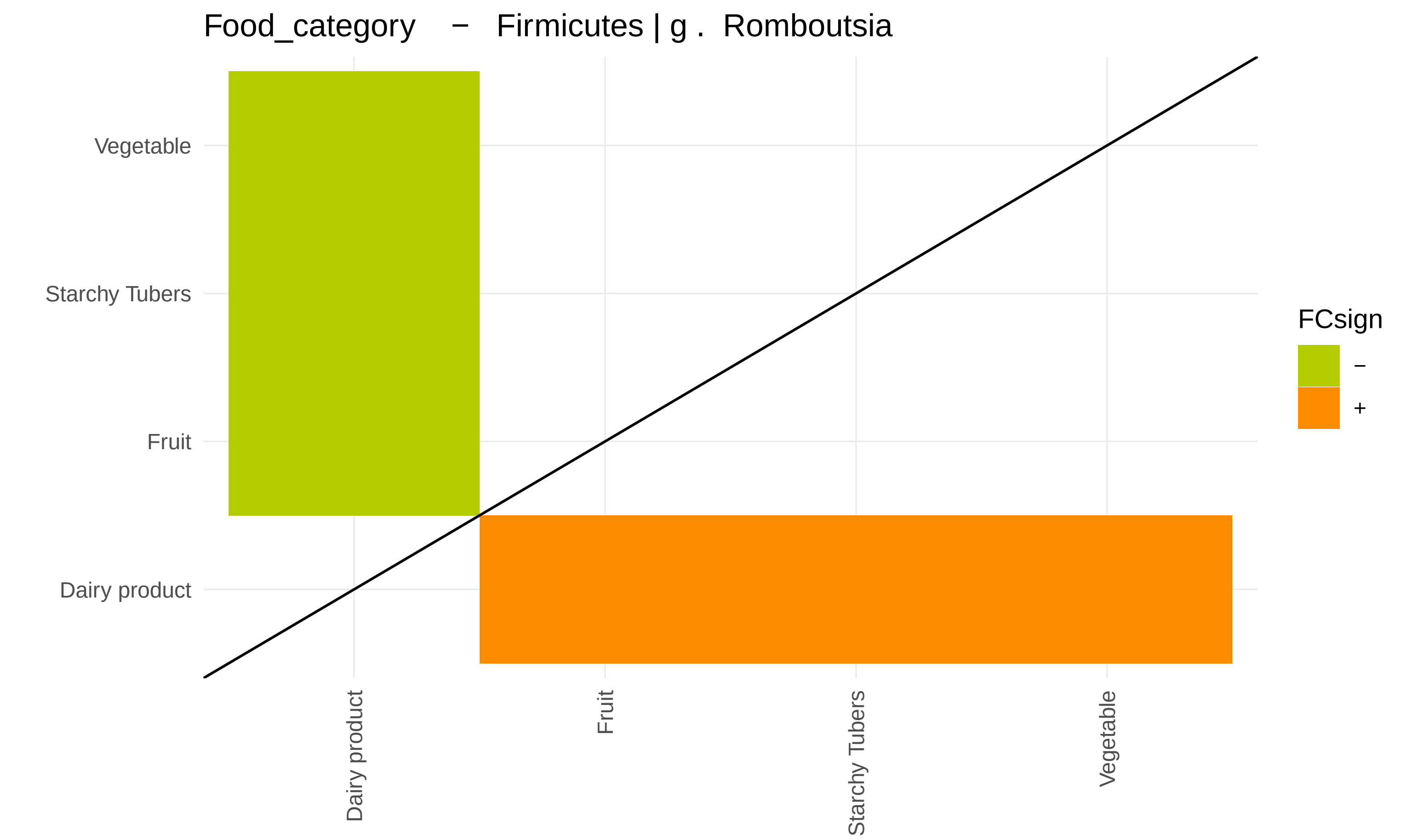

Food_category − Firmicutes | g . Romboutsia
Vegetable
Starchy Tubers
Fruit
Dairy product
Dairy product
Fruit
Starchy Tubers
Vegetable
FCsign
−
+

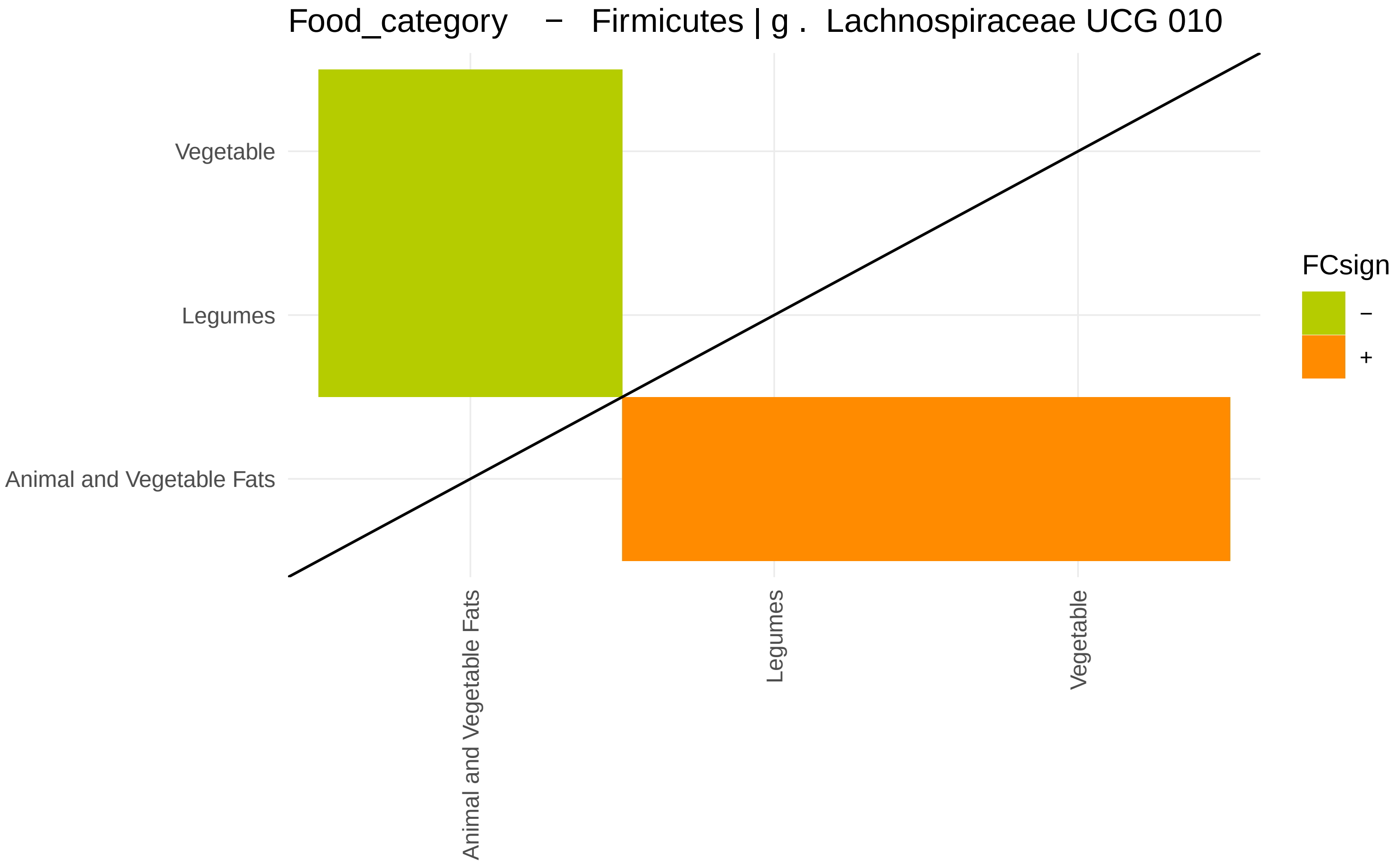
Food_category − Firmicutes | g . Lachnospiraceae UCG 010
Vegetable
Legumes
Animal and Vegetable Fats
Animal and Vegetable Fats
Legumes
Vegetable
FCsign
−
+

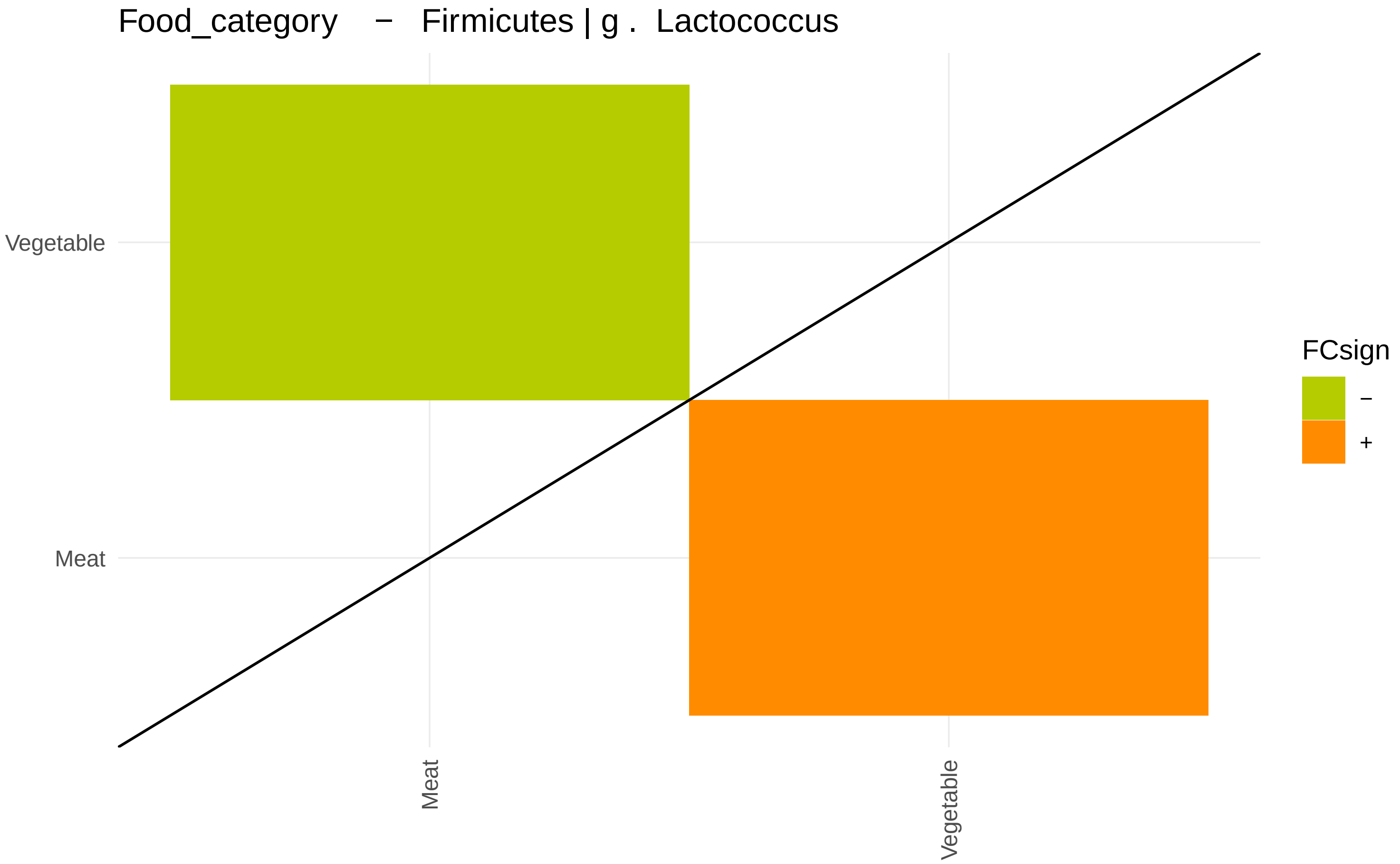
Food_category − Firmicutes | g . Lactococcus
Vegetable
Meat
Meat
Vegetable
FCsign
−
+

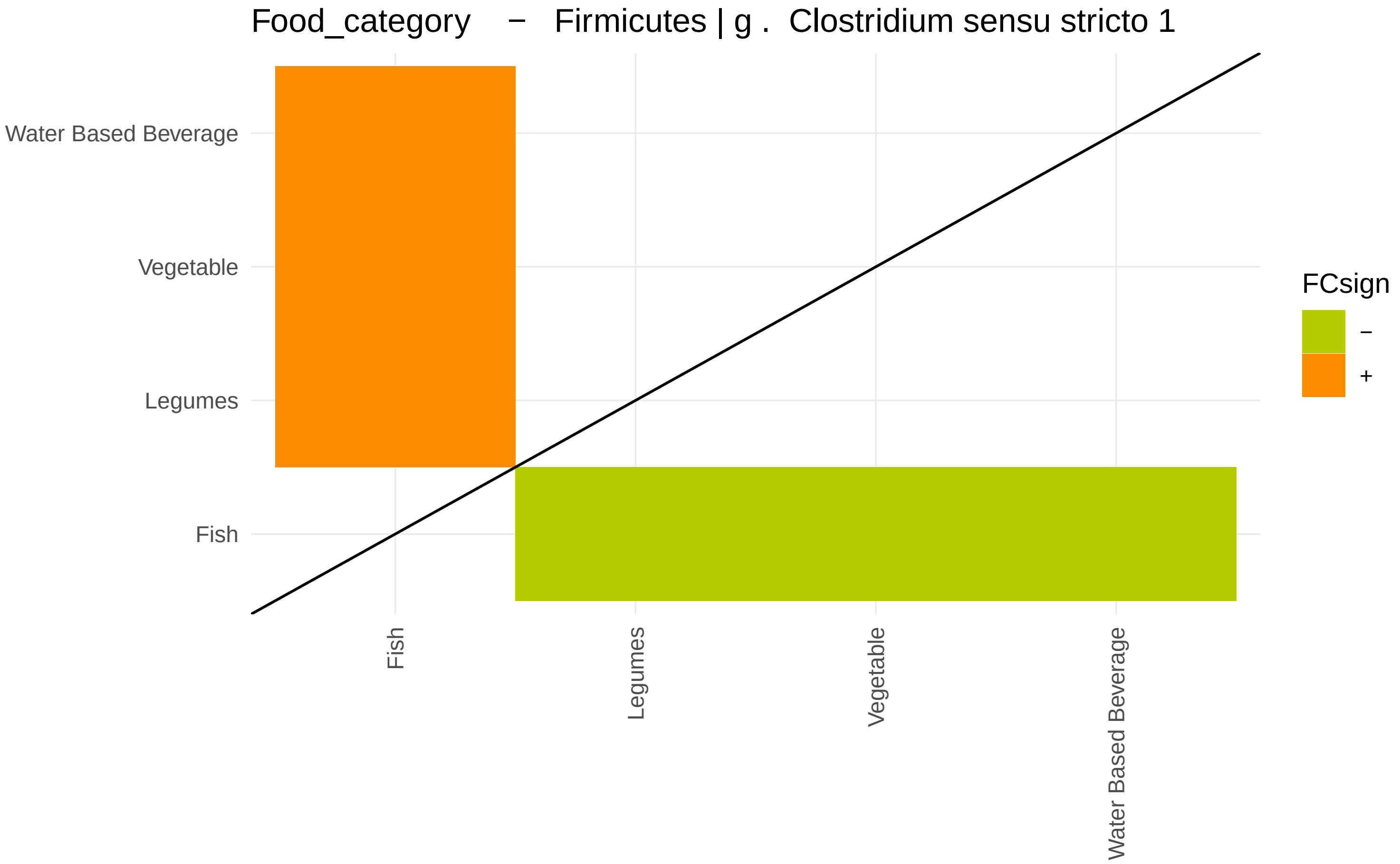
Food_category − Firmicutes | g . Clostridium sensu stricto 1
Water Based Beverage
Vegetable
Legumes
Fish
Fish
Legumes
Vegetable
Water Based Beverage
FCsign
−
+

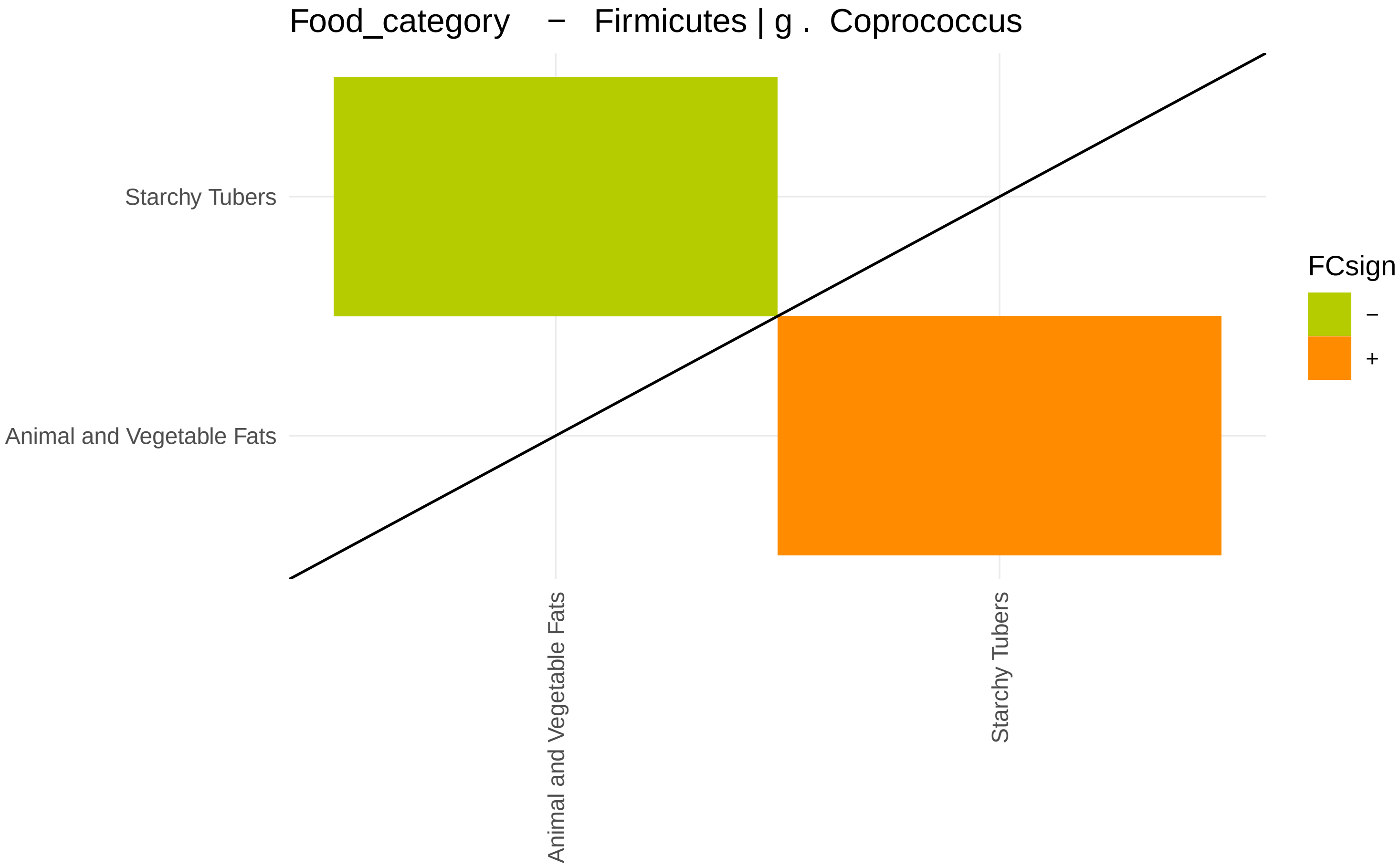
Food_category − Firmicutes | g . Coprococcus
Starchy Tubers
Animal and Vegetable Fats
Animal and Vegetable Fats
Starchy Tubers
FCsign
−
+

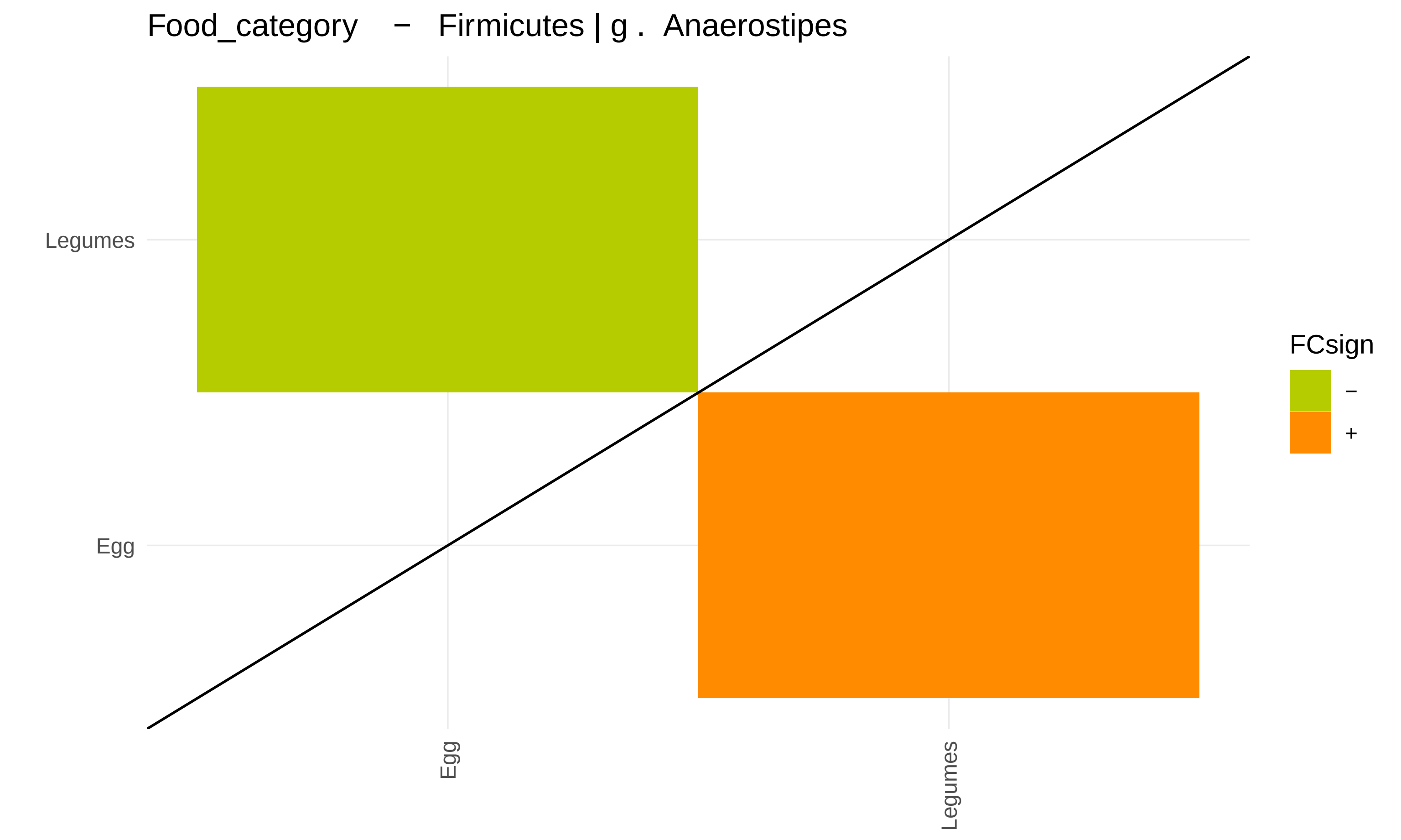

Food_category − Firmicutes | g . Anaerostipes
Legumes
Egg
Egg
Legumes
FCsign
−
+
