# Supplementary material for: Effects of different foods and cooking methods on the gut microbiota: an in vitro approach
Source: Front Microbiol. 2024 Jan 8;14:1334623. doi: 10.3389/fmicb.2023.1334623 (PMC10800916; doi:10.3389/fmicb.2023.1334623)
Supplement: Supplementary Figure 2 — Heatmap of relative abundance differences at species level between food categories. Y-axis food categories have higher (orange), or lower (green) abundance of a given species against X-axis food categories. The ANCOM method was used for comparisons with the Benjamini–Hochberg procedure for false discovery rate control. Significant comparisons (q < 0.05) in all the individuals are represented (PDF). [file Data_Sheet_2.PDF]

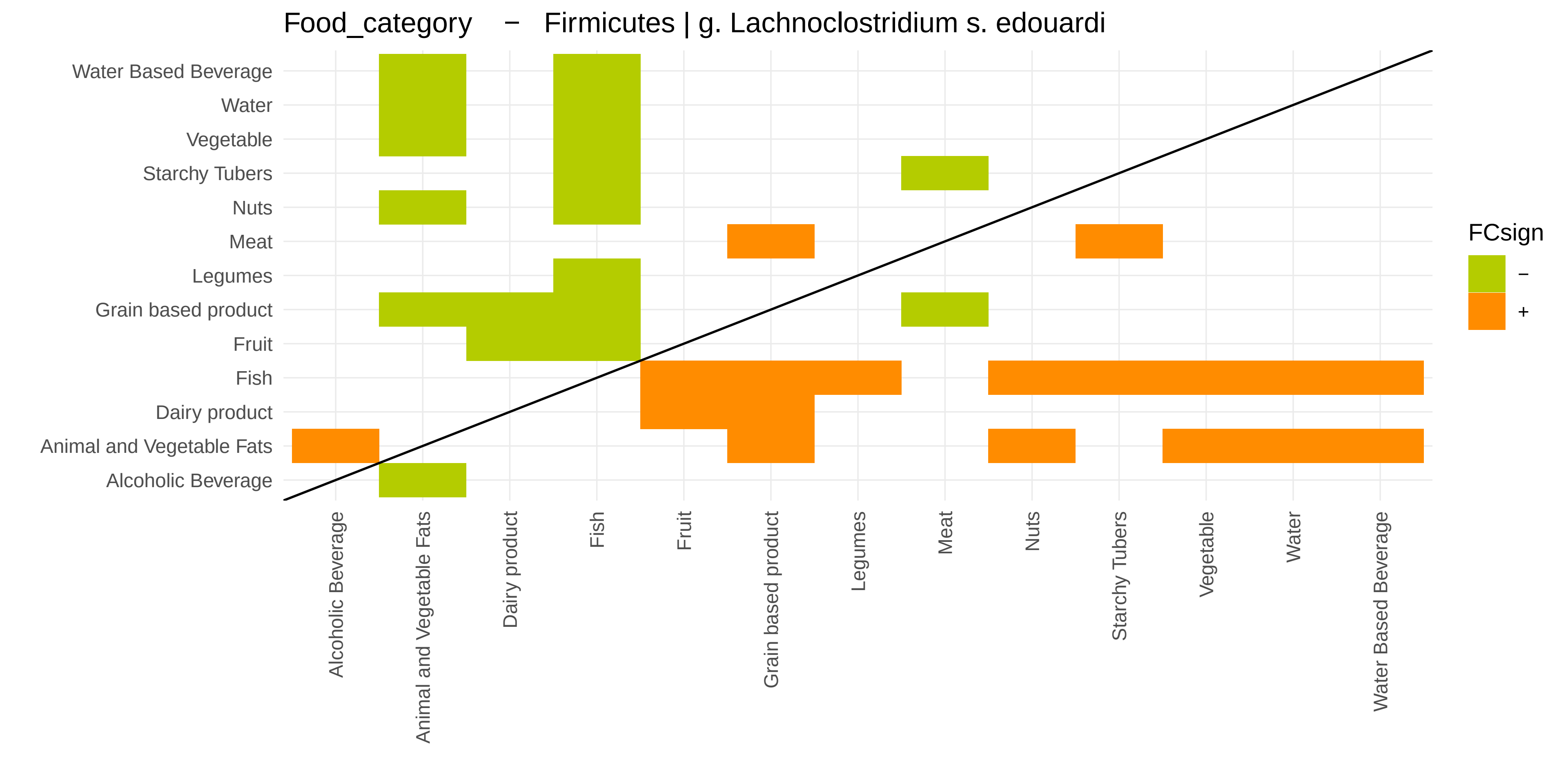

Food_category − Firmicutes | g. Lachnoclostridium s. edouardi
Water Based Beverage
Water
Vegetable
Starchy Tubers
Nuts
Meat
Legumes
Grain based product
Fruit
Fish
Dairy product
Animal and Vegetable Fats
Alcoholic Beverage
Alcoholic Beverage
Animal and Vegetable Fats
Dairy product
Fish
Fruit
Grain based product
Legumes
Meat
Nuts
Starchy Tubers
Vegetable
Water
Water Based Beverage
FCsign
−
+

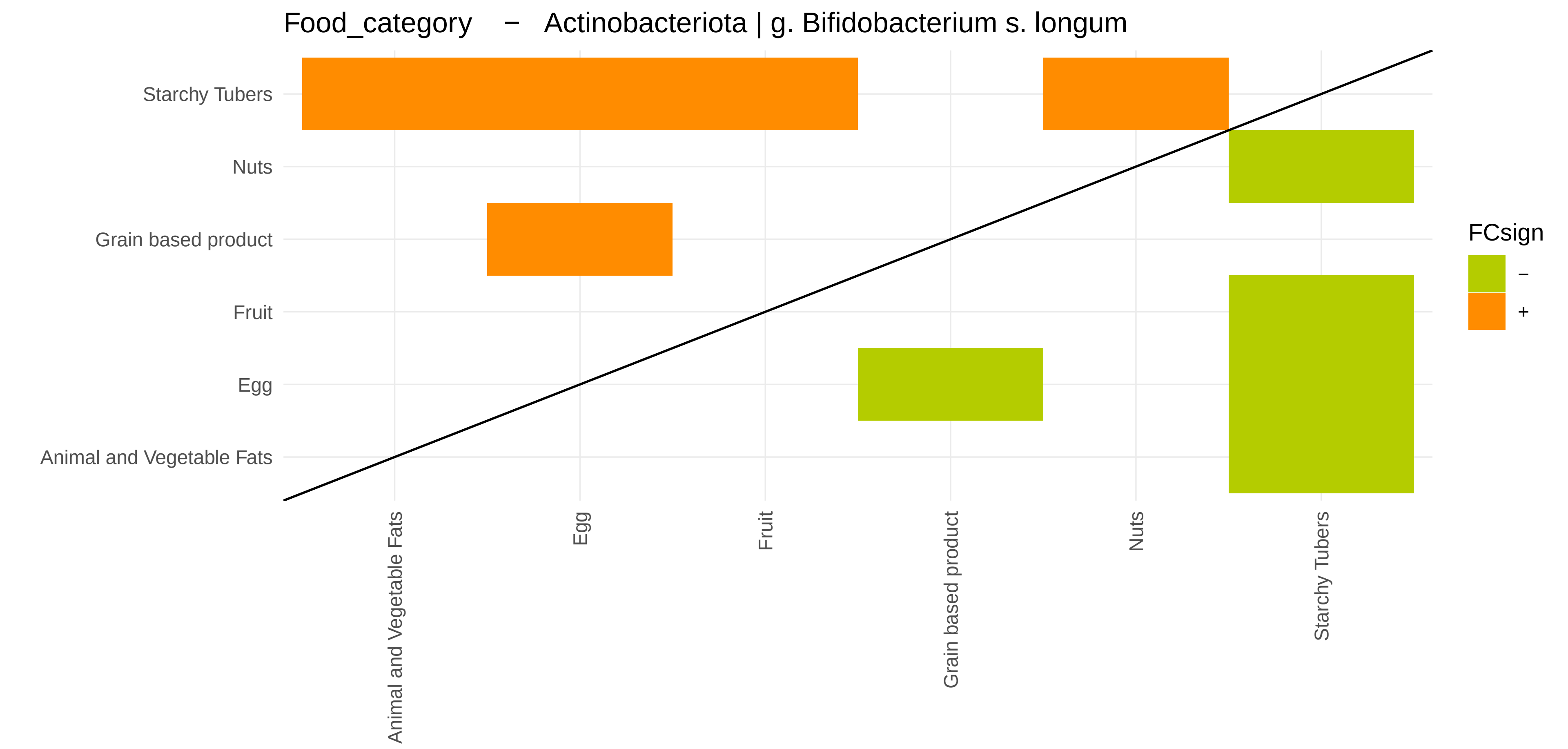

Food_category − Actinobacteriota | g. Bifidobacterium s. longum
Starchy Tubers
Nuts
Grain based product
Fruit
Egg
Animal and Vegetable Fats
Animal and Vegetable Fats
Egg
Fruit
Grain based product
Nuts
Starchy Tubers
FCsign
−
+

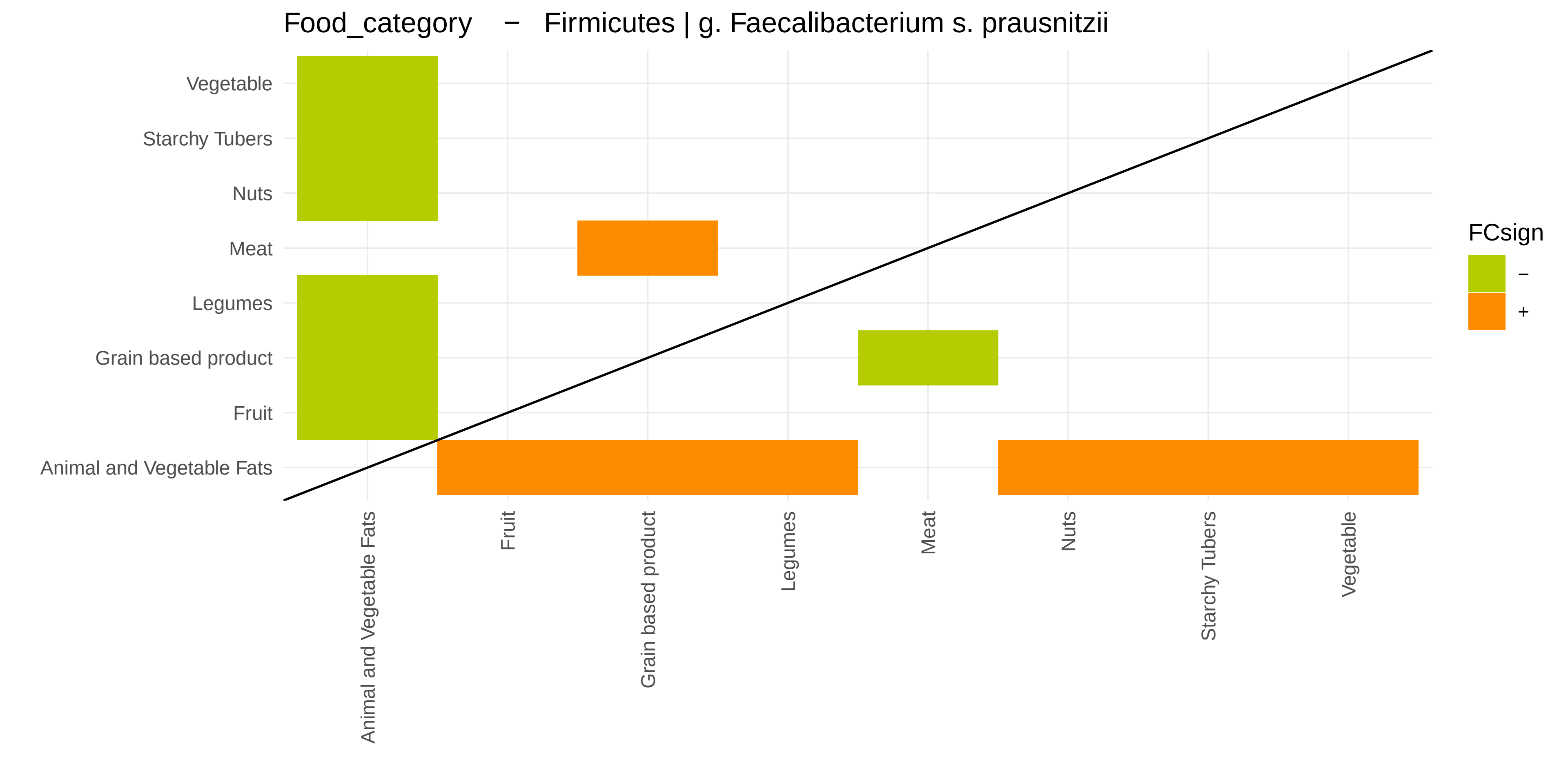

Food_category − Firmicutes | g. Faecalibacterium s. prausnitzii
Vegetable
Starchy Tubers
Nuts
Meat
Legumes
Grain based product
Fruit
Animal and Vegetable Fats
Animal and Vegetable Fats
Fruit
Grain based product
Legumes
Meat
Nuts
Starchy Tubers
Vegetable
FCsign
−
+

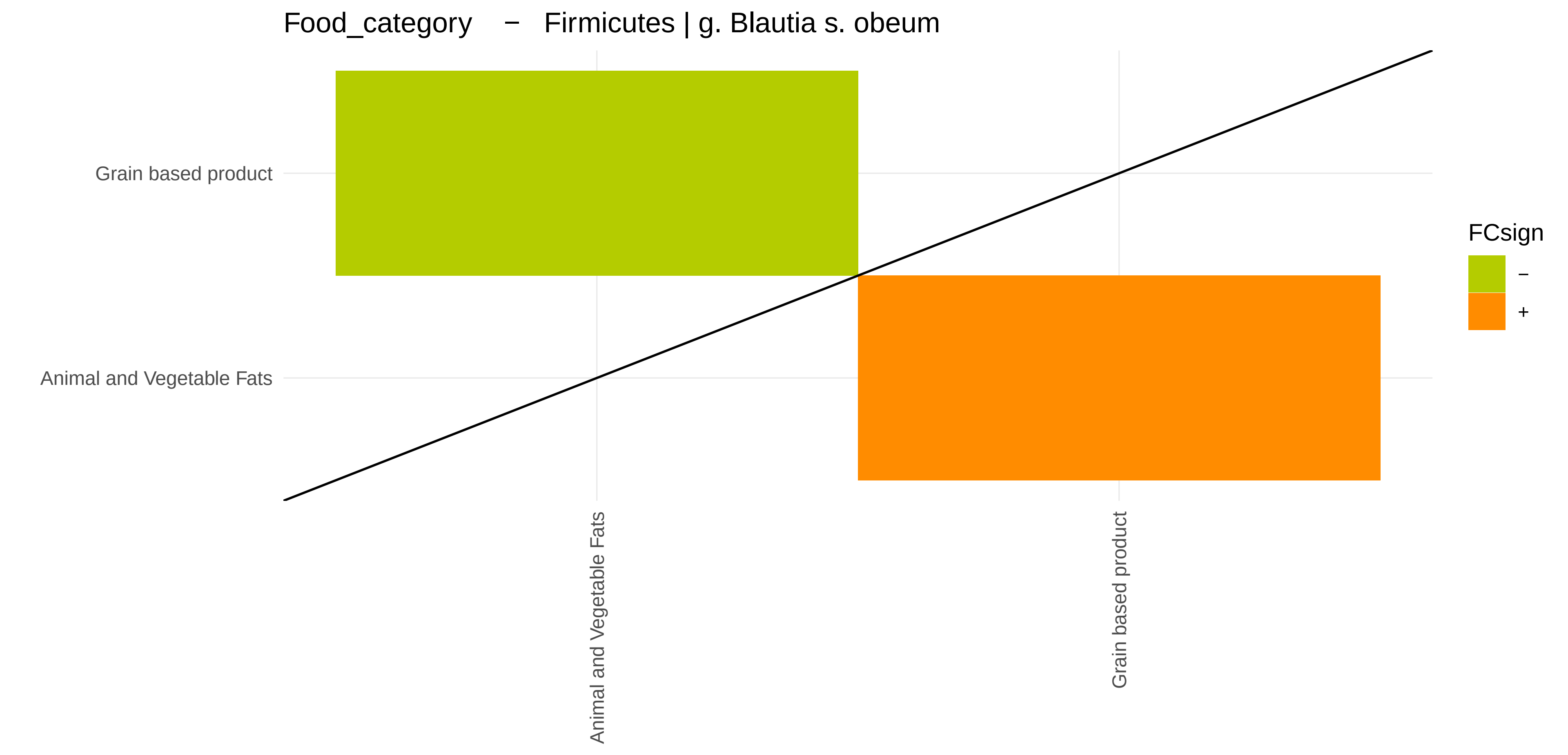

Food_category − Firmicutes | g. Blautia s. obeum
Grain based product
Animal and Vegetable Fats
Animal and Vegetable Fats
Grain based product
FCsign
−
+

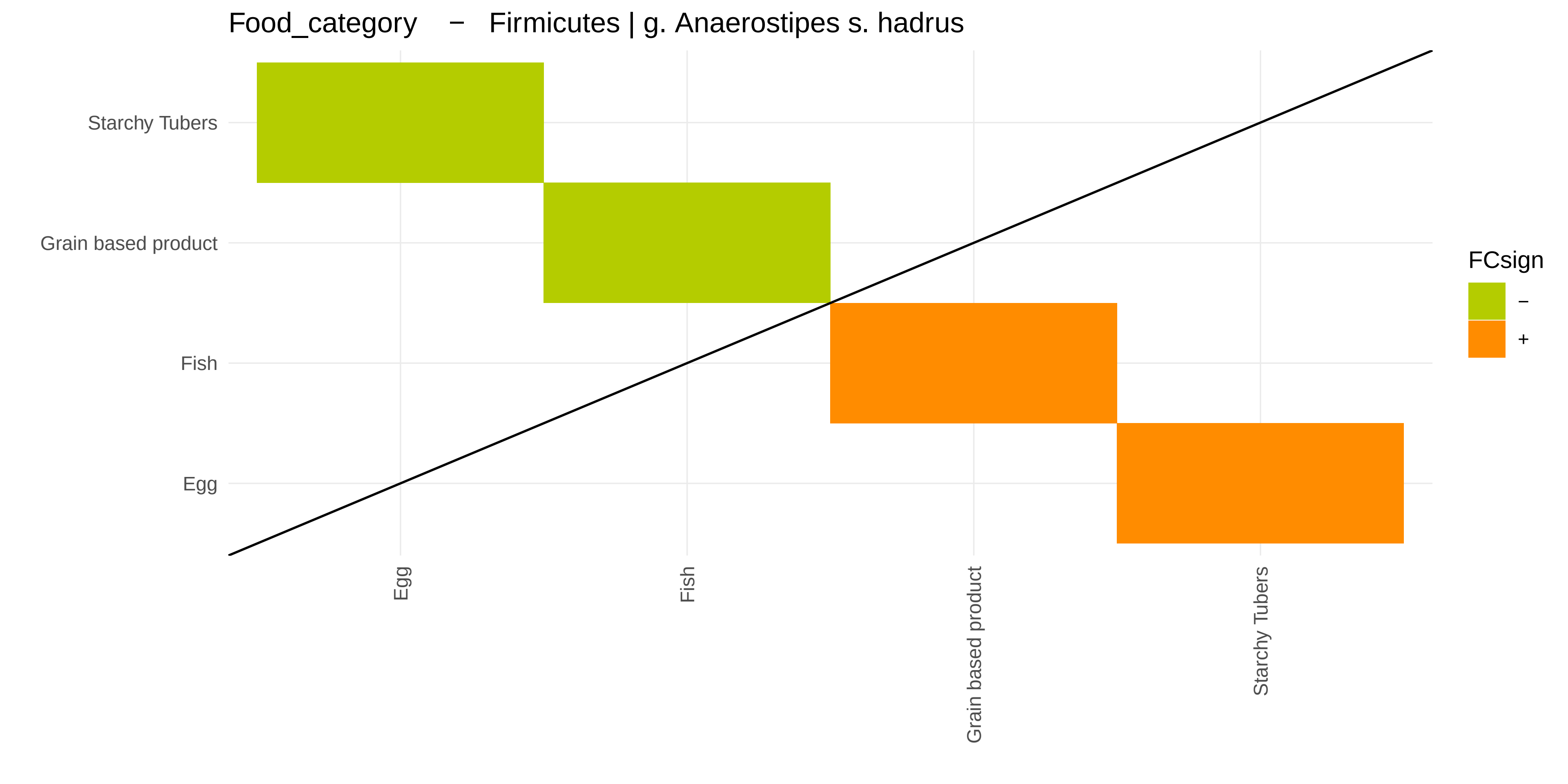
Food_category − Firmicutes | g. Anaerostipes s. hadrus
Starchy Tubers
Grain based product
Fish
Egg
Egg
Fish
Grain based product
Starchy Tubers
FCsign
−
+

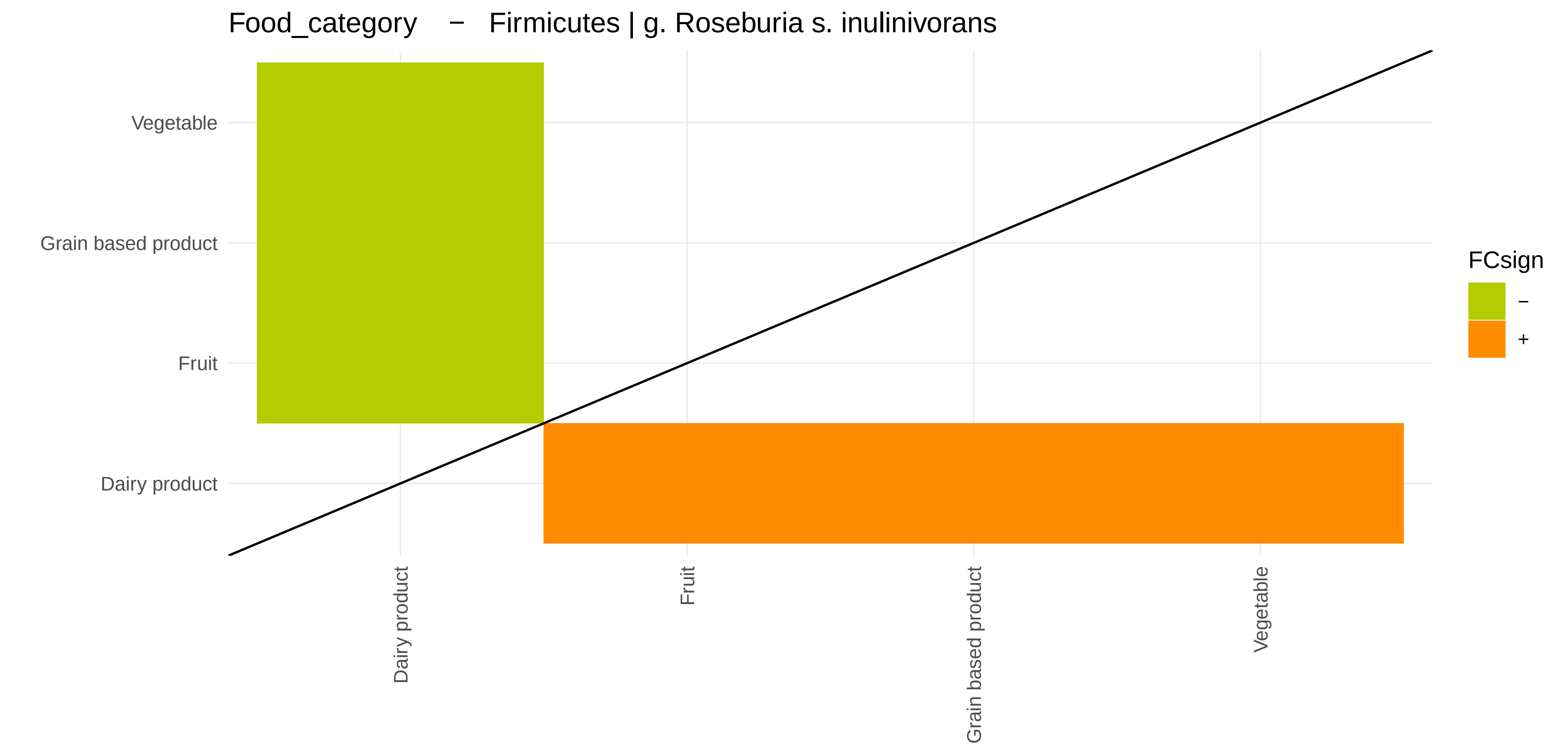
Food_category − Firmicutes | g. Roseburia s. inulinivorans
Vegetable
Grain based product
Fruit
Dairy product
Dairy product
Fruit
Grain based product
Vegetable
FCsign
−
+

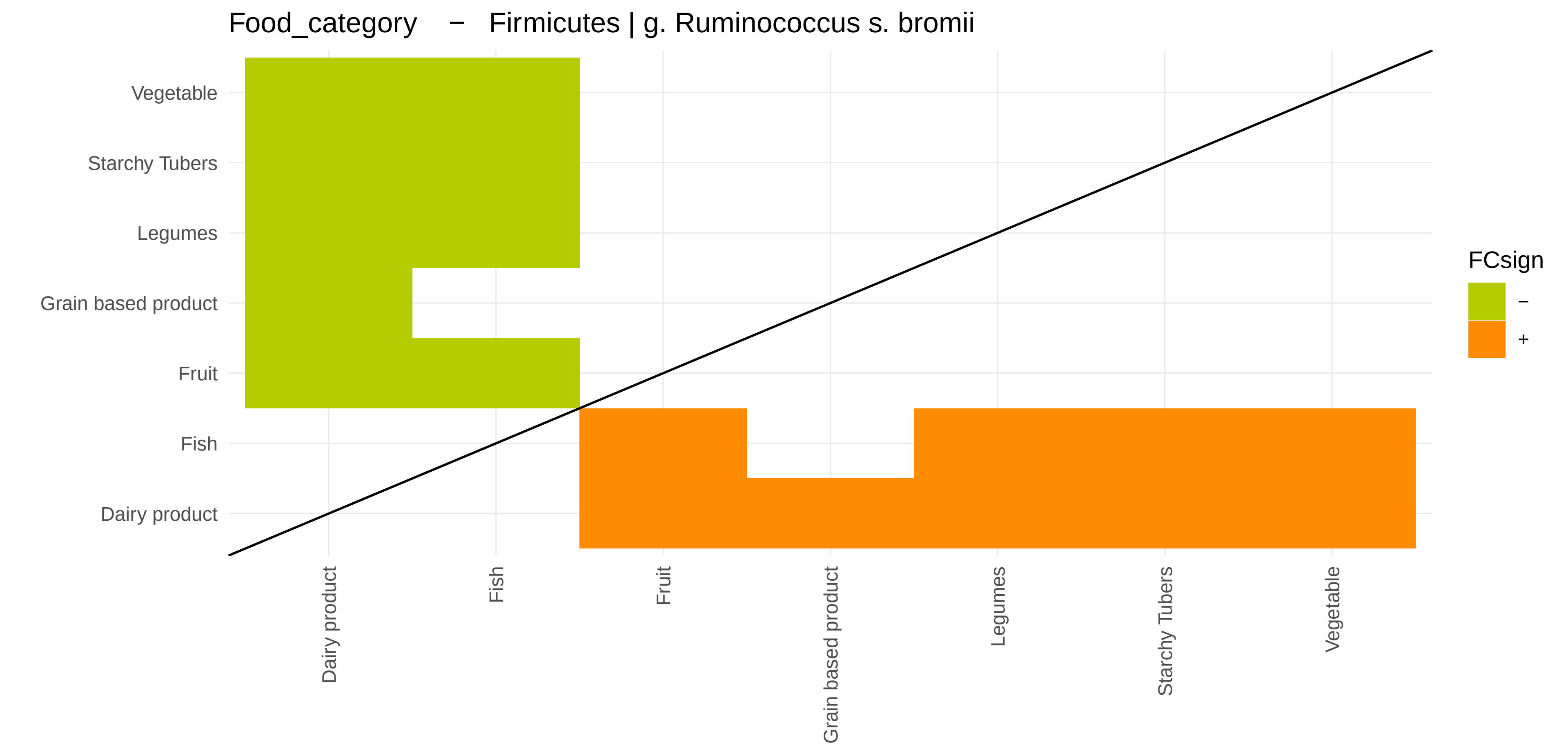

Food_category − Firmicutes | g. Ruminococcus s. bromii
Vegetable
Starchy Tubers
Legumes
Grain based product
Fruit
Fish
Dairy product
Dairy product
Fish
Fruit
Grain based product
Legumes
Starchy Tubers
Vegetable
FCsign
−
+

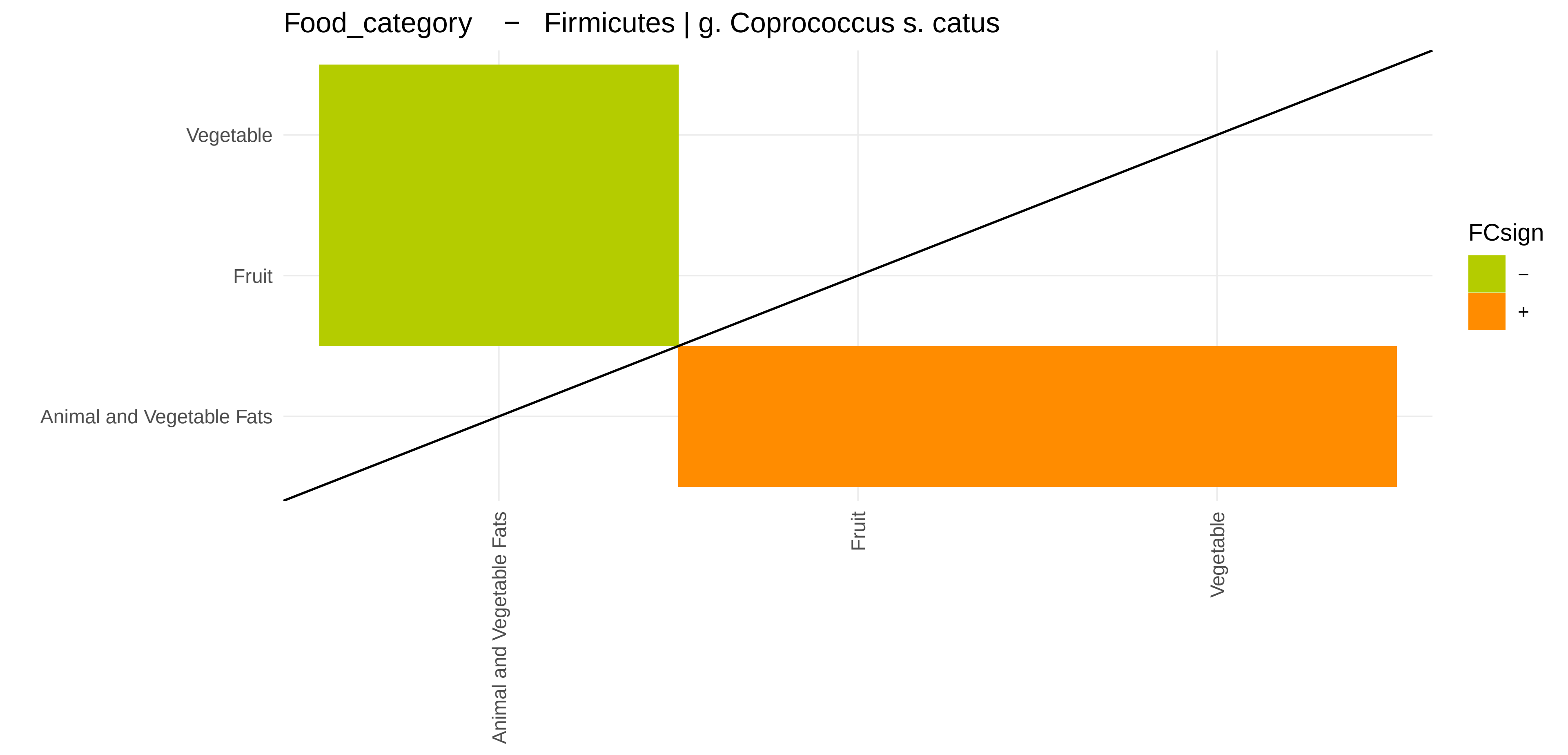
Food_category − Firmicutes | g. Coprococcus s. catus
Vegetable
Fruit
Animal and Vegetable Fats
Animal and Vegetable Fats
Fruit
Vegetable
FCsign
−
+

Food_category − Actinobacteriota | g. Adlercreutzia s. equolifaciens

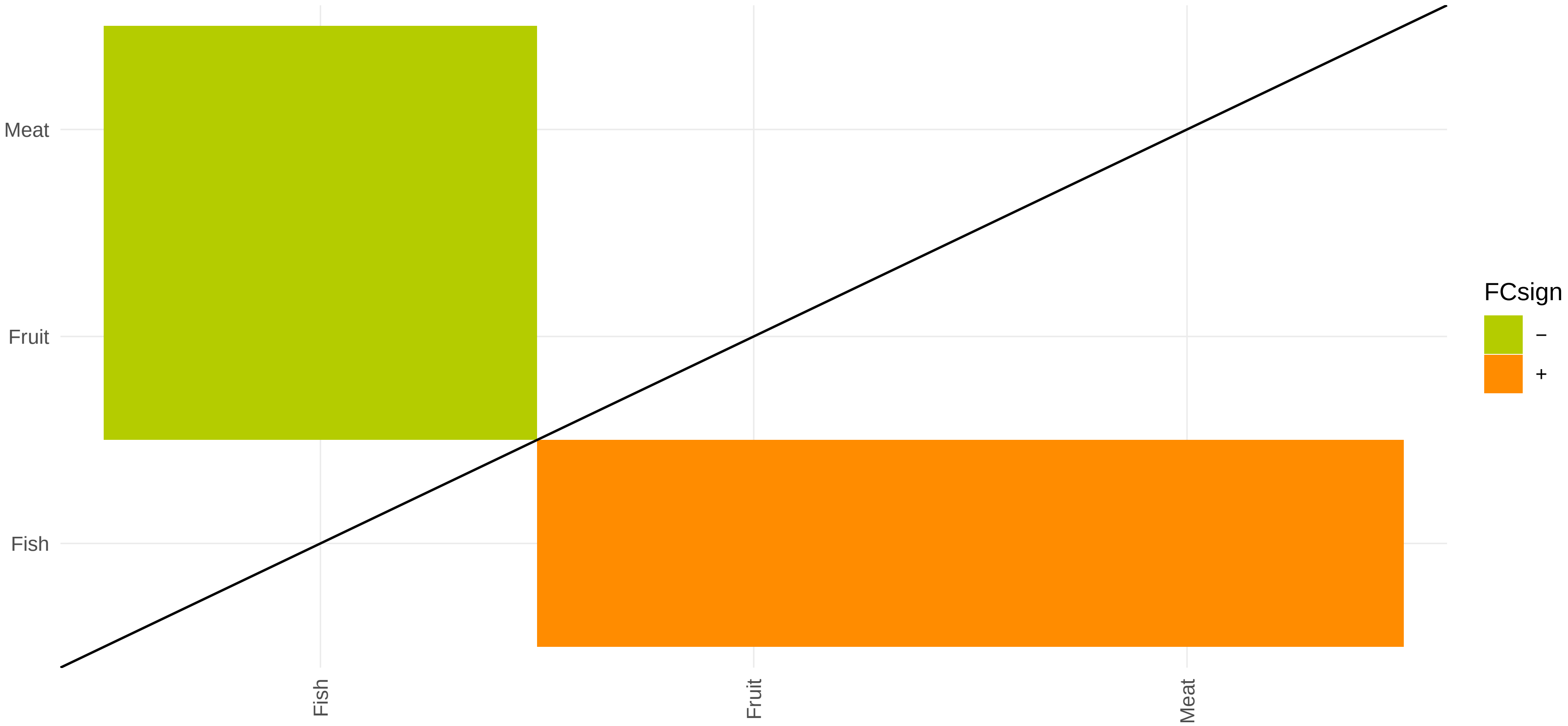

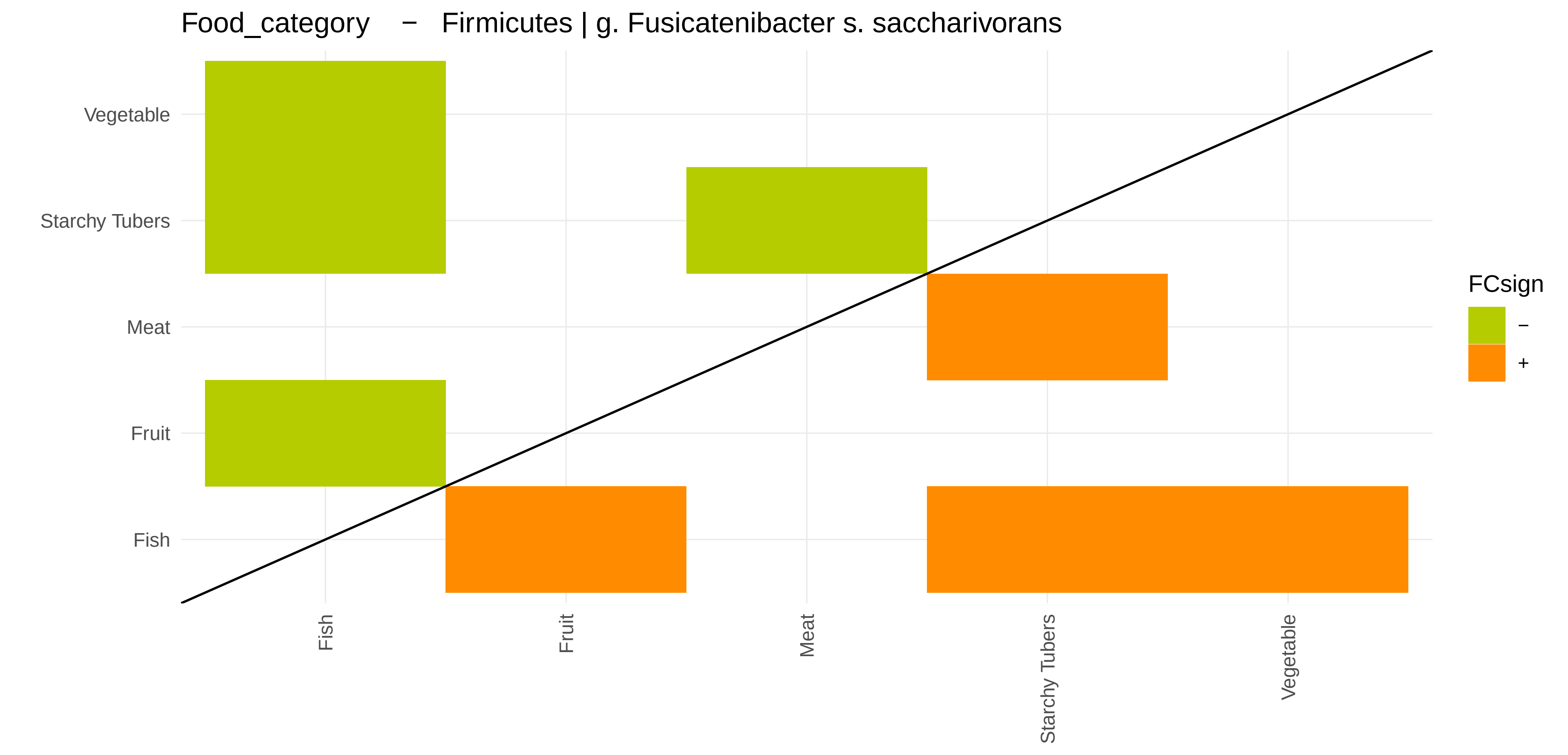
Food_category − Firmicutes | g. Fusicatenibacter s. saccharivorans
Vegetable
Starchy Tubers
Meat
Fruit
Fish
Fish
Fruit
Meat
Starchy Tubers
Vegetable
FCsign
−
+

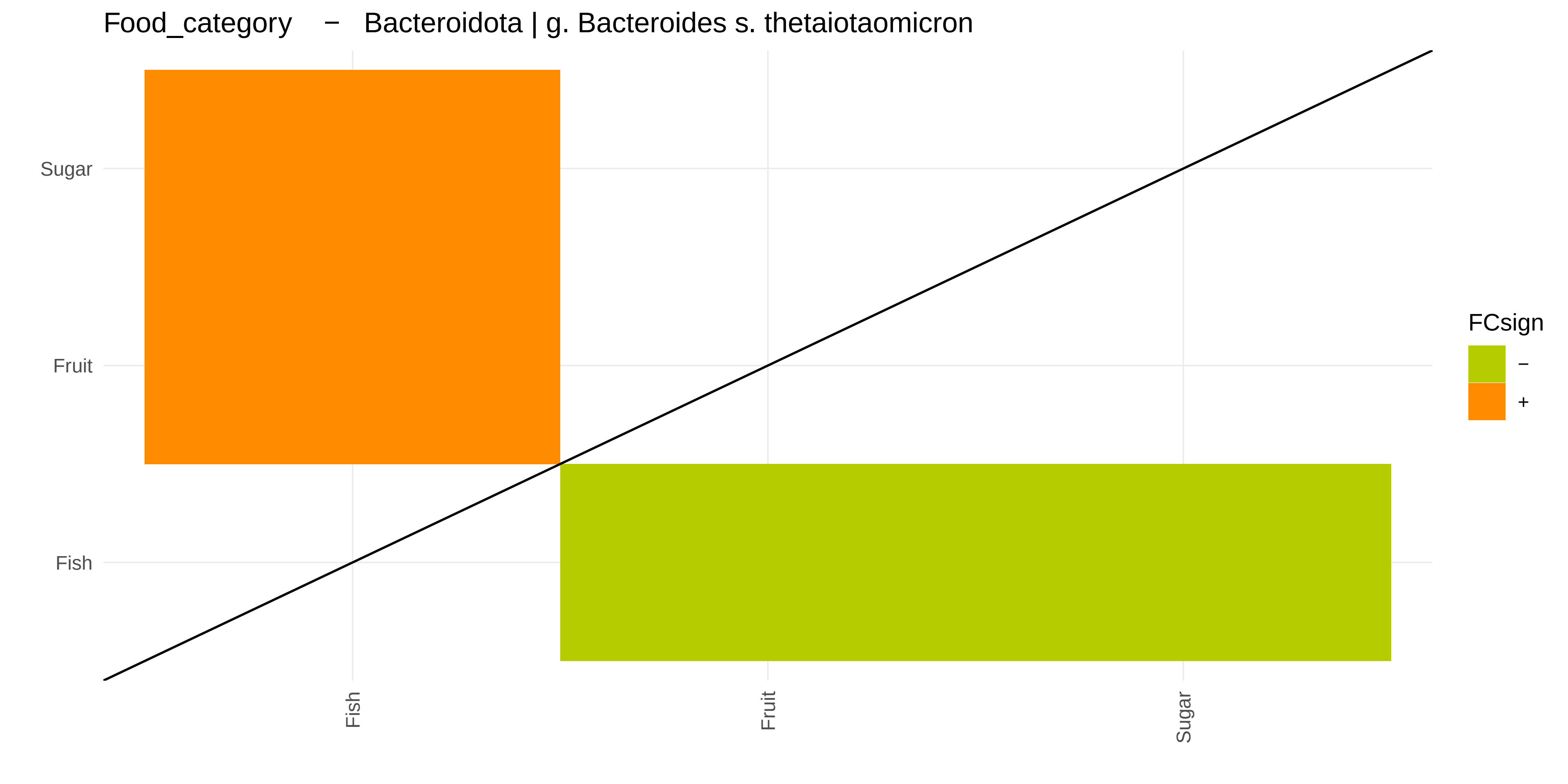

Food_category − Bacteroidota | g. Bacteroides s. thetaiotaomicron
Sugar
Fruit
Fish
Fish
Fruit
Sugar
FCsign
−
+

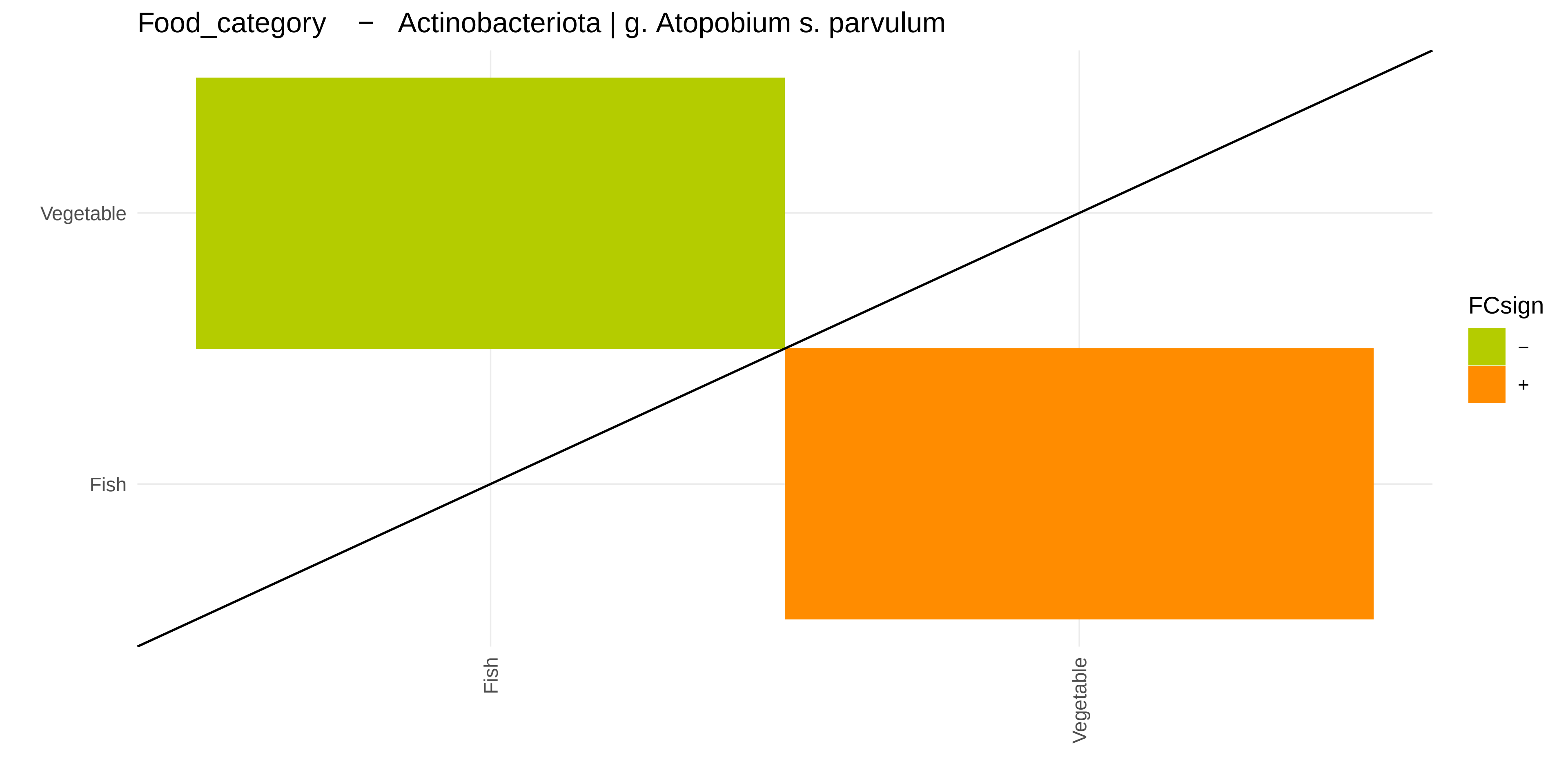
Food_category − Actinobacteriota | g. Atopobium s. parvulum
Vegetable
Fish
Fish
Vegetable
FCsign
−
+

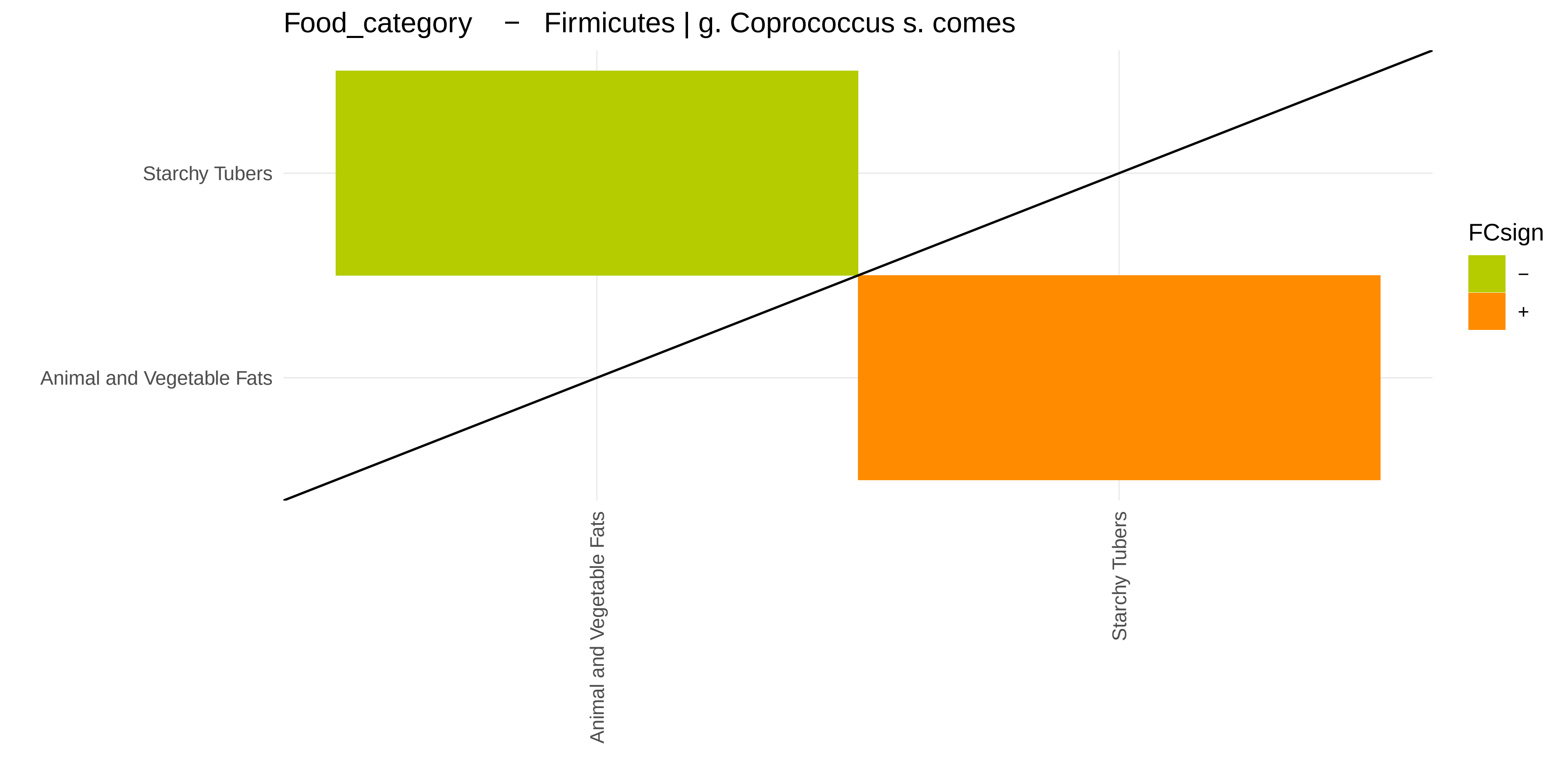
Food_category − Firmicutes | g. Coprococcus s. comes
Starchy Tubers
Animal and Vegetable Fats
Animal and Vegetable Fats
Starchy Tubers
FCsign
−
+

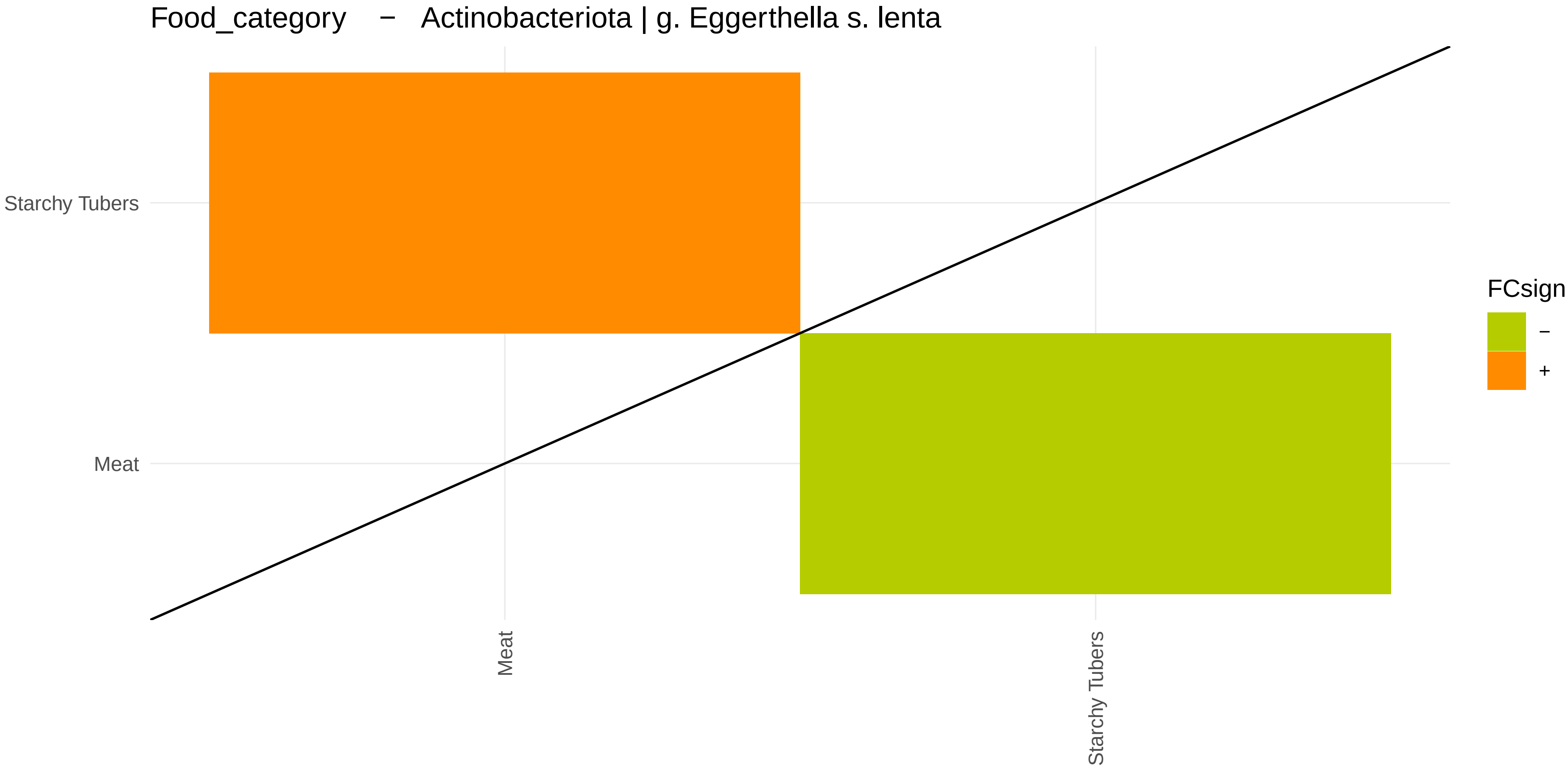

Food_category − Actinobacteriota | g. Eggerthella s. lenta
Starchy Tubers
Meat
Meat
Starchy Tubers
FCsign
−
+

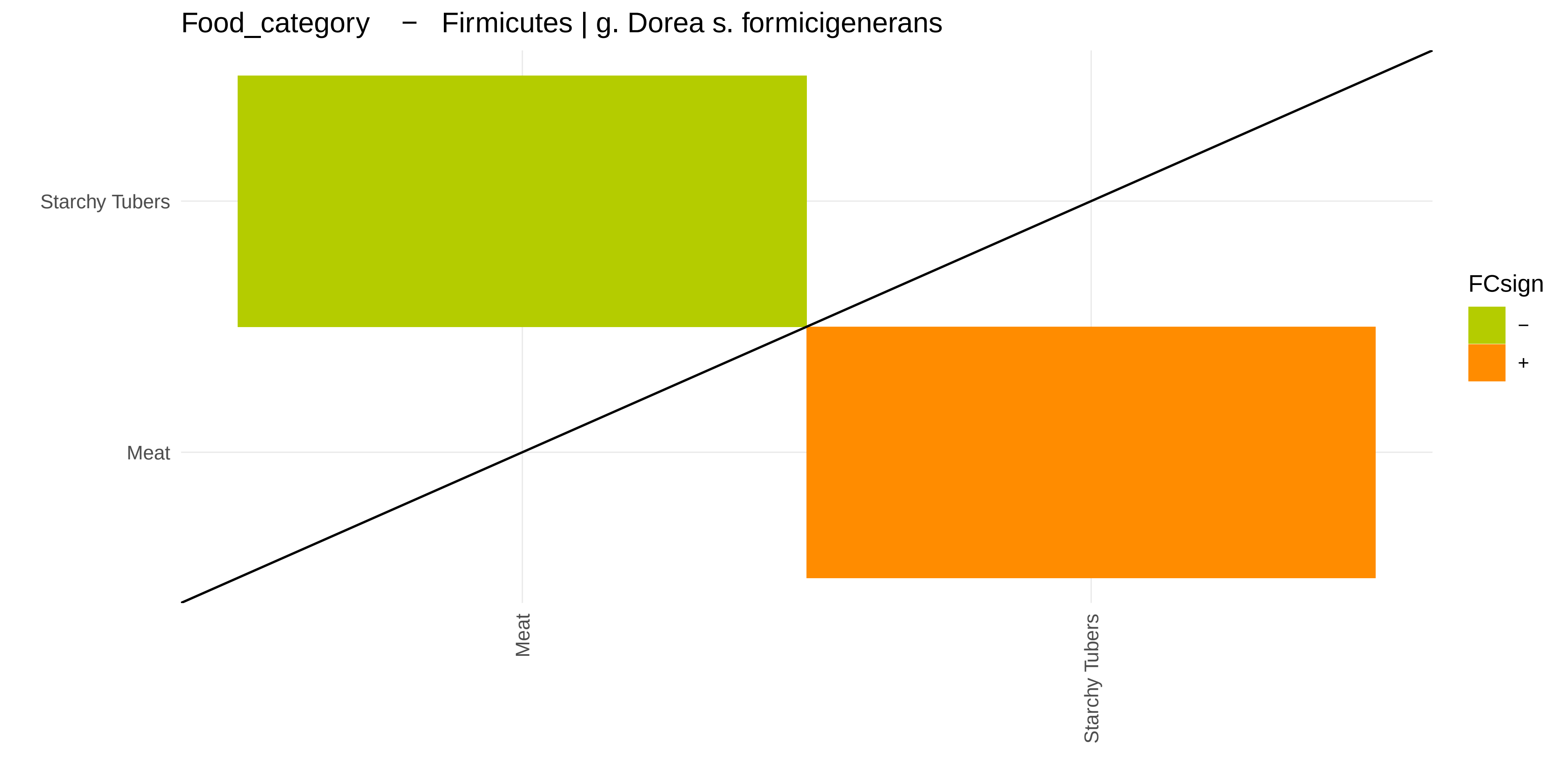

Food_category − Firmicutes | g. Dorea s. formicigenerans
Starchy Tubers
Meat
Meat
Starchy Tubers
FCsign
−
+

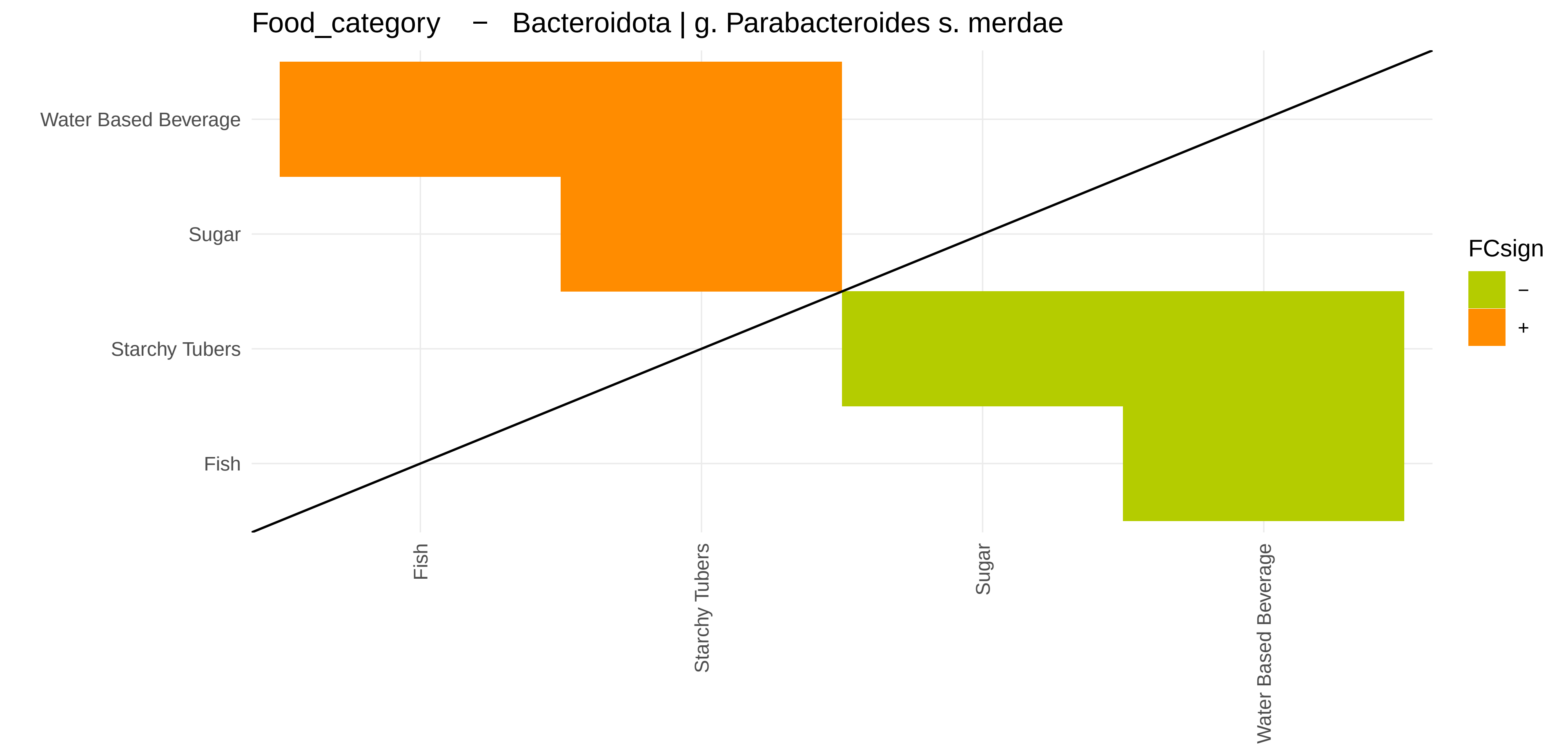
Food_category − Bacteroidota | g. Parabacteroides s. merdae
Water Based Beverage
Sugar
Starchy Tubers
Fish
Fish
Starchy Tubers
Sugar
Water Based Beverage
FCsign
−
+

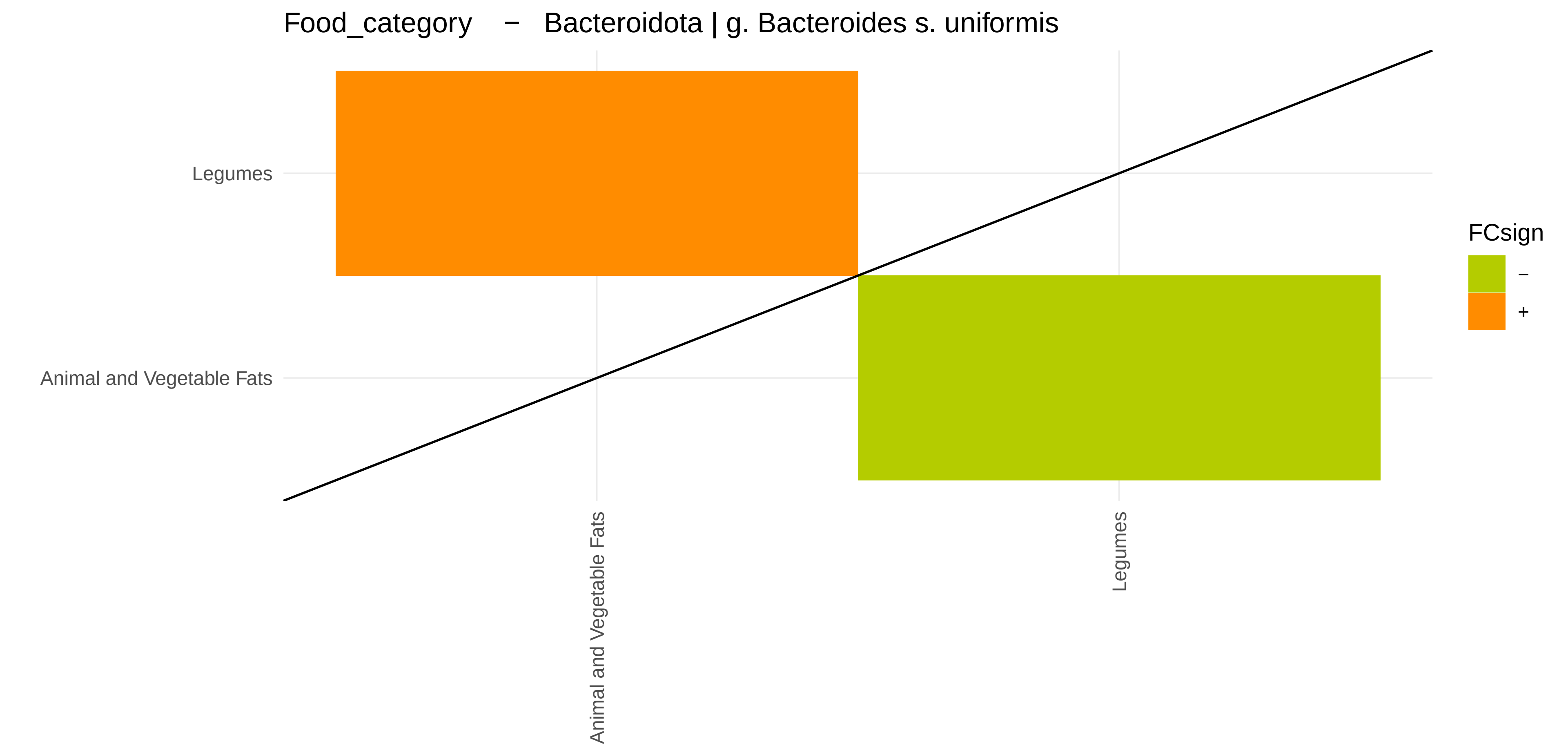

Food_category − Bacteroidota | g. Bacteroides s. uniformis
Legumes
Animal and Vegetable Fats
Animal and Vegetable Fats
Legumes
FCsign
−
+

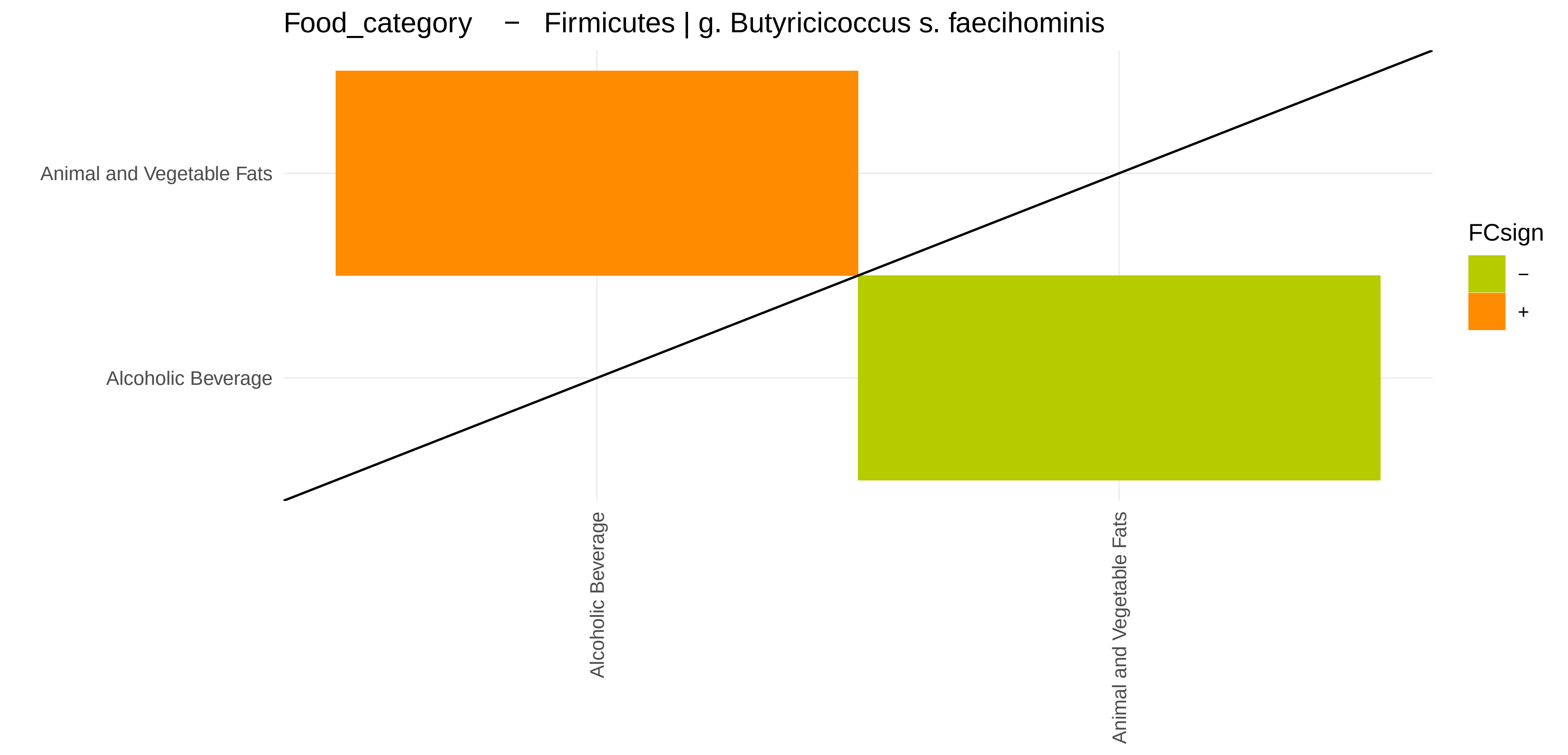

Food_category − Firmicutes | g. Butyricicoccus s. faecihominis
Animal and Vegetable Fats
Alcoholic Beverage
Alcoholic Beverage
Animal and Vegetable Fats
FCsign
−
+
