# Supplementary material for: Effects of different foods and cooking methods on the gut microbiota: an in vitro approach
Source: Front Microbiol. 2024 Jan 8;14:1334623. doi: 10.3389/fmicb.2023.1334623 (PMC10800916; doi:10.3389/fmicb.2023.1334623)
Supplement: Supplementary Figure 3 — Heatmap of relative abundance differences at genus level between foods. Y-axis foods have higher (orange), or lower (green) abundance of a given genus against X-axis foods. The ANCOM method was used for comparisons with the Benjamini–Hochberg procedure for false discovery rate control. Significant comparisons (q < 0.05) in all the individuals are represented (PDF). [file Data_Sheet_3.PDF]

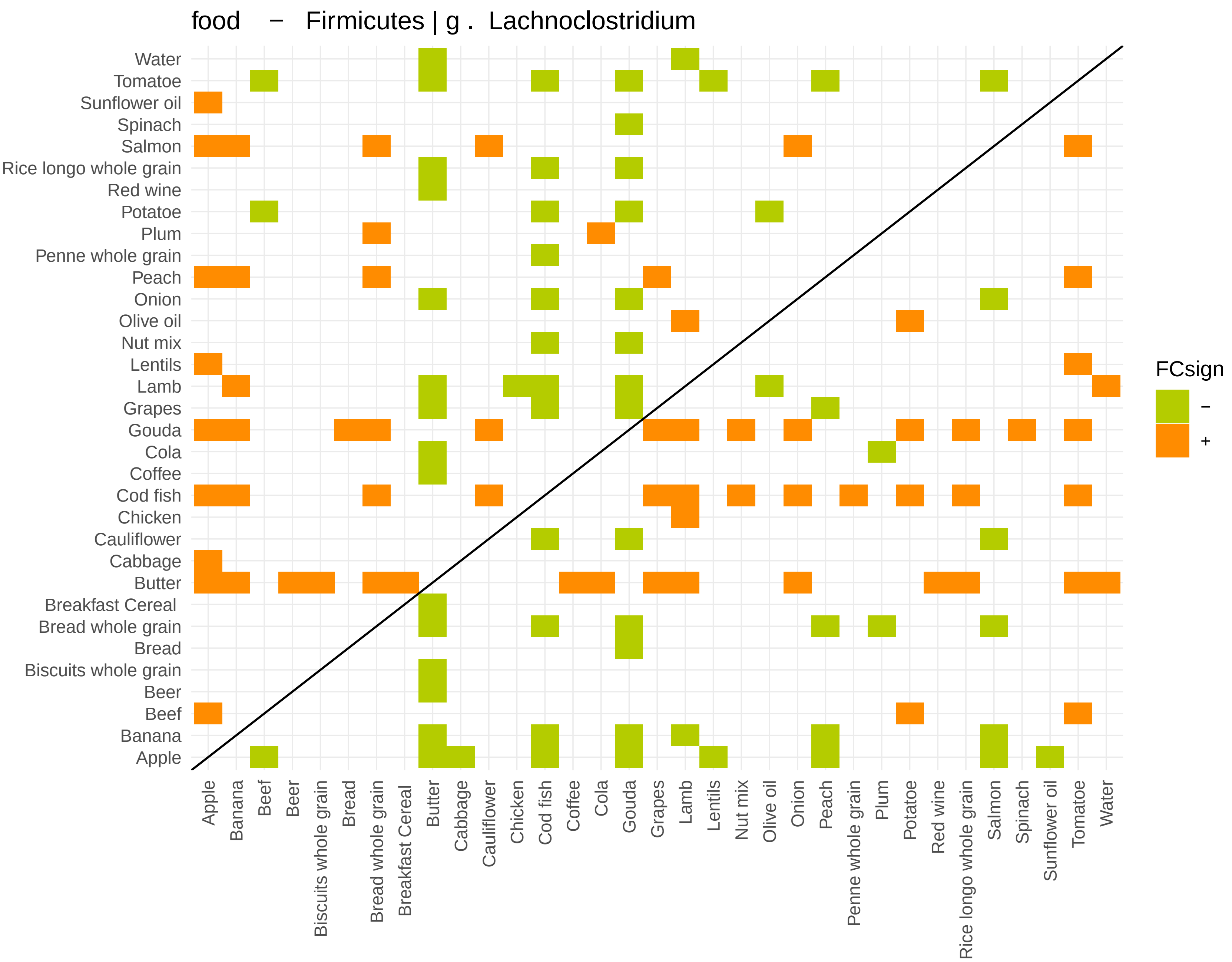
food − Firmicutes | g . Lachnoclostridium
Water
Tomatoe
Sunflower oil
Spinach
Salmon
Rice longo whole grain
Red wine
Potatoe
Plum
Penne whole grain
Peach
Onion
Olive oil
Nut mix
Lentils
Lamb
Grapes
Gouda
Cola
Coffee
Cod fish
Chicken
Cauliflower
Cabbage
Butter
Breakfast Cereal
Bread whole grain
Bread
Biscuits whole grain
Beer
Beef
Banana
Apple
FCsign
−
+

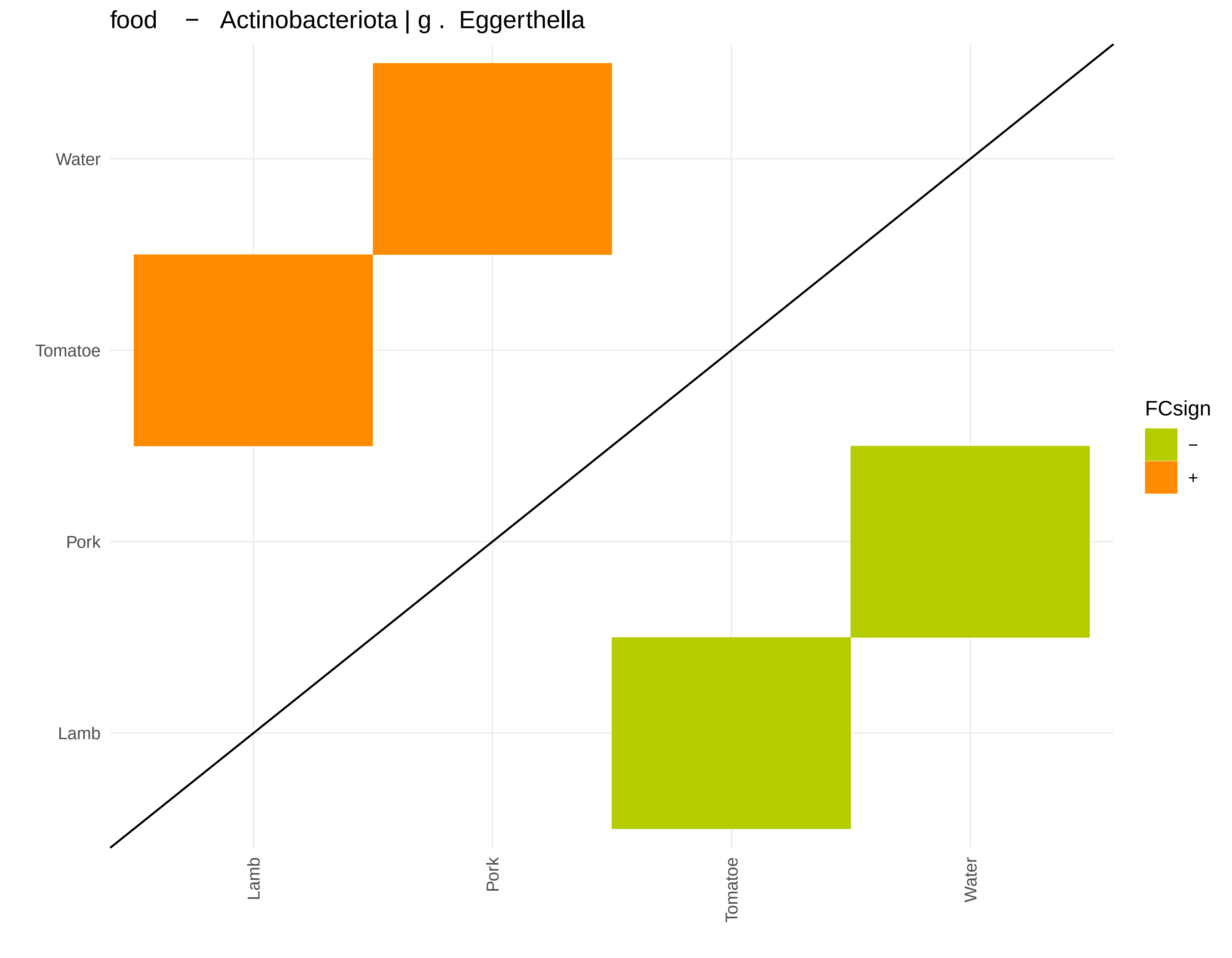
food − Actinobacteriota | g . Eggerthella
Water
Tomatoe
Pork
Lamb
Lamb
Pork
Tomatoe
Water
FCsign
−
+

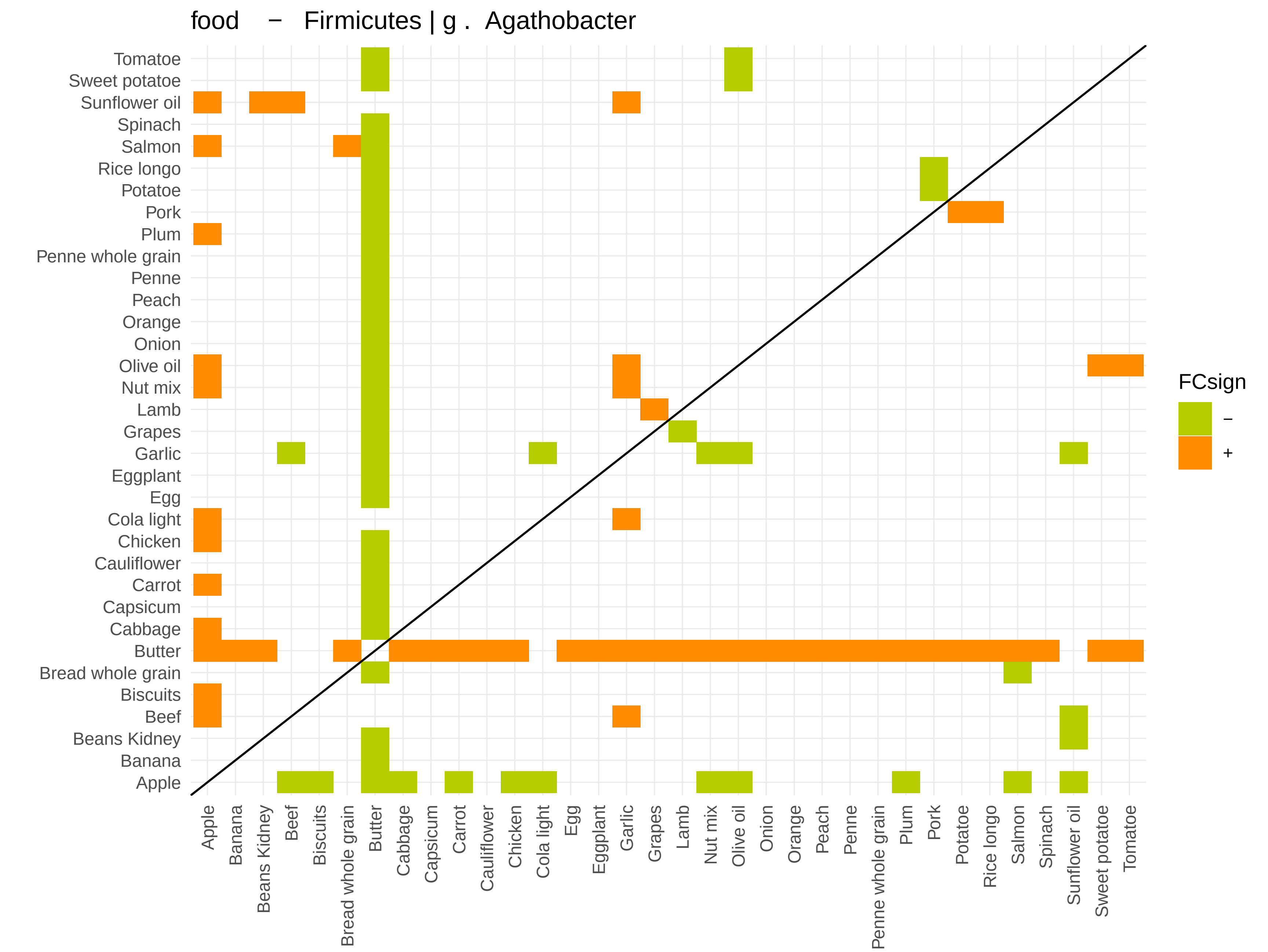

food − Firmicutes | g . Agathobacter
Tomatoe
Sweet potatoe
Sunflower oil
Spinach
Salmon
Rice longo
Potatoe
Pork
Plum
Penne whole grain
Penne
Peach
Orange
Onion
Olive oil
Nut mix
Lamb
Grapes
Garlic
Eggplant
Egg
Cola light
Chicken
Cauliflower
Carrot
Capsicum
Cabbage
Butter
Bread whole grain
Biscuits
Beef
Beans Kidney
Banana
Apple
FCsign
−
+

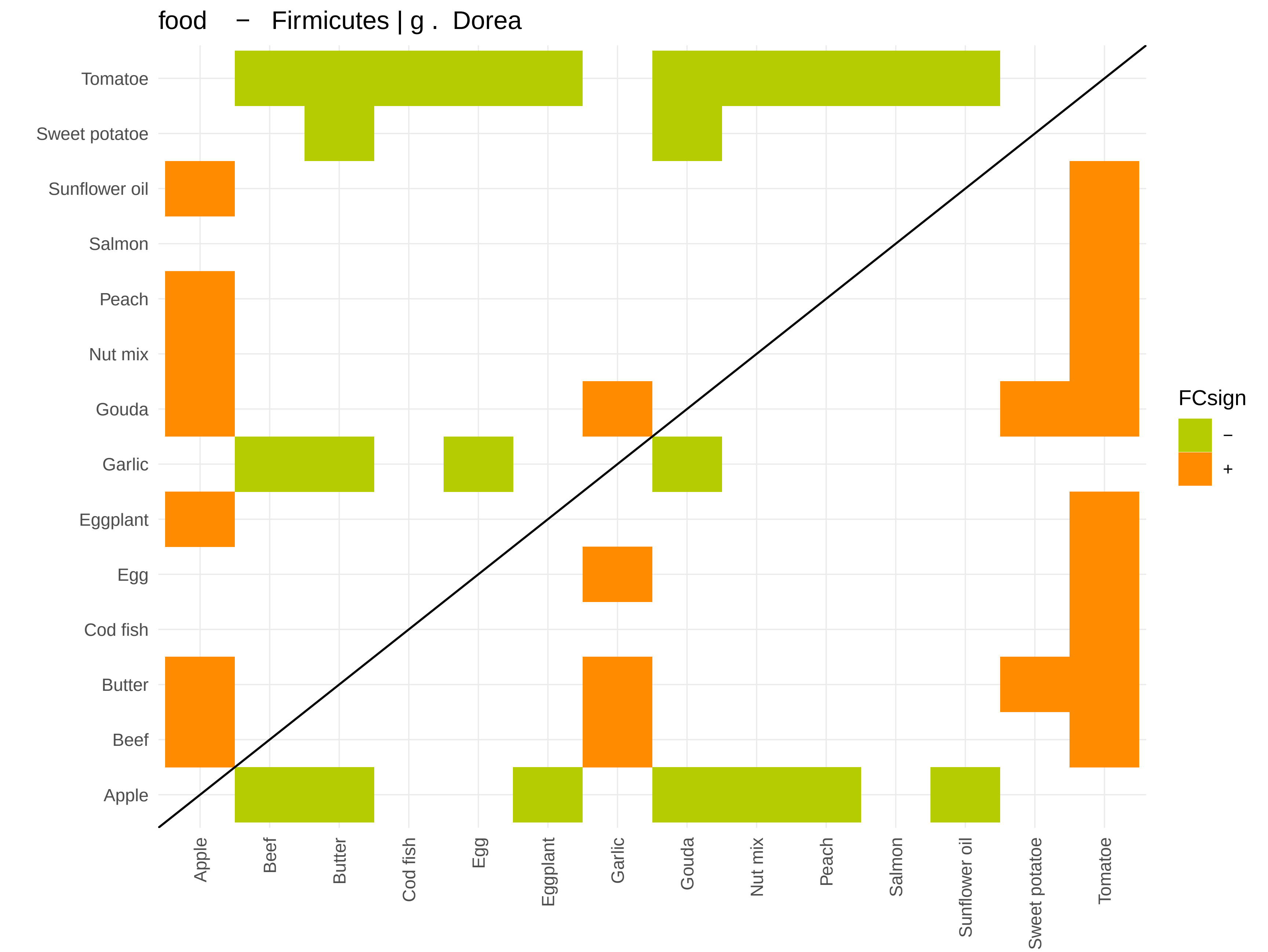
food − Firmicutes | g . Dorea
Tomatoe
Sweet potatoe
Sunflower oil
Salmon
Peach
Nut mix
Gouda
Garlic
Eggplant
Egg
Cod fish
Butter
Beef
Apple
Apple
Beef
Butter
Cod fish
Egg
Eggplant
Garlic
Gouda
Nut mix
Peach
Salmon
Sunflower oil
Sweet potatoe
Tomatoe
FCsign
−
+

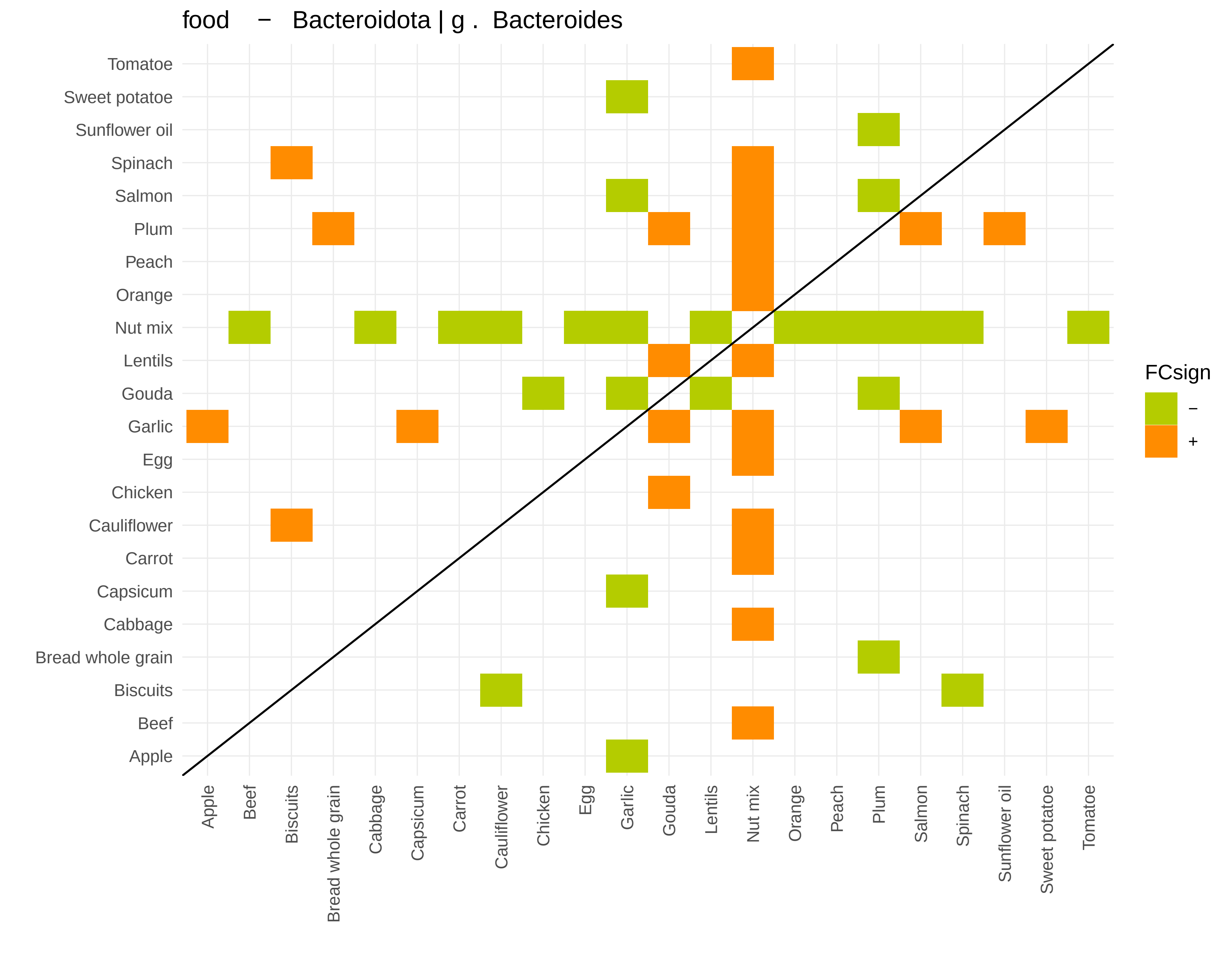
food − Bacteroidota | g . Bacteroides
Tomatoe
Sweet potatoe
Sunflower oil
Spinach
Salmon
Plum
Peach
Orange
Nut mix
Lentils
Gouda
Garlic
Egg
Chicken
Cauliflower
Carrot
Capsicum
Cabbage
Bread whole grain
Biscuits
Beef
Apple
FCsign
−
+

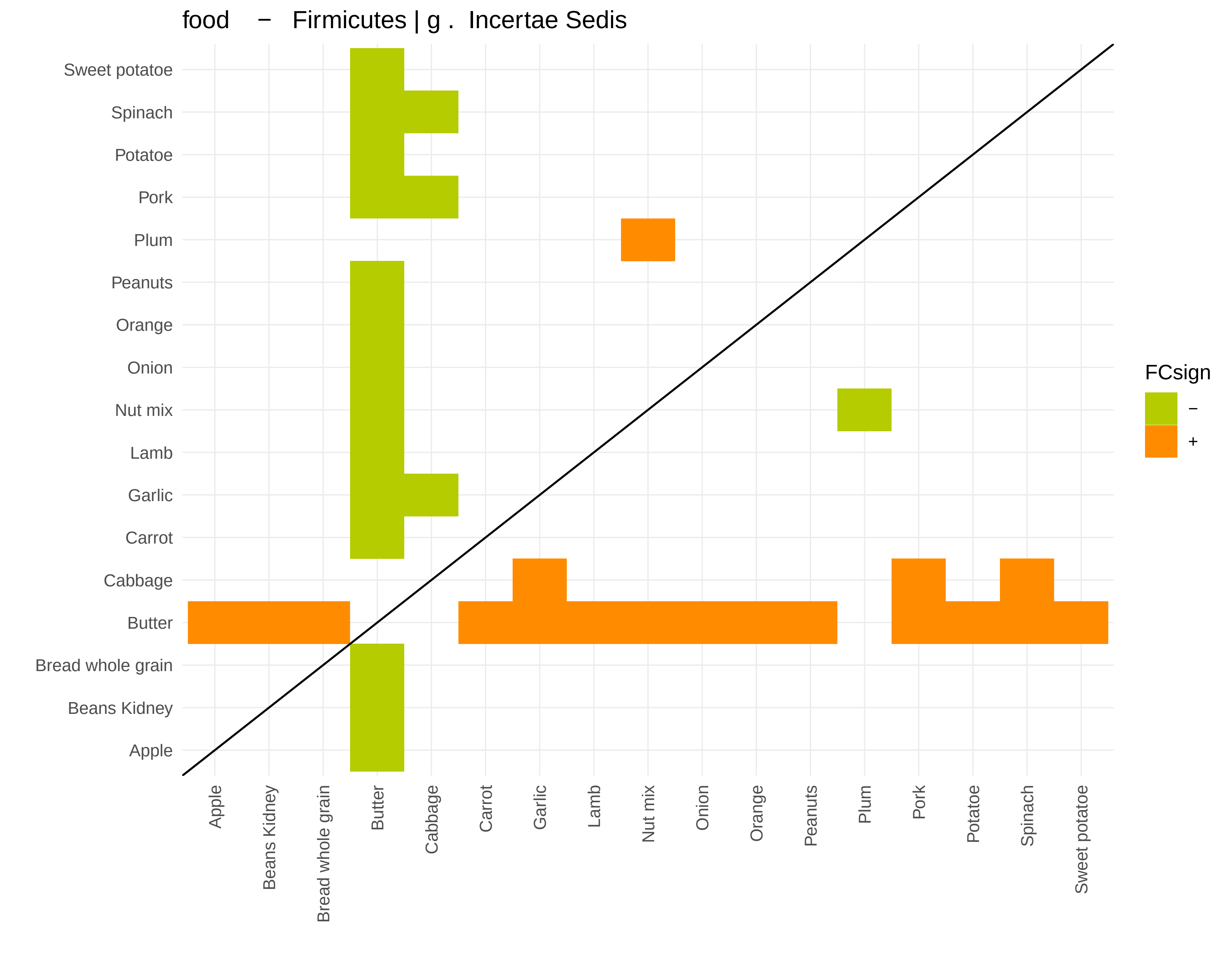
food − Firmicutes | g . Incertae Sedis
Sweet potatoe
Spinach
Potatoe
Pork
Plum
Peanuts
Orange
Onion
Nut mix
Lamb
Garlic
Carrot
Cabbage
Butter
Bread whole grain
Beans Kidney
Apple
Apple
Beans Kidney
Bread whole grain
Butter
Cabbage
Carrot
Garlic
Lamb
Nut mix
Onion
Orange
Peanuts
Plum
Pork
Potatoe
Spinach
Sweet potatoe
FCsign
−
+

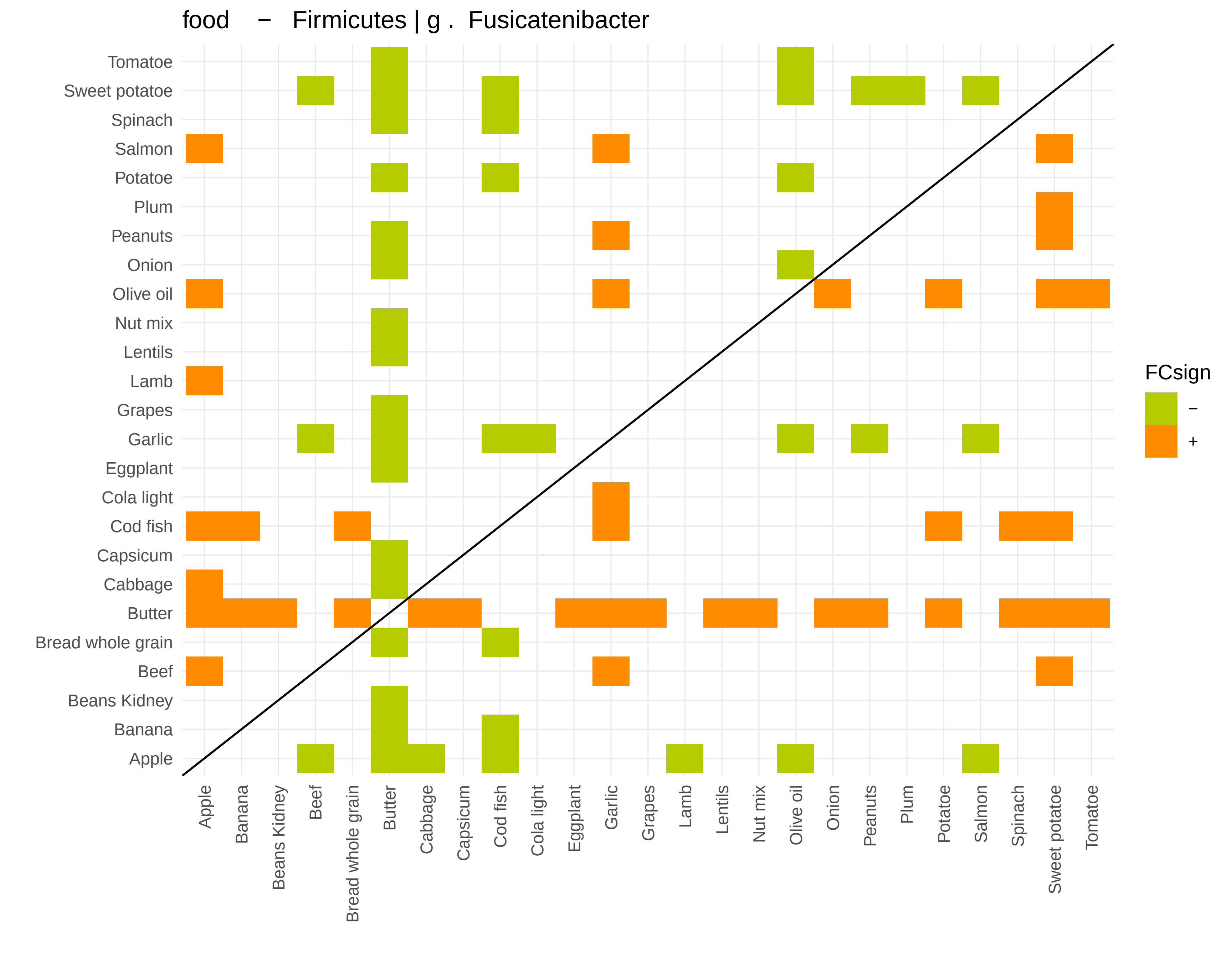
food − Firmicutes | g . Fusicatenibacter
Tomatoe
Sweet potatoe
Spinach
Salmon
Potatoe
Plum
Peanuts
Onion
Olive oil
Nut mix
Lentils
Lamb
Grapes
Garlic
Eggplant
Cola light
Cod fish
Capsicum
Cabbage
Butter
Bread whole grain
Beef
Beans Kidney
Banana
Apple
FCsign
−
+

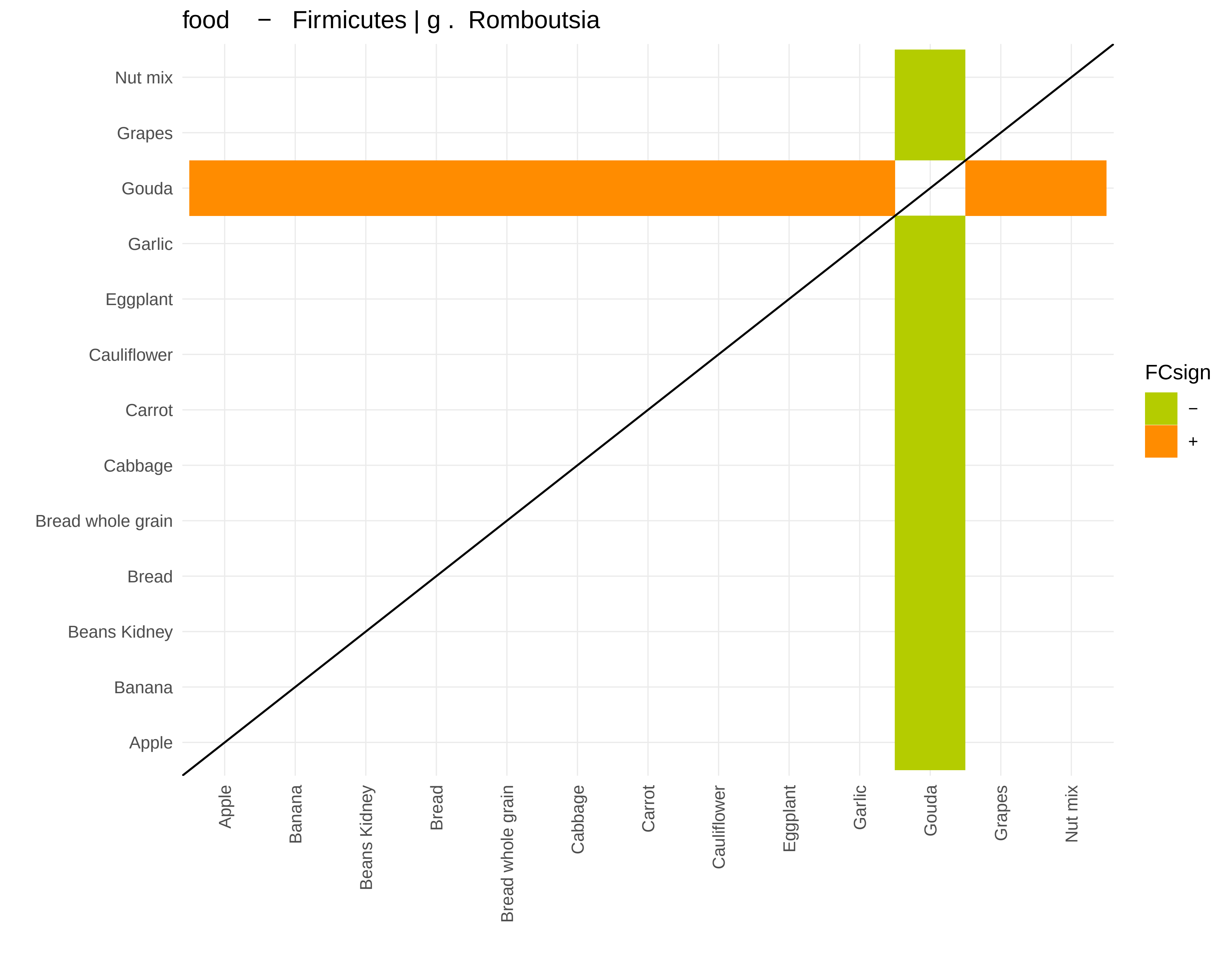

food − Firmicutes | g . Romboutsia
Nut mix
Grapes
Gouda
Garlic
Eggplant
Cauliflower
Carrot
Cabbage
Bread whole grain
Bread
Beans Kidney
Banana
Apple
FCsign
−
+

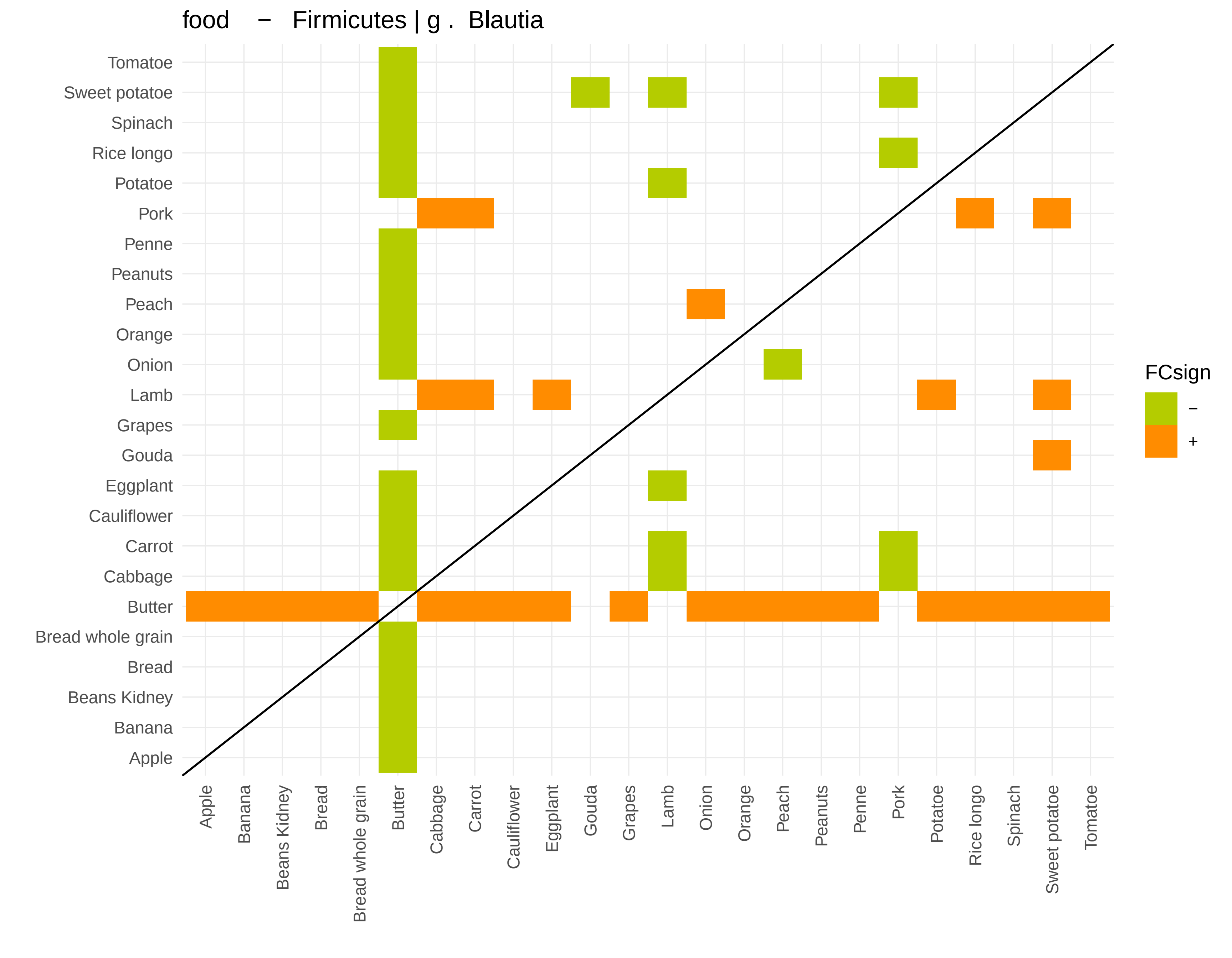
food − Firmicutes | g . Blautia
Tomatoe
Sweet potatoe
Spinach
Rice longo
Potatoe
Pork
Penne
Peanuts
Peach
Orange
Onion
Lamb
Grapes
Gouda
Eggplant
Cauliflower
Carrot
Cabbage
Butter
Bread whole grain
Bread
Beans Kidney
Banana
Apple
FCsign
−
+

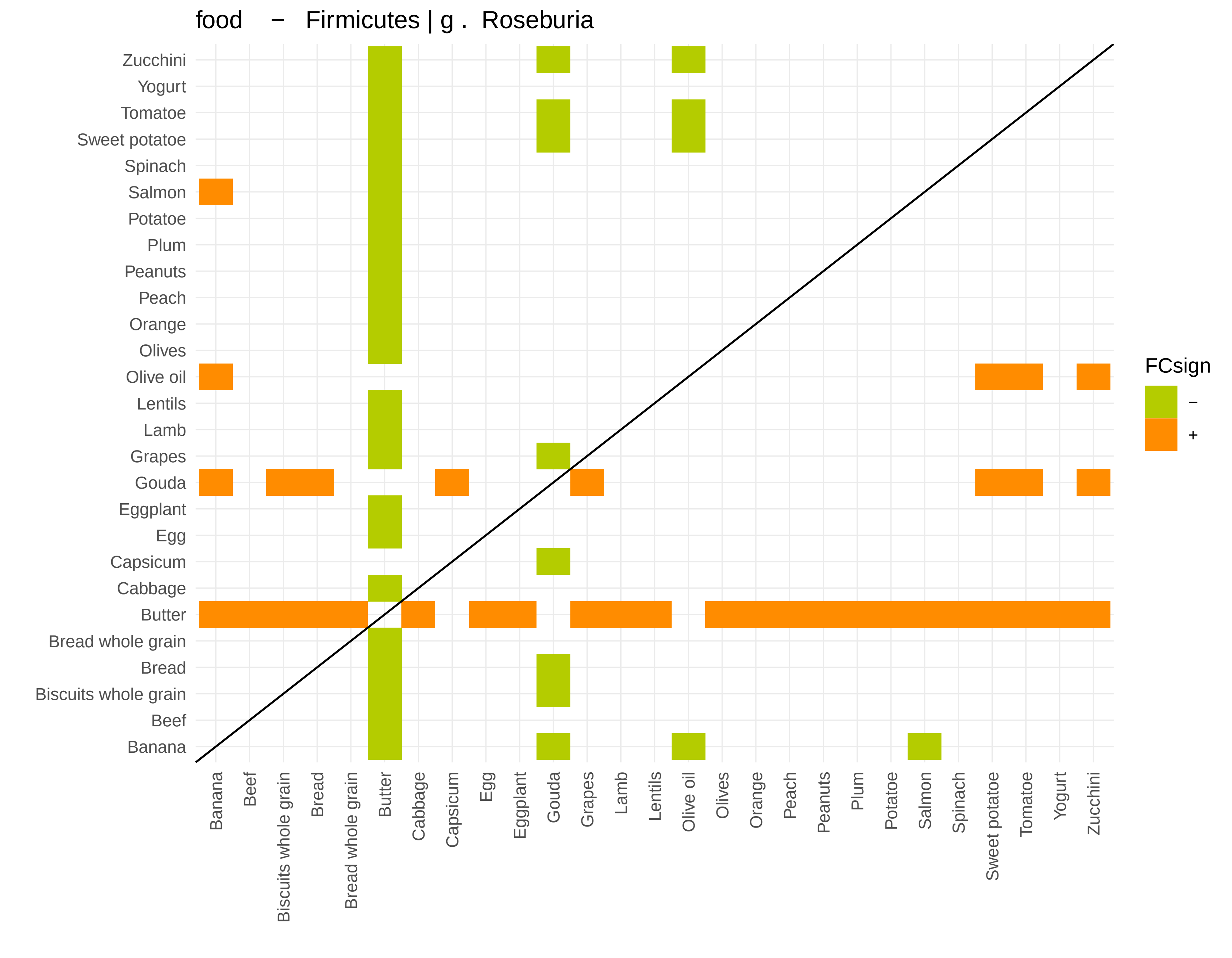
food − Firmicutes | g . Roseburia
Zucchini
Yogurt
Tomatoe
Sweet potatoe
Spinach
Salmon
Potatoe
Plum
Peanuts
Peach
Orange
Olives
Olive oil
Lentils
Lamb
Grapes
Gouda
Eggplant
Egg
Capsicum
Cabbage
Butter
Bread whole grain
Bread
Biscuits whole grain
Beef
Banana
FCsign
−
+

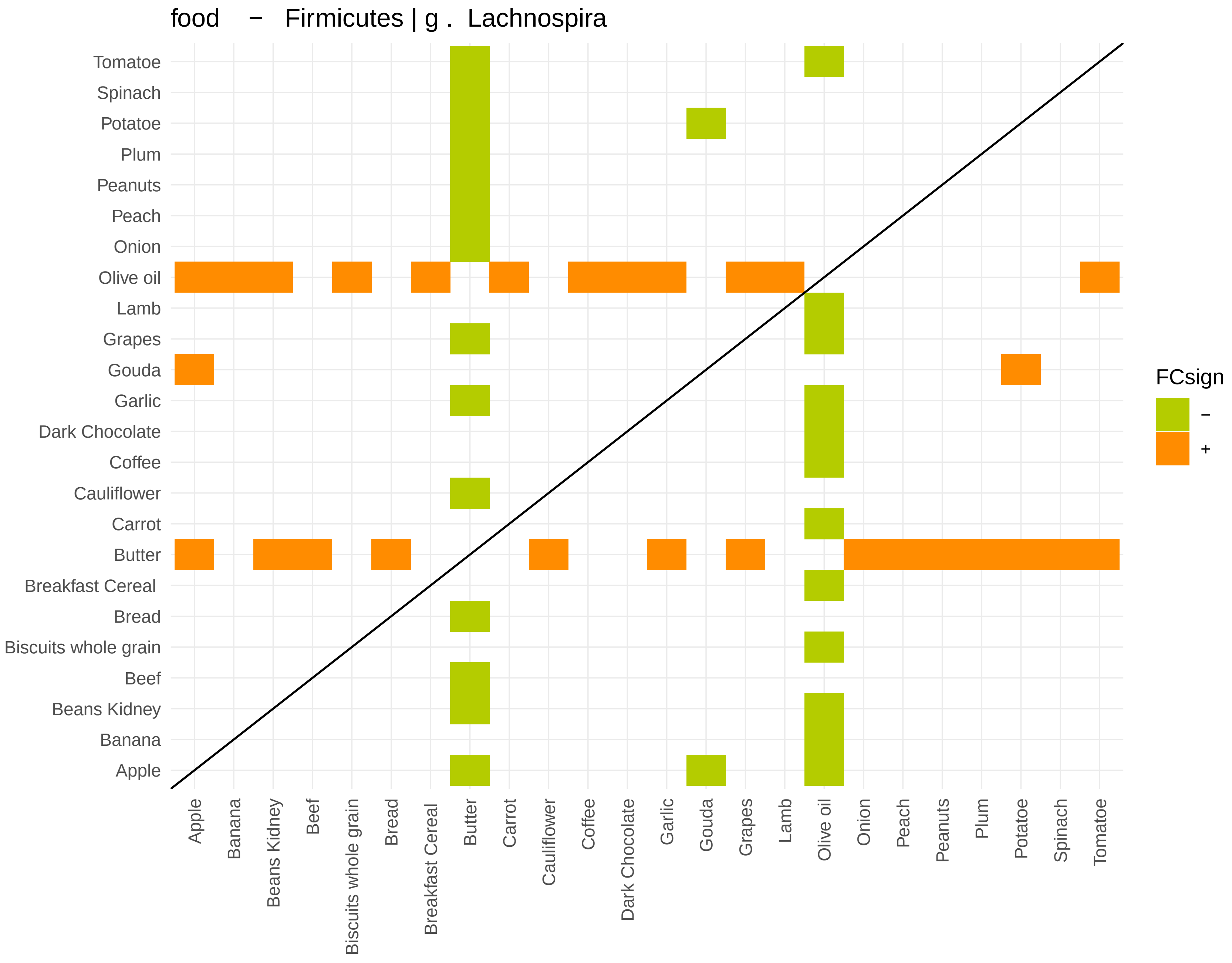
food − Firmicutes | g . Lachnospira
Tomatoe
Spinach
Potatoe
Plum
Peanuts
Peach
Onion
Olive oil
Lamb
Grapes
Gouda
Garlic
Dark Chocolate
Coffee
Cauliflower
Carrot
Butter
Breakfast Cereal
Bread
Biscuits whole grain
Beef
Beans Kidney
Banana
Apple
FCsign
−
+

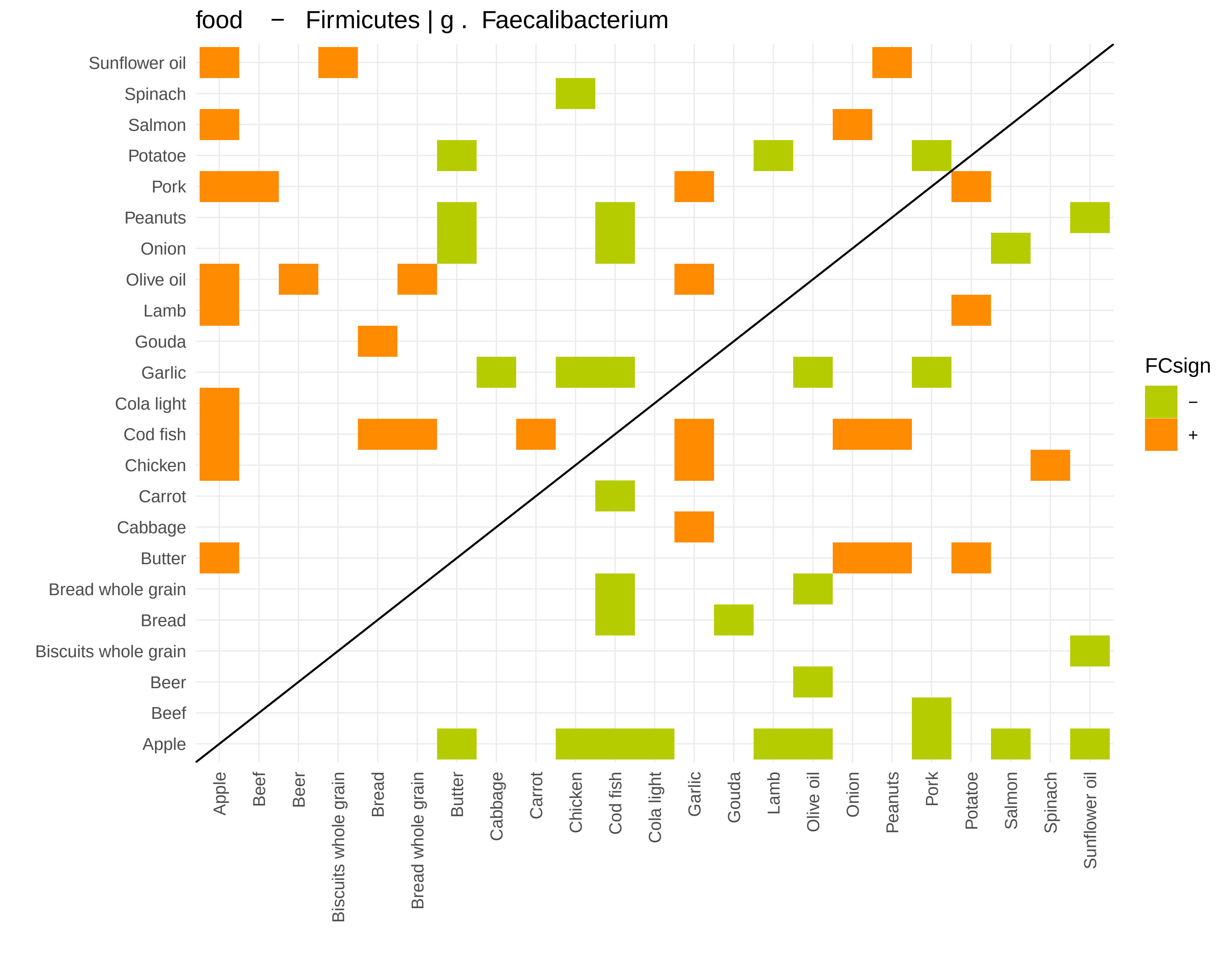

food − Firmicutes | g . Faecalibacterium
Sunflower oil
Spinach
Salmon
Potatoe
Pork
Peanuts
Onion
Olive oil
Lamb
Gouda
Garlic
Cola light
Cod fish
Chicken
Carrot
Cabbage
Butter
Bread whole grain
Bread
Biscuits whole grain
Beer
Beef
Apple
FCsign
−
+

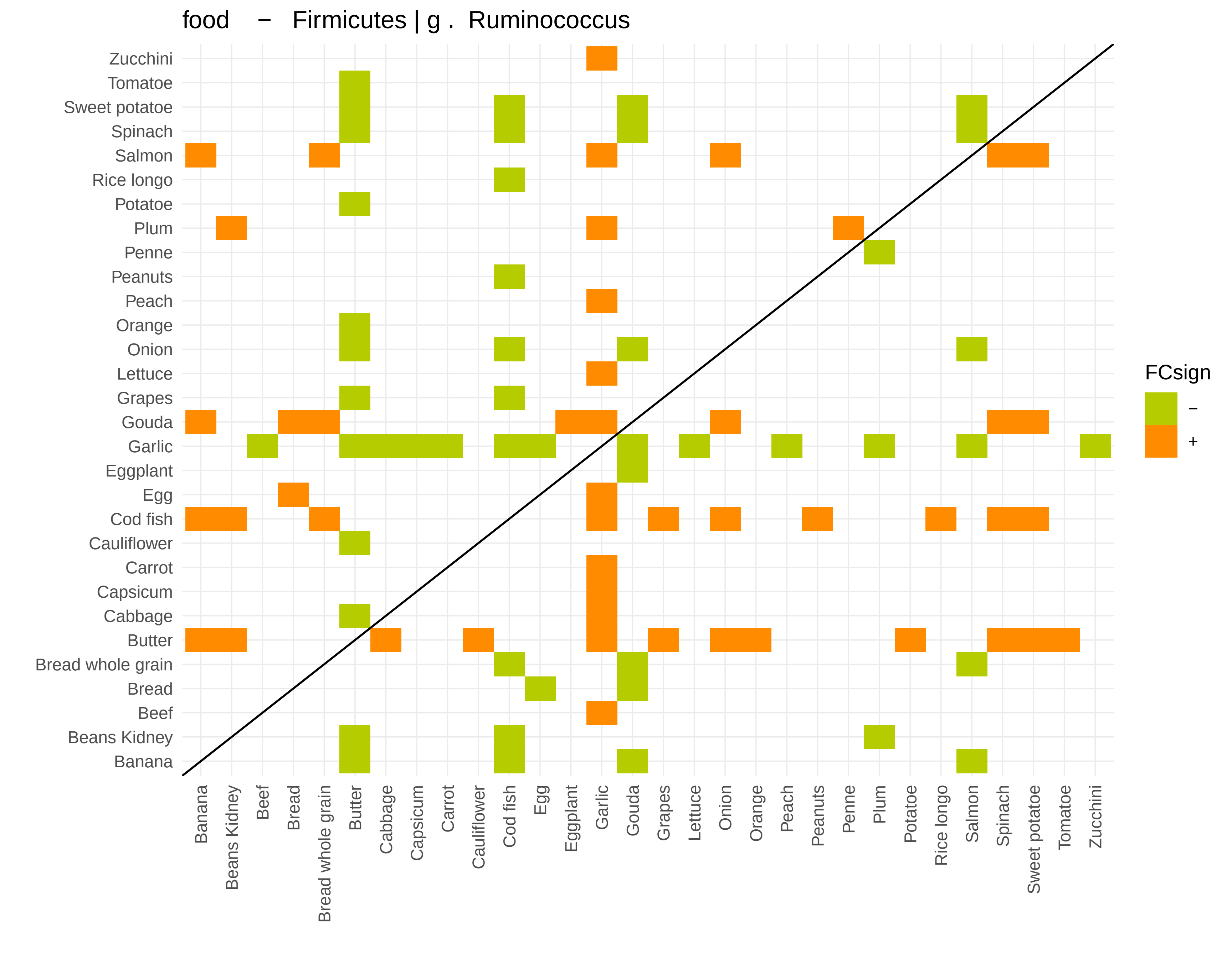
food − Firmicutes | g . Ruminococcus
Zucchini
Tomatoe
Sweet potatoe
Spinach
Salmon
Rice longo
Potatoe
Plum
Penne
Peanuts
Peach
Orange
Onion
Lettuce
Grapes
Gouda
Garlic
Eggplant
Egg
Cod fish
Cauliflower
Carrot
Capsicum
Cabbage
Butter
Bread whole grain
Bread
Beef
Beans Kidney
Banana
FCsign
−
+

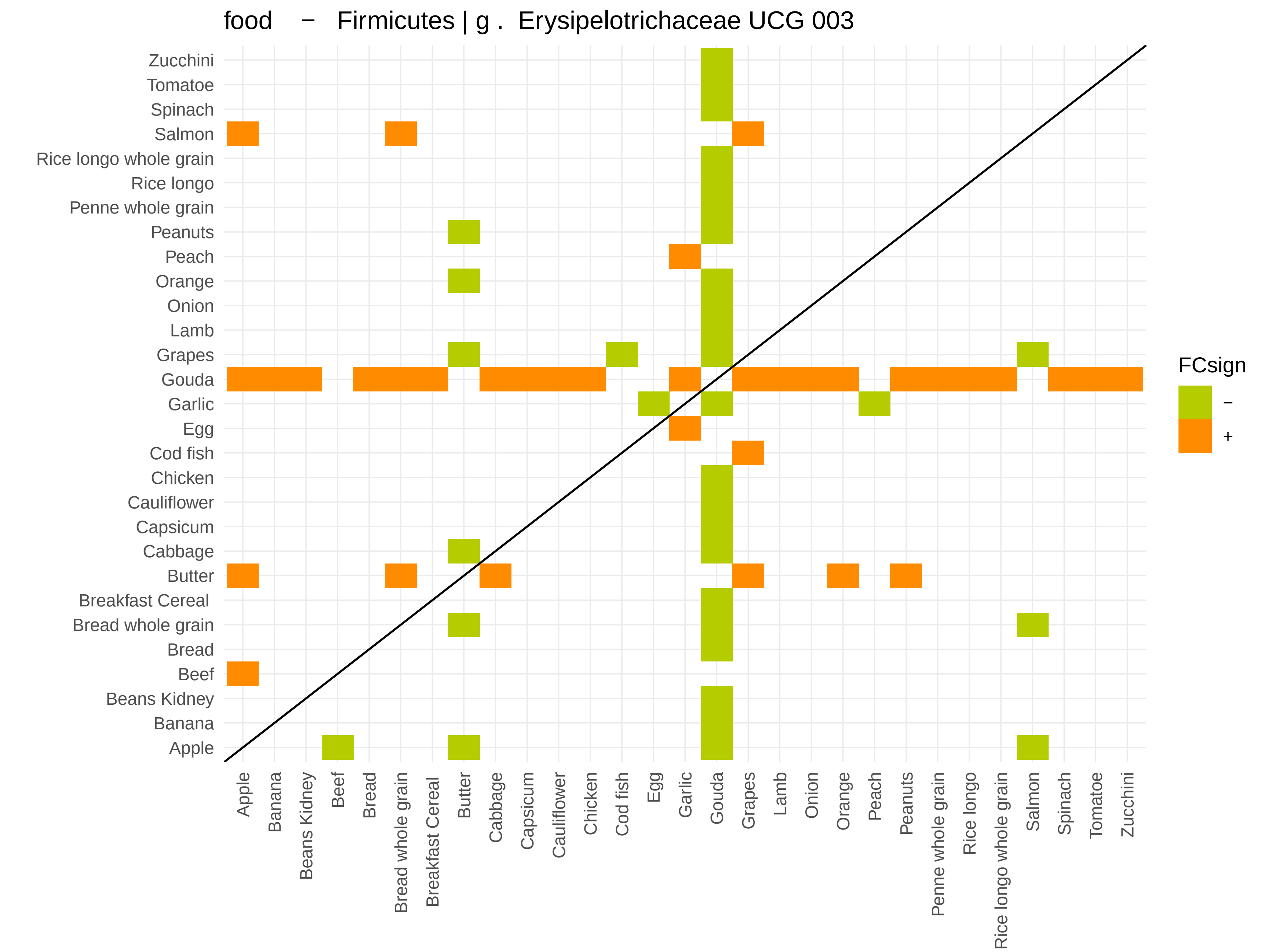

food − Firmicutes | g . Erysipelotrichaceae UCG 003
Zucchini
Tomatoe
Spinach
Salmon
Rice longo whole grain
Rice longo
Penne whole grain
Peanuts
Peach
Orange
Onion
Lamb
Grapes
Gouda
Garlic
Egg
Cod fish
Chicken
Cauliflower
Capsicum
Cabbage
Butter
Breakfast Cereal
Bread whole grain
Bread
Beef
Beans Kidney
Banana
Apple
FCsign
−
+

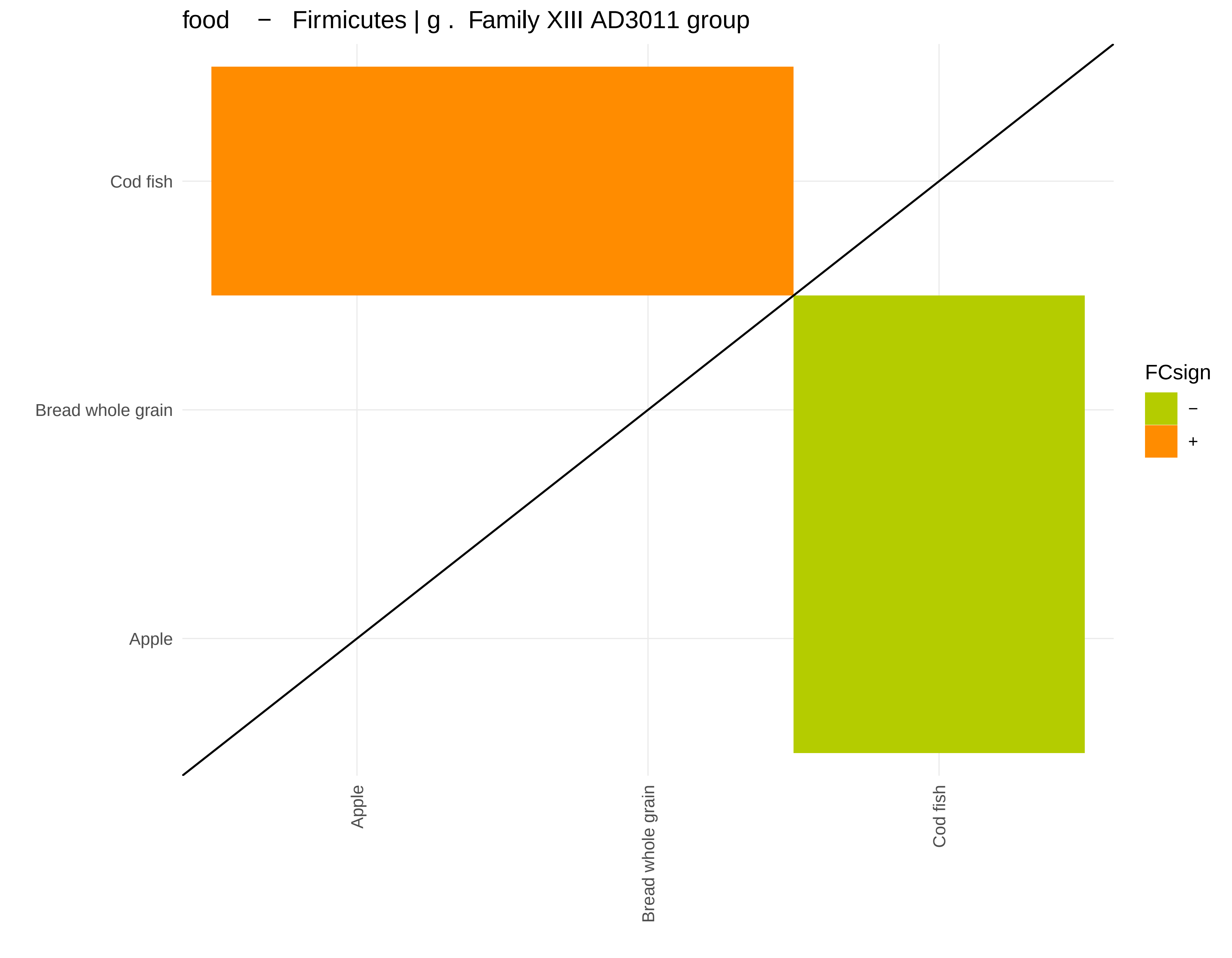
food − Firmicutes | g . Family XIII AD3011 group
Cod fish
Bread whole grain
Apple
Apple
Bread whole grain
Cod fish
FCsign
−
+

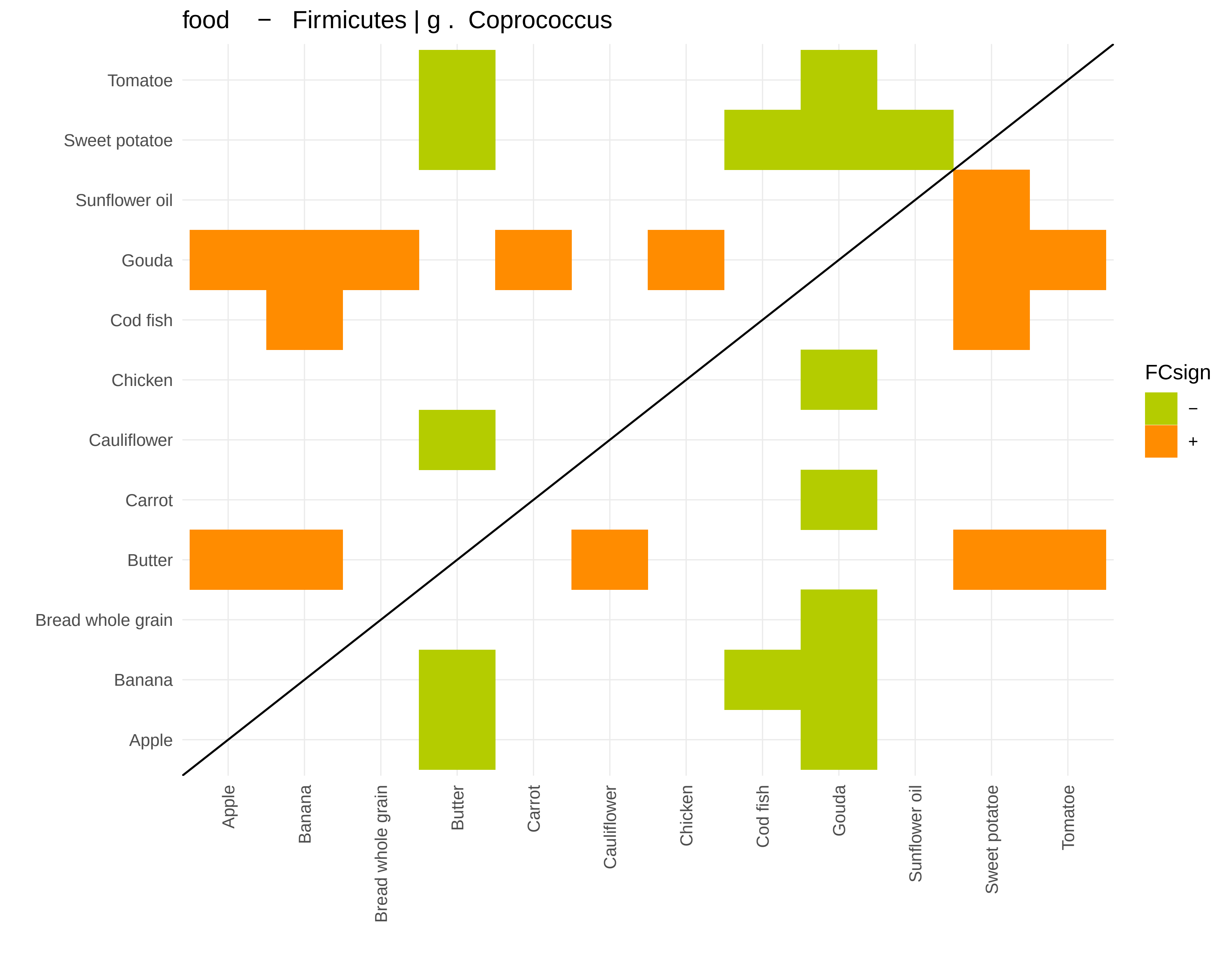
food − Firmicutes | g . Coprococcus
Tomatoe
Sweet potatoe
Sunflower oil
Gouda
Cod fish
Chicken
Cauliflower
Carrot
Butter
Bread whole grain
Banana
Apple
FCsign
−
+

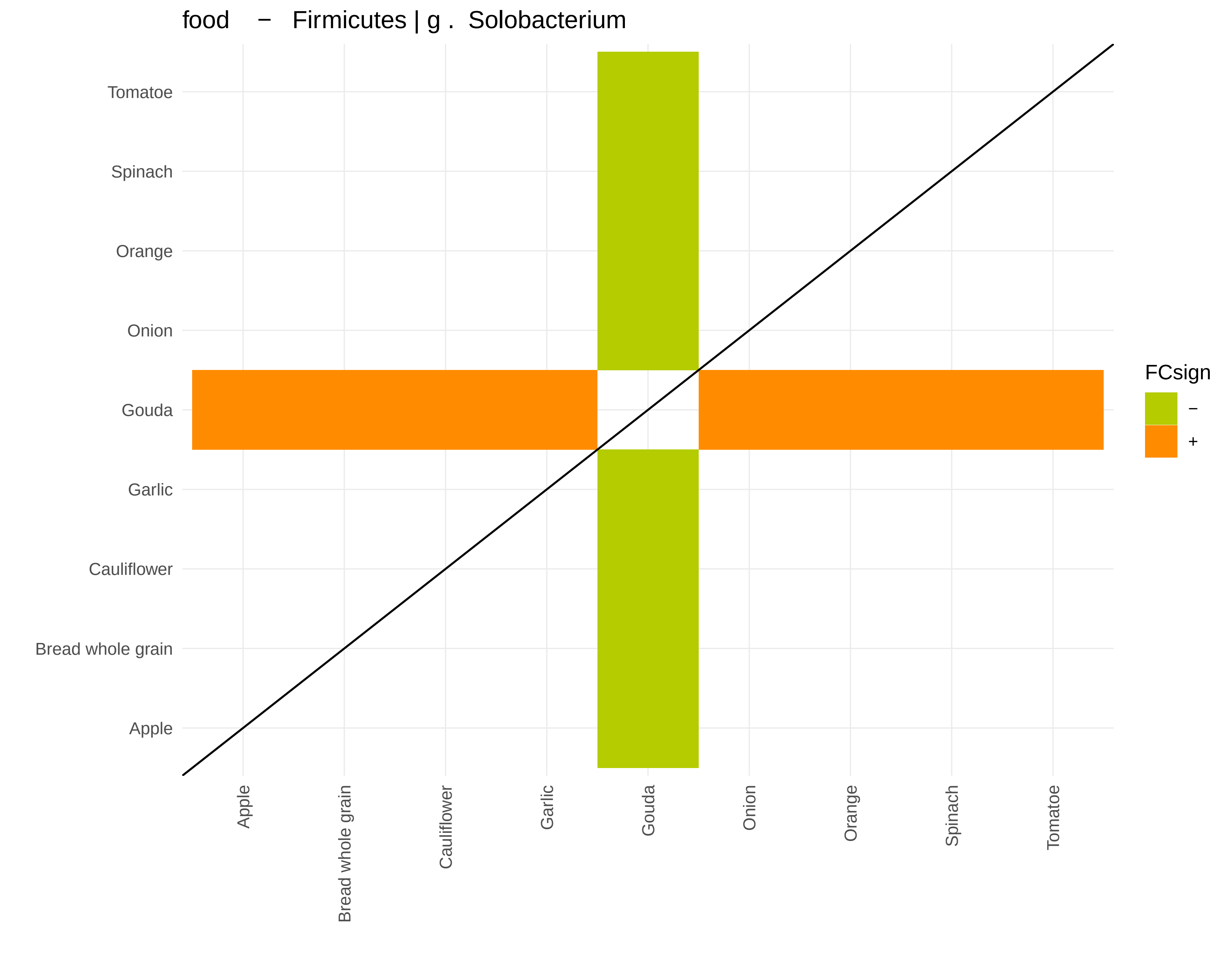

food − Firmicutes | g . Solobacterium
Tomatoe
Spinach
Orange
Onion
Gouda
Garlic
Cauliflower
Bread whole grain
Apple
Apple
Bread whole grain
Cauliflower
Garlic
Gouda
Onion
Orange
Spinach
Tomatoe
FCsign
−
+

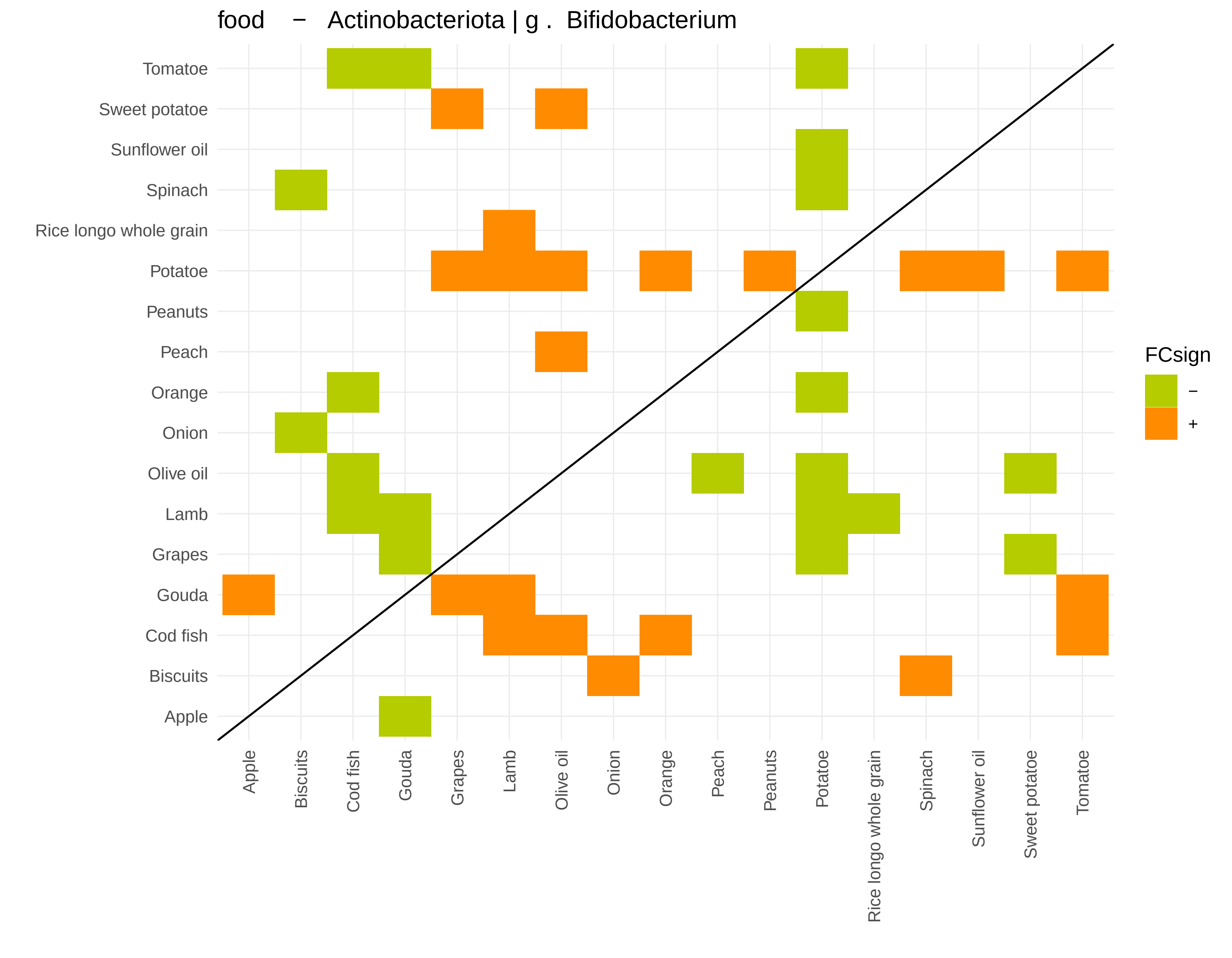
food − Actinobacteriota | g . Bifidobacterium
Tomatoe
Sweet potatoe
Sunflower oil
Spinach
Rice longo whole grain
Potatoe
Peanuts
Peach
Orange
Onion
Olive oil
Lamb
Grapes
Gouda
Cod fish
Biscuits
Apple
FCsign
−
+

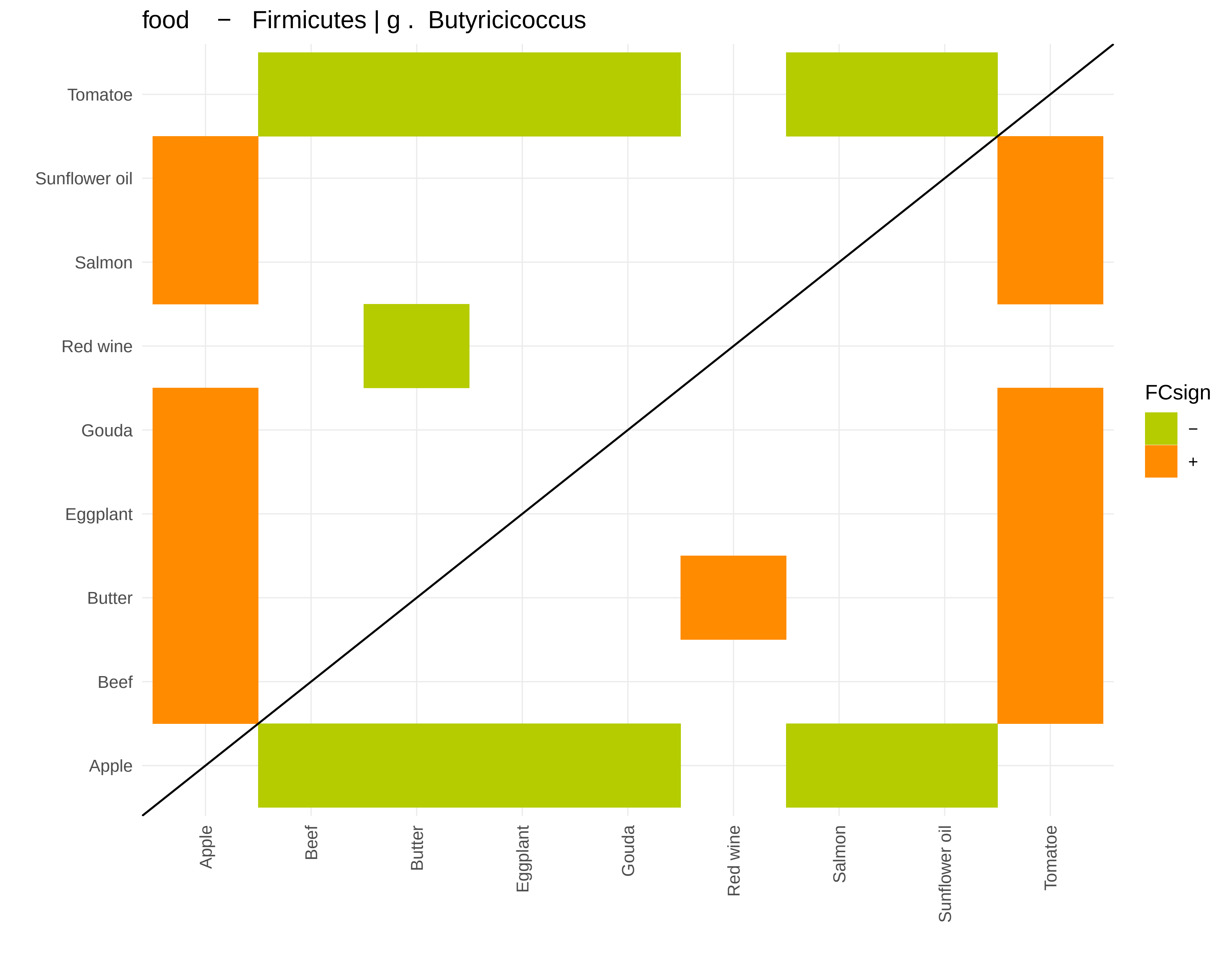
food − Firmicutes | g . Butyricicoccus
Tomatoe
Sunflower oil
Salmon
Red wine
Gouda
Eggplant
Butter
Beef
Apple
Apple
Beef
Butter
Eggplant
Gouda
Red wine
Salmon
Sunflower oil
Tomatoe
FCsign
−
+

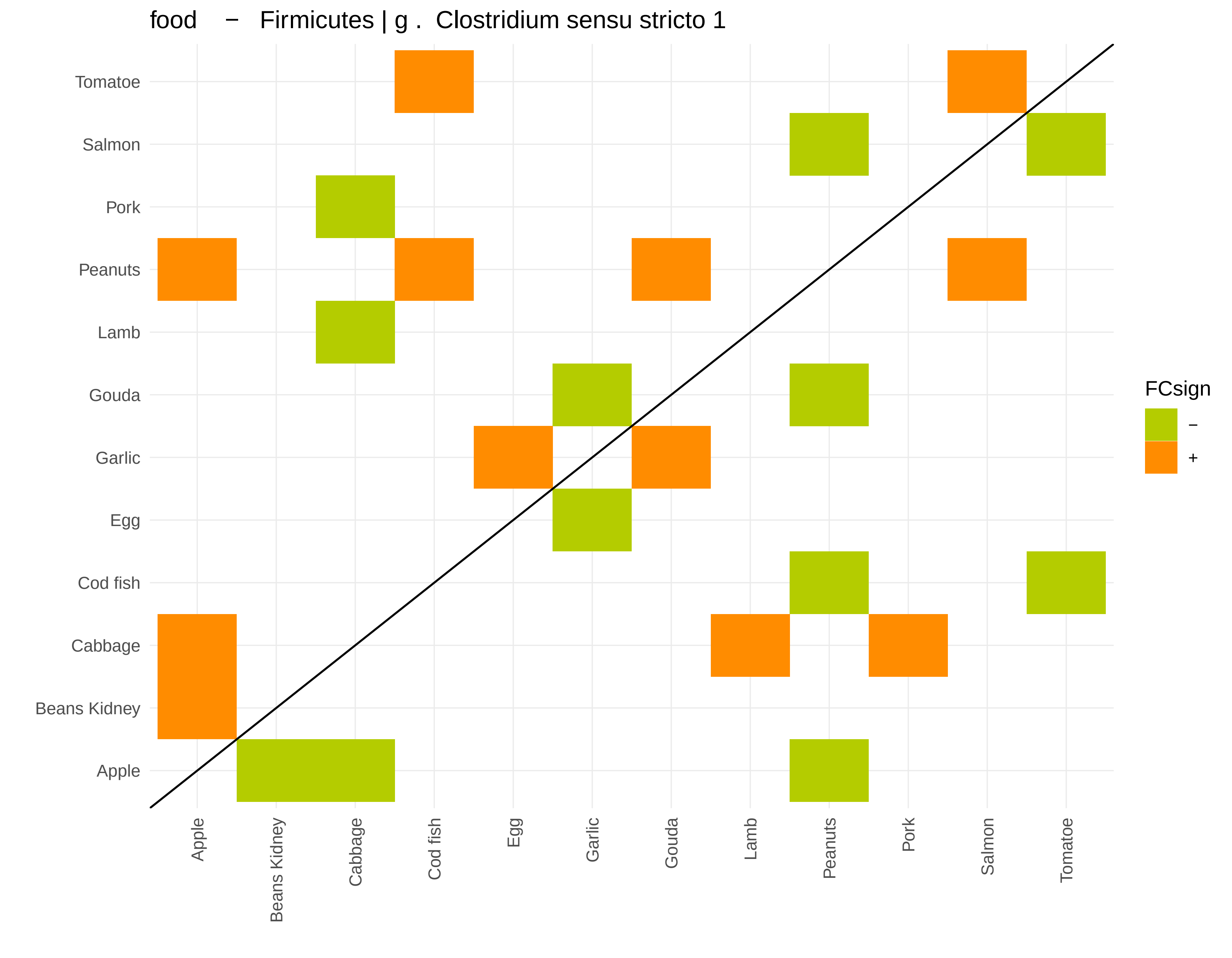

food − Firmicutes | g . Clostridium sensu stricto 1
Tomatoe
Salmon
Pork
Peanuts
Lamb
Gouda
Garlic
Egg
Cod fish
Cabbage
Beans Kidney
Apple
FCsign
−
+

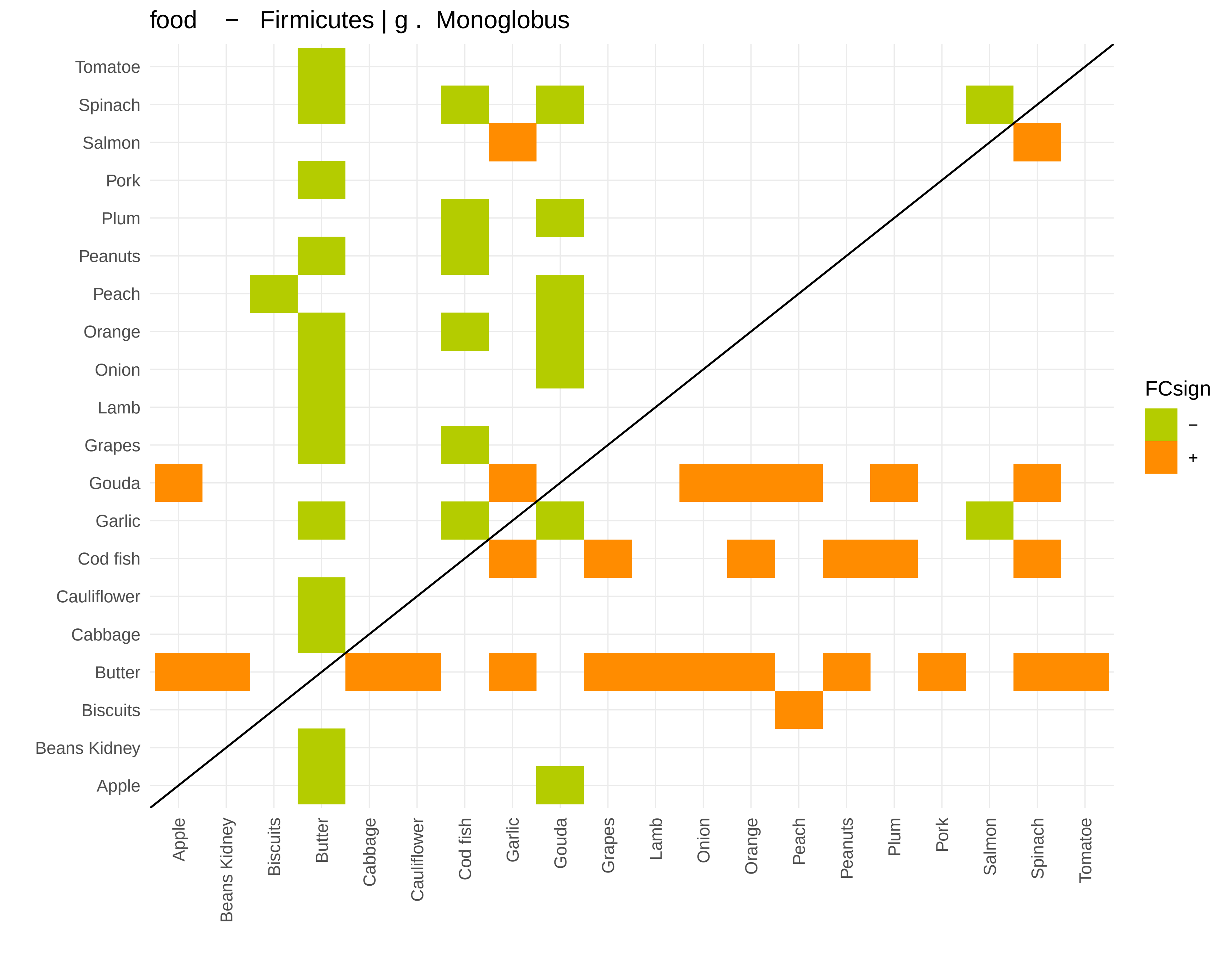
food − Firmicutes | g . Monoglobus
Tomatoe
Spinach
Salmon
Pork
Plum
Peanuts
Peach
Orange
Onion
Lamb
Grapes
Gouda
Garlic
Cod fish
Cauliflower
Cabbage
Butter
Biscuits
Beans Kidney
Apple
FCsign
−
+

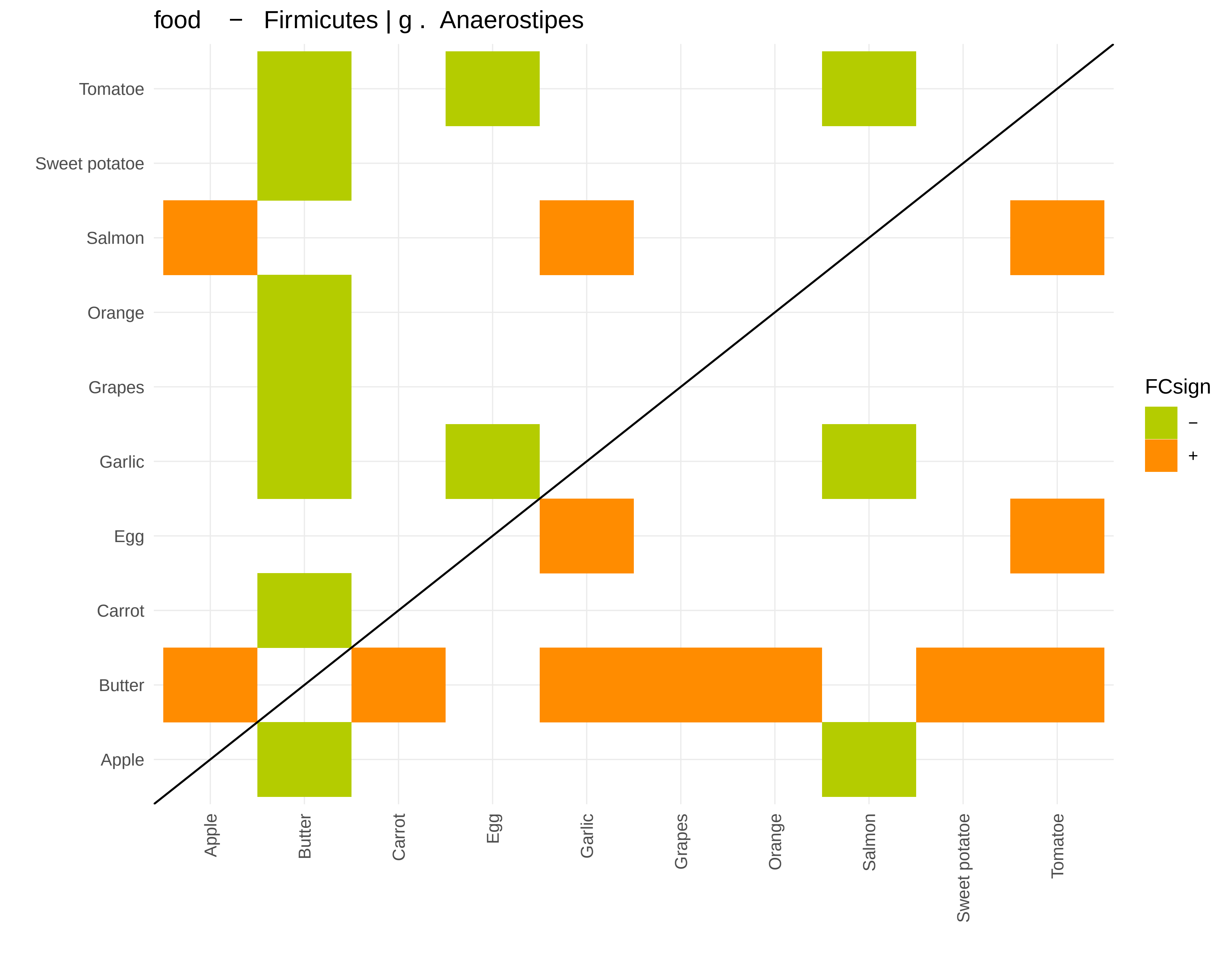
food − Firmicutes | g . Anaerostipes
Tomatoe
Sweet potatoe
Salmon
Orange
Grapes
Garlic
Egg
Carrot
Butter
Apple
Apple
Butter
Carrot
Egg
Garlic
Grapes
Orange
Salmon
Sweet potatoe
Tomatoe
FCsign
−
+

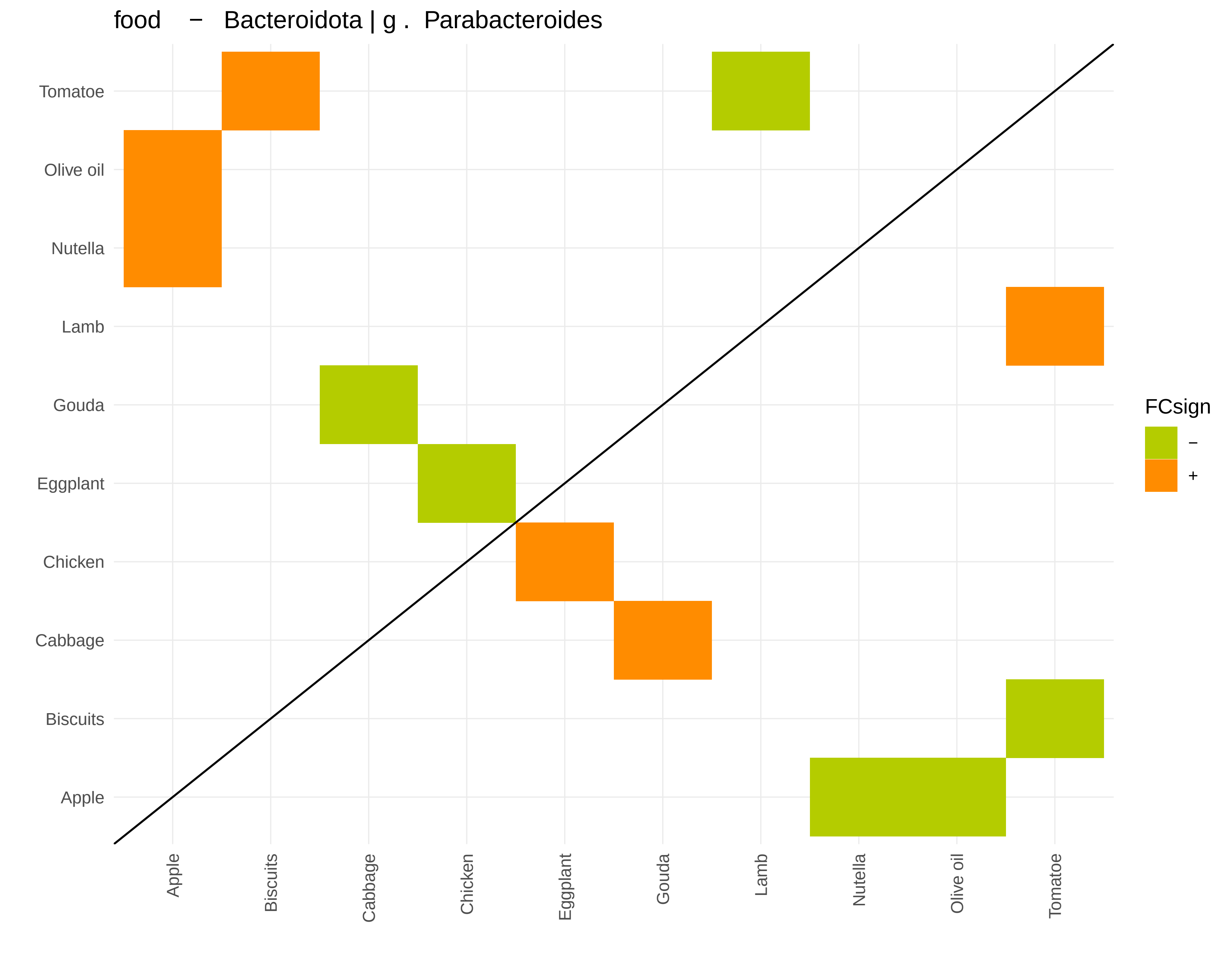

food − Bacteroidota | g . Parabacteroides
Tomatoe
Olive oil
Nutella
Lamb
Gouda
Eggplant
Chicken
Cabbage
Biscuits
Apple
Apple
Biscuits
Cabbage
Chicken
Eggplant
Gouda
Lamb
Nutella
Olive oil
Tomatoe
FCsign
−
+

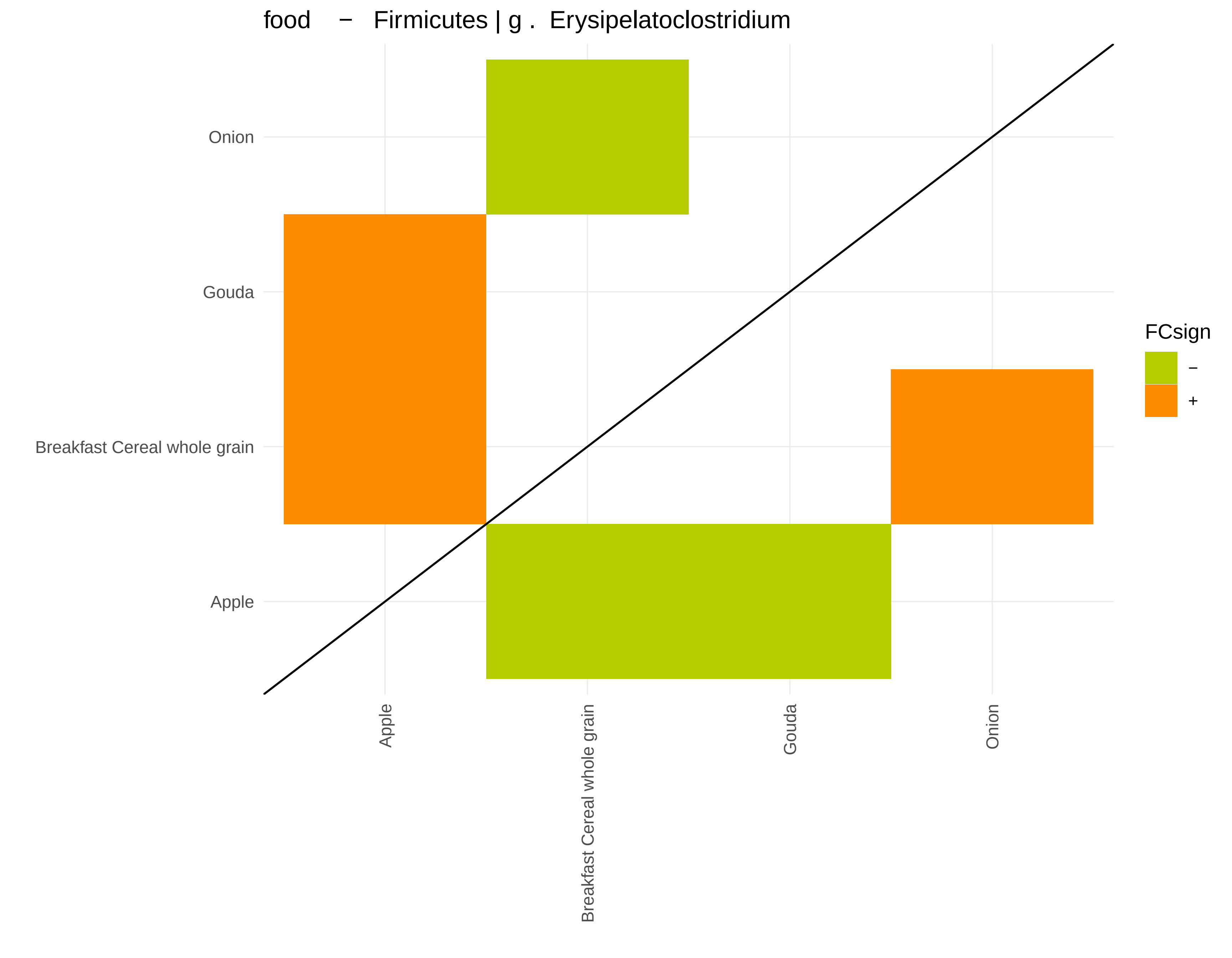

food − Firmicutes | g . Erysipelatoclostridium
Onion
Gouda
Breakfast Cereal whole grain
Apple
Apple
Breakfast Cereal whole grain
Gouda
Onion
FCsign
−
+

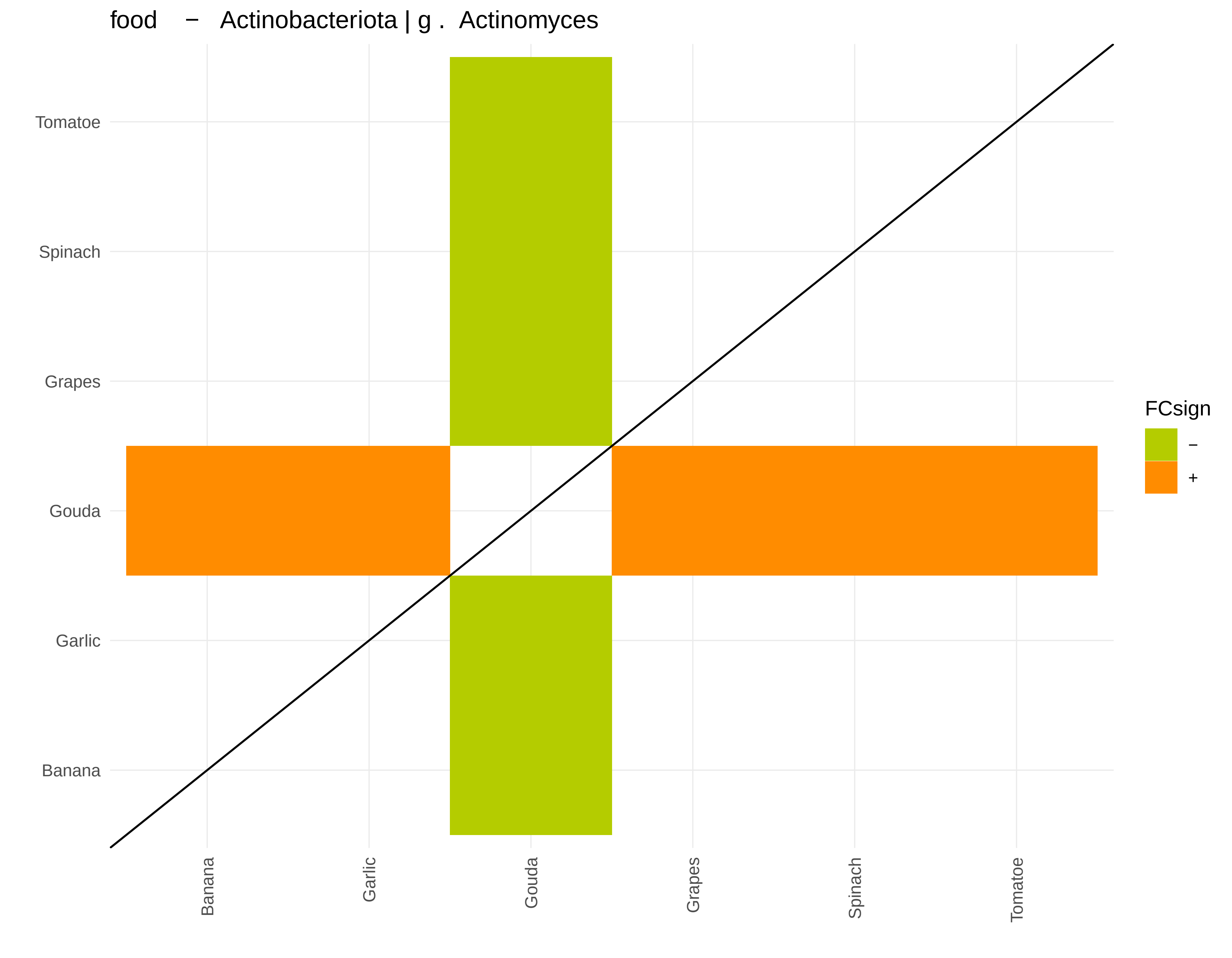
food − Actinobacteriota | g . Actinomyces
Tomatoe
Spinach
Grapes
Gouda
Garlic
Banana
Banana
Garlic
Gouda
Grapes
Spinach
Tomatoe
FCsign
−
+

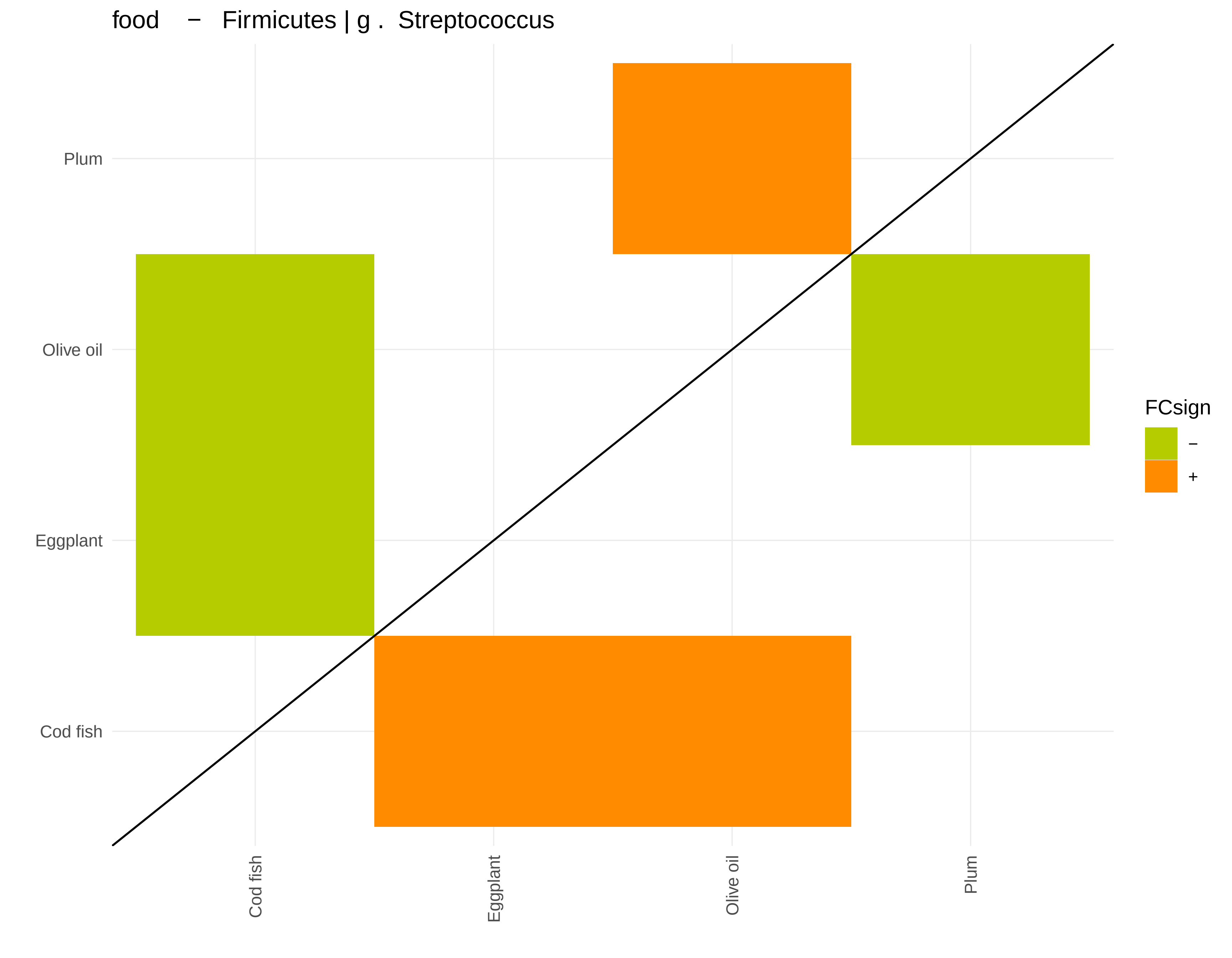

food − Firmicutes | g . Streptococcus
Plum
Olive oil
Eggplant
Cod fish
Cod fish
Eggplant
Olive oil
Plum
FCsign
−
+

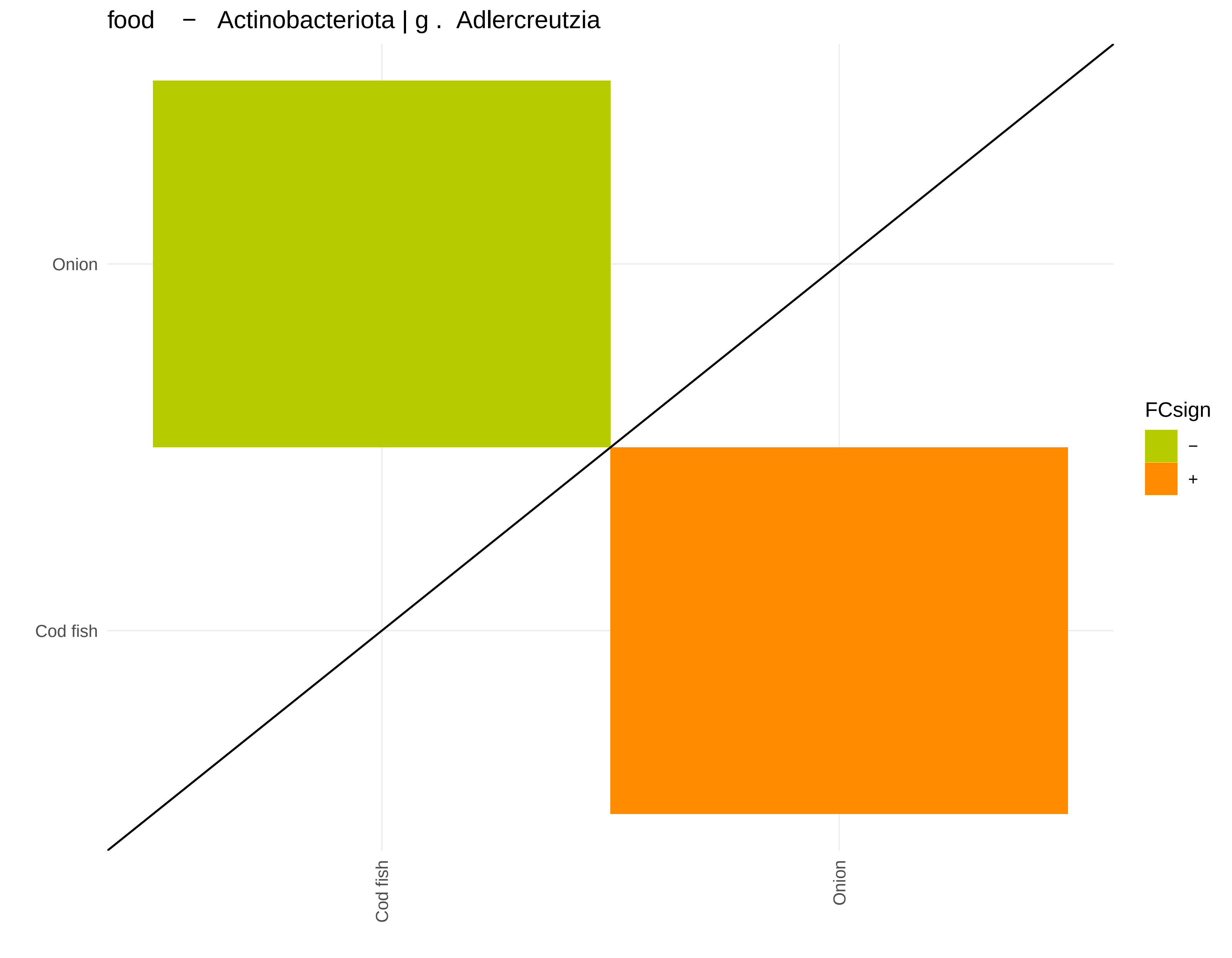
food − Actinobacteriota | g . Adlercreutzia
Onion
Cod fish
Cod fish
Onion
FCsign
−
+

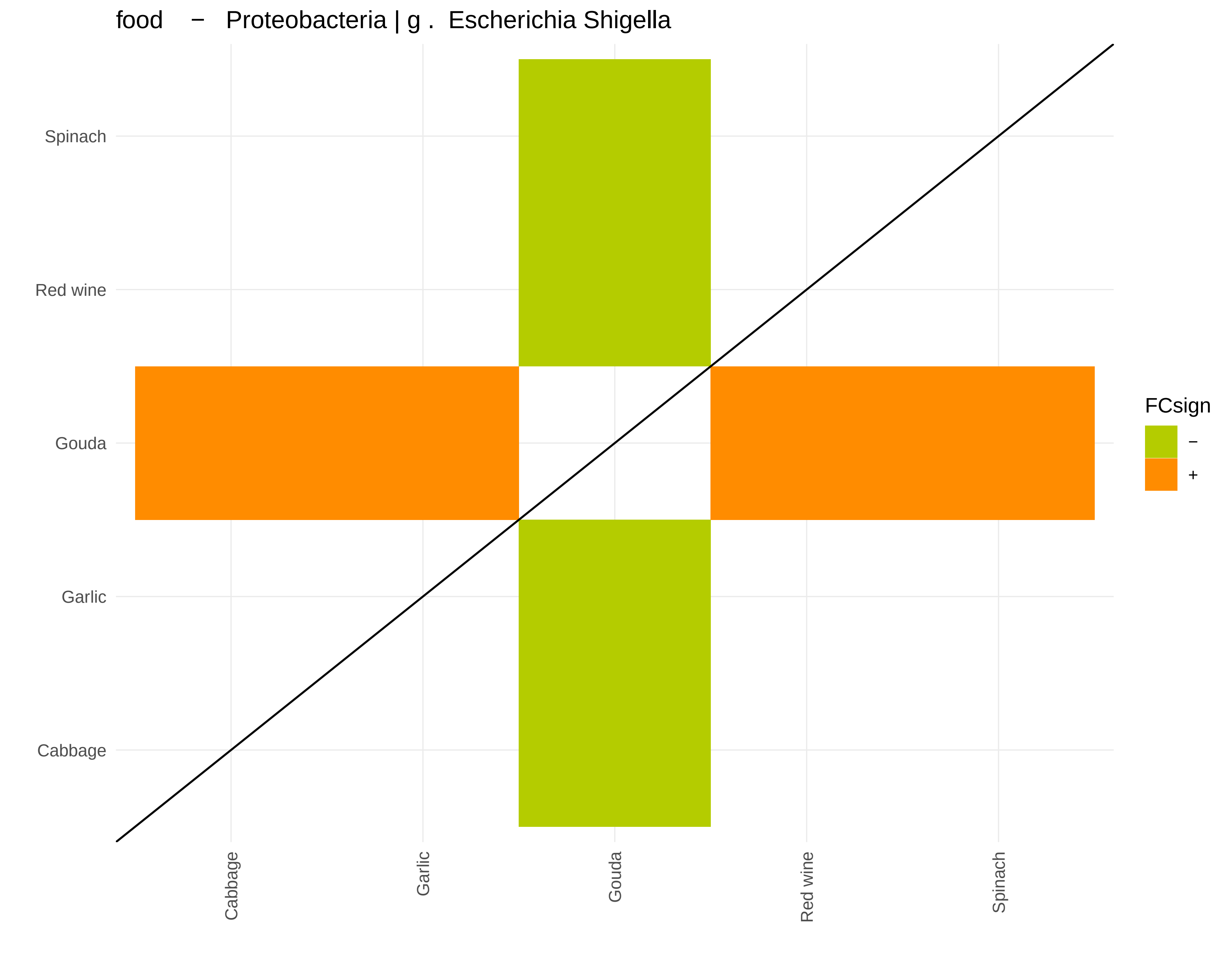
food − Proteobacteria | g . Escherichia Shigella
Spinach
Red wine
Gouda
Garlic
Cabbage
Cabbage
Garlic
Gouda
Red wine
Spinach
FCsign
−
+

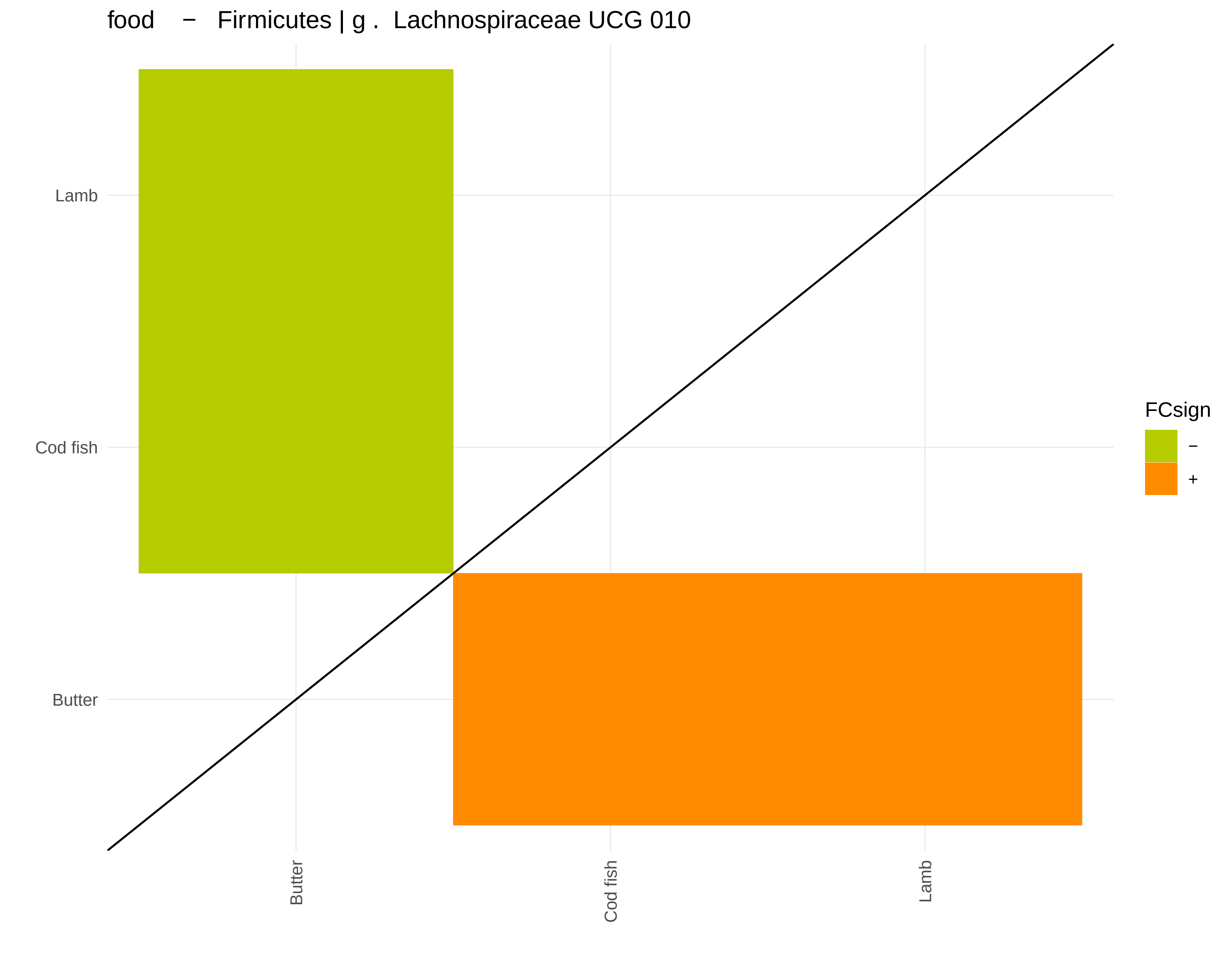

food − Firmicutes | g . Lachnospiraceae UCG 010
Lamb
Cod fish
Butter
Butter
Cod fish
Lamb
FCsign
−
+
