# Supplementary material for: Effects of different foods and cooking methods on the gut microbiota: an in vitro approach
Source: Front Microbiol. 2024 Jan 8;14:1334623. doi: 10.3389/fmicb.2023.1334623 (PMC10800916; doi:10.3389/fmicb.2023.1334623)

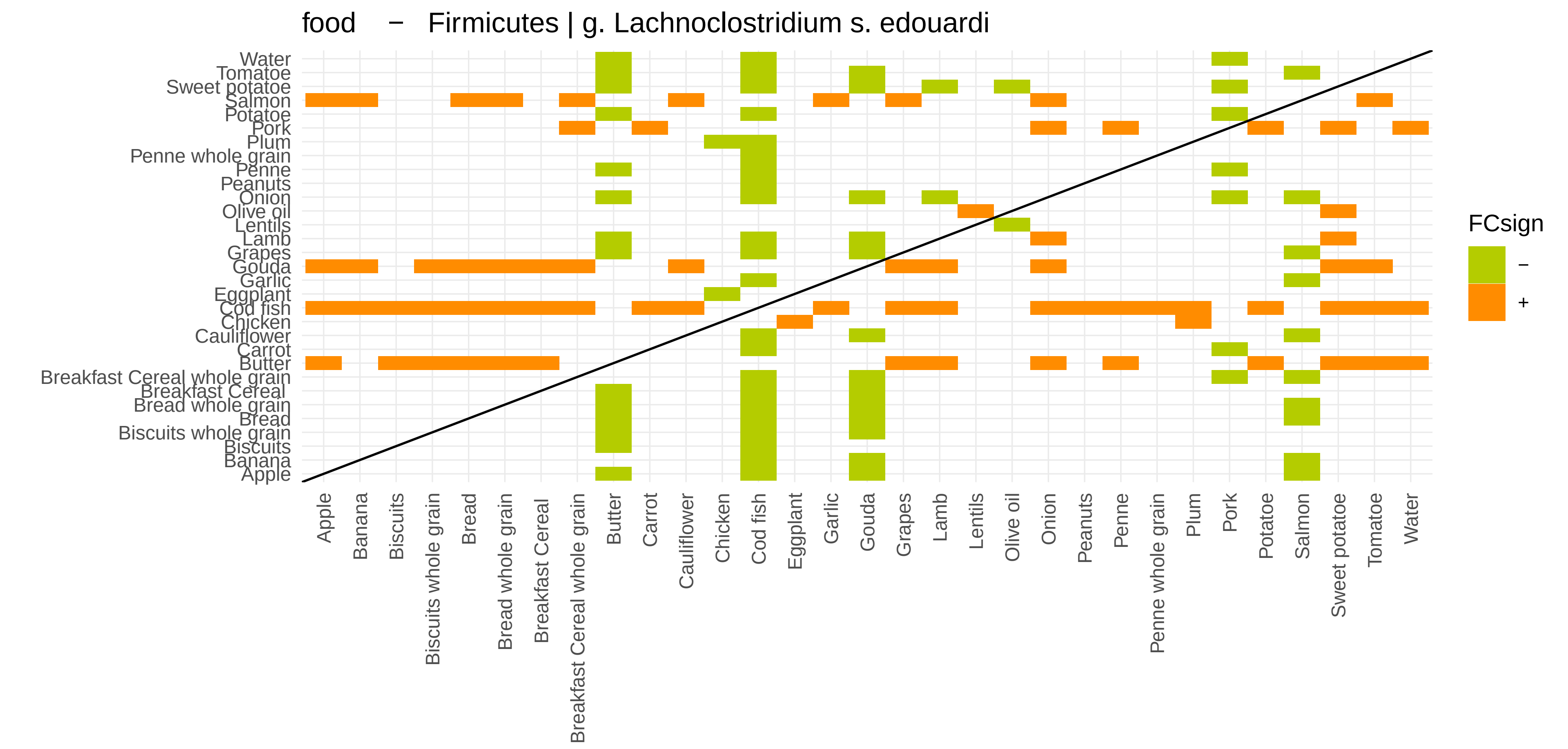

food − Firmicutes | g. Lachnoclostridium s. edouardi
Water
Tomatoe
Sweet potatoe
Salmon
Potatoe
Pork
Plum
Penne whole grain
Penne
Peanuts
Onion
Olive oil
Lentils
Lamb
Grapes
Gouda
Garlic
Eggplant
Cod fish
Chicken
Cauliflower
Carrot
Butter
Breakfast Cereal whole grain
Breakfast Cereal
Bread whole grain
Bread
Biscuits whole grain
Biscuits
Banana
Apple
FCsign
−
+

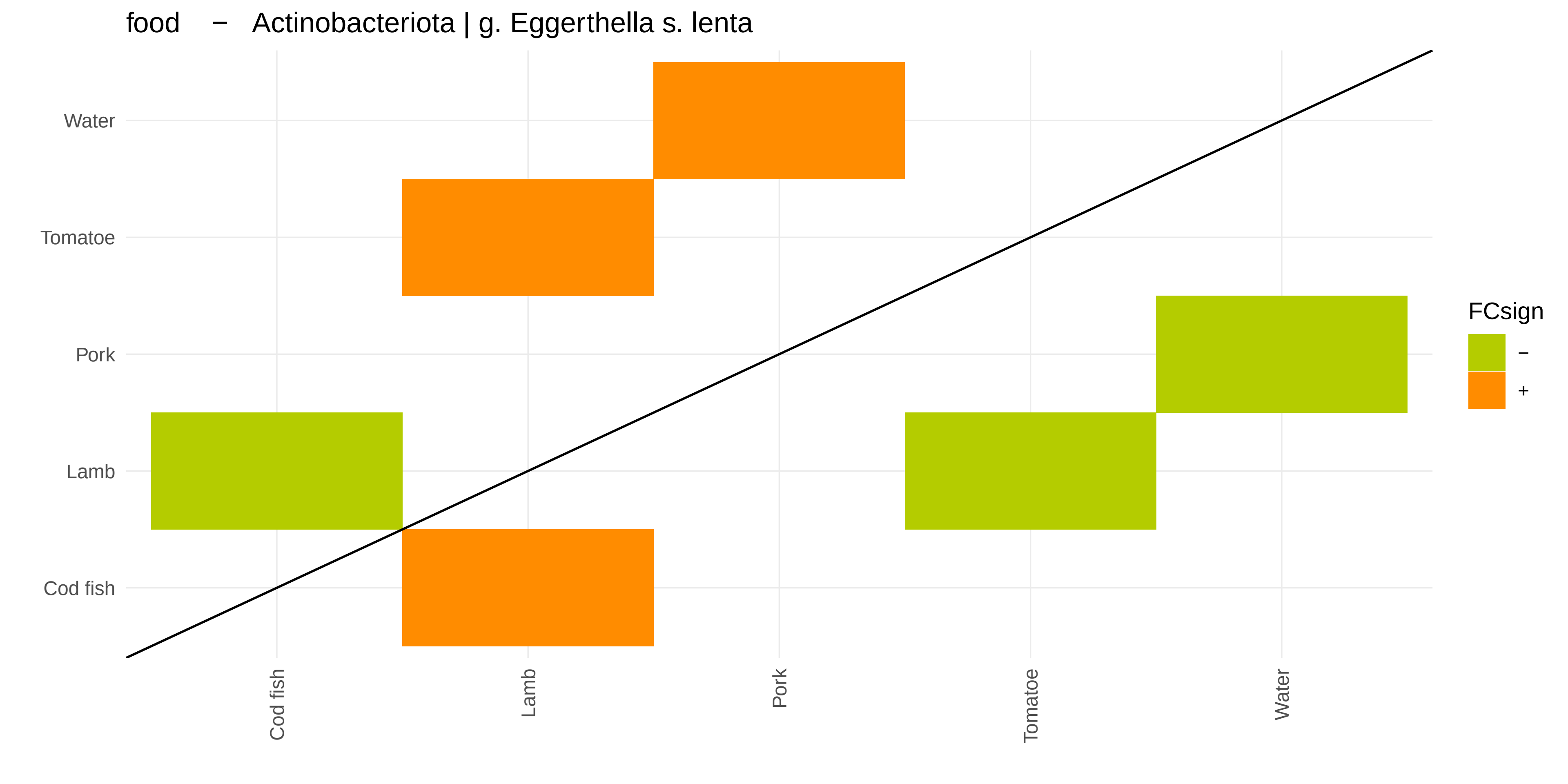
food − Actinobacteriota | g. Eggerthella s. lenta
Water
Tomatoe
Pork
Lamb
Cod fish
Cod fish
Lamb
Pork
Tomatoe
Water
FCsign
−
+

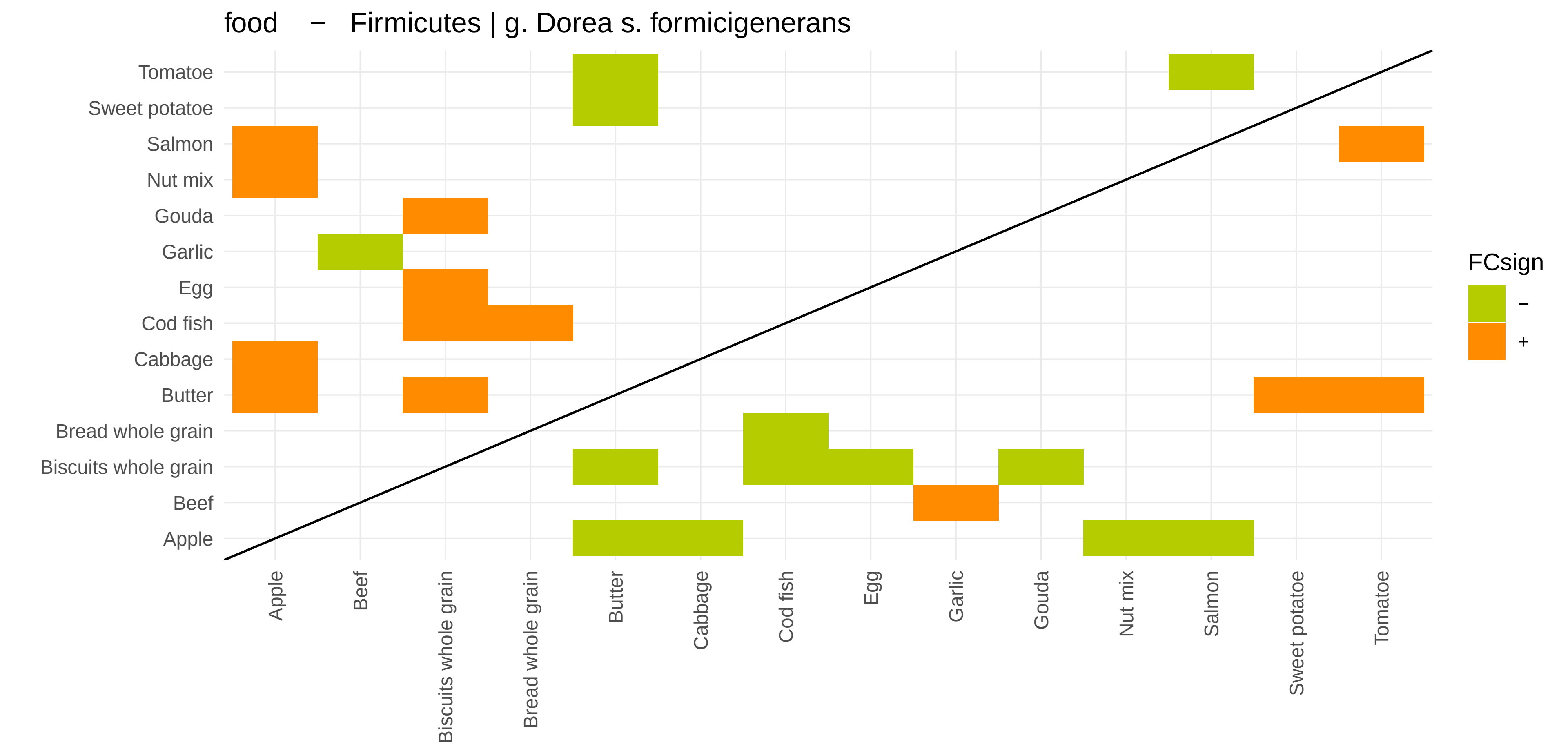
food − Firmicutes | g. Dorea s. formicigenerans
Tomatoe
Sweet potatoe
Salmon
Nut mix
Gouda
Garlic
Egg
Cod fish
Cabbage
Butter
Bread whole grain
Biscuits whole grain
Beef
Apple
FCsign
−
+

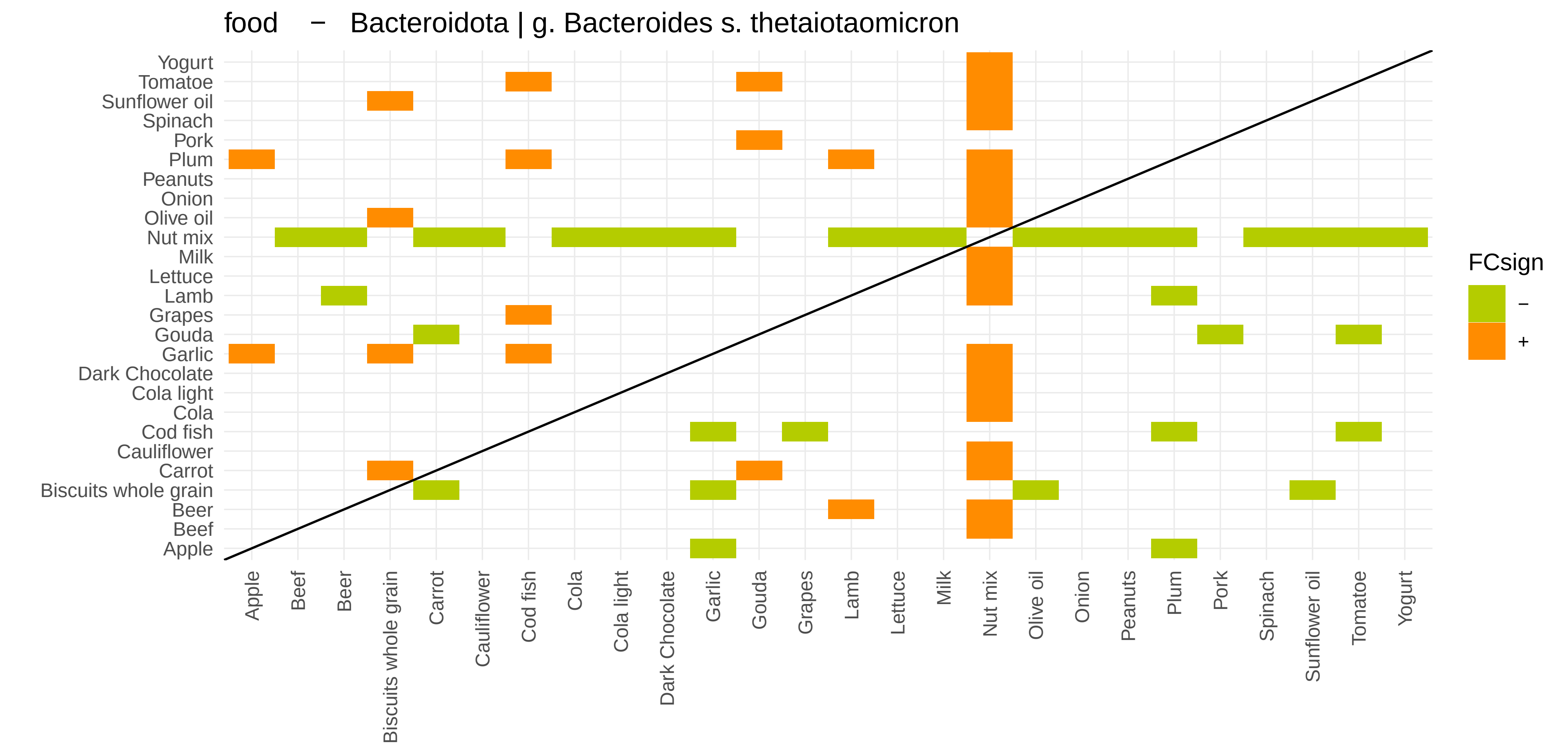
food − Bacteroidota | g. Bacteroides s. thetaiotaomicron
Yogurt
Tomatoe
Sunflower oil
Spinach
Pork
Plum
Peanuts
Onion
Olive oil
Nut mix
Milk
Lettuce
Lamb
Grapes
Gouda
Garlic
Dark Chocolate
Cola light
Cola
Cod fish
Cauliflower
Carrot
Biscuits whole grain
Beer
Beef
Apple
FCsign
−
+

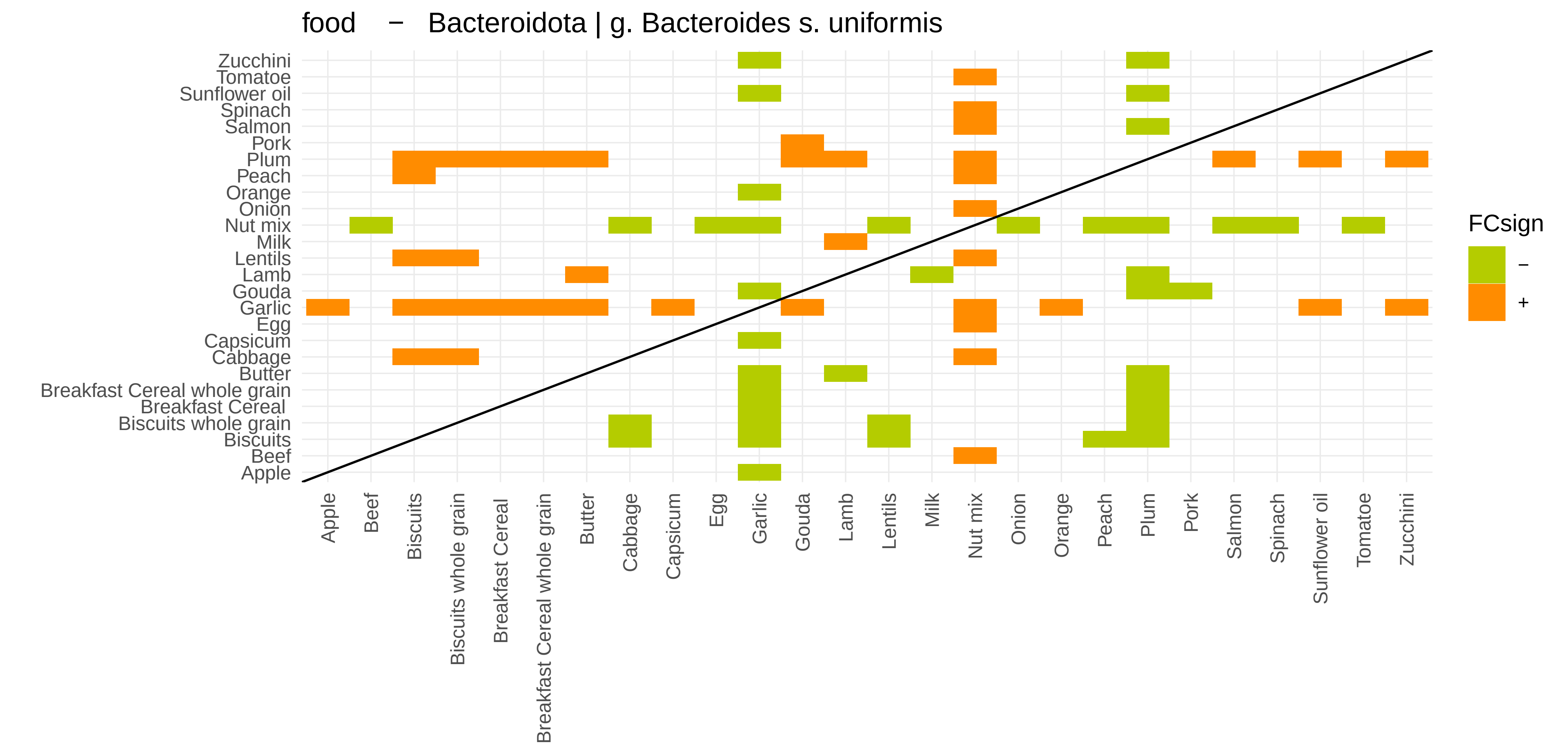

food − Bacteroidota | g. Bacteroides s. uniformis
Zucchini
Tomatoe
Sunflower oil
Spinach
Salmon
Pork
Plum
Peach
Orange
Onion
Nut mix
Milk
Lentils
Lamb
Gouda
Garlic
Egg
Capsicum
Cabbage
Butter
Breakfast Cereal whole grain
Breakfast Cereal
Biscuits whole grain
Biscuits
Beef
Apple
FCsign
−
+

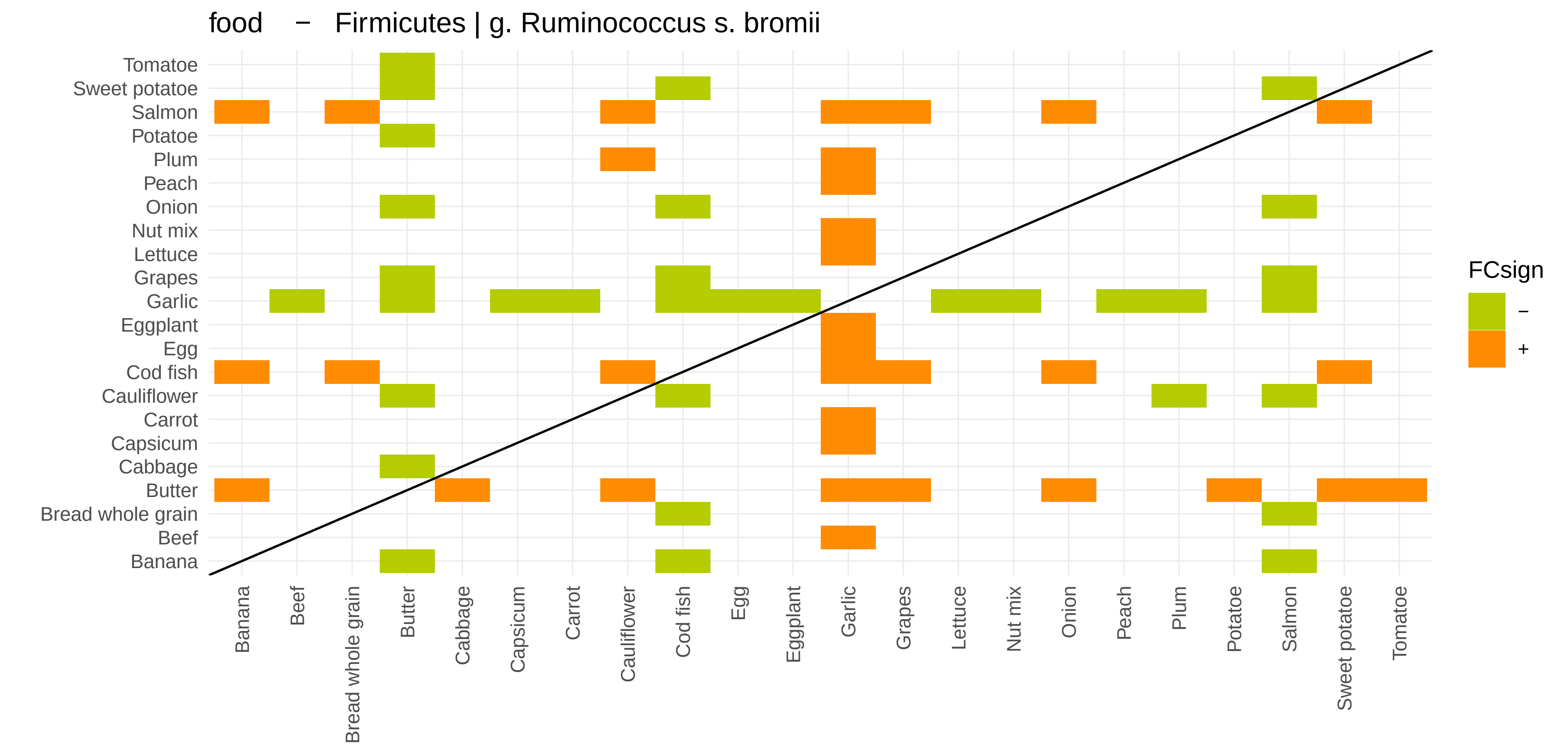
food − Firmicutes | g. Ruminococcus s. bromii
Tomatoe
Sweet potatoe
Salmon
Potatoe
Plum
Peach
Onion
Nut mix
Lettuce
Grapes
Garlic
Eggplant
Egg
Cod fish
Cauliflower
Carrot
Capsicum
Cabbage
Butter
Bread whole grain
Beef
Banana
FCsign
−
+

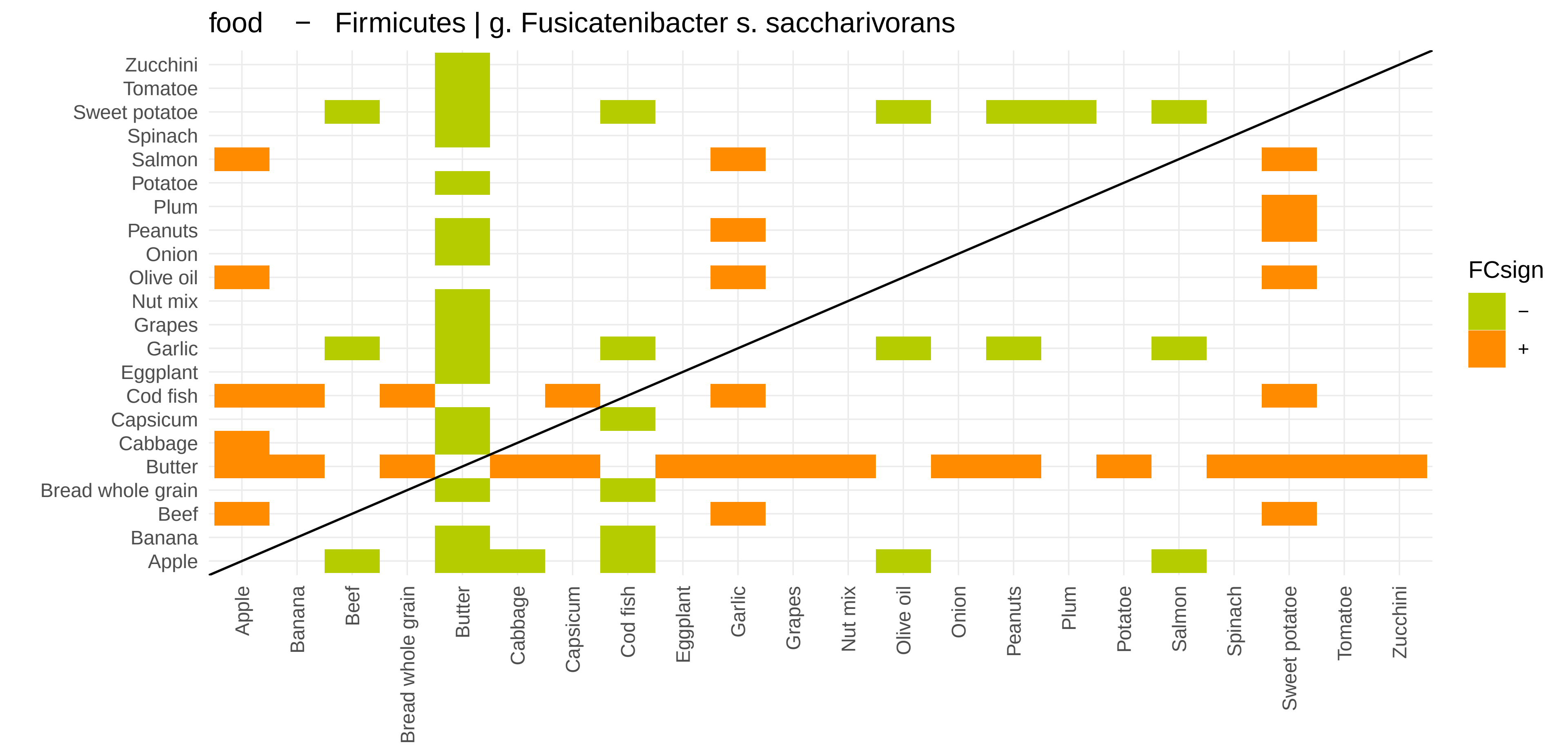

food − Firmicutes | g. Fusicatenibacter s. saccharivorans
Zucchini
Tomatoe
Sweet potatoe
Spinach
Salmon
Potatoe
Plum
Peanuts
Onion
Olive oil
Nut mix
Grapes
Garlic
Eggplant
Cod fish
Capsicum
Cabbage
Butter
Bread whole grain
Beef
Banana
Apple
FCsign
−
+

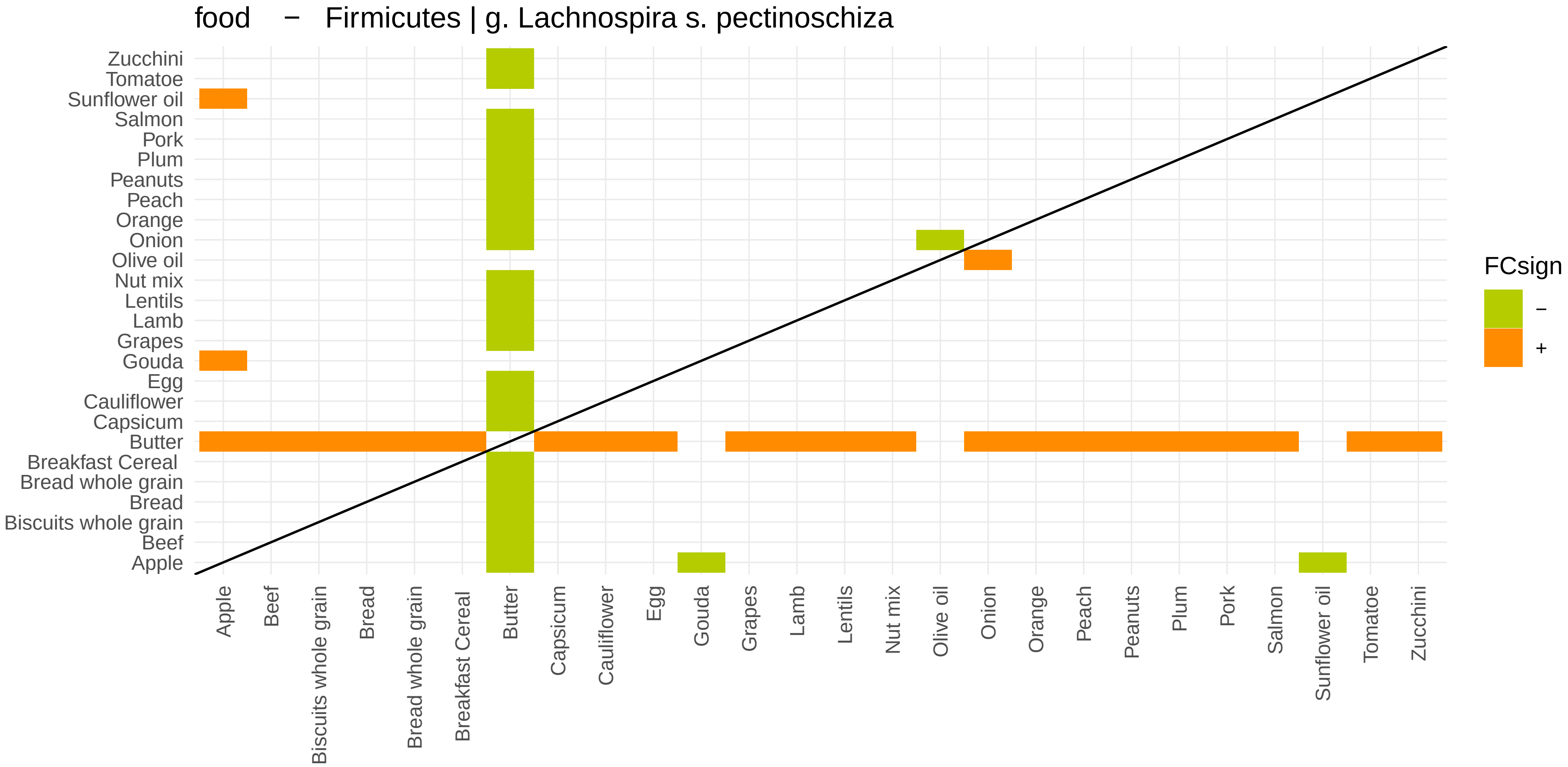
food − Firmicutes | g. Lachnospira s. pectinoschiza
Zucchini
Tomatoe
Sunflower oil
Salmon
Pork
Plum
Peanuts
Peach
Orange
Onion
Olive oil
Nut mix
Lentils
Lamb
Grapes
Gouda
Egg
Cauliflower
Capsicum
Butter
Breakfast Cereal
Bread whole grain
Bread
Biscuits whole grain
Beef
Apple
FCsign
−
+

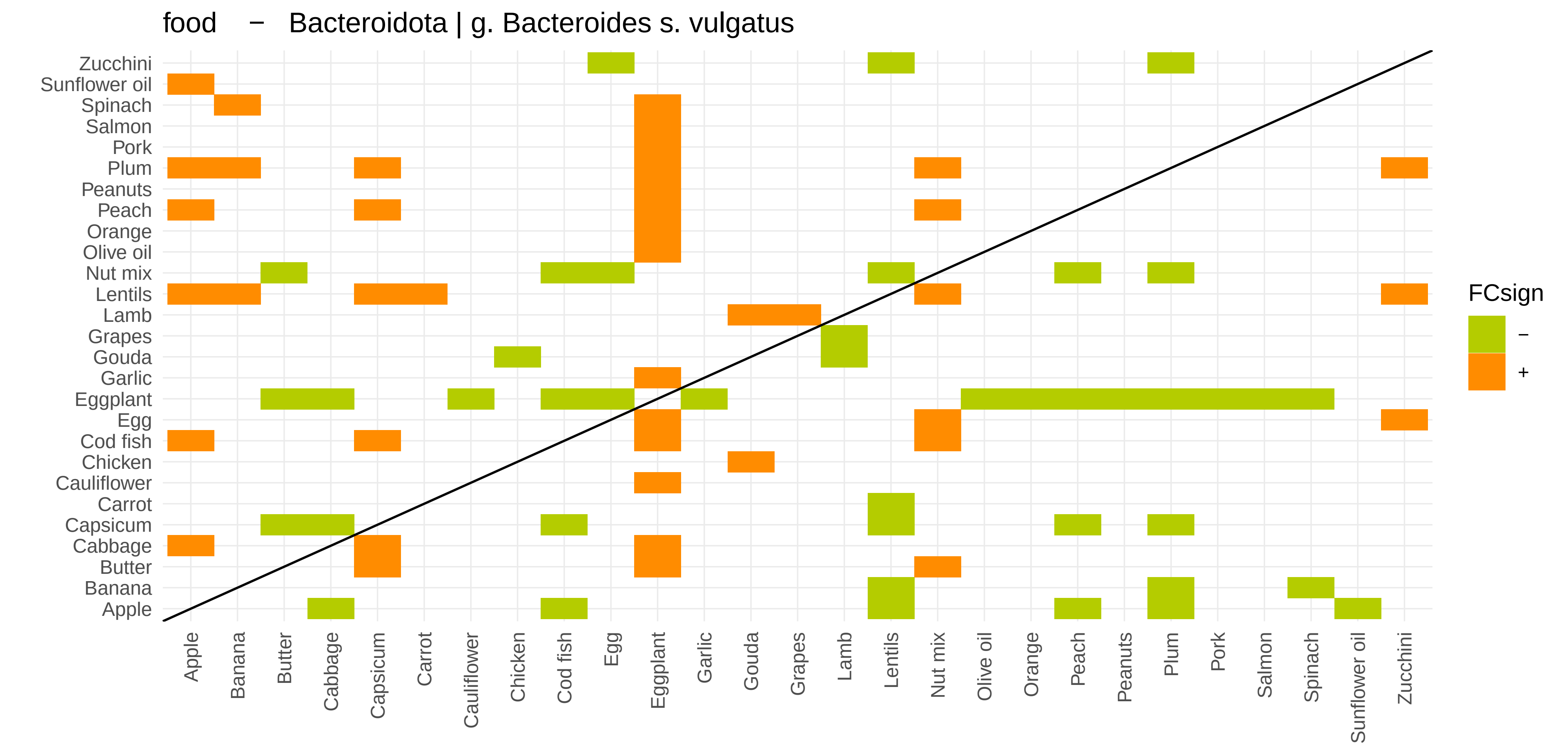
food − Bacteroidota | g. Bacteroides s. vulgatus
Zucchini
Sunflower oil
Spinach
Salmon
Pork
Plum
Peanuts
Peach
Orange
Olive oil
Nut mix
Lentils
Lamb
Grapes
Gouda
Garlic
Eggplant
Egg
Cod fish
Chicken
Cauliflower
Carrot
Capsicum
Cabbage
Butter
Banana
Apple
Apple
Banana
Butter
Cabbage
Capsicum
Carrot
Cauliflower
Chicken
Cod fish
Egg
Eggplant
Garlic
Gouda
Grapes
Lamb
Lentils
Nut mix
Olive oil
Orange
Peach
Peanuts
Plum
Pork
Salmon
Spinach
Sunflower oil
Zucchini
FCsign
−
+

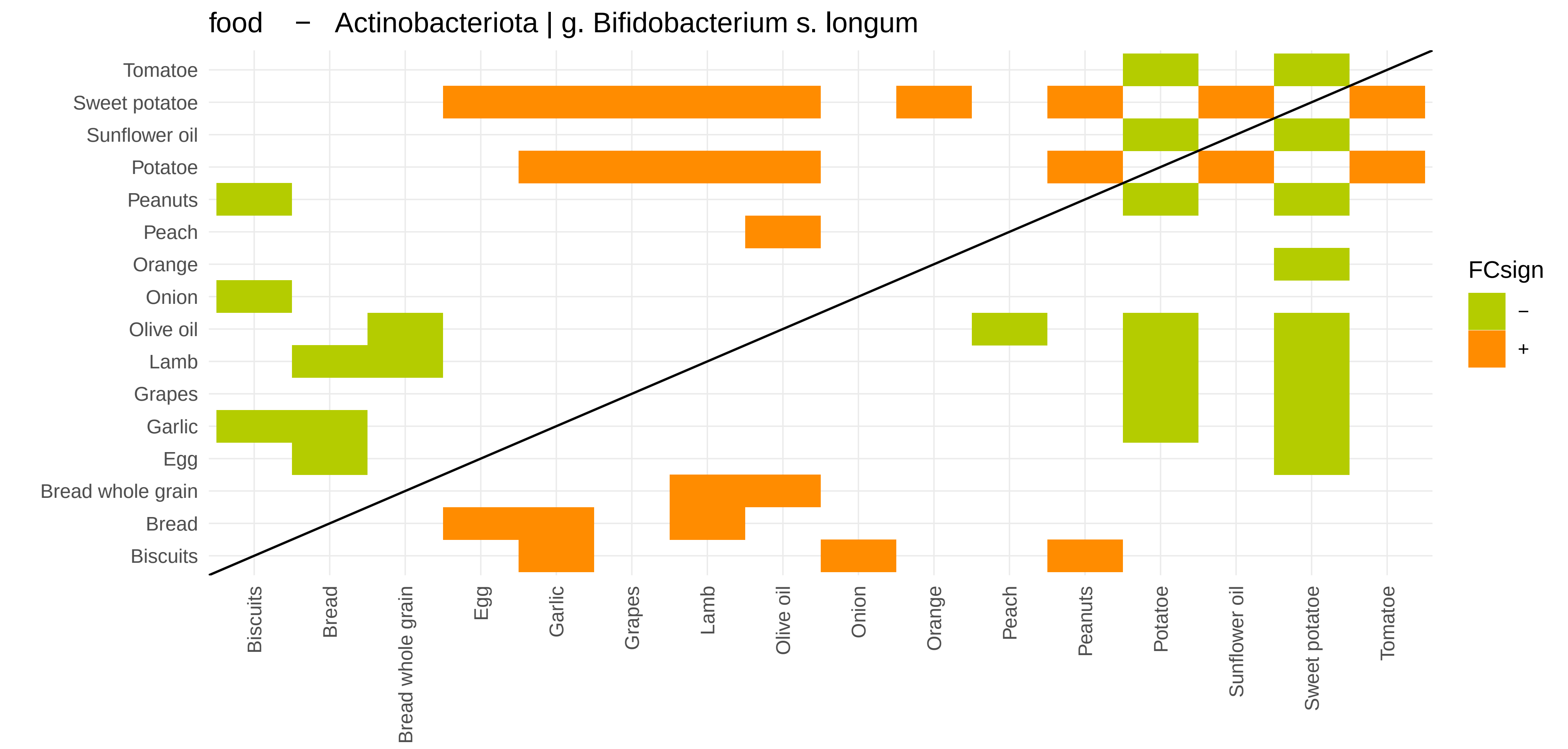
food − Actinobacteriota | g. Bifidobacterium s. longum
Tomatoe
Sweet potatoe
Sunflower oil
Potatoe
Peanuts
Peach
Orange
Onion
Olive oil
Lamb
Grapes
Garlic
Egg
Bread whole grain
Bread
Biscuits
Biscuits
Bread
Bread whole grain
Egg
Garlic
Grapes
Lamb
Olive oil
Onion
Orange
Peach
Peanuts
Potatoe
Sunflower oil
Sweet potatoe
Tomatoe
FCsign
−
+

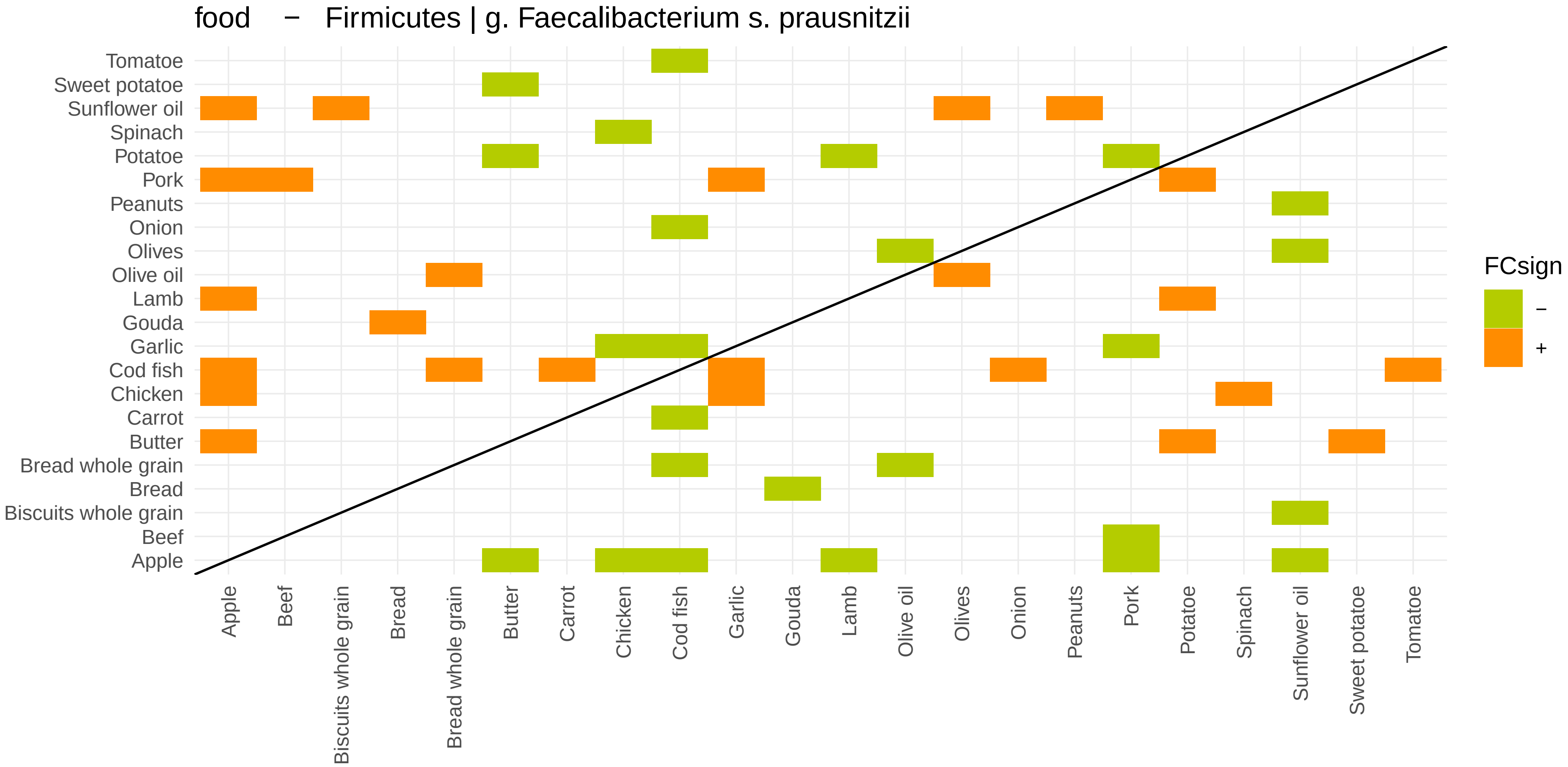

food – Firmicutes | g. Faecalibacterium s. prausnitzii
Tomatoe
Sweet potatoe
Sunflower oil
Spinach
Potatoe
Pork
Peanuts
Onion
Olives
Olive oil
Lamb
Gouda
Garlic
Cod fish
Chicken
Carrot
Butter
Bread whole grain
Bread
Biscuits whole grain
Beef
Apple
FCsign
−
+

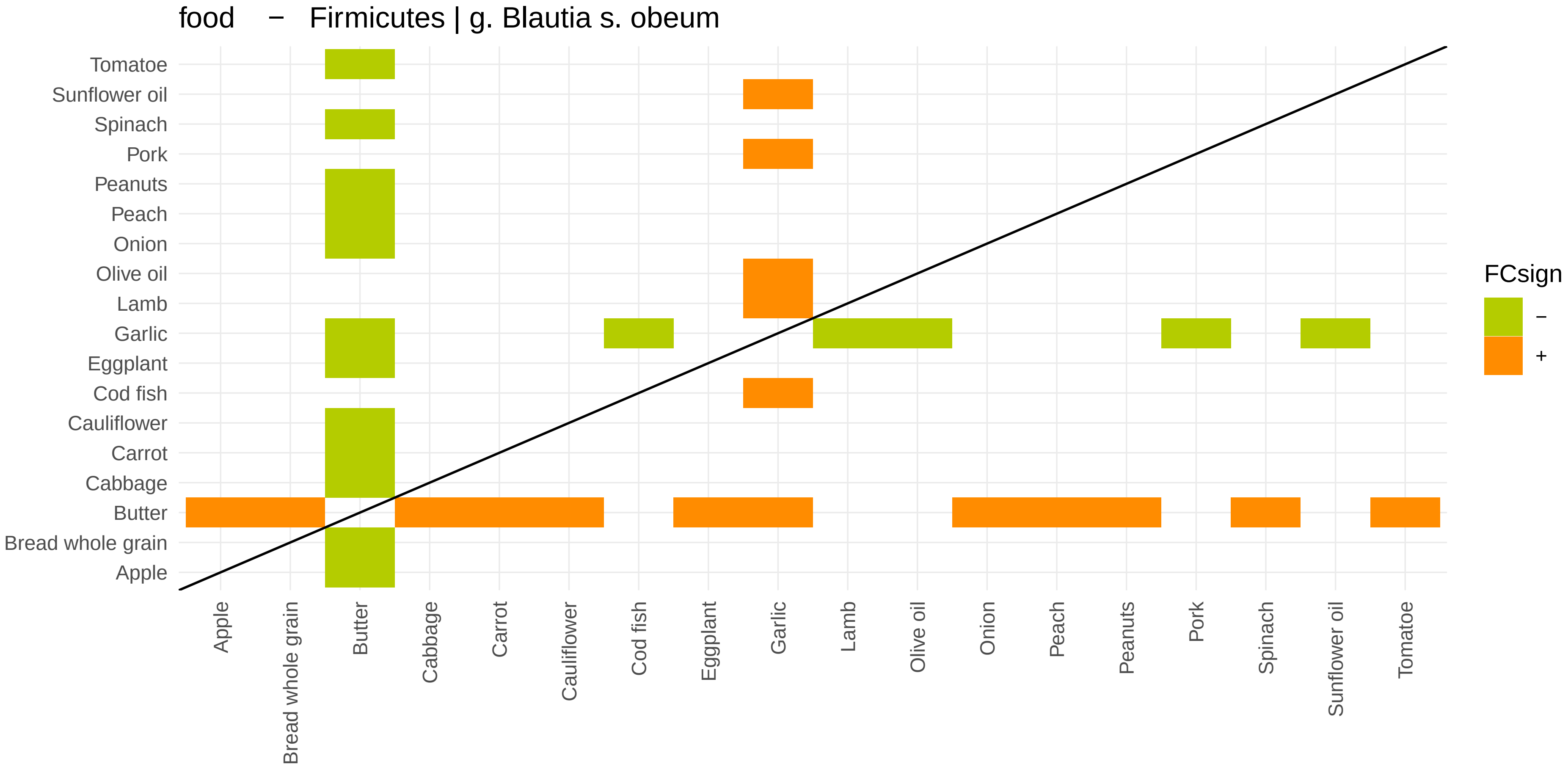
food − Firmicutes | g. Blautia s. obeum
Tomatoe
Sunflower oil
Spinach
Pork
Peanuts
Peach
Onion
Olive oil
Lamb
Garlic
Eggplant
Cod fish
Cauliflower
Carrot
Cabbage
Butter
Bread whole grain
Apple
FCsign
−
+

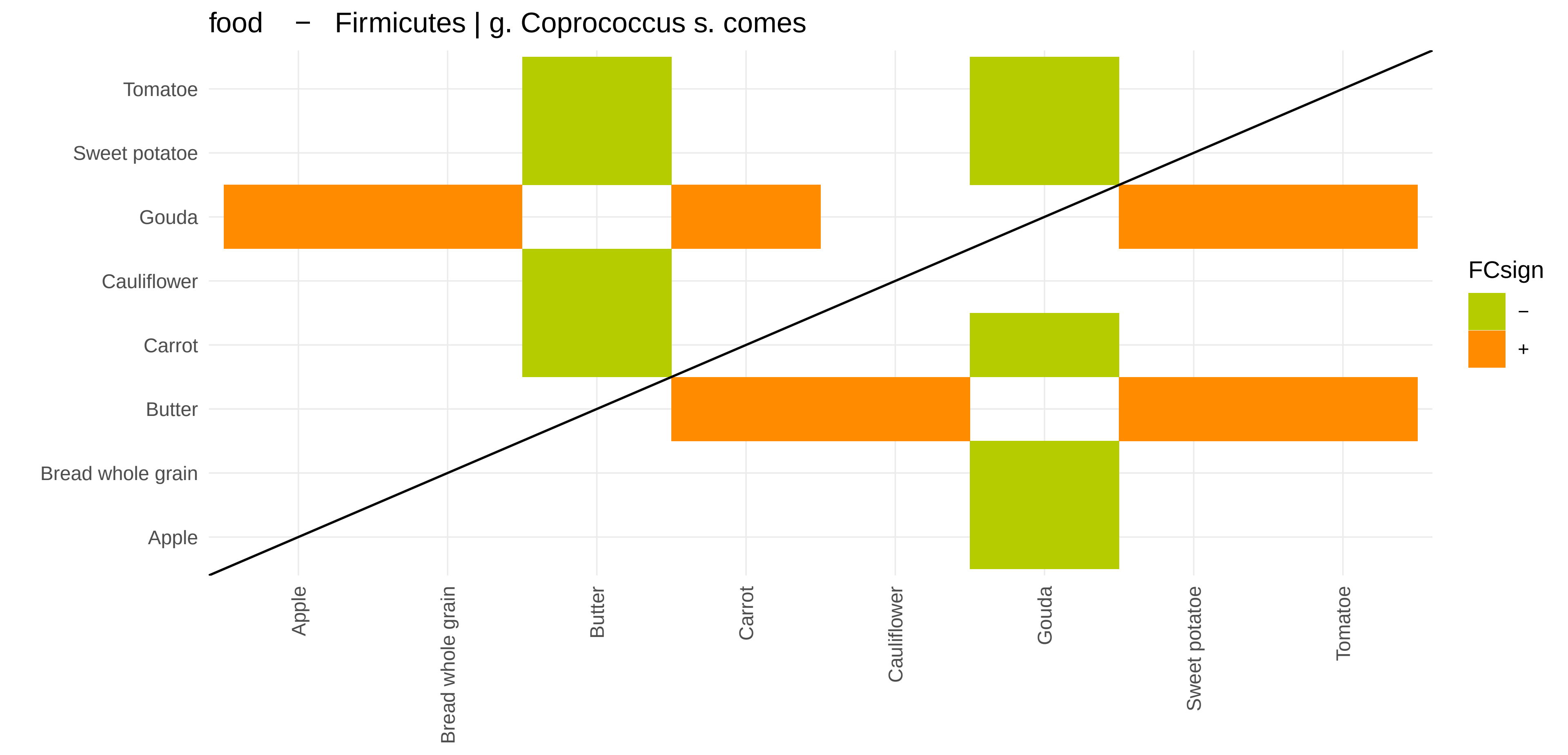

food − Firmicutes | g. Coprococcus s. comes
Tomatoe
Sweet potatoe
Gouda
Cauliflower
Carrot
Butter
Bread whole grain
Apple
FCsign
−
+

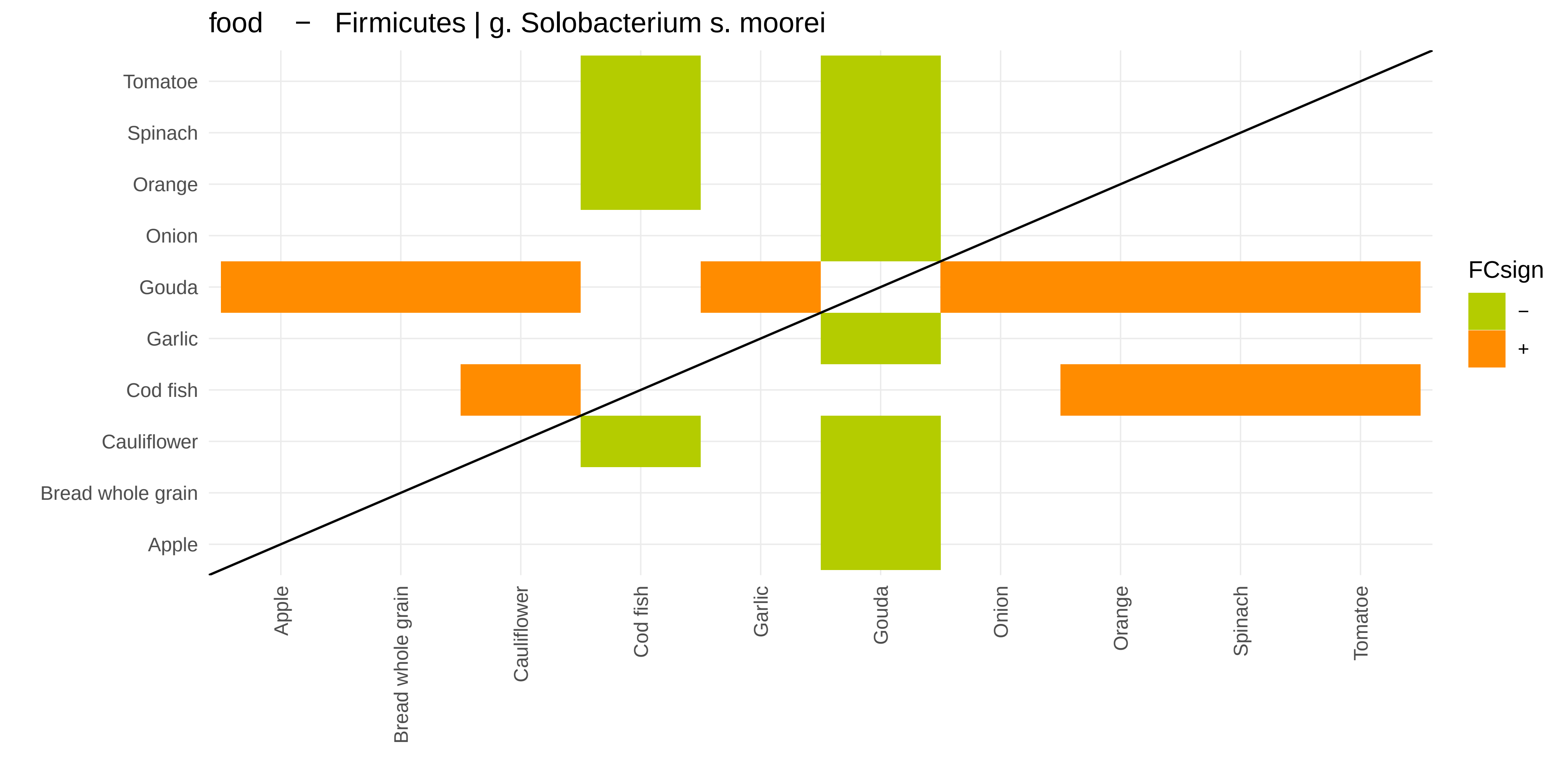
food − Firmicutes | g. Solobacterium s. moorei
Tomatoe
Spinach
Orange
Onion
Gouda
Garlic
Cod fish
Cauliflower
Bread whole grain
Apple
Apple
Bread whole grain
Cauliflower
Cod fish
Garlic
Gouda
Onion
Orange
Spinach
Tomatoe
FCsign
−
+

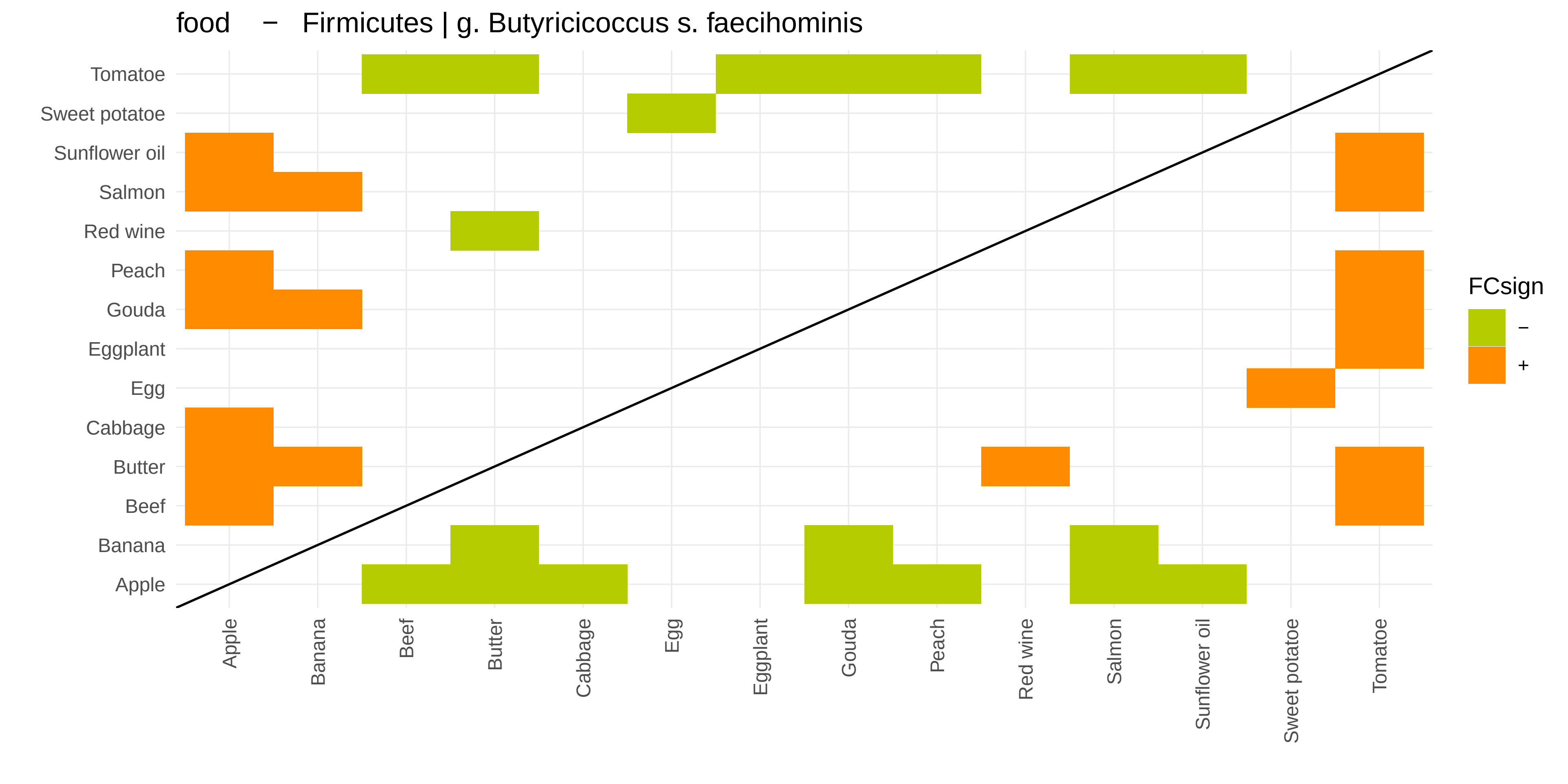

food − Firmicutes | g. Butyricicoccus s. faecihominis
Tomatoe
Sweet potatoe
Sunflower oil
Salmon
Red wine
Peach
Gouda
Eggplant
Egg
Cabbage
Butter
Beef
Banana
Apple
FCsign
−
+

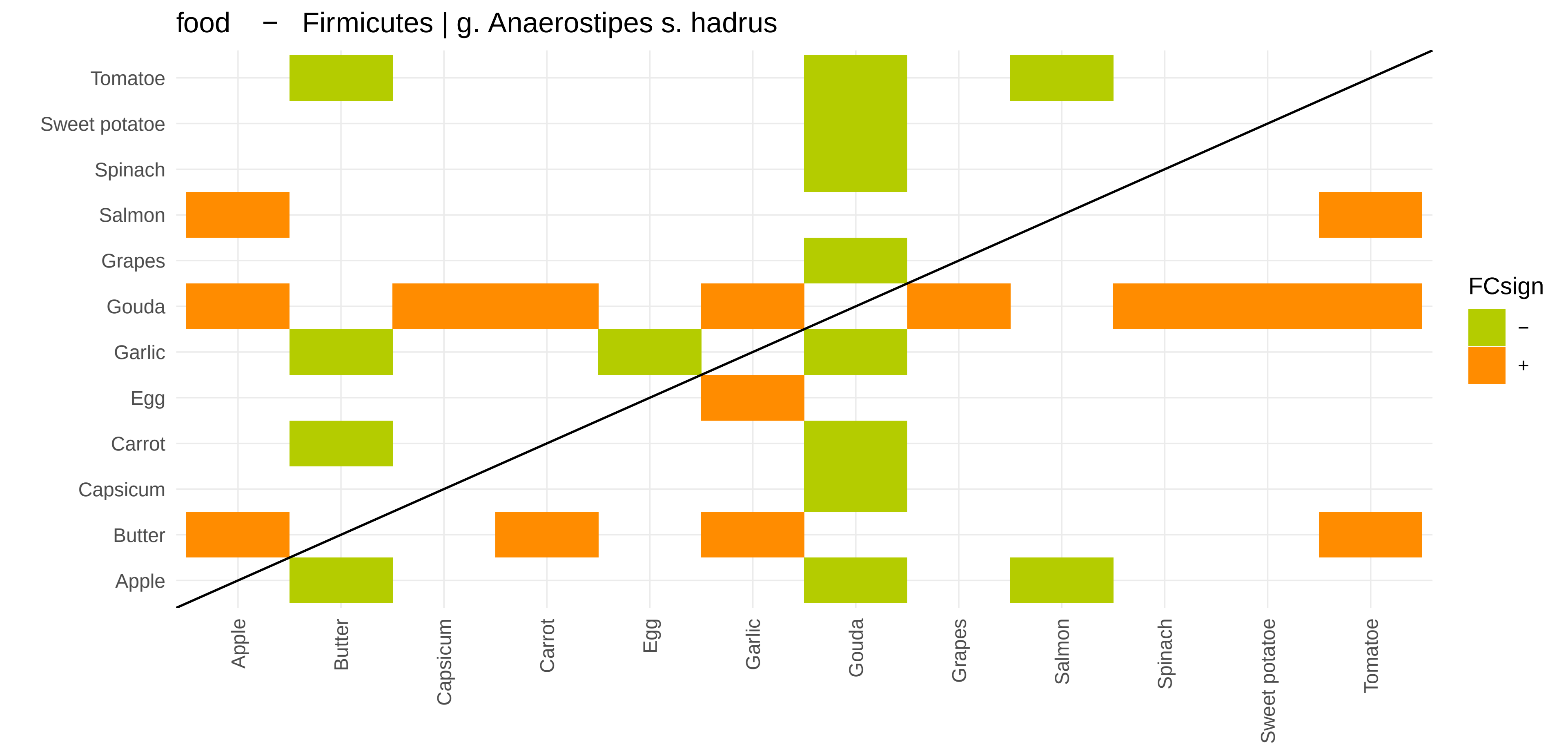

food − Firmicutes | g. Anaerostipes s. hadrus
Tomatoe
Sweet potatoe
Spinach
Salmon
Grapes
Gouda
Garlic
Egg
Carrot
Capsicum
Butter
Apple
Apple
Butter
Capsicum
Carrot
Egg
Garlic
Gouda
Grapes
Salmon
Spinach
Sweet potatoe
Tomatoe
FCsign
−
+

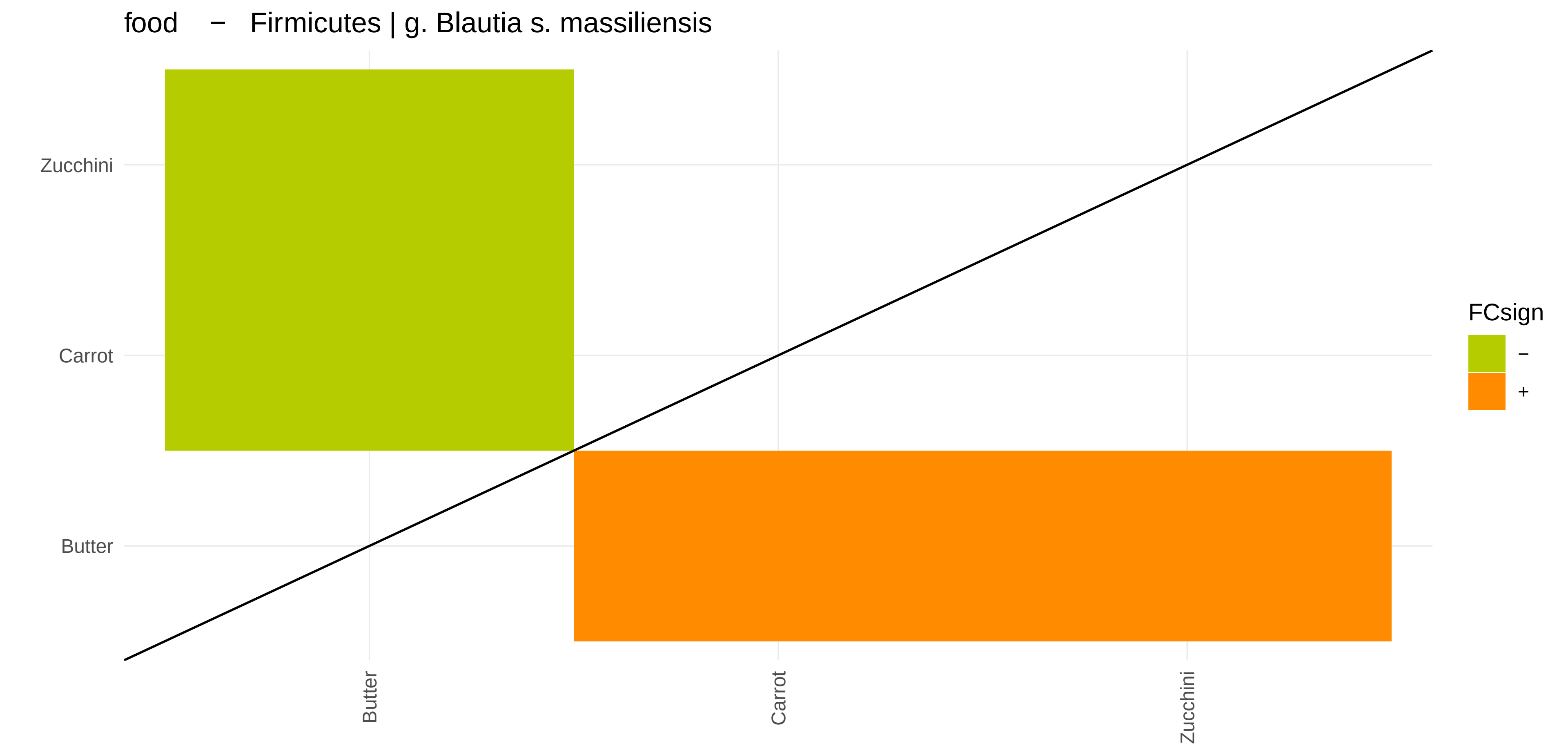

food − Firmicutes | g. Blautia s. massiliensis
Zucchini
Carrot
Butter
Butter
Carrot
Zucchini
FCsign
−
+

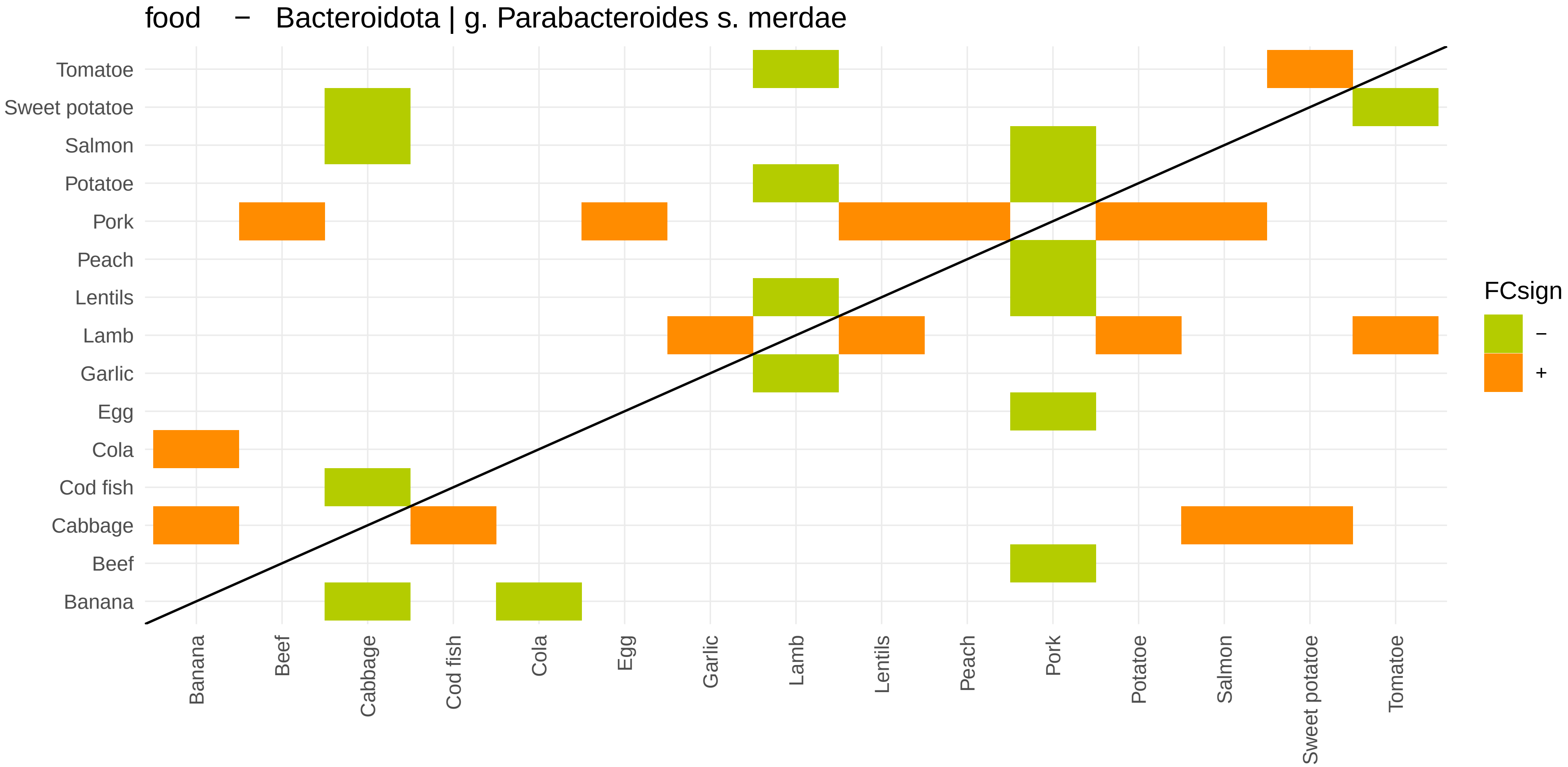
food − Bacteroidota | g. Parabacteroides s. merdae
Tomatoe
Sweet potatoe
Salmon
Potatoe
Pork
Peach
Lentils
Lamb
Garlic
Egg
Cola
Cod fish
Cabbage
Beef
Banana
FCsign
−
+

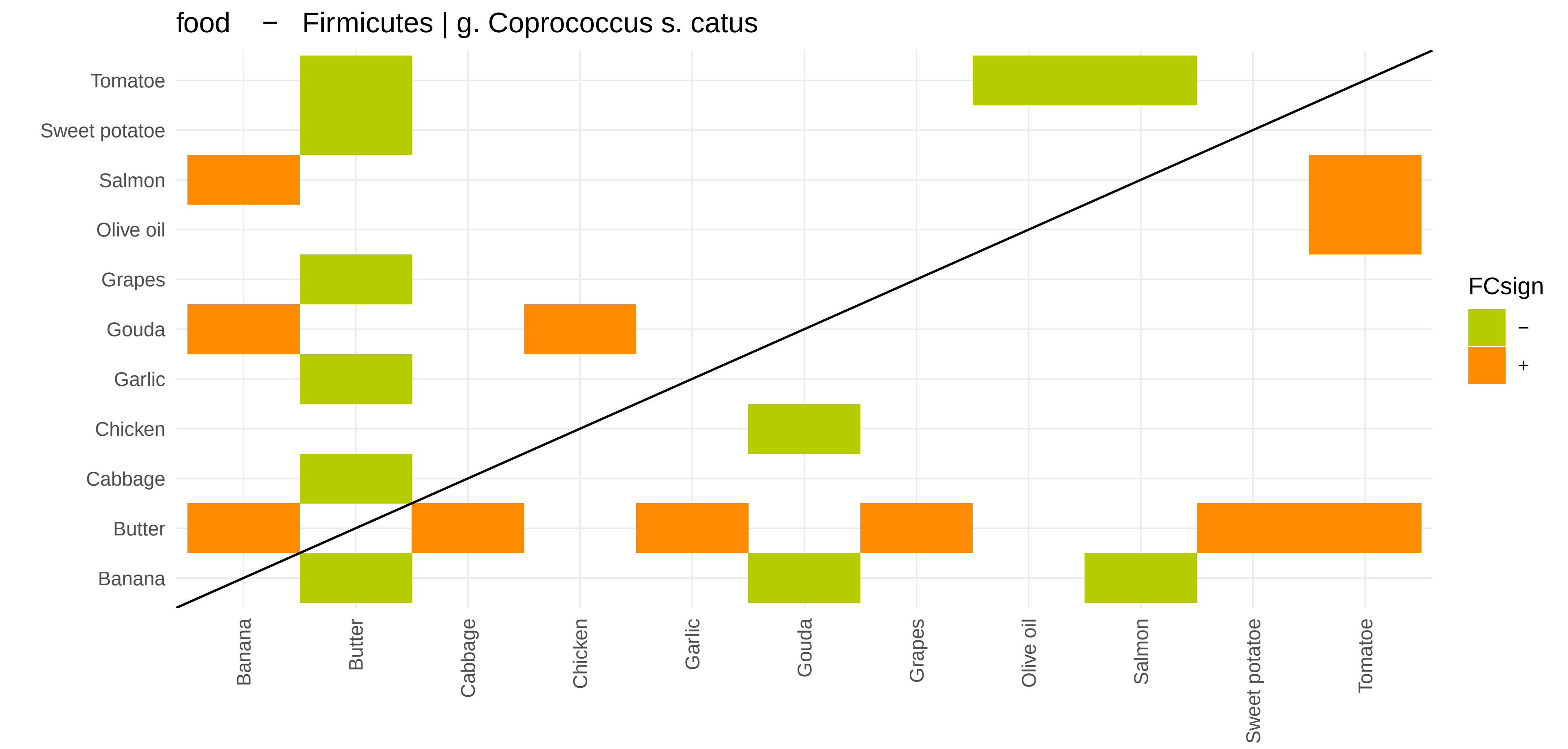

food − Firmicutes | g. Coprococcus s. catus
Tomatoe
Sweet potatoe
Salmon
Olive oil
Grapes
Gouda
Garlic
Chicken
Cabbage
Butter
Banana
Banana
Butter
Cabbage
Chicken
Garlic
Gouda
Grapes
Olive oil
Salmon
Sweet potatoe
Tomatoe
FCsign
−
+

food − Actinobacteriota | g. Adlercreutzia s. equolifaciens

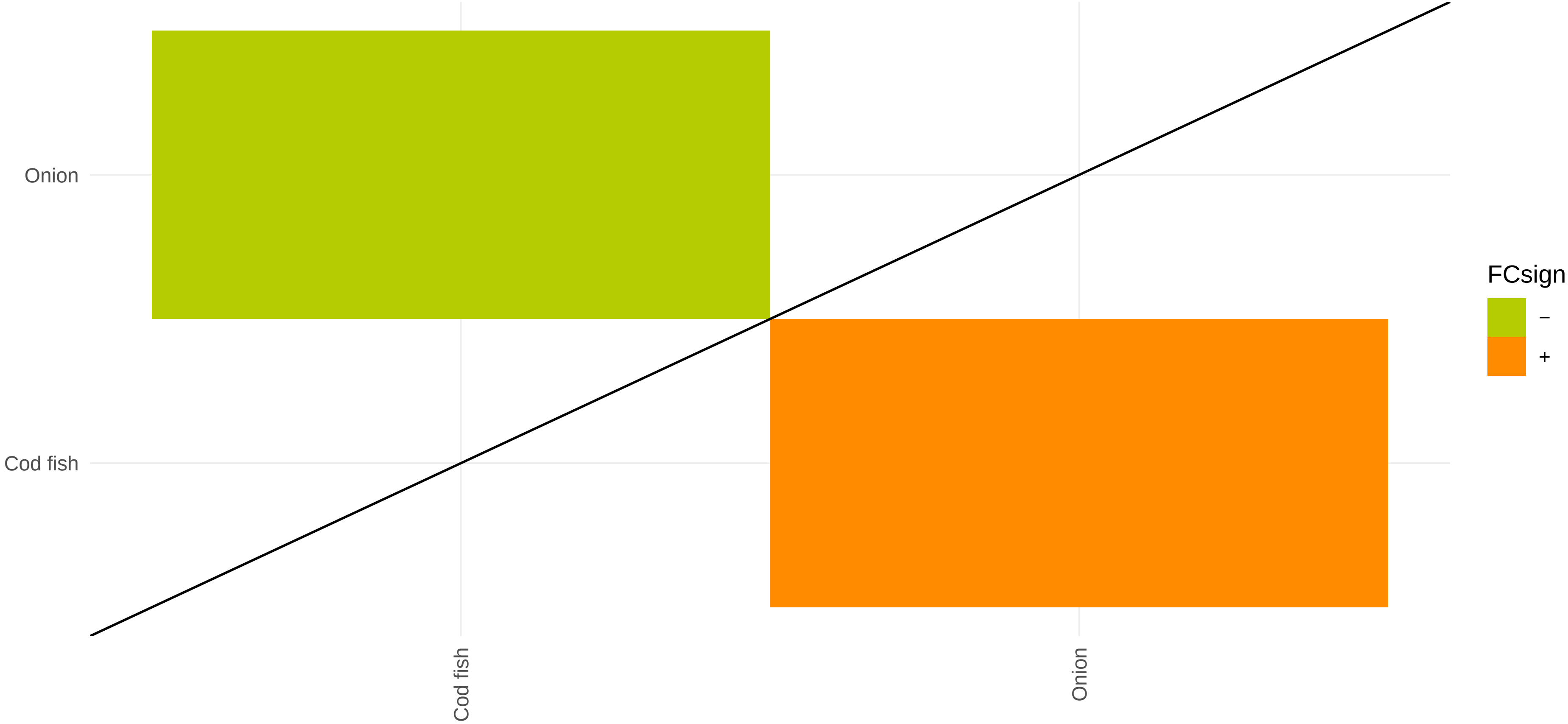

Supplement: Supplementary Figure 4 — Heatmap of relative abundance differences at species level between foods. Y-axis foods have higher (orange) or lower (green) abundance of a given species against X-axis foods. The ANCOM method was used for comparisons with the Benjamini–Hochberg procedure for false discovery rate control. Significant comparisons (q < 0.05) in all the individuals are represented (PDF). [file Data_Sheet_4.PDF]
