# Supplementary material for: Effects of different foods and cooking methods on the gut microbiota: an in vitro approach
Source: Front Microbiol. 2024 Jan 8;14:1334623. doi: 10.3389/fmicb.2023.1334623 (PMC10800916; doi:10.3389/fmicb.2023.1334623)
Supplement: Supplementary Figure 5 — Fold-changes in relative abundance of bacterial genera between cooking methods per food category. Fold-changes are represented as log2(FC). Significant comparisons (q < 0.05) according to ANCOM for each individual are represented. Cooking methods are represented in different colors and the individuals in which a given comparison is significant are indicated on the bars (PDF). [file Data_Sheet_5.PDF]

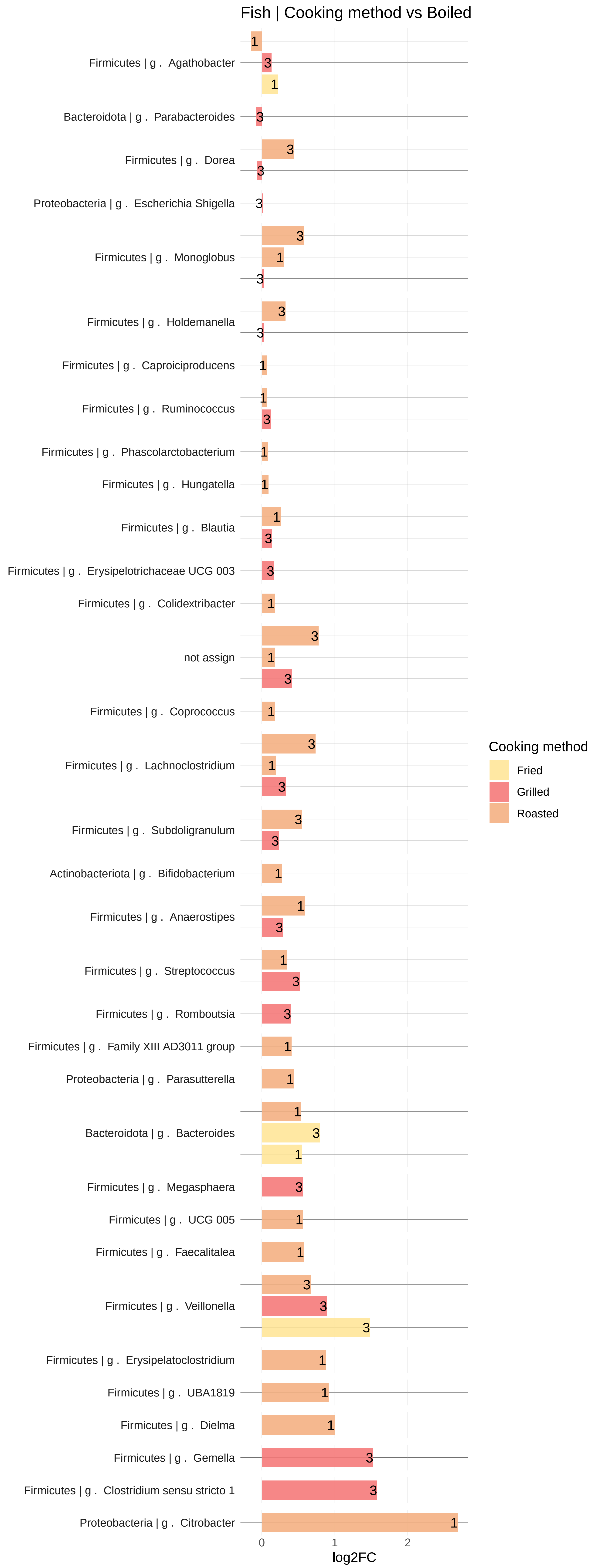

Fish | Cooking method vs Boiled
Firmicutes | g . Agathobacter
Bacteroidota | g . Parabacteroides
Firmicutes | g . Dorea
Proteobacteria | g . Escherichia Shigella
Firmicutes | g . Monoglobus
Firmicutes | g . Holdemanella
Firmicutes | g . Caproiciproducens
Firmicutes | g . Ruminococcus
Firmicutes | g . Phascolarctobacterium
Firmicutes | g . Hungatella
Firmicutes | g . Blautia
Firmicutes | g . Erysipelotrichaceae UCG 003
Firmicutes | g . Colidextribacter
not assign
Firmicutes | g . Coprococcus
Firmicutes | g . Lachnoclostridium
Firmicutes | g . Subdoligranulum
Actinobacteriota | g . Bifidobacterium
Firmicutes | g . Anaerostipes
Firmicutes | g . Streptococcus
Firmicutes | g . Romboutsia
Firmicutes | g . Family XIII AD3011 group
Proteobacteria | g . Parasutterella
Bacteroidota | g . Bacteroides
Firmicutes | g . Megasphaera
Firmicutes | g . UCG 005
Firmicutes | g . Faecalitalea
Firmicutes | g . Veillonella
Firmicutes | g . Erysipelatoclostridium
Firmicutes | g . UBA1819
Firmicutes | g . Dielma
Firmicutes | g . Gemella
Firmicutes | g . Clostridium sensu stricto 1
Proteobacteria | g . Citrobacter
0
1
2
log2FC
Cooking method
Fried
Grilled
Roasted

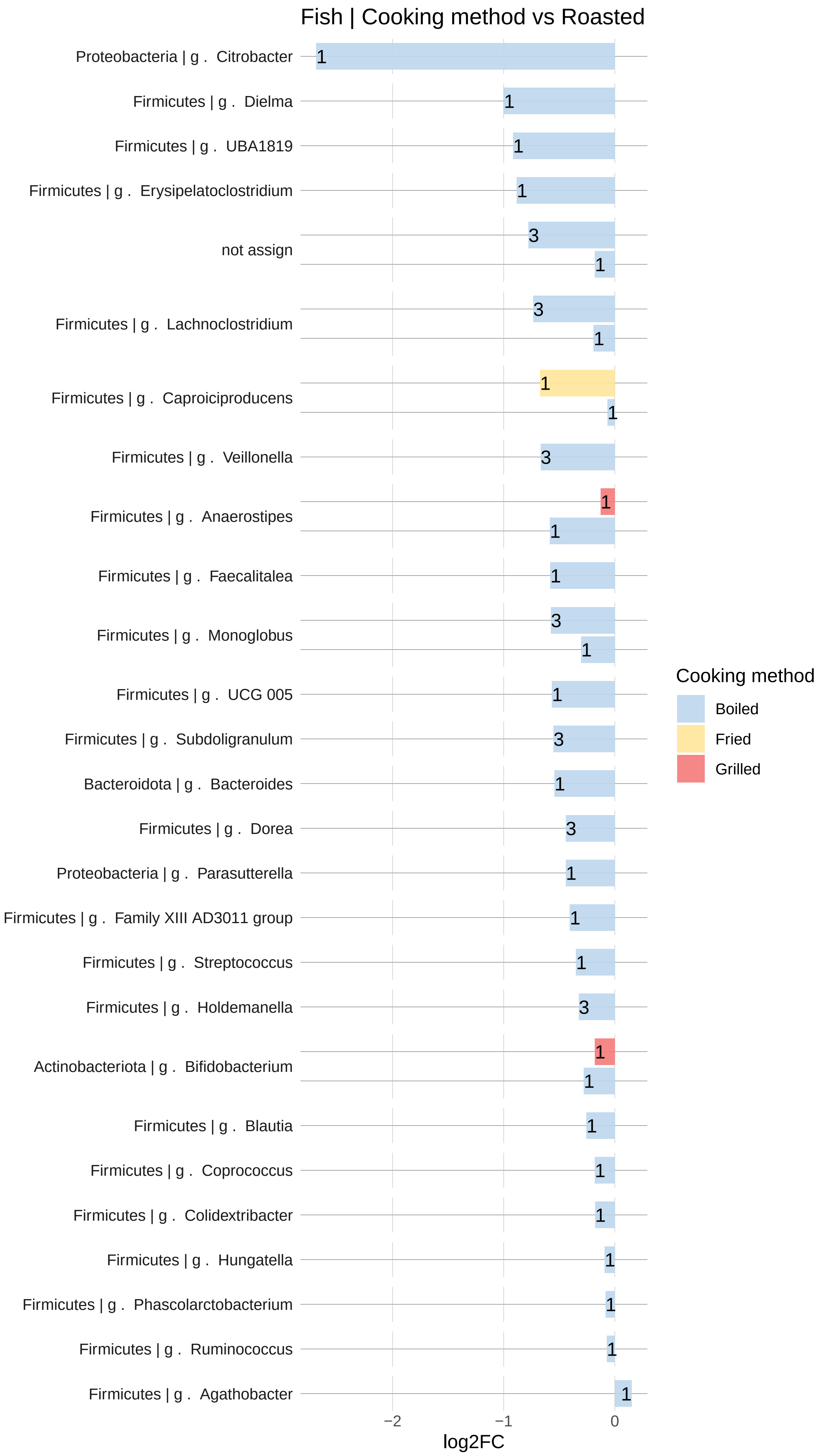

Fish | Cooking method vs Roasted
Proteobacteria | g . Citrobacter
Firmicutes | g . Dielma
Firmicutes | g . UBA1819
Firmicutes | g . Erysipelatoclostridium
not assign
Firmicutes | g . Lachnoclostridium
Firmicutes | g . Caproiciproducens
Firmicutes | g . Veillonella
Firmicutes | g . Anaerostipes
Firmicutes | g . Faecalitalea
Firmicutes | g . Monoglobus
Firmicutes | g . UCG 005
Firmicutes | g . Subdoligranulum
Bacteroidota | g . Bacteroides
Firmicutes | g . Dorea
Proteobacteria | g . Parasutterella
Firmicutes | g . Family XIII AD3011 group
Firmicutes | g . Streptococcus
Firmicutes | g . Holdemanella
Actinobacteriota | g . Bifidobacterium
Firmicutes | g . Blautia
Firmicutes | g . Coprococcus
Firmicutes | g . Colidextribacter
Firmicutes | g . Hungatella
Firmicutes | g . Phascolarctobacterium
Firmicutes | g . Ruminococcus
Firmicutes | g . Agathobacter
−2
−1
0
log2FC
Cooking method
Boiled
Fried
Grilled

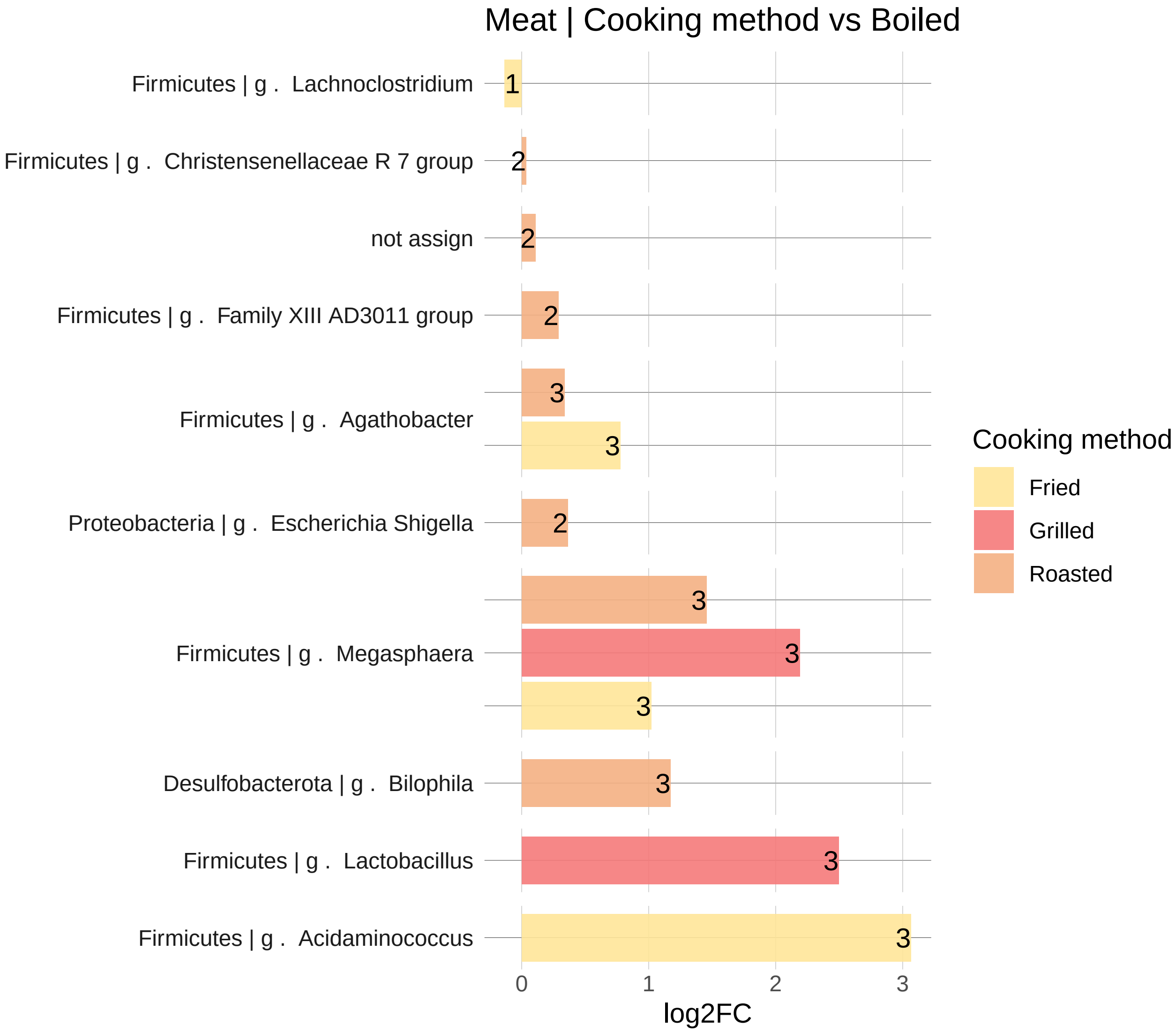

Meat | Cooking method vs Boiled
Firmicutes | g . Lachnoclostridium
Firmicutes | g . Christensenellaceae R 7 group
not assign
Firmicutes | g . Family XIII AD3011 group
Firmicutes | g . Agathobacter
Proteobacteria | g . Escherichia Shigella
Firmicutes | g . Megasphaera
Desulfobacterota | g . Bilophila
Firmicutes | g . Lactobacillus
Firmicutes | g . Acidaminococcus
1
2
2
2
3
3
2
3
3
3
3
3
3
0
1
2
3
log2FC
Cooking method
Fried
Grilled
Roasted

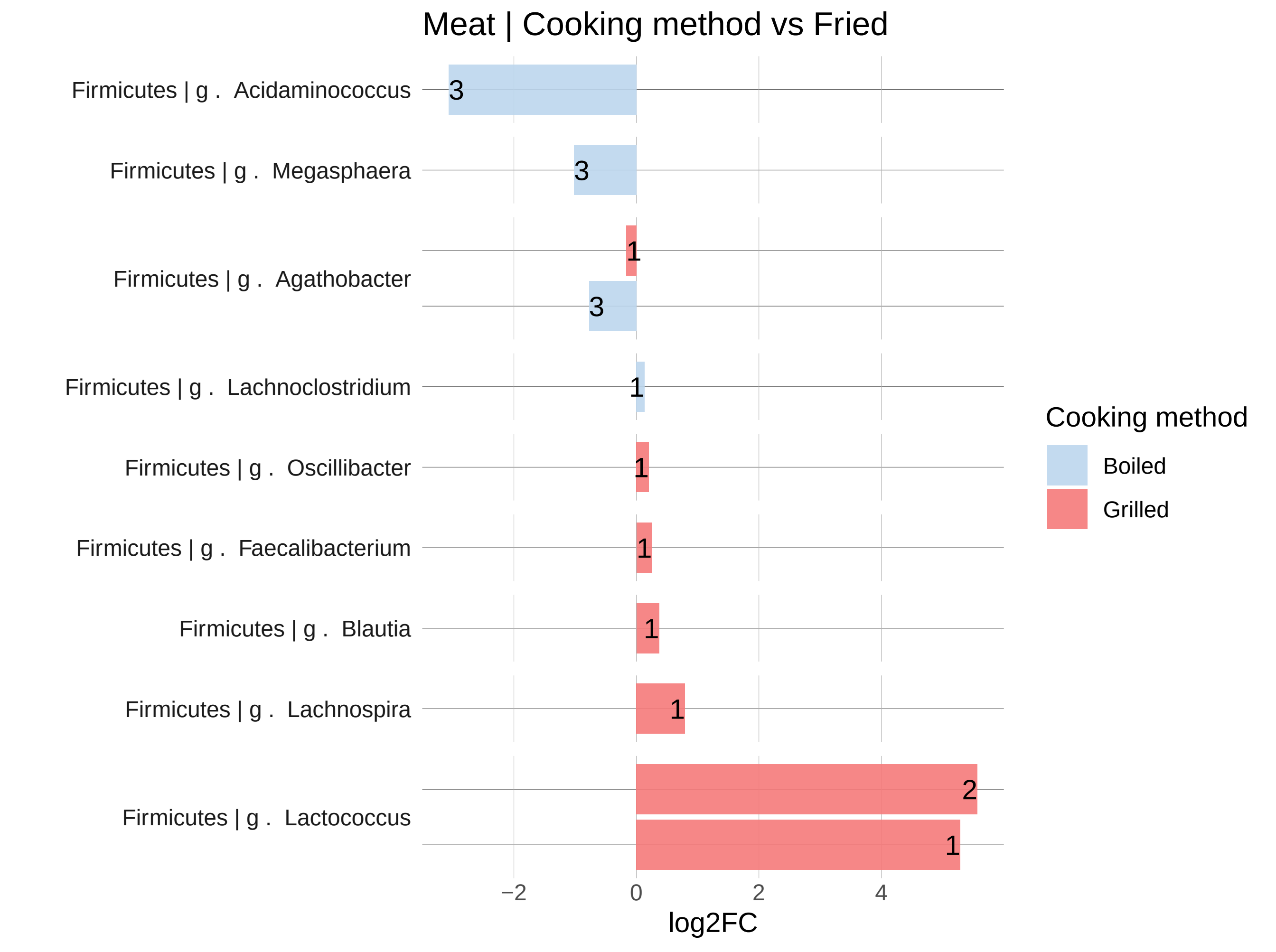
Meat | Cooking method vs Fried
Firmicutes | g . Acidaminococcus
Firmicutes | g . Megasphaera
Firmicutes | g . Agathobacter
Firmicutes | g . Lachnoclostridium
Firmicutes | g . Oscillibacter
Firmicutes | g . Faecalibacterium
Firmicutes | g . Blautia
Firmicutes | g . Lachnospira
Firmicutes | g . Lactococcus
3
3
1
3
1
1
1
1
1
2
1
−2
0
2
4
log2FC
Cooking method
Boiled
Grilled

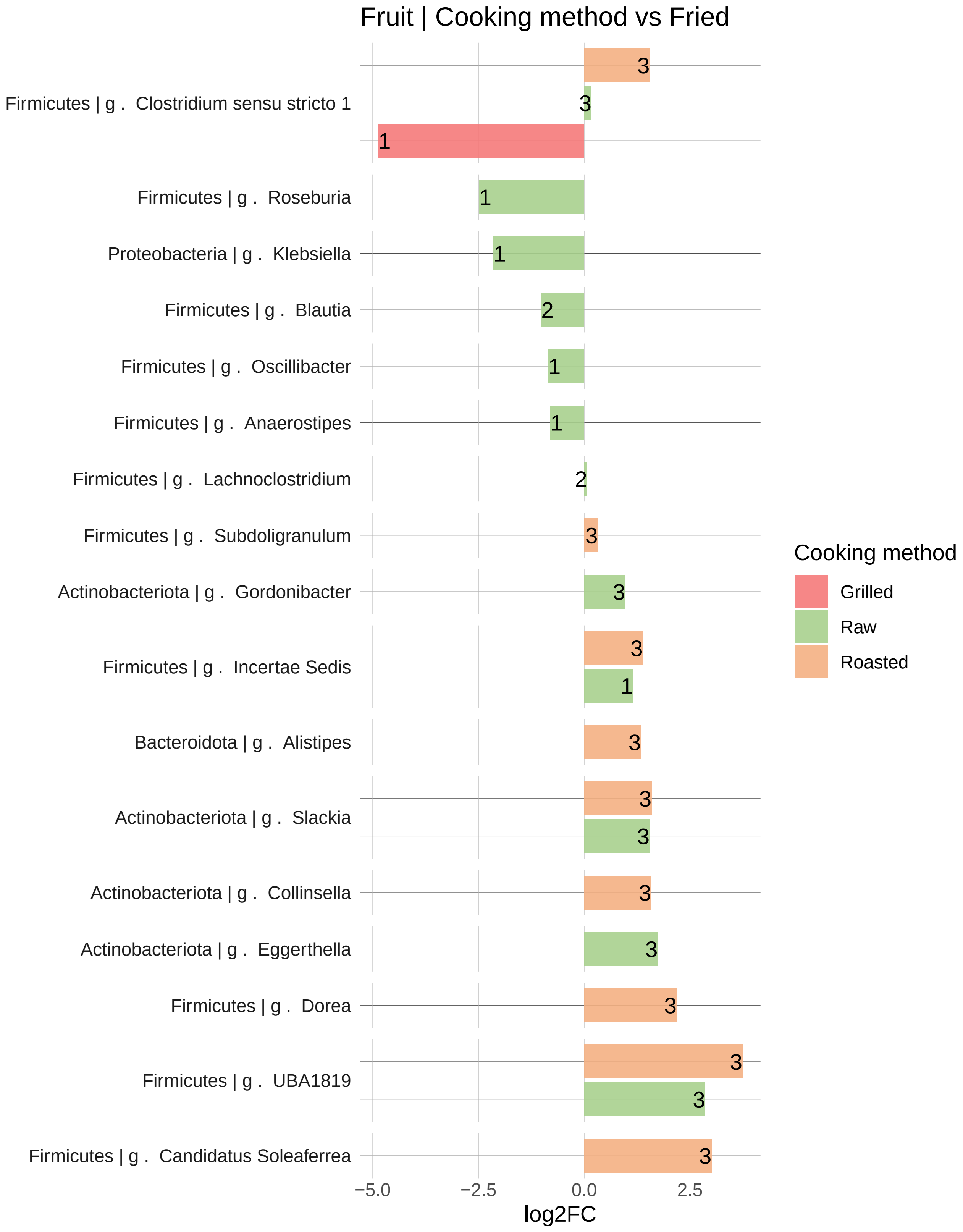
Fruit | Cooking method vs Fried
Firmicutes | g . Clostridium sensu stricto 1
Firmicutes | g . Roseburia
Proteobacteria | g . Klebsiella
Firmicutes | g . Blautia
Firmicutes | g . Oscillibacter
Firmicutes | g . Anaerostipes
Firmicutes | g . Lachnoclostridium
Firmicutes | g . Subdoligranulum
Actinobacteriota | g . Gordonibacter
Firmicutes | g . Incertae Sedis
Bacteroidota | g . Alistipes
Actinobacteriota | g . Slackia
Actinobacteriota | g . Collinsella
Actinobacteriota | g . Eggerthella
Firmicutes | g . Dorea
Firmicutes | g . UBA1819
Firmicutes | g . Candidatus Soleaferrea
−5.0
−2.5
0.0
2.5
log2FC
Cooking method
Grilled
Raw
Roasted

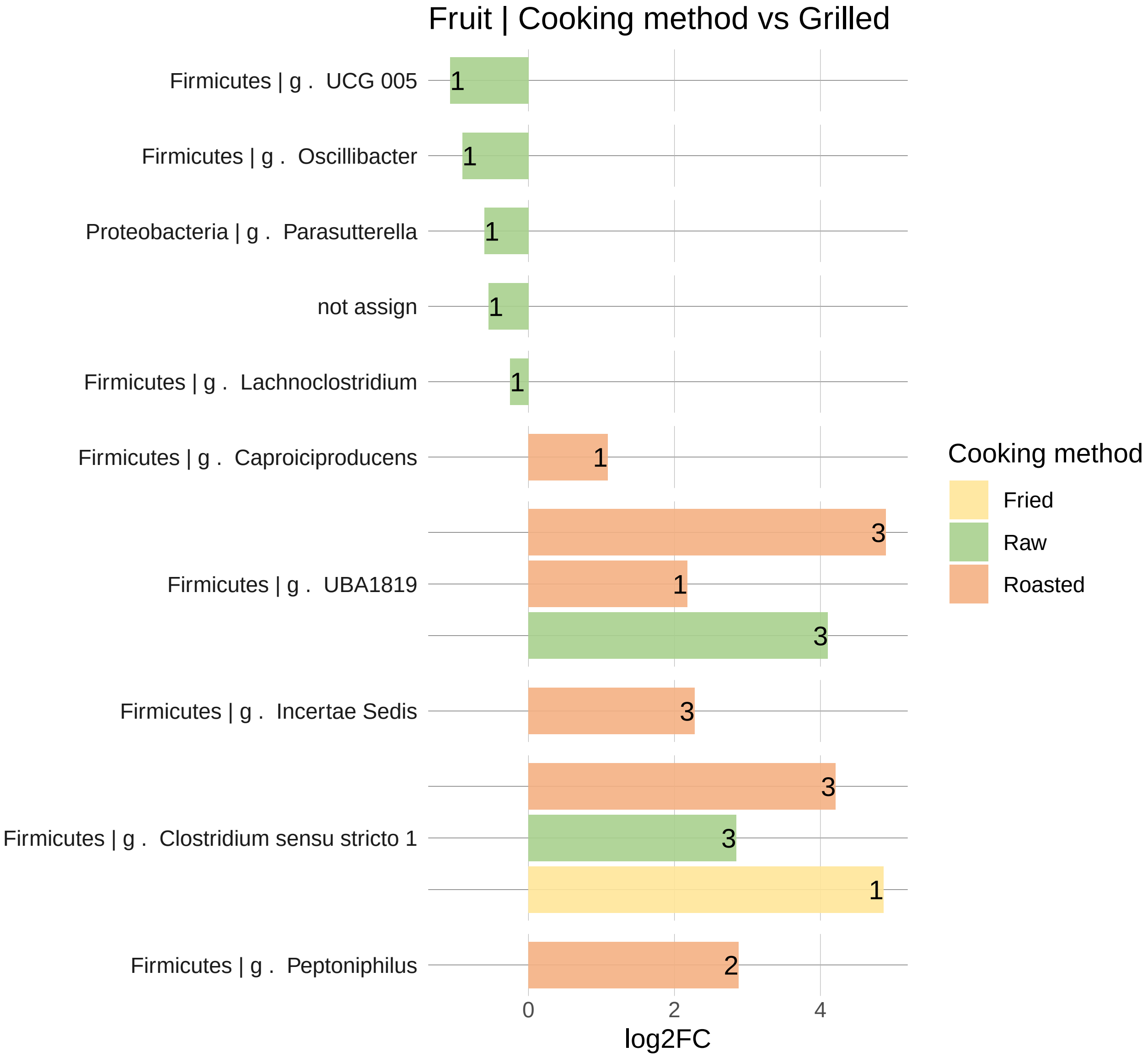
Fruit | Cooking method vs Grilled
Firmicutes | g . UCG 005
Firmicutes | g . Oscillibacter
Proteobacteria | g . Parasutterella
not assign
Firmicutes | g . Lachnoclostridium
Firmicutes | g . Caproiciproducens
Firmicutes | g . UBA1819
Firmicutes | g . Incertae Sedis
Firmicutes | g . Clostridium sensu stricto 1
Firmicutes | g . Peptoniphilus
1
1
1
1
1
1
3
1
3
3
3
3
1
2
0
2
4
log2FC
Cooking method
Fried
Raw
Roasted

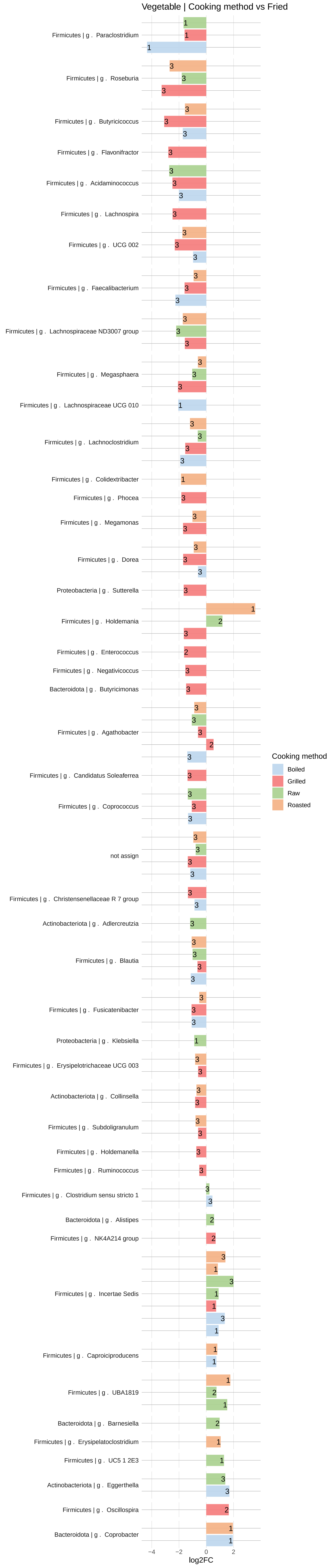
Vegetable | Cooking method vs Fried
Firmicutes | g . Paraclostridium
Firmicutes | g . Roseburia
Firmicutes | g . Butyricicoccus
Firmicutes | g . Flavonifractor
Firmicutes | g . Acidaminococcus
Firmicutes | g . Lachnospira
Firmicutes | g . UCG 002
Firmicutes | g . Faecalibacterium
Firmicutes | g . Lachnospiraceae ND3007 group
Firmicutes | g . Megasphaera
Firmicutes | g . Lachnospiraceae UCG 010
Firmicutes | g . Lachnoclostridium
Firmicutes | g . Colidextribacter
Firmicutes | g . Phocea
Firmicutes | g . Megamonas
Firmicutes | g . Dorea
Proteobacteria | g . Sutterella
Firmicutes | g . Holdemania
Firmicutes | g . Enterococcus
Firmicutes | g . Negativicoccus
Bacteroidota | g . Butyricimonas
Firmicutes | g . Agathobacter
Firmicutes | g . Candidatus Soleaferrea
Firmicutes | g . Coprococcus
not assign
Firmicutes | g . Christensenellaceae R 7 group
Actinobacteriota | g . Adlercreutzia
Firmicutes | g . Blautia
Firmicutes | g . Fusicatenibacter
Proteobacteria | g . Klebsiella
Firmicutes | g . Erysipelotrichaceae UCG 003
Actinobacteriota | g . Collinsella
Firmicutes | g . Subdoligranulum
Firmicutes | g . Holdemanella
Firmicutes | g . Ruminococcus
Firmicutes | g . Clostridium sensu stricto 1
Bacteroidota | g . Alistipes
Firmicutes | g . NK4A214 group
Firmicutes | g . Incertae Sedis
Firmicutes | g . Caproiciproducens
Firmicutes | g . UBA1819
Bacteroidota | g . Barnesiella
Firmicutes | g . Erysipelatoclostridium
Firmicutes | g . UC5 1 2E3
Actinobacteriota | g . Eggerthella
Firmicutes | g . Oscillospira
Bacteroidota | g . Coprobacter
Cooking method
Boiled
Grilled
Raw
Roasted
−4
−2
0
2
log2FC

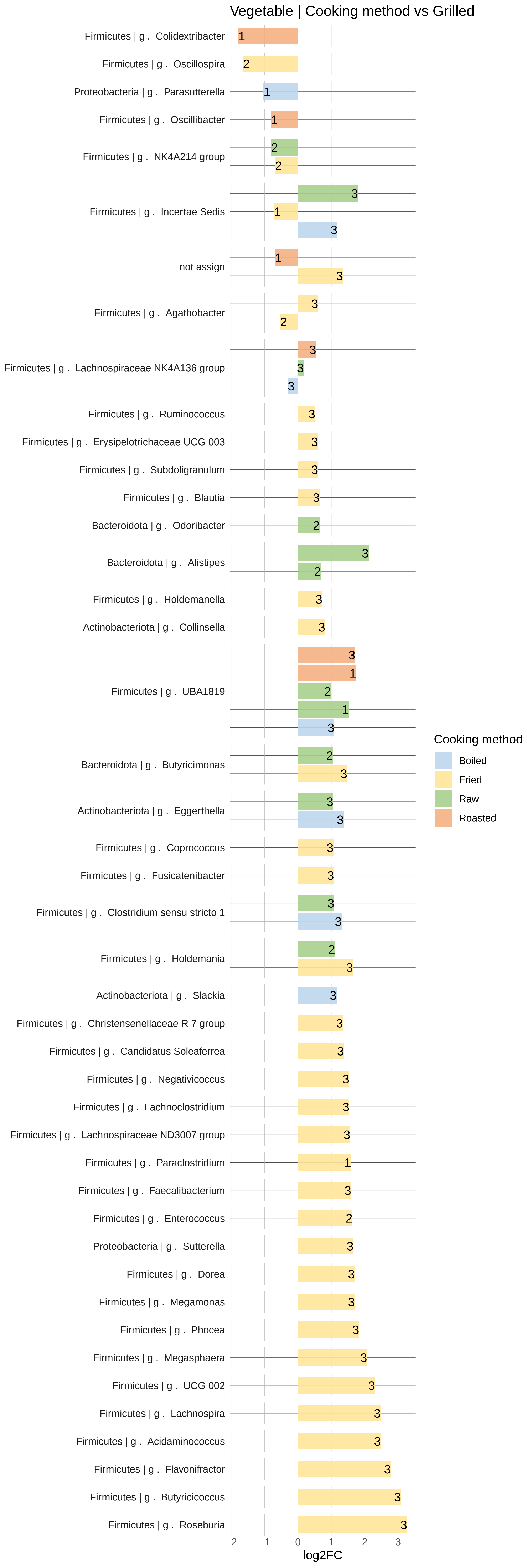
Vegetable | Cooking method vs Grilled
Firmicutes | g . Colidextribacter
Firmicutes | g . Oscillospira
Proteobacteria | g . Parasutterella
Firmicutes | g . Oscillibacter
Firmicutes | g . NK4A214 group
Firmicutes | g . Incertae Sedis
not assign
Firmicutes | g . Agathobacter
Firmicutes | g . Lachnospiraceae NK4A136 group
Firmicutes | g . Ruminococcus
Firmicutes | g . Erysipelotrichaceae UCG 003
Firmicutes | g . Subdoligranulum
Firmicutes | g . Blautia
Bacteroidota | g . Odoribacter
Bacteroidota | g . Alistipes
Firmicutes | g . Holdemanella
Actinobacteriota | g . Collinsella
Firmicutes | g . UBA1819
Bacteroidota | g . Butyricimonas
Actinobacteriota | g . Eggerthella
Firmicutes | g . Coprococcus
Firmicutes | g . Fusicatenibacter
Firmicutes | g . Clostridium sensu stricto 1
Firmicutes | g . Holdemania
Actinobacteriota | g . Slackia
Firmicutes | g . Christensenellaceae R 7 group
Firmicutes | g . Candidatus Soleaferrea
Firmicutes | g . Negativicoccus
Firmicutes | g . Lachnoclostridium
Firmicutes | g . Lachnospiraceae ND3007 group
Firmicutes | g . Paraclostridium
Firmicutes | g . Faecalibacterium
Firmicutes | g . Enterococcus
Proteobacteria | g . Sutterella
Firmicutes | g . Dorea
Firmicutes | g . Megamonas
Firmicutes | g . Phocea
Firmicutes | g . Megasphaera
Firmicutes | g . UCG 002
Firmicutes | g . Lachnospira
Firmicutes | g . Acidaminococcus
Firmicutes | g . Flavonifractor
Firmicutes | g . Butyricicoccus
Firmicutes | g . Roseburia
−2
−1
0
1
2
3
log2FC
Cooking method
Boiled
Fried
Raw
Roasted

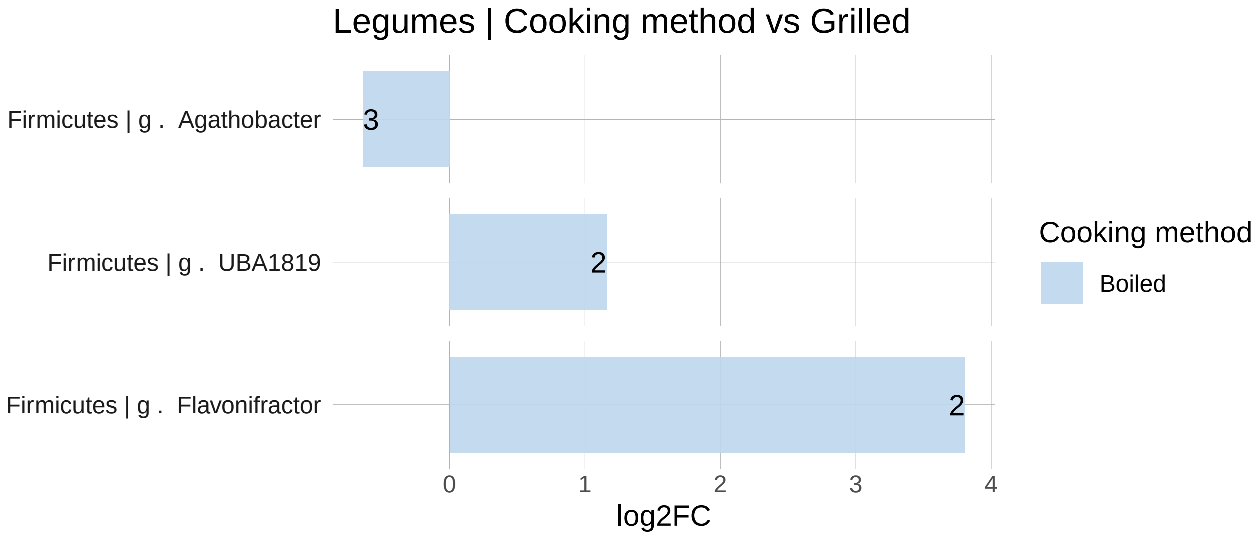

Legumes | Cooking method vs Grilled
Firmicutes | g . Agathobacter
Firmicutes | g . UBA1819
Firmicutes | g . Flavonifractor
3
2
2
0
1
2
3
4
log2FC
Cooking method
Boiled

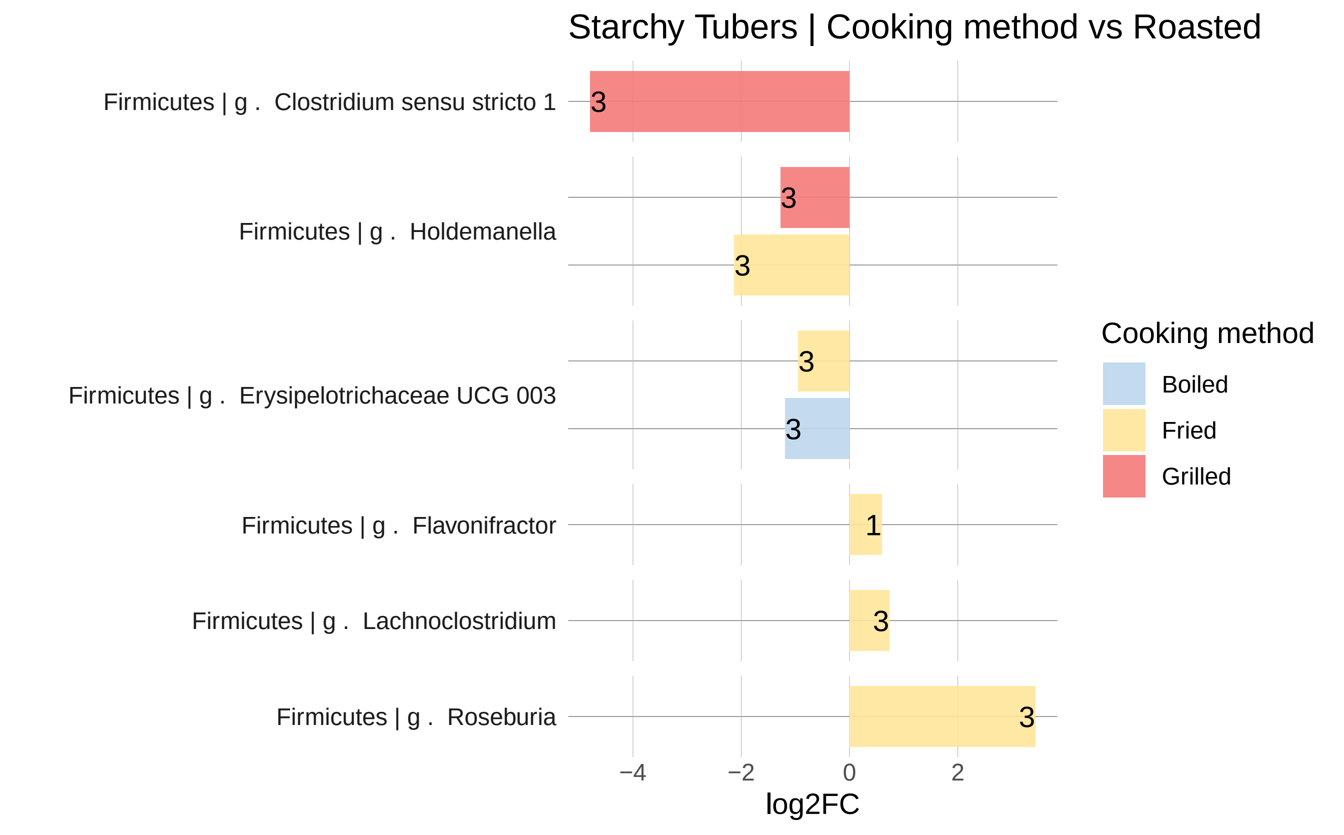

Starchy Tubers | Cooking method vs Roasted
Firmicutes | g . Clostridium sensu stricto 1
Firmicutes | g . Holdemanella
Firmicutes | g . Erysipelotrichaceae UCG 003
Firmicutes | g . Flavonifractor
Firmicutes | g . Lachnoclostridium
Firmicutes | g . Roseburia
3
3
3
3
3
1
3
3
−4
−2
0
2
log2FC
Cooking method
Boiled
Fried
Grilled

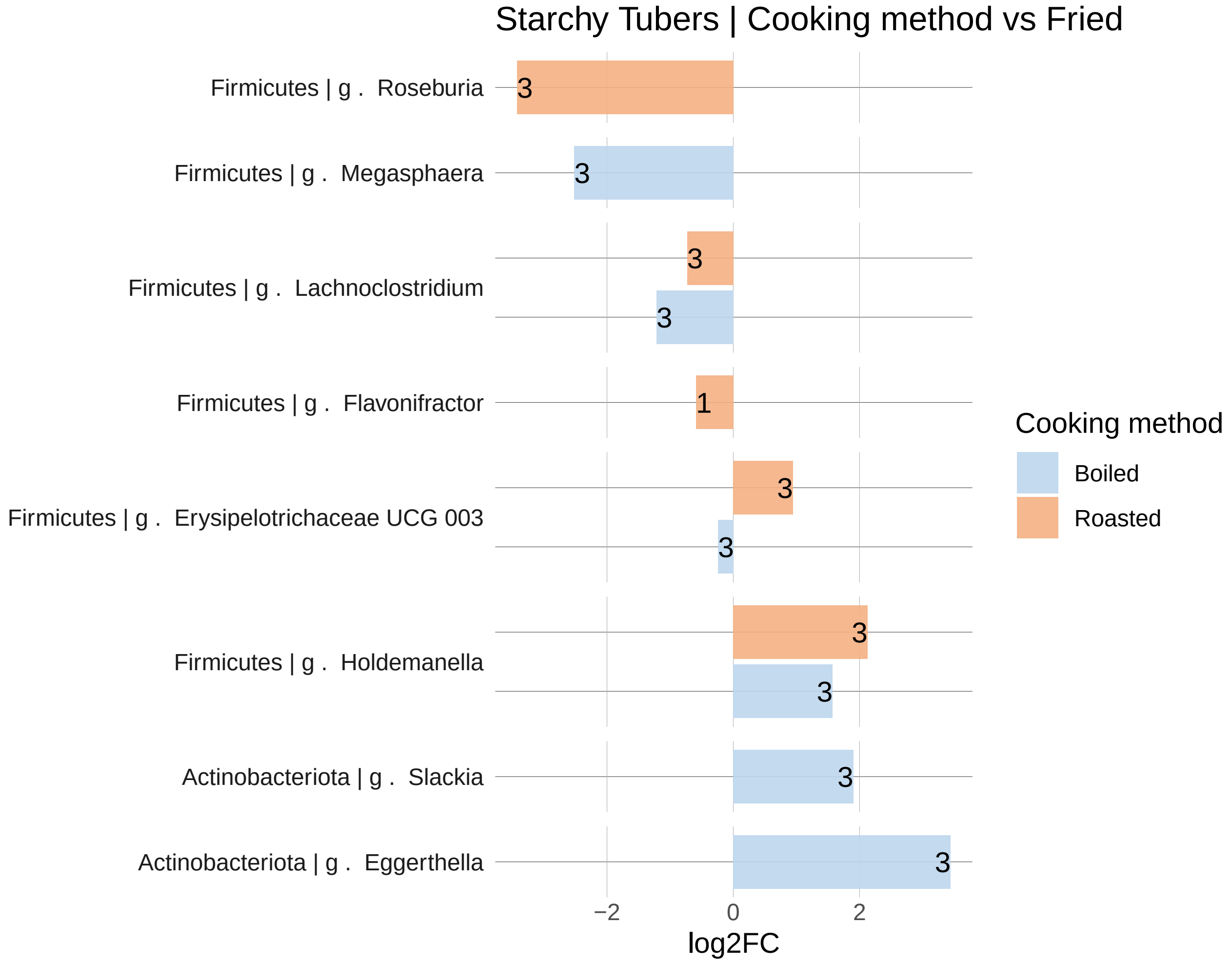

Starchy Tubers | Cooking method vs Fried
Firmicutes | g . Roseburia
Firmicutes | g . Megasphaera
Firmicutes | g . Lachnoclostridium
Firmicutes | g . Flavonifractor
Firmicutes | g . Erysipelotrichaceae UCG 003
Firmicutes | g . Holdemanella
Actinobacteriota | g . Slackia
Actinobacteriota | g . Eggerthella
3
3
3
3
1
3
3
3
3
3
3
−2
0
2
log2FC
Cooking method
Boiled
Roasted

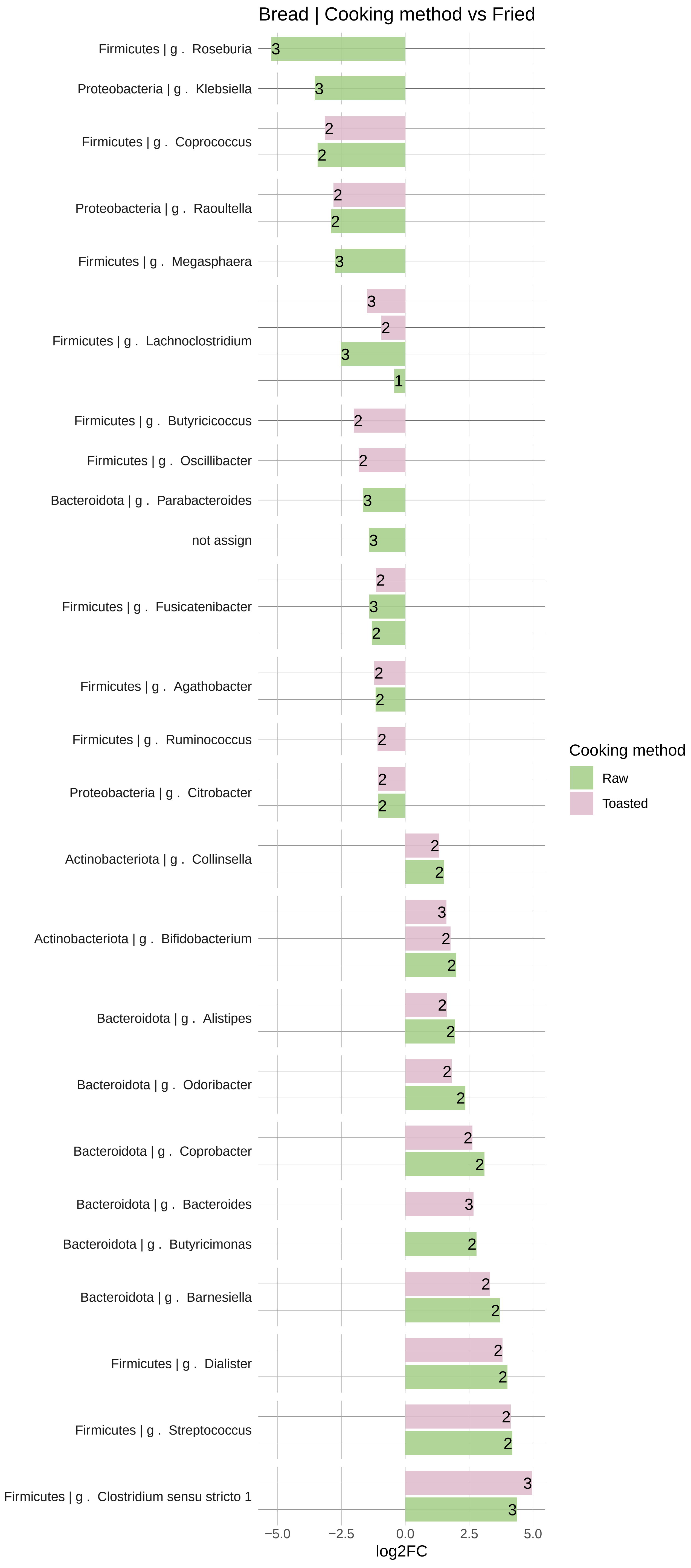

Bread | Cooking method vs Fried
Firmicutes | g . Roseburia
Proteobacteria | g . Klebsiella
Firmicutes | g . Coprococcus
Proteobacteria | g . Raoultella
Firmicutes | g . Megasphaera
Firmicutes | g . Lachnoclostridium
Firmicutes | g . Butyricicoccus
Firmicutes | g . Oscillibacter
Bacteroidota | g . Parabacteroides
not assign
Firmicutes | g . Fusicatenibacter
Firmicutes | g . Agathobacter
Firmicutes | g . Ruminococcus
Proteobacteria | g . Citrobacter
Actinobacteriota | g . Collinsella
Actinobacteriota | g . Bifidobacterium
Bacteroidota | g . Alistipes
Bacteroidota | g . Odoribacter
Bacteroidota | g . Coprobacter
Bacteroidota | g . Bacteroides
Bacteroidota | g . Butyricimonas
Bacteroidota | g . Barnesiella
Firmicutes | g . Dialister
Firmicutes | g . Streptococcus
Firmicutes | g . Clostridium sensu stricto 1
−5.0
−2.5
0.0
2.5
5.0
log2FC
Cooking method
Raw
Toasted
