# Supplementary material for: Effects of different foods and cooking methods on the gut microbiota: an in vitro approach
Source: Front Microbiol. 2024 Jan 8;14:1334623. doi: 10.3389/fmicb.2023.1334623 (PMC10800916; doi:10.3389/fmicb.2023.1334623)
Supplement: Supplementary Figure 6 — Fold-changes in relative abundance of bacterial species between cooking methods per food category. Fold-changes are represented as log2(FC). Significant comparisons (q < 0.05) according to ANCOM for each individual are represented. Cooking methods are shown in different colors and the individuals in which a given comparison is significant are indicated (PDF). [file Data_Sheet_6.PDF]

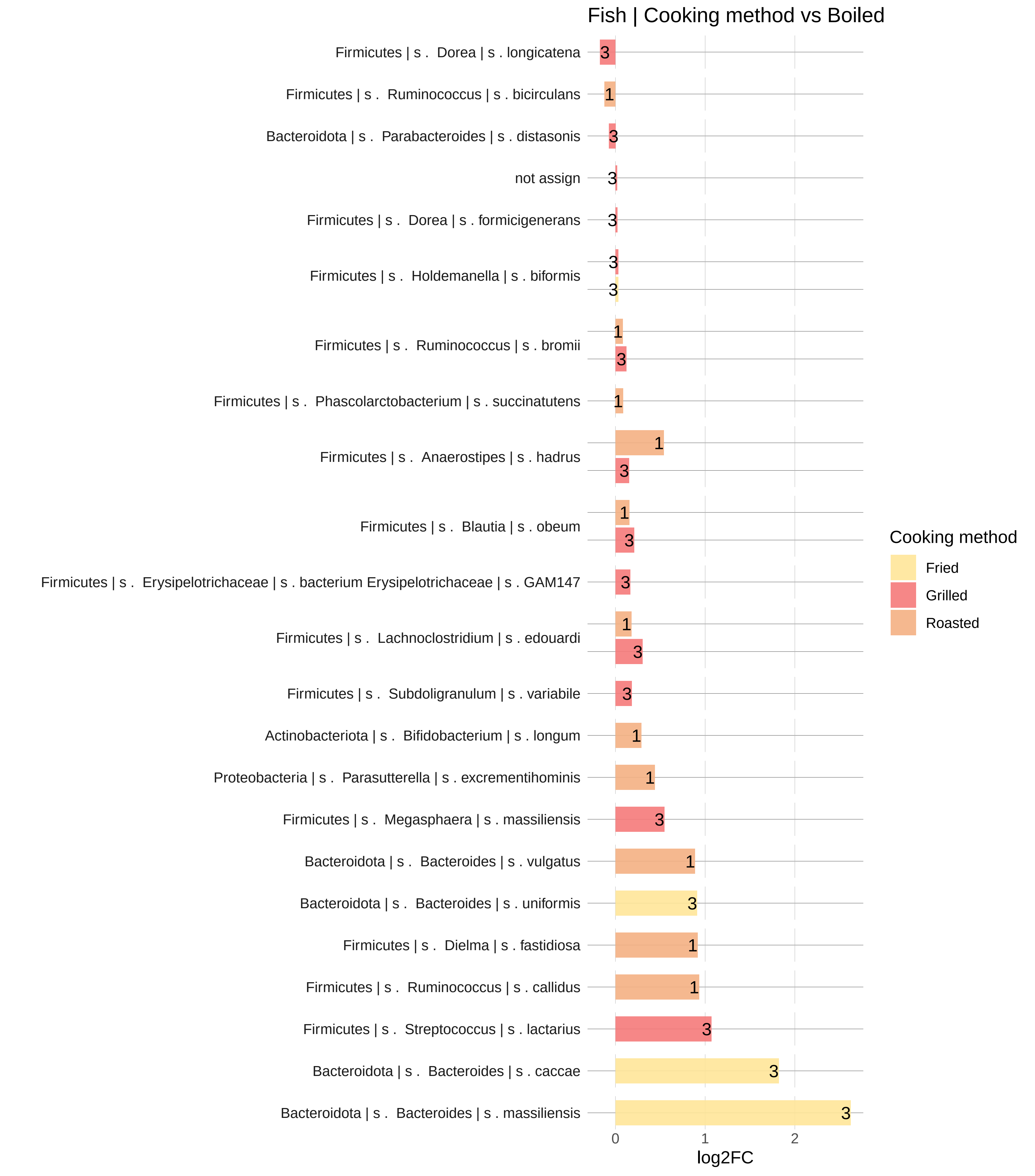
Fish | Cooking method vs Boiled
Firmicutes | s . Dorea | s . longicatena
Firmicutes | s . Ruminococcus | s . bicirculans
Bacteroidota | s . Parabacteroides | s . distasonis
not assign
Firmicutes | s . Dorea | s . formicigenerans
Firmicutes | s . Holdemanella | s . biformis
Firmicutes | s . Ruminococcus | s . bromii
Firmicutes | s . Phascolarctobacterium | s . succinatutens
Firmicutes | s . Anaerostipes | s . hadrus
Firmicutes | s . Blautia | s . obeum
Firmicutes | s . Erysipelotrichaceae | s . bacterium Erysipelotrichaceae | s . GAM147
Firmicutes | s . Lachnoclostridium | s . edouardi
Firmicutes | s . Subdoligranulum | s . variabile
Actinobacteriota | s . Bifidobacterium | s . longum
Proteobacteria | s . Parasutterella | s . excrementihominis
Firmicutes | s . Megasphaera | s . massiliensis
Bacteroidota | s . Bacteroides | s . vulgatus
Bacteroidota | s . Bacteroides | s . uniformis
Firmicutes | s . Dielma | s . fastidiosa
Firmicutes | s . Ruminococcus | s . callidus
Firmicutes | s . Streptococcus | s . lactarius
Bacteroidota | s . Bacteroides | s . caccae
Bacteroidota | s . Bacteroides | s . massiliensis
0
1
2
log2FC
Cooking method
Fried
Grilled
Roasted

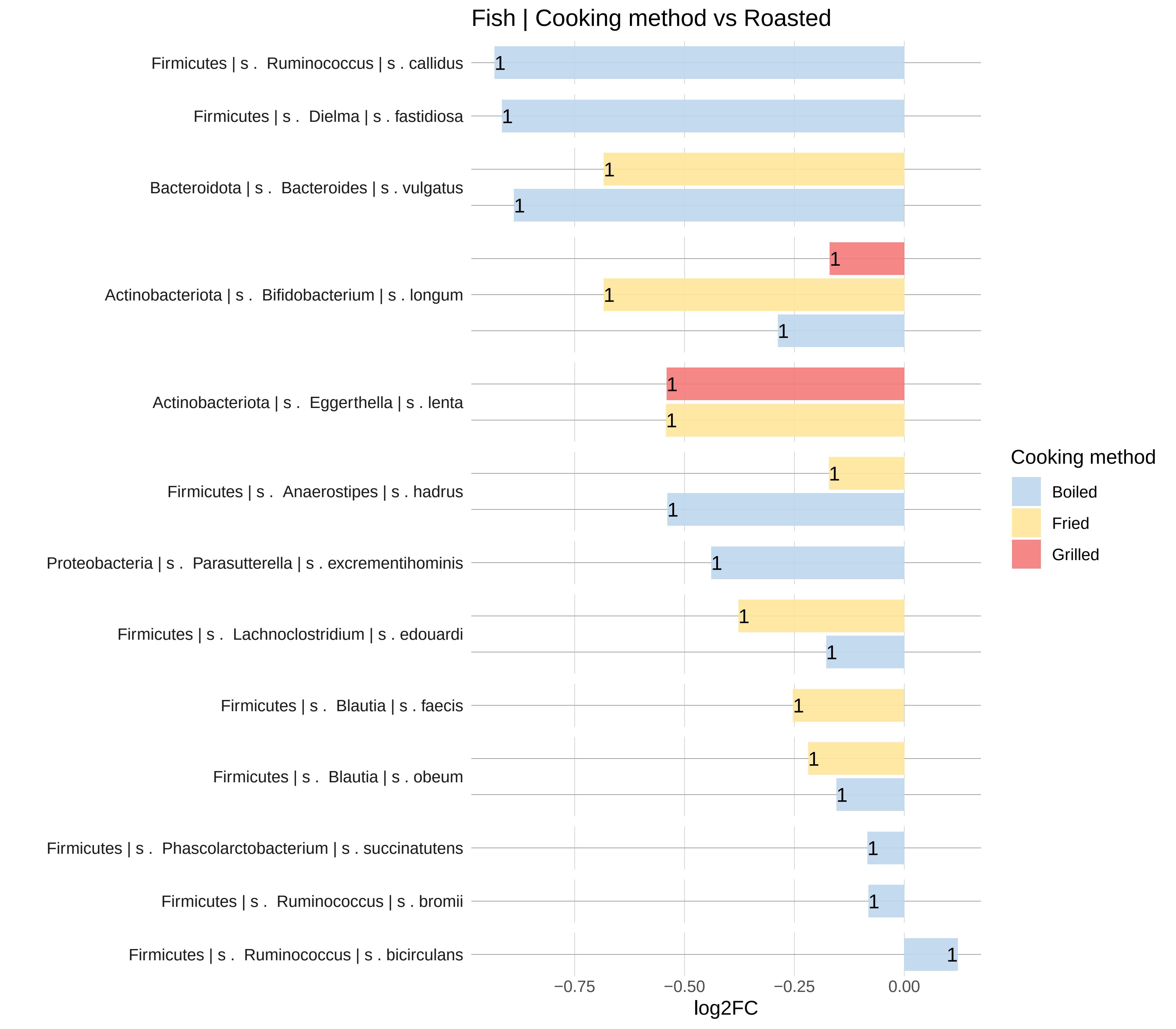

Fish | Cooking method vs Roasted
Firmicutes | s . Ruminococcus | s . callidus
Firmicutes | s . Dielma | s . fastidiosa
Bacteroidota | s . Bacteroides | s . vulgatus
Actinobacteriota | s . Bifidobacterium | s . longum
Actinobacteriota | s . Eggerthella | s . lenta
Firmicutes | s . Anaerostipes | s . hadrus
Proteobacteria | s . Parasutterella | s . excrementihominis
Firmicutes | s . Lachnoclostridium | s . edouardi
Firmicutes | s . Blautia | s . faecis
Firmicutes | s . Blautia | s . obeum
Firmicutes | s . Phascolarctobacterium | s . succinatutens
Firmicutes | s . Ruminococcus | s . bromii
Firmicutes | s . Ruminococcus | s . bicirculans
−0.75
−0.50
−0.25
0.00
log2FC
Cooking method
Boiled
Fried
Grilled

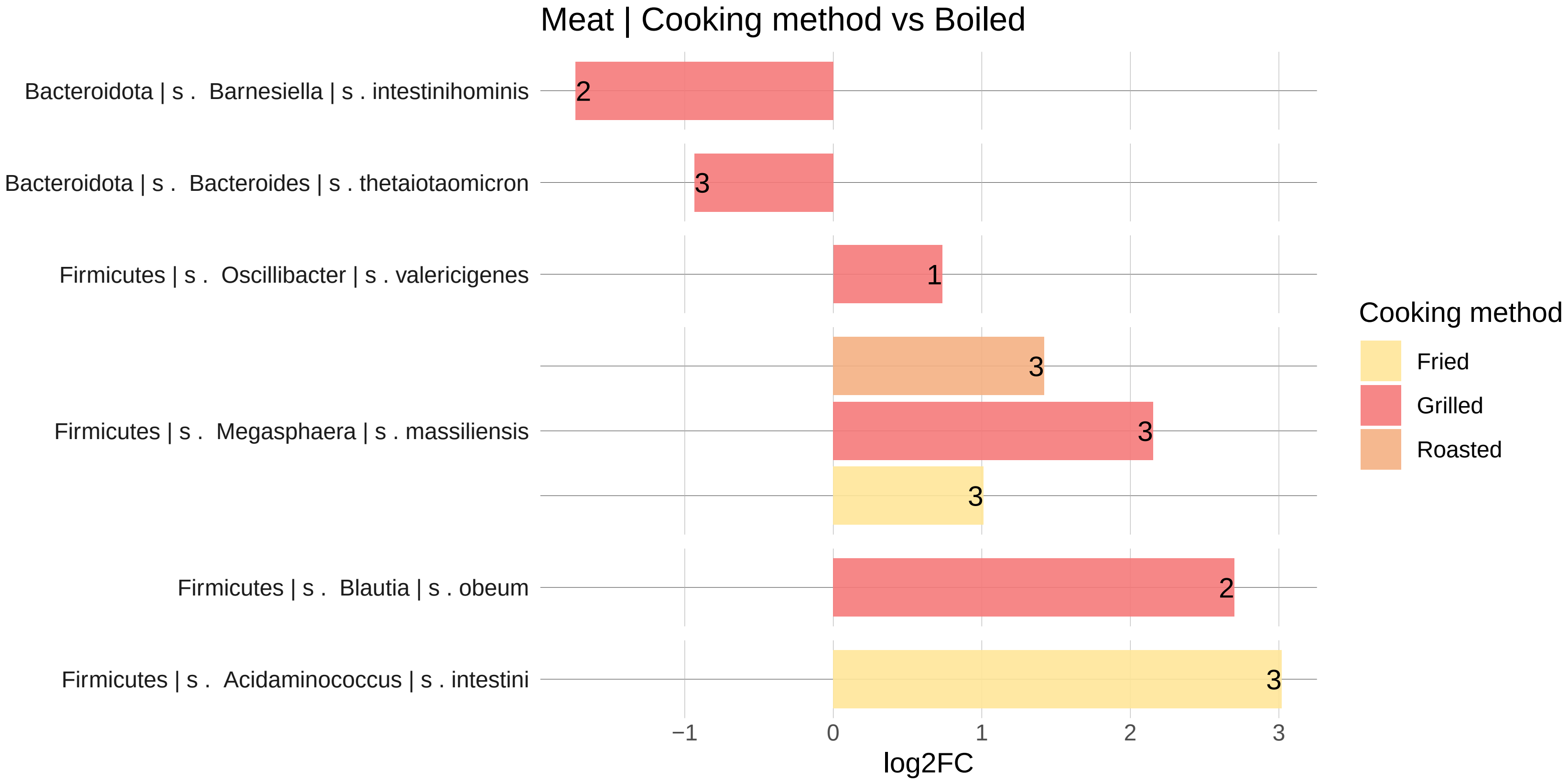
Meat | Cooking method vs Boiled
Bacteroidota | s . Barnesiella | s . intestinihominis
Bacteroidota | s . Bacteroides | s . thetaiotaomicron
Firmicutes | s . Oscillibacter | s . valericigenes
Firmicutes | s . Megasphaera | s . massiliensis
Firmicutes | s . Blautia | s . obeum
Firmicutes | s . Acidaminococcus | s . intestini
2
3
1
3
3
3
2
3
−1
0
1
2
3
log2FC
Cooking method
Fried
Grilled
Roasted

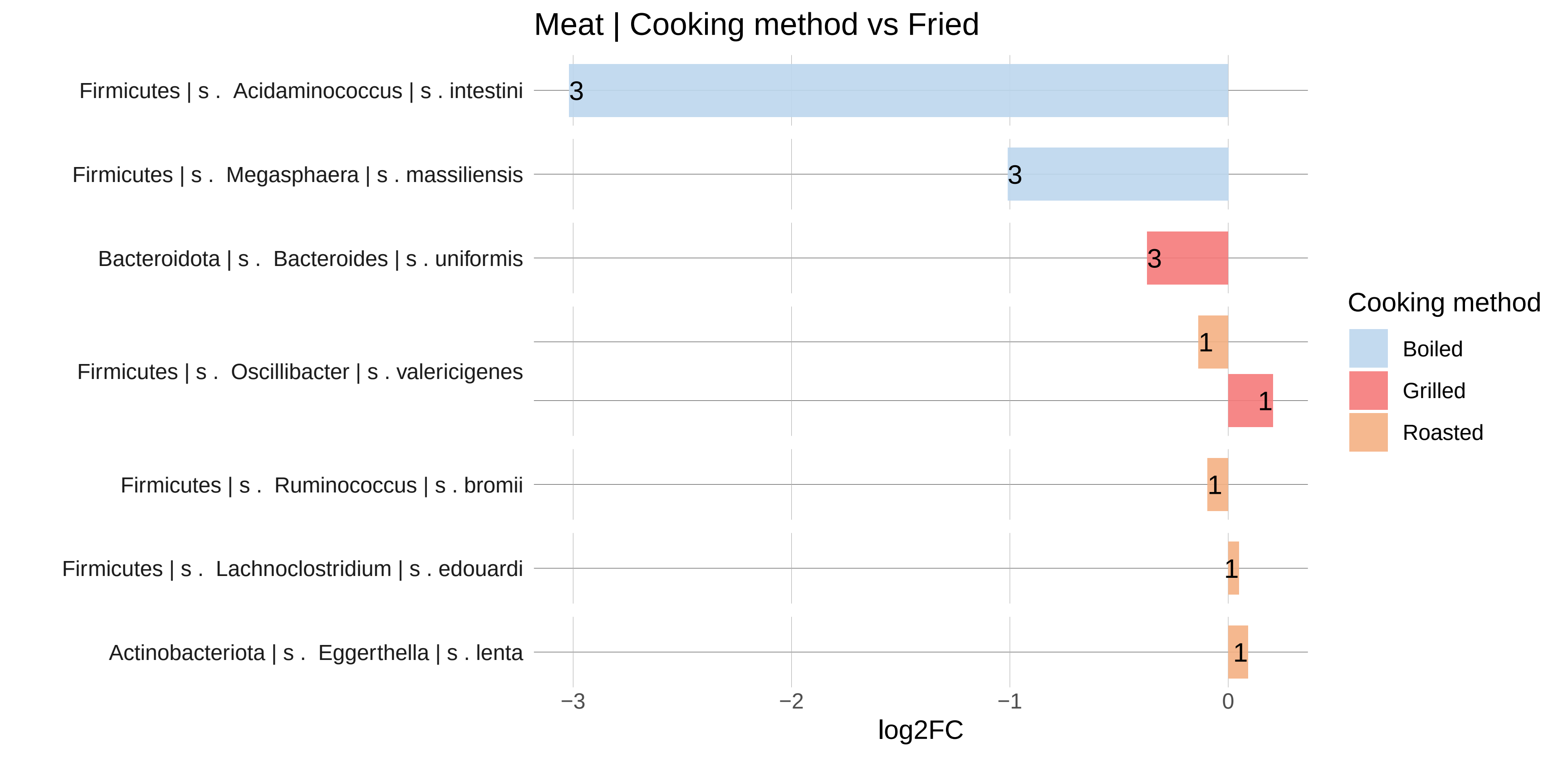

Meat | Cooking method vs Fried
Firmicutes | s . Acidaminococcus | s . intestini
Firmicutes | s . Megasphaera | s . massiliensis
Bacteroidota | s . Bacteroides | s . uniformis
Firmicutes | s . Oscillibacter | s . valericigenes
Firmicutes | s . Ruminococcus | s . bromii
Firmicutes | s . Lachnoclostridium | s . edouardi
Actinobacteriota | s . Eggerthella | s . lenta
3
3
3
1
1
1
1
1
−3
−2
−1
0
log2FC
Cooking method
Boiled
Grilled
Roasted

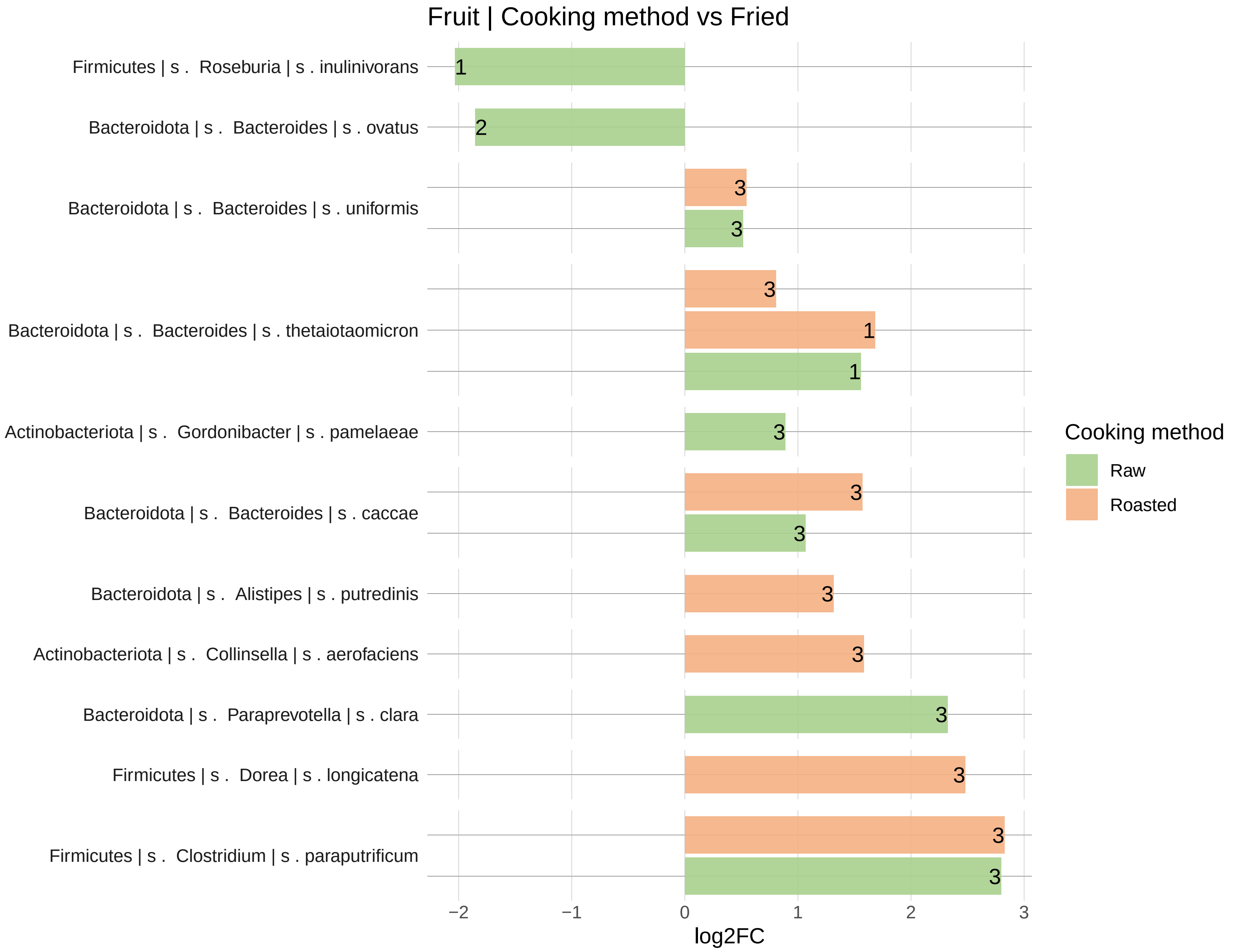

Fruit | Cooking method vs Fried
Firmicutes | s . Roseburia | s . inulinivorans
Bacteroidota | s . Bacteroides | s . ovatus
Bacteroidota | s . Bacteroides | s . uniformis
Bacteroidota | s . Bacteroides | s . thetaiotaomicron
Actinobacteriota | s . Gordonibacter | s . pamelaeae
Bacteroidota | s . Bacteroides | s . caccae
Bacteroidota | s . Alistipes | s . putredinis
Actinobacteriota | s . Collinsella | s . aerofaciens
Bacteroidota | s . Paraprevotella | s . clara
Firmicutes | s . Dorea | s . longicatena
Firmicutes | s . Clostridium | s . paraputrificum
−2
−1
0
1
2
3
log2FC
Cooking method
Raw
Roasted

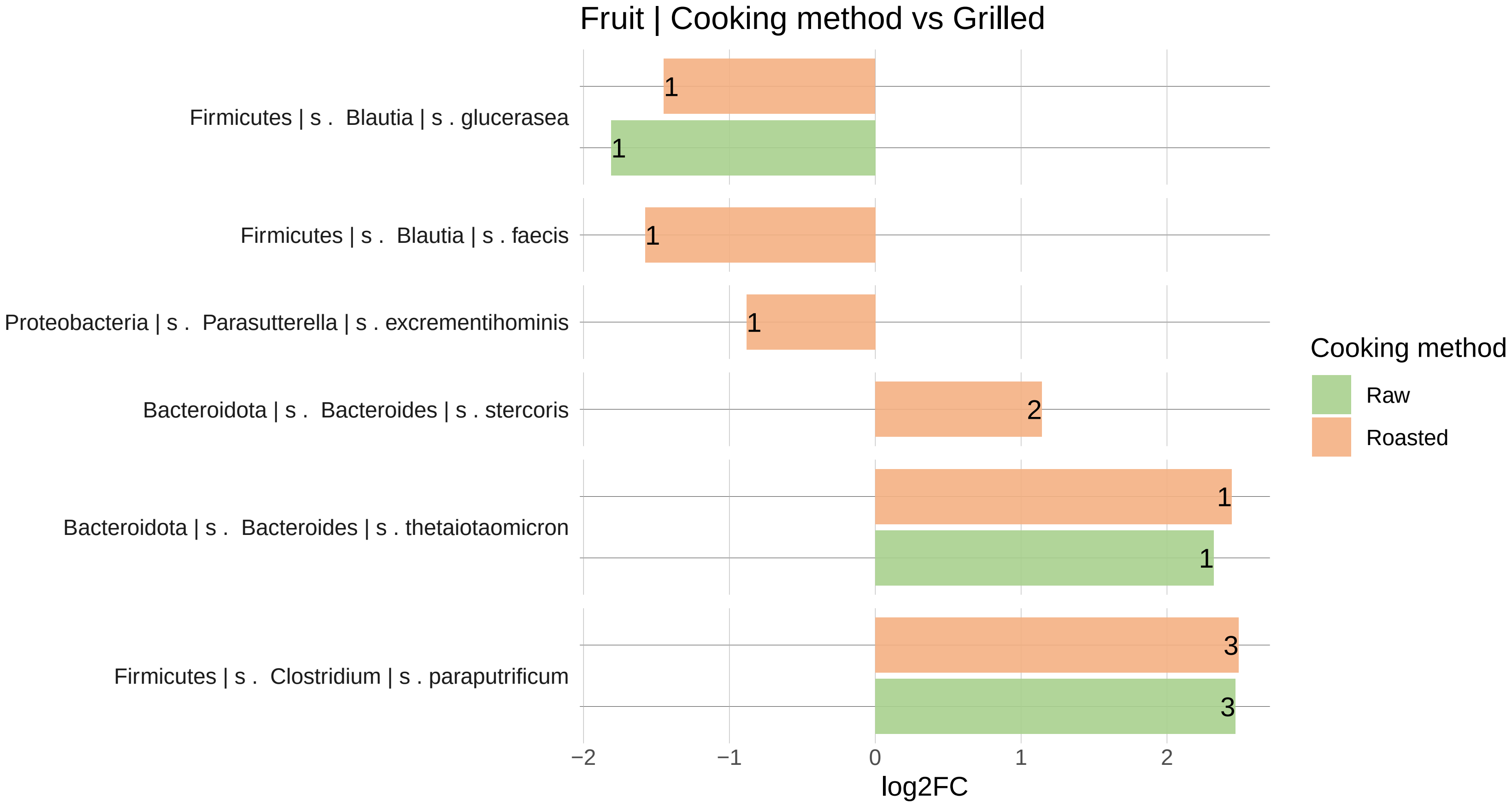

Fruit | Cooking method vs Grilled
Firmicutes | s . Blautia | s . glucerasea
Firmicutes | s . Blautia | s . faecis
Proteobacteria | s . Parasutterella | s . excrementihominis
Bacteroidota | s . Bacteroides | s . stercoris
Bacteroidota | s . Bacteroides | s . thetaiotaomicron
Firmicutes | s . Clostridium | s . paraputrificum
1
1
1
1
2
1
1
3
3
−2
−1
0
1
2
log2FC
Cooking method
Raw
Roasted

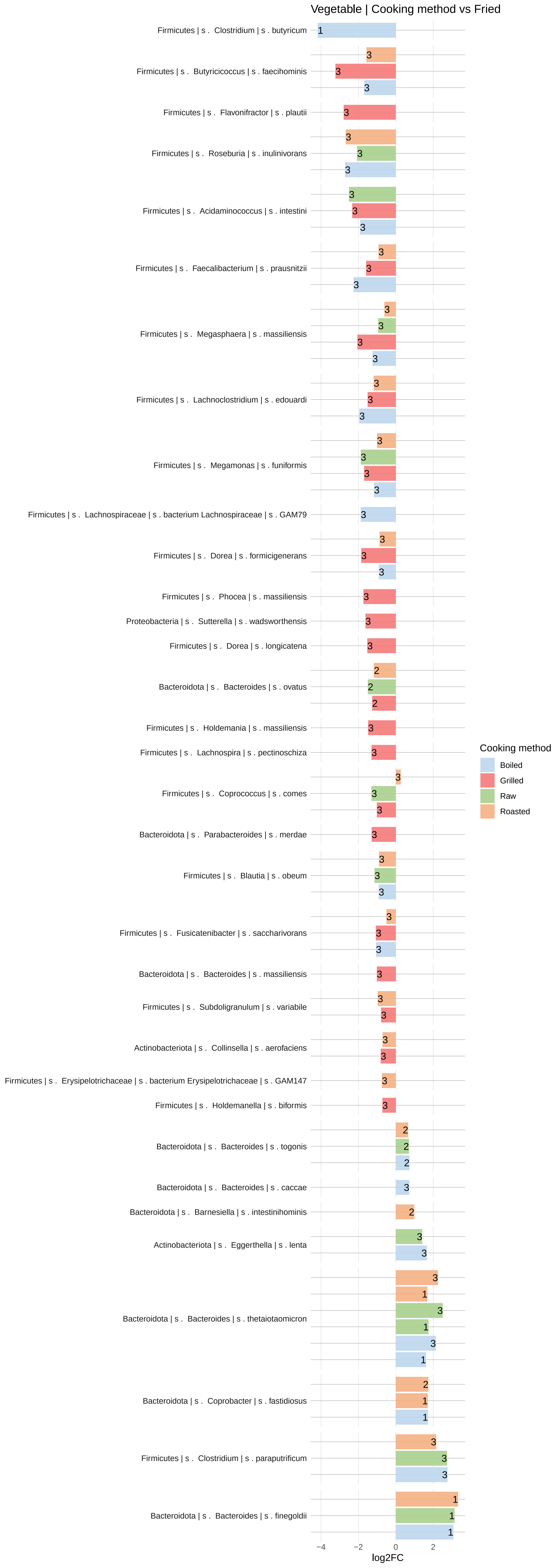

Vegetable | Cooking method vs Fried
Firmicutes | s . Clostridium | s . butyricum
Firmicutes | s . Butyricicoccus | s . faecihominis
Firmicutes | s . Flavonifractor | s . plautii
Firmicutes | s . Roseburia | s . inulinivorans
Firmicutes | s . Acidaminococcus | s . intestini
Firmicutes | s . Faecalibacterium | s . prausnitzii
Firmicutes | s . Megasphaera | s . massiliensis
Firmicutes | s . Lachnoclostridium | s . edouardi
Firmicutes | s . Megamonas | s . funiformis
Firmicutes | s . Lachnospiraceae | s . bacterium Lachnospiraceae | s . GAM79
Firmicutes | s . Dorea | s . formicigenerans
Firmicutes | s . Phocea | s . massiliensis
Proteobacteria | s . Sutterella | s . wadsworthensis
Firmicutes | s . Dorea | s . longicatena
Bacteroidota | s . Bacteroides | s . ovatus
Firmicutes | s . Holdemania | s . massiliensis
Firmicutes | s . Lachnospira | s . pectinoschiza
Firmicutes | s . Coprococcus | s . comes
Bacteroidota | s . Parabacteroides | s . merdae
Firmicutes | s . Blautia | s . obeum
Firmicutes | s . Fusicatenibacter | s . saccharivorans
Bacteroidota | s . Bacteroides | s . massiliensis
Firmicutes | s . Subdoligranulum | s . variabile
Actinobacteriota | s . Collinsella | s . aerofaciens
Firmicutes | s . Erysipelotrichaceae | s . bacterium Erysipelotrichaceae | s . GAM147
Firmicutes | s . Holdemanella | s . biformis
Bacteroidota | s . Bacteroides | s . togonis
Bacteroidota | s . Bacteroides | s . caccae
Bacteroidota | s . Barnesiella | s . intestinihominis
Actinobacteriota | s . Eggerthella | s . lenta
Bacteroidota | s . Bacteroides | s . thetaiotaomicron
Bacteroidota | s . Coprobacter | s . fastidiosus
Firmicutes | s . Clostridium | s . paraputrificum
Bacteroidota | s . Bacteroides | s . finegoldii
−4
−2
0
2
log2FC
Cooking method
Boiled
Grilled
Raw
Roasted

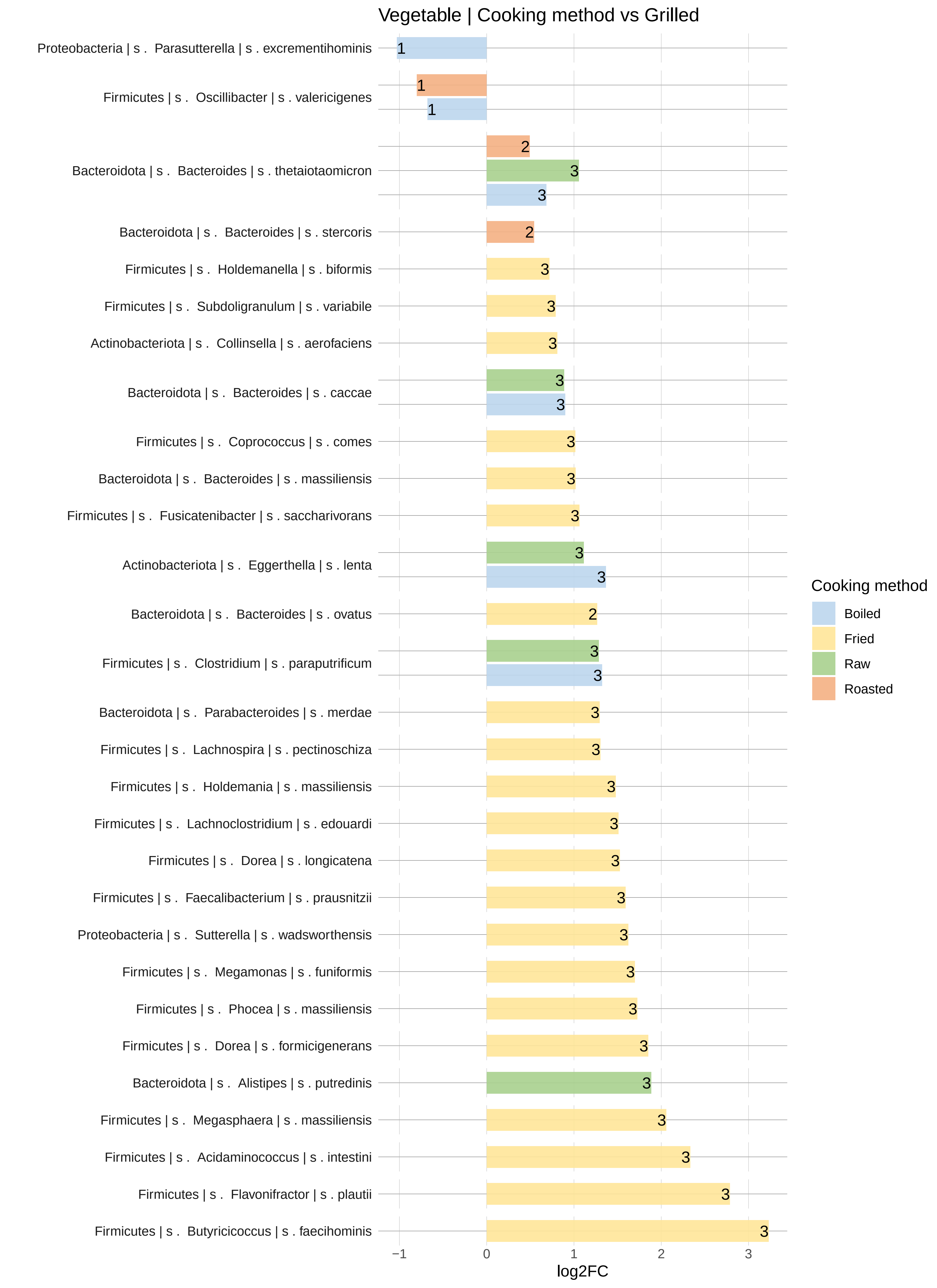

Vegetable | Cooking method vs Grilled
Proteobacteria | s . Parasutterella | s . excrementihominis
Firmicutes | s . Oscillibacter | s . valericigenes
Bacteroidota | s . Bacteroides | s . thetaiotaomicron
Bacteroidota | s . Bacteroides | s . stercoris
Firmicutes | s . Holdemanella | s . biformis
Firmicutes | s . Subdoligranulum | s . variabile
Actinobacteriota | s . Collinsella | s . aerofaciens
Bacteroidota | s . Bacteroides | s . caccae
Firmicutes | s . Coprococcus | s . comes
Bacteroidota | s . Bacteroides | s . massiliensis
Firmicutes | s . Fusicatenibacter | s . saccharivorans
Actinobacteriota | s . Eggerthella | s . lenta
Bacteroidota | s . Bacteroides | s . ovatus
Firmicutes | s . Clostridium | s . paraputrificum
Bacteroidota | s . Parabacteroides | s . merdae
Firmicutes | s . Lachnospira | s . pectinoschiza
Firmicutes | s . Holdemania | s . massiliensis
Firmicutes | s . Lachnoclostridium | s . edouardi
Firmicutes | s . Dorea | s . longicatena
Firmicutes | s . Faecalibacterium | s . prausnitzii
Proteobacteria | s . Sutterella | s . wadsworthensis
Firmicutes | s . Megamonas | s . funiformis
Firmicutes | s . Phocea | s . massiliensis
Firmicutes | s . Dorea | s . formicigenerans
Bacteroidota | s . Alistipes | s . putredinis
Firmicutes | s . Megasphaera | s . massiliensis
Firmicutes | s . Acidaminococcus | s . intestini
Firmicutes | s . Flavonifractor | s . plautii
Firmicutes | s . Butyricicoccus | s . faecihominis
−1
0
1
2
3
log2FC
Cooking method
Boiled
Fried
Raw
Roasted

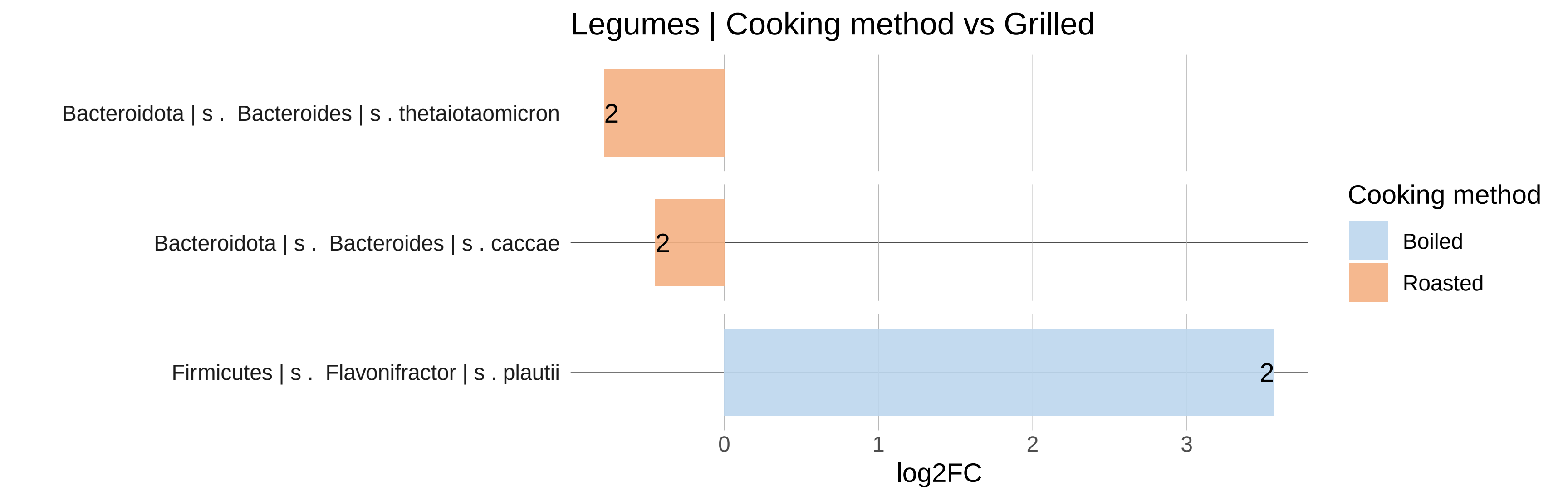

Legumes | Cooking method vs Grilled
Bacteroidota | s . Bacteroides | s . thetaiotaomicron
Bacteroidota | s . Bacteroides | s . caccae
Firmicutes | s . Flavonifractor | s . plautii
2
2
2
0
1
2
3
log2FC
Cooking method
Boiled
Roasted

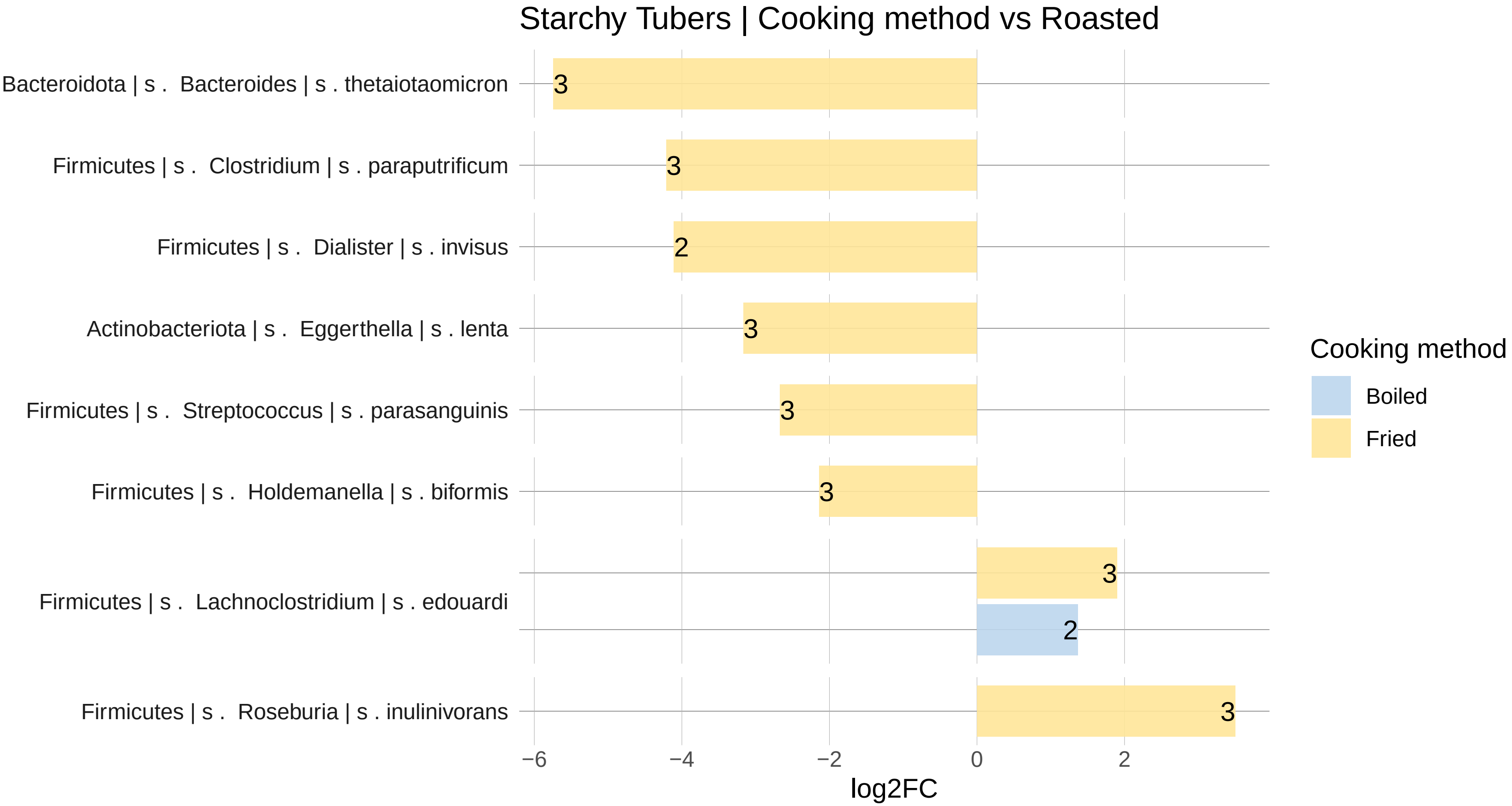
Starchy Tubers | Cooking method vs Roasted
Bacteroidota | s . Bacteroides | s . thetaiotaomicron
Firmicutes | s . Clostridium | s . paraputrificum
Firmicutes | s . Dialister | s . invisus
Actinobacteriota | s . Eggerthella | s . lenta
Firmicutes | s . Streptococcus | s . parasanguinis
Firmicutes | s . Holdemanella | s . biformis
Firmicutes | s . Lachnoclostridium | s . edouardi
Firmicutes | s . Roseburia | s . inulinivorans
3
3
2
3
3
3
3
2
3
−6
−4
−2
0
2
log2FC
Cooking method
Boiled
Fried

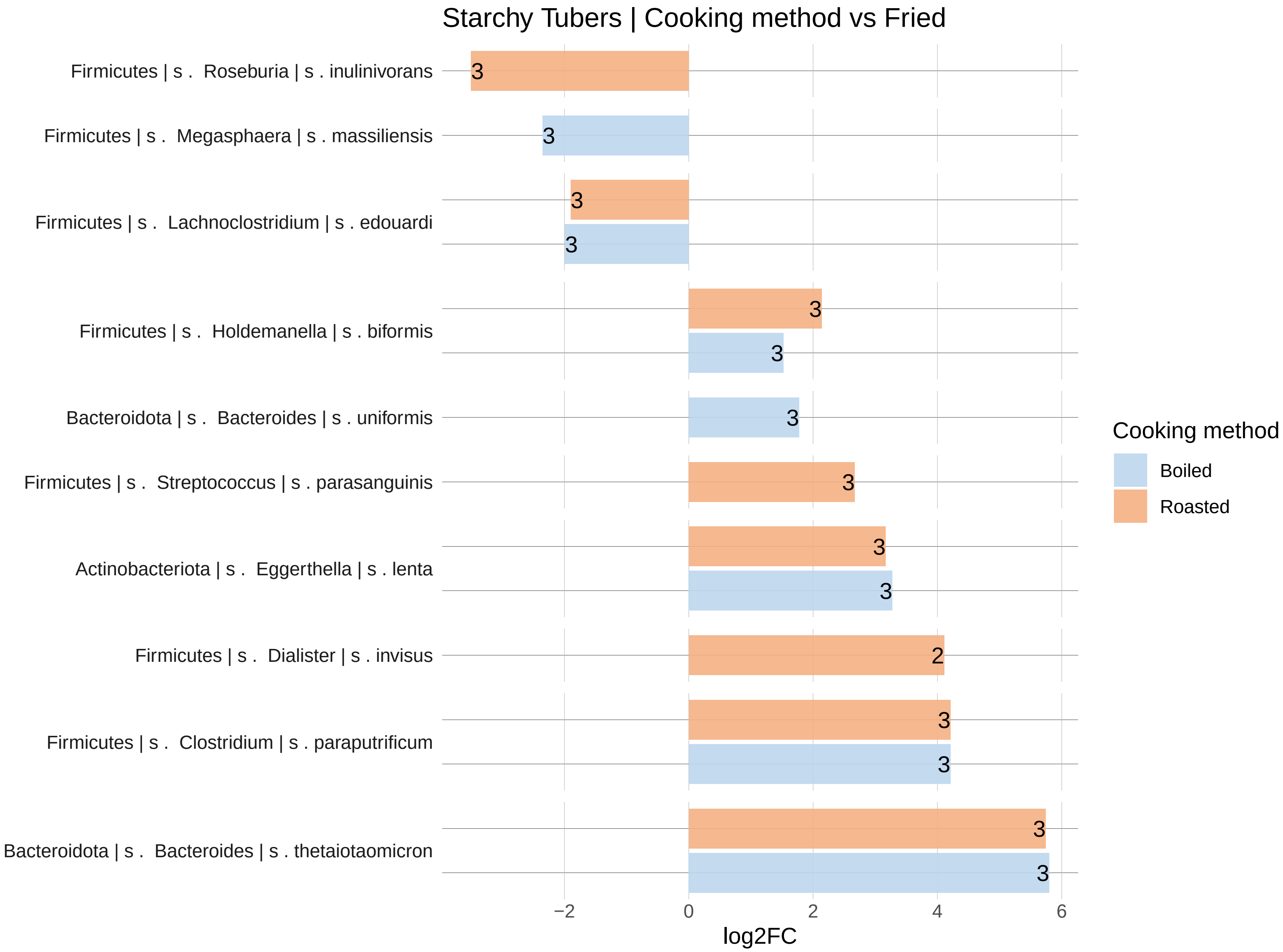

Starchy Tubers | Cooking method vs Fried
Firmicutes | s . Roseburia | s . inulinivorans
Firmicutes | s . Megasphaera | s . massiliensis
Firmicutes | s . Lachnoclostridium | s . edouardi
Firmicutes | s . Holdemanella | s . biformis
Bacteroidota | s . Bacteroides | s . uniformis
Firmicutes | s . Streptococcus | s . parasanguinis
Actinobacteriota | s . Eggerthella | s . lenta
Firmicutes | s . Dialister | s . invisus
Firmicutes | s . Clostridium | s . paraputrificum
Bacteroidota | s . Bacteroides | s . thetaiotaomicron
3
3
3
3
3
3
3
3
3
3
2
3
3
3
3
−2
0
2
4
6
log2FC
Cooking method
Boiled
Roasted

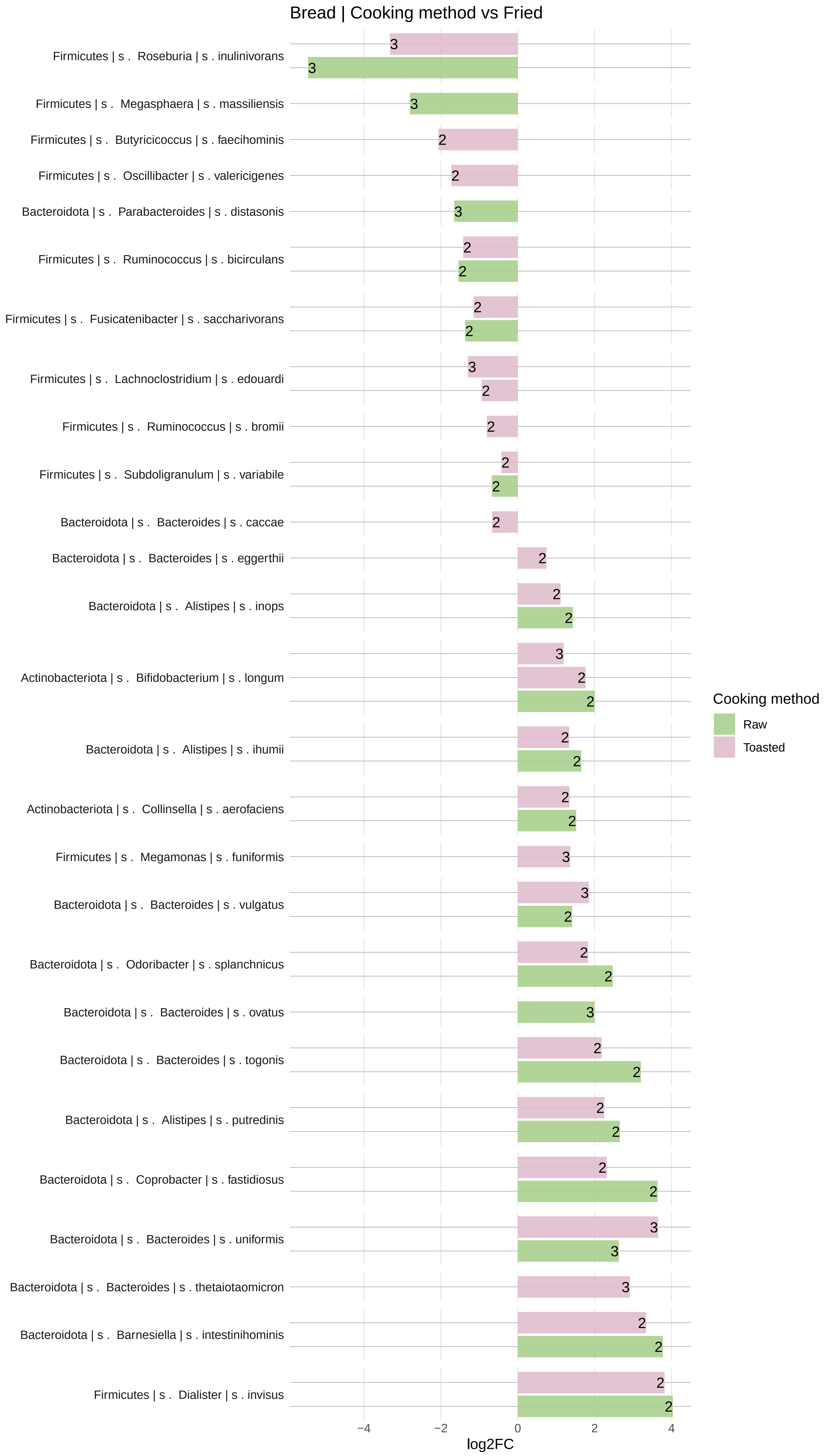
Bread | Cooking method vs Fried
Firmicutes | s . Roseburia | s . inulinivorans
Firmicutes | s . Megasphaera | s . massiliensis
Firmicutes | s . Butyricicoccus | s . faecihominis
Firmicutes | s . Oscillibacter | s . valericigenes
Bacteroidota | s . Parabacteroides | s . distasonis
Firmicutes | s . Ruminococcus | s . bicirculans
Firmicutes | s . Fusicatenibacter | s . saccharivorans
Firmicutes | s . Lachnoclostridium | s . edouardi
Firmicutes | s . Ruminococcus | s . bromii
Firmicutes | s . Subdoligranulum | s . variabile
Bacteroidota | s . Bacteroides | s . caccae
Bacteroidota | s . Bacteroides | s . eggerthii
Bacteroidota | s . Alistipes | s . inops
Actinobacteriota | s . Bifidobacterium | s . longum
Bacteroidota | s . Alistipes | s . ihumii
Actinobacteriota | s . Collinsella | s . aerofaciens
Firmicutes | s . Megamonas | s . funiformis
Bacteroidota | s . Bacteroides | s . vulgatus
Bacteroidota | s . Odoribacter | s . splanchnicus
Bacteroidota | s . Bacteroides | s . ovatus
Bacteroidota | s . Bacteroides | s . togonis
Bacteroidota | s . Alistipes | s . putredinis
Bacteroidota | s . Coprobacter | s . fastidiosus
Bacteroidota | s . Bacteroides | s . uniformis
Bacteroidota | s . Bacteroides | s . thetaiotaomicron
Bacteroidota | s . Barnesiella | s . intestinihominis
Firmicutes | s . Dialister | s . invisus
−4
−2
0
2
4
log2FC
Cooking method
Raw
Toasted
